# Catalytic 1,2‐Migratory Insertion in a Bismuth Redox Platform: Reductive Arylation of Aldehydes

**DOI:** 10.1002/anie.202510360

**Published:** 2025-08-06

**Authors:** Xiangrong Liu, Hye Won Moon, Davide Spinnato, Markus Leutzsch, Josep Cornella

**Affiliations:** ^1^ Max‐Planck‐Institut für Kohlenforschung Kaiser‐Wilhelm‐Platz 1 45470 Mülheim an der Ruhr Germany

**Keywords:** 1,2‐Migratory insertion, Arylation, Bismuth, Polyfluoroarene, Three‐component

## Abstract

Herein, we report a catalytic defluorinative arylation of aldehydes with (per) A fluoroarenes facilitated by a pincer‐based PheBox‐Bi(I) under mild conditions. The protocol features various novel aspects in bismuth redox catalysis; namely, (1) a catalytic 1,2‐aryl migratory insertion to forge a C─C bond, (2) an unprecedented example of multicomponent reaction through four elementary organometallic steps at a Bi center, (3) an unusual strategy for Bi(I) compounds regeneration via O─Si reductive elimination. Experimental and computational studies aided in dissecting the various mechanistic aspects of the bismuth redox cycle.

## Graphical abstract

We report a multicomponent reaction involving an aryl halide, an aldehyde, and a silane derivative, facilitated by low‐valent bismuth redox catalysis under mild conditions. The protocol represents an unprecedented example of four elementary organometallic steps at a Bi center within the catalytic cycle. Experimental and computational studies indicate the involvement of intermediate species that supports a Bi(I)/Bi(III) cycle.

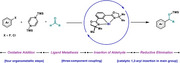

The 1,2‐addition of aryl groups to carbonyl compounds has become a cornerstone disconnection in organic chemistry with a profound synthetic relevance. A plethora of organometallic reagents have found their use in this approach, spearheaded by the well‐known Grignard and aryl‐lithium reagents (Figure 1a).^[^ ^1^, ^2^, ^3^, ^4^, ^5^, ^6^, ^7^, ^8^, ^9^, ^10^, ^11^, ^12^, ^13^ ^]^ However, for years, chemists have devoted efforts in finding alternatives that would overcome the intrinsically limited functional group tolerance and chemoselectivity issues associated with these strategies.^[^ ^14^, ^15^ ^]^ Approaches based on transition metals joined this field and remarkably expanded this coupling to milder aryl nucleophiles and structurally diverse aldehydes.^[^ ^6^, ^16^, ^17^, ^18^, ^19^ ^]^ Recently, transition metal catalysis also offered the possibility to bypass the use of organonucleophiles via reductive cross‐couplings.^[^ ^20^, ^21^, ^22^, ^23^, ^24^ ^]^ In this approach, organic halides and aldehydes can be coupled to forge the desired C─C bond aided by an external reducing agent.^[^ ^25^, ^26^, ^27^, ^28^ ^]^ Despite the breadth of opportunities with transition metals, this reactivity remains challenging within the field of main group catalysis, mainly due to the redox nature of the catalytic process (Figure 1b). Indeed, reductive Barbier‐type reactions with carbonyl‐type compounds have been reported, albeit largely limited to allylation and crotylation.^[^ ^29^, ^30^, ^31^, ^32^, ^33^, ^34^, ^35^ ^]^ As part of our ongoing efforts to apply the unique reactivity of bismuth in various catalytic redox manifolds,^[^ ^36^, ^37^, ^38^, ^39^, ^40^ ^]^ we envisaged that a catalytic 1,2‐arylation of aldehydes would be a suitable platform to expand the palette of opportunities of this element.^[^ ^41^, ^42^, ^43^ ^]^ Herein, we report on the ability of a low‐valent Bi(I) complex to engage in a reductive arylation of aldehydes using polyfluorinated aryl fluorides and chlorides to afford the desired arylated alcohols. The process combines various novel aspects in bismuth redox catalysis; namely an unprecedented catalytic 1,2‐aryl insertion reaction to forge a C─C bond embedded in a multicomponent coupling reaction featuring four organometallic steps.^[^ ^44^, ^45^ ^]^ It is also important to mention that the use of (per)fluorinated arenes as defluorinative coupling partners in the 1,2‐arylation of aldehydes is unique,^[^ ^46^, ^47^, ^48^, ^49^, ^50^, ^51^, ^52^ ^]^ thus highlighting the mechanistically distinct possibilities enabled by bismuth redox catalysis (Figure 1).

**Figure 1:**
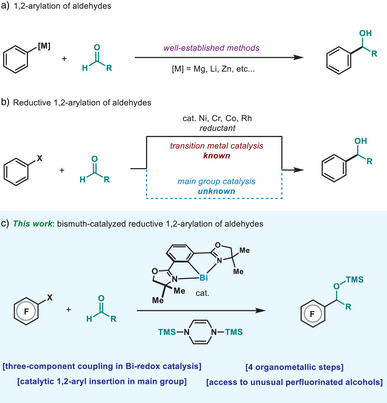
a) The venerable 1,2‐arylation of aldehydes. b) The upsurge of transition metal‐catalyzed reductive 1,2‐arylation using aryl halides. c) Reductive 1,2‐arylation of aldehydes through a low‐valent bismuth redox platform.

Based on our previous studies on the ability of Bi(I) complexes to activate C─F bonds,^[^ ^53^ ^]^ we selected fluorinated arene **1a** and aldehyde **2a** as model substrates to test the putative 1,2‐migratory insertion. TMS‐DHP **3a** was then chosen as a fluorophilic reductant due to the facile cleavage of Si─N bond driven by rearomatization.^[^ ^54^, ^55^, ^56^, ^57^ ^]^ After examination of the reaction parameters, 5 mol% of bismuthinidene **5a** excelled in catalyzing the defluorinative arylation of **2a** at 40 °C in MeCN, obtaining **4a** in 93% isolated yield (Table 1, entry 1). As expected from the reductive coupling, **4a** consists of a diarylalcohol protected with a TMS group. As shown in Table 1, both higher and lower temperatures resulted in lower yields of product (entries 2 and 3). Interestingly, concentration was also a parameter that needed control, as dilution resulted in a much lower yield of **4a** (entry 4). Although DMA could be used as solvent, the yields were markedly lower (entry 5). Control experiments verified the essential role of **5a** for the reaction to proceed; in the absence of Bi, no product was observed (entry 6). Similarly to our previous hydrodefluorination (HDF) reaction,^[^ ^53^ ^]^ the use of less nucleophilic bismuthinidene **5b** barely afforded the desired product (entry 7). Replacement of **5a** with simple BiCl_3_ was not successful (entry 8). The choice of the Si‐based reducing agent was important, as exemplified by the dramatically low yields obtained when using TMS‒TMS and DMPS‒DMPS (entries 9 and 10).^[^ ^58^, ^59^ ^]^ Finally, the use of exogenous fluoride anions does not trigger reactivity (entry 11).

**Table 1:** Optimization of the Bi‐catalyzed reductive arylation of aldehydes with perfluoroarenes.

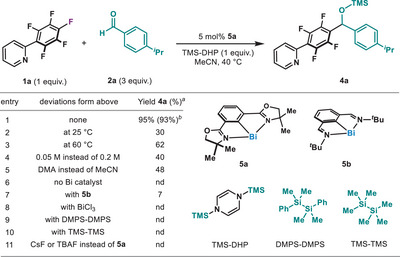

See Supporting Information for full experimental details. DMA: Dimethylacetamide; nd: not detected.

^a)^ Yields determined by quantitative ^1^H NMR yield using trichloroethene as internal standard on 0.05 mmol scale.

^b)^ Isolated yield on 0.20 mmol scale.

To investigate the generality of the process, we initially explored the scope of polyfluorinated arenes (Scheme 1). Pentafluorobenzenes substituted at the *para* position by heterocycles such as pyridine (**4a**, **4b**, and **4c**), pyrimidine (**4d**), thiophene (**4e**), or oxazoline (**4f**), all afforded the three‐component product in excellent yields. The presence of a phosphine did not lead to catalyst poisoning and **4g** was also obtained in excellent yields. When the *para*‐substituent was replaced by a simple Ph, the yield of **4h** was reduced to 38% even with extended reaction times. Notably, unsaturated functionalities such as alkenes (**4i**) and alkynes (**4j**) were tolerated with moderate yields. Octafluoronaphthalene also afforded the desired product **4k** in good yields. However, substrates with lower degrees of fluorination were unproductive under the optimized reaction conditions (Table S3). Interestingly, when replacing the F group with Cl, selective C─Cl cleavage occurred, thus yielding products **4a**, **4l**, and **4m** in moderate yields. These last examples showcase that the catalytic platform can be extended to aryl chlorides. The scope of the carbonyl partner revealed that various substituted benzaldehydes could be accommodated; *ortho*‐, *meta*‐, and *para*‐substituted methoxybenzaldehydes afforded the coupling products in excellent yields (**4n**, **4o**, and **4p**). Substitution of the aromatic ring with halogens was also amenable, as exemplified by **4q** and **4r**. The use of π‐extended aldehydes such as naphthalene‐2‐carbaldehyde or phenanthrene‐9‐carbaldehyde satisfactorily delivered the C─C coupled product (**4s** and **4u**). Heterocyclic scaffolds such as benzothiophene could be accommodated (**4v**). Finally, conjugated cinnamaldehyde was also included in acceptable yields (**4w**). With this protocol, tertiary aliphatic aldehydes such as pivalaldehyde and 1‐methylcyclohexane‐1‐carbaldehyde also engaged in the coupling (**4x** and **4y**), albeit in lower yields. It is worth pointing out that accommodation of aliphatic and hindered aldehydes is one of the main challenges in reductive couplings.^[^ ^60^, ^61^ ^]^ Addition to ketones as well as secondary and primary aliphatic aldehydes remained outside the scope of this protocol (see Supporting Information).

**Scheme 1:**
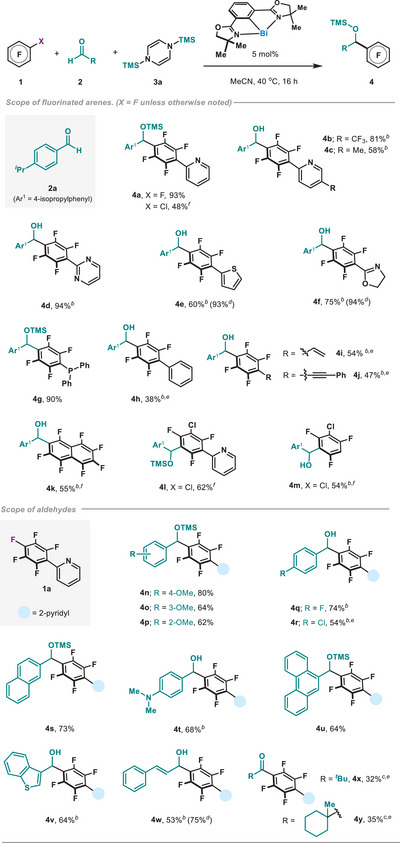
Scope of the Bi(I)‐catalyzed reductive arylation of aldehydes.*^a^* a) All yields are isolated on 0.20 mmol scale unless otherwise noted (see Supporting Information for full synthetic details). b) Isolated yields after TMS deprotection. c) Isolated yields after TMS deprotection and oxidation (see Supporting Information for details). d) Yields determined by quantitative ^1^H NMR spectroscopy before deprotection using trichloroethylene as internal standard. e) Reactions conducted for 72 h. f) 10 mol % of **5a**, 5.0 equiv of **2a** and 2.0 equiv of **3a** were used.

At this point, we conducted a series of stoichiometric experiments to shed light into the putative operating mechanism (Figure 2). Based on previous results from our laboratory,^[^ ^53^ ^]^ we speculated an initial C─F cleavage by **5a**. When polyfluoroarene **1a** and bismuthinidene **5a** were mixed at 40 °C in MeCN‐*d*_3_, the formation of deuterodefluorination product **6a** and the corresponding bismuth(III) difluoride byproduct **5d** was obtained. When the reaction was conducted in the presence of LiOTf to promote fluoride abstraction, adduct **5e** was identified as the major Bi‐based product, as judged by ^1^H NMR. HRMS analysis of an aliquot suggested the presence of **5f** in the reaction mixture, in agreement with our previous observations. Hence, **5a** is able to cleave the C─F bond of **1a**, leading to a highly reactive Bi(III)─Ar intermediate. Considering the recent reports on organobismuth(I) complexes engaging in radical processes,^[^ ^36^, ^37^, ^38^, ^39^, ^40^, ^62^, ^63^, ^64^ ^]^ as well as the observation of product **6a**, we evaluated the possibility of a radical pathway. Performing the reaction in the presence of TEMPO or BHT resulted in complete suppression of the reactivity, due to their inherent side reactions with **5a** and **3a**, respectively. Nevertheless, the reaction proceeded with slight loss of yield in the presence of DPE, without radical trapping product being observed. Moreover, the experimentally measured *E*_1/2_ of **5a** under the reaction conditions is ca. 1.4 V more positive than the reduction onset potential of **1a**, thus precluding a net oxidative addition initiated by single‐electron transfer from the bismuth complex.^[^ ^65^ ^]^ Based on these data, we postulate that **5a** cleaves the C─F bond in **1a** through an S_N_Ar‐type oxidative addition pathway. Formation of **6a** originates from the reaction of an extremely polarized and basic Bi(III)‒Ar able to deprotonate MeCN.^[^ ^66^ ^]^

**Figure 2:**
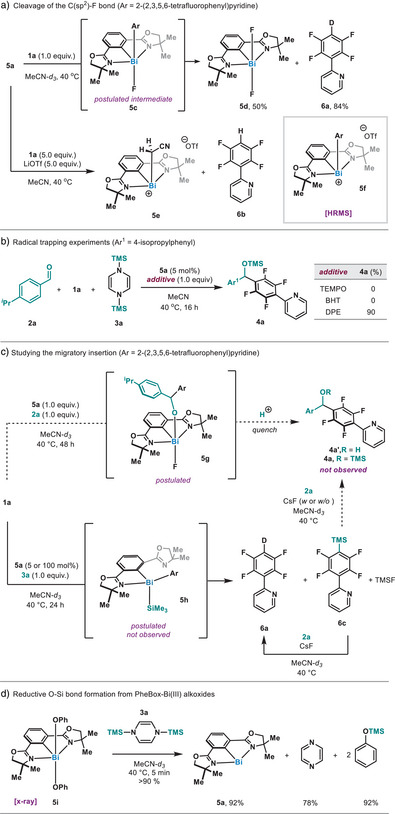
a) OA of **5a** with **1a**. b) Experiments with radical scavengers. c) Investigations into the migratory insertion process. d) O─Si bond formation from bismuth(III) alkoxides.

One of the most intriguing aspects of the transformation is the C─C bond formation, as it represents an unprecedented formal 1,2‐aryl migratory insertion. When Bi(I) **5a** and **1a** were mixed in the presence of aldehyde **2a** (Figure 2c), neither insertion product **5g** was detected by NMR or HRMS, nor alcohol **4a’** was obtained after mild acidic quench. Instead, the aldehyde remained unreactive. However, when the aldehyde was replaced by silicon‐based reductant **3a**, the C─Si bond formation product **6c** was observed, together with deuterated product **6a**. We believe that the former originates from a putative intermediate **5h**, followed by a C─Si reductive elimination^[^ ^67^ ^]^ (*vide infra*). Control experiments revealed that **6c** is not formed by the simple combination of **1a** and **3a** with or without CsF (see Supporting Information for details). At this point, we wondered whether compound **6c** could be a competent intermediate in the reaction.^[^ ^68^ ^]^ Yet, when **6c** was mixed with **2a** at 40 °C in the presence of CsF, hydrodefluorination product was exclusively obtained. This suggests that a pathway involving an in situ generated TMS‒Ar is unlikely, and points to a unique reactivity involving the Bi(III)‒Ar in combination with TMS‒DHP (vide infra). Finally, we confirmed that RO‒TMS bond formation can occur from alkoxy bismuth(III) model substrate **5i** in the presence of TMS‒DHP, thus leading to a complete regeneration of Bi(I) (Figure 2d).^[^ ^69^ ^]^ It is also worth pointing out that this represents a novel strategy to access Bi(I) compounds.^[^ ^56^, ^57^ ^]^ Taken together, these results point to **5h** being responsible for an insertion of aldehyde into the Bi─C bond.

At this point, DFT calculations were performed to examine the feasibility of intermediate **5h** in a 1,2‐insertion with an aldehyde (Figure 3). Initially, potential energy surface modeling of the reductive elimination of C─Si bond in **5h** was conducted, in order to study the formation of **6c** (Figure 2c). Consistent with the experimental observation, the C─Si bond formation is found to be viable with **TS1’** (Δ*G* ^‡^ = 25.9 kcal mol^−1^), supporting the formation of intermediate **5h**. We then investigated the insertion of benzaldehyde into the Bi─C and Bi─Si bonds in **5h**. The insertion into the Bi─C bond is predicted to be kinetically more accessible via **TS1^BiC^** (Δ*G* ^‡^ = 15.1 kcal mol^−1^). Albeit thermodynamically favored, insertion into the Bi─Si bond is calculated to be kinetically less accessible (**TS1^BiSi^**, Δ*G* ^‡^ = 40.5 kcal mol^−1^). The opposite selectivity observed in the C═O insertion step compared to lighter Pn─Si bonds, such as P─Si bonds,^[^ ^70^, ^71^ ^]^ is rationalized by the comparatively higher nucleophilicity of the *ipso* carbon of the elongated Bi─Ar bond. In agreement with the kinetic preference of insertion over C─Si formation in **6c**, the kinetic barrier for **TS1^BiC^** is much lower than that for **TS1’**. These results support the proposed aldehyde insertion into the Bi─C bond in **5h**. Additionally, reductive elimination from **INT1^BiC^** to forge the O─Si bond was found to be highly exergonic with a barrier of 11.9 kcal mol^−1^ (**TS2**), affording the product (**4z**) and returning the Bi catalyst to **5a**.^[^ ^72^, ^73^, ^74^, ^75^ ^]^ A summarized mechanism is depicted in Figure 4.

**Figure 3:**
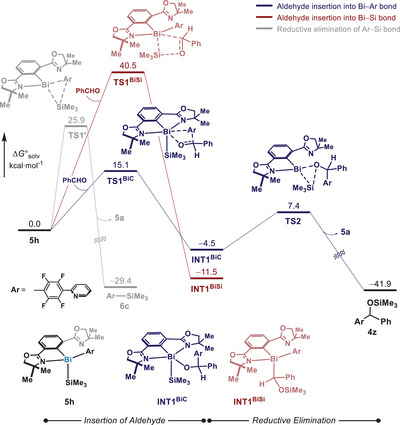
DFT calculations of benzaldehyde insertion by **5h** at the PBE0‐D3BJ/Def2‐TZVP/CPCM(acetonitrile) level of theory. Relative free energies are given in kcal mol^−1^.

**Figure 4:**
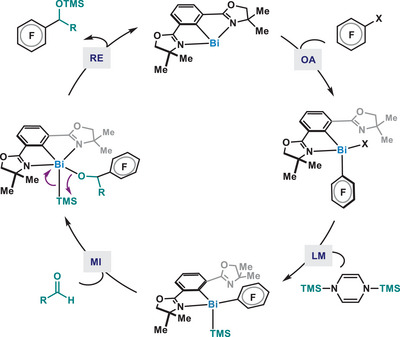
Proposed mechanistic cycle.

In conclusion, we report the catalytic activity of Phebox‐Bi(I) (**5a**) in the reductive coupling reaction between aldehydes and a variety of polyfluoroarenes under mild conditions. Mechanistic investigations support a Bi(I)/(III) redox cycle, where the Bi center undergoes formal oxidative addition, ligand metathesis, migratory insertion and reductive elimination steps, conventionally exploited in transition‐metal catalysis. The facile cycling through four elementary organometallic steps in the Bi(I)/Bi(III) redox manifold serves as a response to the long‐standing challenge in the field of redox catalysis using low‐valent main‐group compounds, potentially enabling a myriad of catalytic redox processes beyond C─C bond formation.

## Conflict of Interests

The authors declare no conflict of interest.

## Supporting information



Supporting Information

Supporting Information

## Data Availability

The data that support the findings of this study are available in the Supporting Information of this article.
