# Supplementary material for: Catalytic 1,2‐Migratory Insertion in a Bismuth Redox Platform: Reductive Arylation of Aldehydes
Source: Angew Chem Int Ed Engl. 2025 Aug 6;64(38):e202510360. doi: 10.1002/anie.202510360 (PMC12435408; doi:10.1002/anie.202510360)
Supplement: Supplementary file 1 — Supporting Information [file ANIE-64-e202510360-s001.pdf]

Supporting Information

Catalytic 1,2-Migratory Insertion in a Bismuth Redox Platform: Reductive Arylation of Aldehydes

Xiangrong Liu, Hye Won Moon, Davide Spinnato, Markus Leutzsch and Josep Cornella*

Max-Planck-Institut für Kohlenforschung, Kaiser-Wilhelm-Platz 1, Mülheim an der Ruhr, 45470, Germany

*E-mail: cornella@kofo.mpg.de

Table of Contents

1. GENERAL INFORMATION 3

2. EXPERIMENTAL DETAILS 4

2.1 Preparation of polyfluoroarenes 4

2.2 Optimization of reaction conditions 5

2.3 General procedures for the reductive arylation reaction 6

2.4 Additional examples tested 8

2.5 Characterization Data 9

3. MECHANISTIC STUDIES 19

3.1 Control experiments 19

3.2 Stochiometric reaction between Bi(I) and polyfluoroarene 20

3.3 Radical trapping experiments 31

3.4 Cyclic voltammetry 33

3.5 Studying the migratory insertion 34

3.6 Reductive elimination of Bi(III) with 3a 40

3.7 Kinetic experiments of the catalytic arylation reaction 41

4. COMPUTATIONAL DETAILS AND DISCUSSION 44

5. REFERENCES 47

6. CRYSTALLOGRAPHIC DATA 48

Single crystal structure analysis of complex 4g’ 48

Single crystal structure analysis of complex 5i 53

7. NMR SPECTRA 58

8. CARTESIAN COORDINATES 116

1. General information

Unless otherwise stated, all experiments were performed under argon using standard Schlenk techniques or in a MBraun argon-filled glove box. Column chromatography was carried out on silica gel (VWR Silica gel 60, 40-63μm). Mass spectra were acquired using the following instruments: EI - Finnigan MAT 8200 (70 eV); ESI-MS - Bruker ESQ 3000. Accurate mass determinations: Bruker APEX III FT-MS (7 T magnet) or Finnigan MAT 95.

NMR data were recorded using a Bruker AVIII HD 300 MHz, Bruker AVIII HD 400 MHz, Bruker AVIII 500 MHz or Bruker AVNeo 600 MHz NMR spectrometer. ^{1}H and ^{13}C chemical shifts are reported relative to the solvent residual peaks as an internal reference. For ^{1}H NMR the following residual proton peaks of the deuterated solvents were used: $CDCl_3$, $\delta_H(CHCl_3)$ 7.260; THF-d_8, $\delta_H((CD_2)_3CHDO)$ 3.580; CD_3CN, δ_H (CHD_2CN) 1.940. For ^{13}C NMR: $CDCl_3$, δ 77.16; THF-d_8, δ 67.57; CD_3CN, δ 1.32. ^{19}F NMR shifts are reported relative to the ^{19}F resonances of $CFCl_3$.^{19}F data at 282 MHz NMR is generally reported with ^{1}H decoupling, and ^{19}F data at 565 MHz NMR is reported without ^{1}H decoupling due to hardware limitations. 1D ^{29}Si NMR spectra were generally acquired with a refocused INEPT sequence and broadband proton decoupling. ^{29}Si NMR shifts are reported relative to tetramethylsilane. Data are reported as follows: chemical shift, multiplicity (br = broad, s = singlet, d = doublet, t = triplet, q = quartet, p = quintet, hept = heptaplet , m = multiplet, dd = doublet of doublets, dt = doublet of triplets, td = triplet of doublets, dp = doublet of quintet, ddd = doublet of doublet of doublets, ddt = doublet of doublet of triplets, dddd = doublet of doublet of doublet of doublets), coupling constant (Hz), and integration.

Unless otherwise noted, THF, MeCN, *n*-pentane, THF-d_8, MeCN-d_3 and other solvents were distilled from the proper drying agents (THF, ether and THF-d_8, Na/benzophenone; *n*-pentane, Na; MeCN and MeCN-d_3, CaH_2) and stored over 4 Å molecular sieves under argon prior to use. 4 Å molecular sieves were activated at 250 °C under high vacuum (1×10^{-2} bar) for 3 days.

Unless otherwise noted, all reagents were obtained from commercial suppliers and used without further purification. PheBox-Bi(I) **5a**, (^{t}BuBis-imine)Bi(I) **5b**, 1,4-bis(trimethylsilyl)-1,4-dihydropyrazine (**3a**), and some polyfluoroarenes (**1**) were prepared according to reported procedures. [1-5]

2. Experimental details

2.1 Preparation of polyfluoroarenes

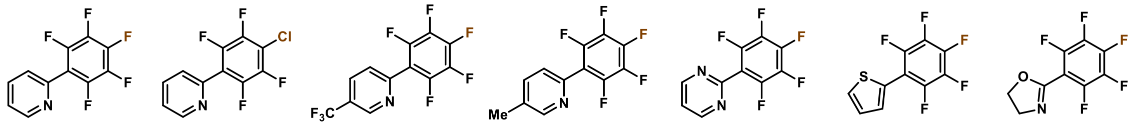

1a 1a´ 1b 1c 1d 1e 1f

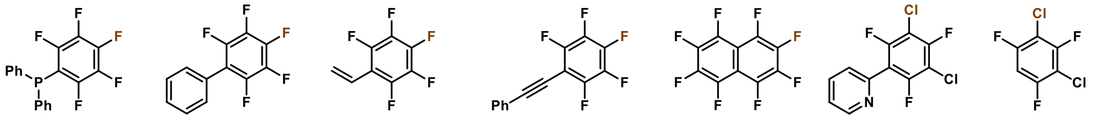

1g 1h 1i 1j 1k 1l 1m

Unless otherwise noted, polyfluoroarenes were obtained from commercial suppliers and used without further purification. In addition, polyfluoroarenes **1a**–**f, 1i**–**1j** and **1l** were prepared according to reported procedures.[4-5]

(1a´) 2-(4-chloro-2,3,5,6-tetrafluorophenyl)pyridine

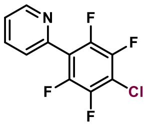

Prepared according to modified literature procedure.[4] A 10 mL Schlenk flask equipped with a magnetic stir bar was charged with the 2-bromopyridine (2.0 mmol), phenanthroline (10 mol %), 3-chloro-1,2,4,5-tetrafluorobenzene (2.0 equiv) and a mixture (1:1) of DMF and xylene (total amount: 1.2 mL). The flask was evacuated and refilled with argon. Under dynamic Ar flow, CuI (10 mol %) and K_3PO_4 (3.0 equiv) were added to the mixture, and the mixture was placed in a preheated oil bath (130 ^oC) for 24 h. The reaction mixture was then cooled to room temperature and diluted with ethyl acetate (20 mL). The resulting solution was washed with brine (3 x 15 mL), dried over anhydrous $MgSO_4$, and concentrated under vacuum. The crude mixture was purified by flash chromatography on silica gel (eluent: hexanes/EtOAc = 10:1), affording **1a´** as a yellow oil (300 mg, 55% yield).

^{1}H NMR (400 MHz, $CDCl_3$) δ 8.84 – 8.71 (m, 1H), 7.83 (td, *J* = 7.8, 1.8 Hz, 1H), 7.48 (dt, *J* = 7.9, 1.2 Hz, 1H), 7.38 (ddd, *J* = 7.6, 4.9, 1.1 Hz, 1H).
^{19}F NMR (282 MHz, $CDCl_3$) δ -140.74 – -140.96 (m), -142.60 – -142.83 (m).
^{13}C NMR (101 MHz, $CDCl_3$) δ 150.3, 147.2, 146.3 – 145.5 (m), 143.6 – 143.0 (m), 136.8, 126.0 (t, *J* = 2.0 Hz), 124.0, 119.0 (t, *J* = 16.3 Hz), 113.0 – 112.2 (m).
HRMS (ESI-TOF): calc'd for $C_{11}H_5NF_4Cl$ $[M+H]^+$: 262.0041, found: 262.0040.

(1l) 2-(3,5-dichloro-2,4,6-trifluorophenyl)pyridine

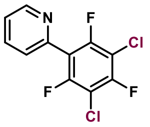

Prepared according to modified literature procedure.[4] A 10 mL Schlenk flask equipped with a magnetic stir bar was charged with the 2-bromopyridine (2.0 mmol), phenanthroline (10 mol %), 2,4-dichloro-1,3,5-trifluorobenzene (2.0 equiv) and a mixture (1:1) of DMF and xylene (total volume: 1.2 mL). The flask was evacuated and refilled with argon. Under dynamic Ar flow, CuI (10 mol %) and K_3PO_4 (3.0 equiv) were added to the mixture, and the mixture was placed in a preheated oil bath (130 ^oC) for 24 h. The reaction mixture was then cooled to room temperature and diluted with ethyl acetate (20 mL). The resulting solution was washed with brine (3 x 15 mL), dried over anhydrous $MgSO_4$, and concentrated under vacuum. The crude mixture was purified by flash chromatography on silica gel (eluent: hexanes/EtOAc = 10:1), affording **1l** as pale yellow solid (410 mg, 70% yield).

^{1}H NMR (400 MHz, $CDCl_3$) δ 8.84 – 8.70 (m, 1H), 7.84 (td, *J* = 7.7, 1.8 Hz, 1H), 7.46 (dt, *J* = 7.9, 1.2 Hz, 1H), 7.38 (ddd, *J* = 7.7, 4.9, 1.2 Hz, 1H).

^{19}F NMR (282 MHz, $CDCl_3$) δ –110.54 (t, *J* = 2.4 Hz), –115.23 (d, *J* = 2.3 Hz).

^{13}C NMR (101 MHz, $CDCl_3$) δ 157.1 – 155.7 (m), 154.3 – 153.3 (m), 150.3, 147.5, 136.9, 126.0, 123.9, 117.1 – 115.5 (m), 108.5 – 107.4 (m).

HRMS (EI): calc'd for $C_{11}H_4NF_3Cl_2$ $[M]^+$: 276.9667, found: 276.9670.

2.2 Optimization of reaction conditions

A culture tube with a Teflon screw-cap equipped with a Teflon-coated stir bar was used. 2-(perfluorophenyl) pyridine **1a** (12.3 mg, 0.05 mmol, 1.0 equiv.) and 4-isopropylbenzaldehyde **2a** (23 µL, 0.15 mmol, 3.0 equiv.) were introduced into the culture tube. Then, in an argon-filled glovebox, catalyst **5** (2.5 µmol, 0.05 equiv.), Si-based reagent **3** (0.05 mmol, 1.0 equiv.) and the solvent (0.25 mL) were added. The culture tube was sealed with a Teflon screw-cap and taken out of the glovebox and the mixture was stirred for 16 h at the appropriate temperature. After completion of the reaction, the solvent was removed under reduced pressure. The crude mixture was diluted with 0.50 mL of $CDCl_3$, 1,1,2-trichloroethene (9.0 µL, 0.10 mmol, 2.0 equiv.) was added as the internal standard, and quantitive ^{1}H NMR was recorded to determine the yield.

Table S1. Reaction optimization under different solvents and temperature[a]

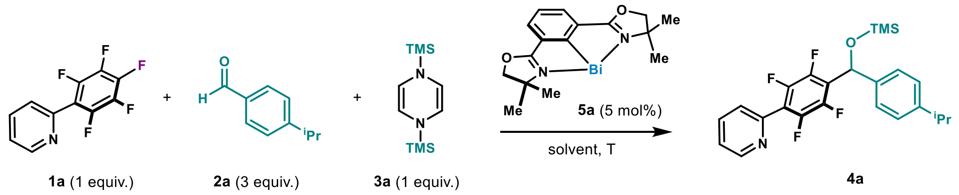

entry	solvent	T/°C	Yield/%
1	MeCN	60	62
2	EtOAc	60	nd
3	pentane	60	nd
4	ether	60	nd
5	toluene	60	nd
6	THF	60	nd
7	MeCN	40	95(93[b])
8	MeCN	25	30
9	DMA	40	48
10	^{t}BuCN	40	12
11	PhCN	40	41
12 [c]	MeCN	40	40
13 [d]	MeCN	40	58
14	DMA	60	80

[a]Reactions performed on 0.05 mmol scale. Yields calculated by quantitative ^{1}H NMR using 1,1,2-trichloroethene as internal standard. [b]Isolated yield on 0.20 mmol scale reaction. [c]0.05 M concentration. [d]2.0 equiv. of aldehyde was added.

Alternative solvents such as DMA (entry 14) could also afford the desired product in 80% yield at 60 °C, suggesting that MeCN acts as a solvent rather than an active participant in the reaction. More specifically, we believe that due to the highly polar nature of Bi(III) intermediates and TSs involved in our catalytic reaction, coordinating and polar solvents such as CH_3CN (ε= 37.5) or DMA (ε= 37.8) perform better if compared to coordinating but poorly polar solvents such as THF (ε= 7.6) or EtOAc (ε= 6.0).

Table S2. Reaction optimization using different catalysts and silanes[a]

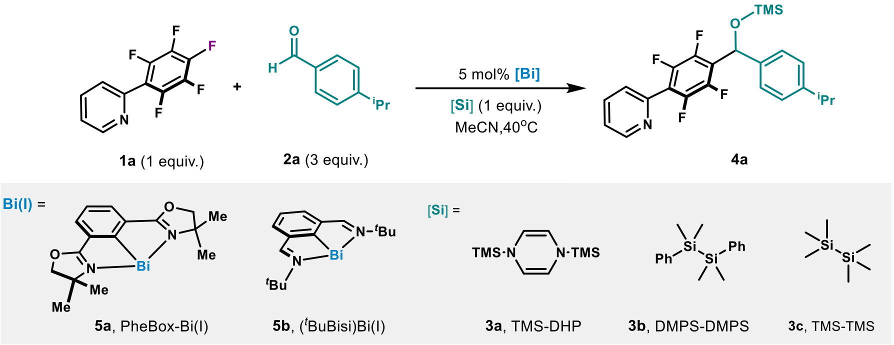

entry	[Bi]	[Si]	Yield/%
1	**5a**	**3a**	95(93[b])
2	**5b**	**3a**	7
3	$BiCl_3$	**3a**	nd
4	none	**3a**	nd
5[c]	none	**3a**	nd
6[d]	none	**3a**	nd
7	**5a**	**3b**	nd
8	**5a**	**3c**	nd

[a]Reactions performed on 0.05 mmol scale. Yields calculated by quantitative ^{1}H NMR yield using 1,1,2-trichloroethene as internal standard. [b]Isolated yield on 0.20 mmol scale reaction. [c]CsF (5 mol %) as catalyst. [d]TBAF (5 mol %) as catalyst.

2.3 General procedures for the reductive arylation reaction

General Procedure A:

Polyfluoroarenes **1** (0.20 mmol, 1.0 equiv.) and aldehydes **2** (0.60 mmol, 3.0 equiv.) were introduced into a culture tube equipped with a Teflon-coated stir bar. (*Note: volatile reactants were introduced inside a glovebox*). Then, inside an argon-filled glovebox, PheBox-Bi(I) catalyst **5a** (4.8 mg, 0.01 mmol, 0.05 equiv.), 1,4-bis(trimethylsilyl)-1,4-dihydropyrazine **3a** (0.05 mmol, 1.0 equiv.) and anhydrous MeCN (1.0 mL, 0.20 M) were added and the culture tube was sealed with a Teflon screw-cap. The culture tube was taken outside the glovebox and the mixture was stirred at 40 °C for 16 h. Upon completion of the reaction, the mixture was concentrated, diluted with EtOAc (approximately 4 mL), washed with brine (approximately 3 mL), and dried over Na_2SO_4. After filtration, the organic layer was concentrated and purified by flash column chromatography on silica gel to afford the desired products.

For unstable products that are prone to decompose on silica gel after the reductive arylation reaction, TMS deprotection and/or subsequent oxidation steps was performed as outlined below to acquire more stable alcohols or ketones suitable for characterization.

General Procedure B with TMS deprotection:

Polyfluoroarenes **1** (0.20 mmol, 1.0 equiv.) and aldehydes **2** (0.60 mmol, 3.0 equiv.) were introduced into a culture tube equipped with a Teflon-coated stir bar. (*Note: volatile reactants were introduced inside a glovebox*). Then, inside an argon-filled glovebox, PheBox-Bi(I) catalyst **5a** (4.8 mg, 0.01 mmol, 0.05 equiv.), 1,4-bis(trimethylsilyl)-1,4-dihydropyrazine **3a** (0.05 mmol, 1.0 equiv.) and anhydrous MeCN (1.0 mL, 0.20 M) were added and the culture tube was sealed with a Teflon screw-cap. The culture tube was taken outside the glovebox and the mixture was stirred at 40 °C for 16 h. Upon completion of the reaction, the mixture was concentrated and redissolved in THF (1.0 mL). TBAF (0.40 mL, 1.0 mol/L in THF, 2.0 equiv.) was added in one portion and the mixture was stirred at 25 °C for 6 h. Then, the mixture was diluted with EtOAc, washed with brine, and dried over Na_2SO_4. After filtration, the organic layer was concentrated and purified by flash column chromatography on silica gel to afford the desired products.

General Procedure C with TMS deprotection and oxidation I:

Polyfluoroarenes **1** (0.20 mmol, 1.0 equiv.) and aldehydes **2** (0.60 mmol, 3.0 equiv.) were introduced into a culture tube equipped with a Teflon-coated stir bar. (*Note: volatile reactants were introduced inside a glovebox*). Then, inside an argon-filled glovebox, PheBox-Bi(I) catalyst **5a** (4.8 mg, 0.01 mmol, 0.05 equiv.), 1,4-bis(trimethylsilyl)-1,4-dihydropyrazine **3a** (0.05 mmol, 1.0 equiv.) and anhydrous MeCN (1.0 mL, 0.20 M) were added and the culture tube was sealed with a Teflon screw-cap. The culture tube was taken outside the glovebox and the mixture was stirred at 40 °C for 16 h. Upon completion of the reaction, the mixture was concentrated under reduced pressure and redissolved in THF (1.0 mL). TBAF (0.40 mL, 1.0 mol/L in THF, 2.0 equiv.) was added in one portion and the mixture was stirred at 25 °C for 6 h. The reaction was quenched with water and extracted with EtOAc. The organic layer was then collected and concentrated. The crude mixture was redissolved in DCM (2.0 mL) and pyridinium chlorofrochromate (65 mg, 0.30 mmol, 1.5 equiv.) was added under an argon atmosphere and the mixture was left stirring at 25 °C for 12 h (overnight). Upon completion, the crude mixture was concentrated and purified by flash column chromatography on silica gel to afford the desired products.

General Procedure D with TMS deprotection and oxidation II:

Polyfluoroarenes **1** (0.20 mmol, 1.0 equiv.) and aldehydes **2** (0.60 mmol, 3.0 equiv.) were introduced into a culture tube equipped with a Teflon-coated stir bar. (*Note: volatile reactants were introduced inside a glovebox*). Then, inside an argon-filled glovebox, PheBox-Bi(I) catalyst **5a** (4.8 mg, 0.01 mmol, 0.05 equiv.), 1,4-bis(trimethylsilyl)-1,4-dihydropyrazine **3a** (0.05 mmol, 1.0 equiv.) and anhydrous MeCN (1.0 mL, 0.20 M) were added and the culture tube was sealed with a Teflon screw-cap. The culture tube was taken outside the glovebox and the mixture was stirred at 40 °C for 16 h. Upon completion of the reaction, the mixture was concentrated under reduced pressure and redissolved in THF (1.0 mL). TBAF (0.40 mL, 1.0 mol/L in THF, 2.0 equiv.) was added in one portion and the mixture was stirred at 25 °C for 6 h. The reaction was quenched with water and extracted with EtOAc. The organic layer was then collected and concentrated. The crude compounds were re-dissolved in acetone (1.0 mL) and a solution of CrO_3 (2.0 M, 40 mg in 0.80 mL) in a mixture of water/sulfuric acid (5/1, *v*/*v*) was added. The mixture was stirred at 25 °C for 12 h (overnight). Upon completion, the reaction mixture was quenched with isopropanol, extracted with EtOAc and washed with water. The organic layer was concentrated and purified by flash column chromatography on silica gel to afford the desired products.

2.4 Additional examples tested

Table S3. Unsuccessful and/or moderately successful polyfluoroarenes[a]

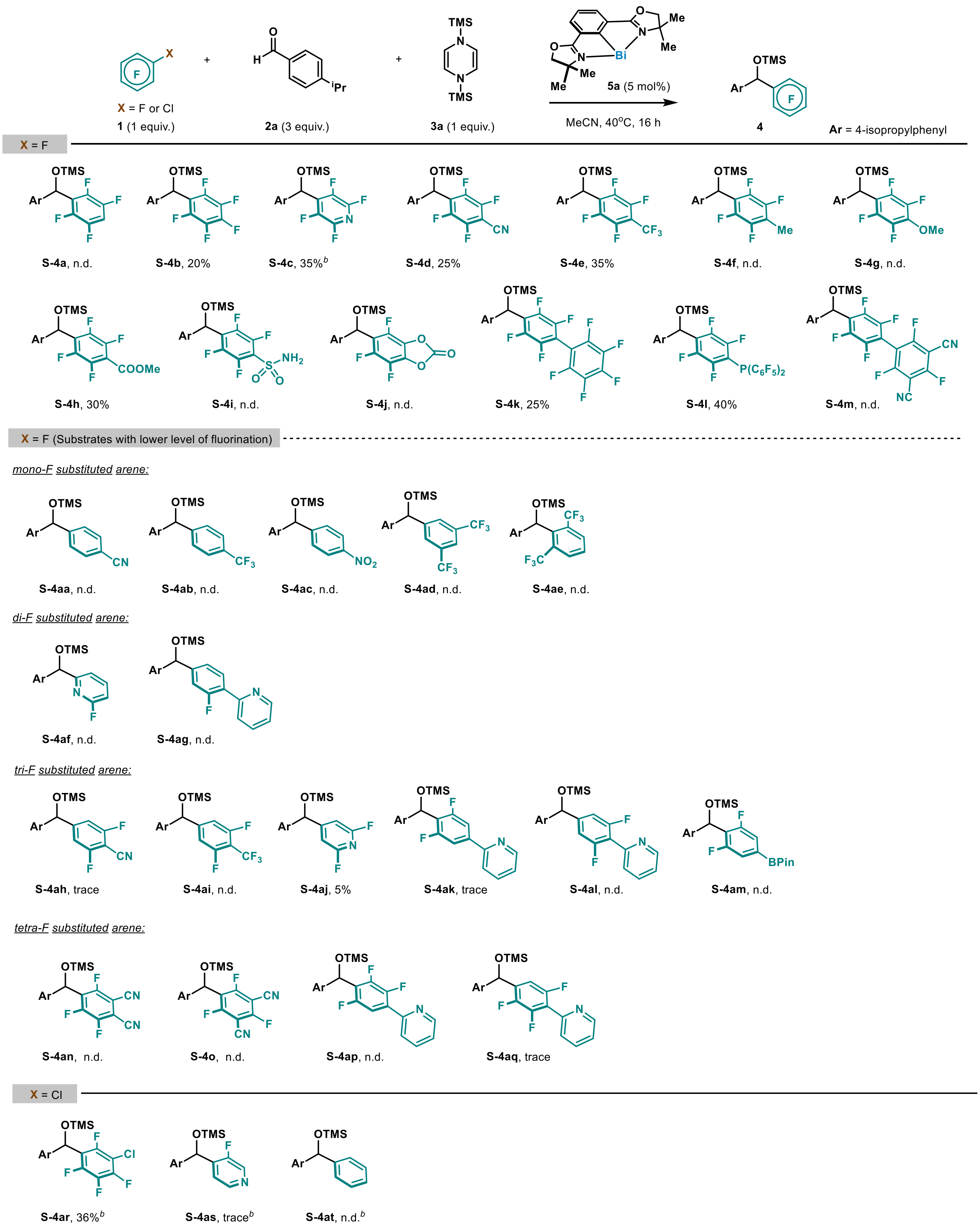

[a]Reactions performed on 0.05 mmol scale. Yields calculated by quantitative ^{1}H NMR using 1,1,2-trichloroethene as internal standard. [b]10mol% of **5a**, 2.00 equiv. of **3a** and 5.00 equiv. of **2a** were used.

Table S4. Unsuccessful and/or moderately successful aldehydes[a]

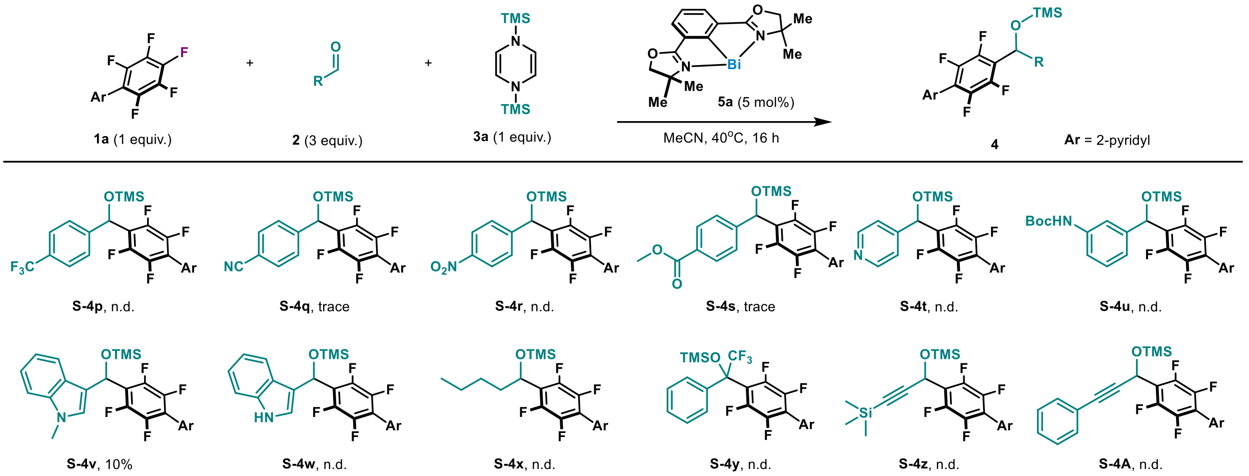

Reactions performed on 0.05 mmol scale. Yields calculated by quantitative ^{1}H NMR using 1,1,2-trichloroethene as internal standard.

2.5 Characterization Data

(4a) 2-(2,3,5,6-tetrafluoro-4-((4-isopropylphenyl)((trimethylsilyl)oxy)methyl)phenyl)pyridine:

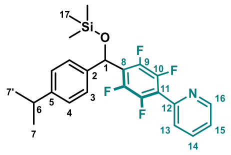

Following **General Procedure A** on 0.20 mmol scale. Purification by flash column chromatography on silica gel (eluent: hexanes/EtOAc = 30:1) afforded 83 mg (93%) of the title compound **4a** as light yellow oil.

^{1}H NMR (600 MHz, $CDCl_3$) δ 8.76 (dt, *J* = 4.9, 1.8 Hz, 1H, 16), 7.81 (td, *J* = 7.8, 1.8 Hz, 1H, 14), 7.47 (dp, *J* = 7.8, 1.0 Hz, 1H, 13), 7.39 – 7.35 (m, 1H, 3), 7.36 (ddd, *J* = 7.7, 4.9, 1.2 Hz, 2H, 15), 7.22 – 7.18 (m, 2H, 4), 6.27 (s, 1H, 1), 2.90 (hept, *J* = 6.9 Hz, 1H, 6), 1.24 (d, *J* = 6.9 Hz, 3H, 7'), 1.24 (d, *J* = 6.9 Hz, 3H, 7), 0.15 (s, 9H, 17).
^{19}F NMR (565 MHz, $CDCl_3$) δ -143.07 (dd, *J* = 22.2, 12.7 Hz, 9), -144.27 (dd, *J* = 22.2, 12.7 Hz, 10).
^{29}Si NMR (119 MHz, $CDCl_3$) δ 20.62.
^{13}C NMR (151 MHz, $CDCl_3$) δ 150.2 (16), 148.4 (5), 147.9 (12), 144.5 (dddd, *J* = 249.1, 14.3, 6.6, 4.0 Hz, 9), 144.3 (ddt, *J* = 249.9, 15.0, 4.5 Hz, 10), 139.1 (2), 136.7 (14), 126.5 (4), 126.0 (t, *J* = 1.9 Hz, 13), 125.6 (3), 123.80 (t, *J* = 14.9 Hz, 8), 123.76 (15), 119.2(t, *J* = 16.1 Hz, 11), 67.3 (1), 33.9 (6), 24.12(7'), 24.11(7), -0.11 (17).
HRMS (ESI-TOF): calc'd for $C_{24}H_{25}F_4N_1O_1Si_1Na$ $[M+Na]^+$: 470.1534, found: 470.1541.

(4b) (4-isopropylphenyl)(2,3,5,6-tetrafluoro-4-(5-(trifluoromethyl)pyridin-2-yl)phenyl)methanol

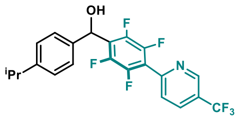

Following **General Procedure B** on 0.20 mmol scale. Purification by flash column chromatography on silica gel (eluent: hexanes/EtOAc = 5:1 ~ 3:1) afforded 72 mg (81%) of the title compound **4b** as yellow oil.

^{1}H NMR (600 MHz, $CDCl_3$) δ 9.04 – 9.00 (m, 1H), 8.09 (ddd, *J* = 8.2, 2.4, 0.7 Hz, 1H), 7.64 (d, *J* = 8.1 Hz, 1H), 7.37 (d, *J* = 8.2 Hz, 2H), 7.28 – 7.21 (m, 2H), 6.29 (d, *J* = 6.8 Hz, 1H), 3.13 – 3.05 (m, 1H), 2.92 (hept, *J* = 6.9 Hz, 1H), 1.25 (d, *J* = 6.9 Hz, 6H).
^{19}F NMR (565 MHz, $CDCl_3$) δ -62.60, -142.81 (dd, *J* = 22.0, 12.6 Hz), -143.54 (dd, *J* = 21.9, 12.6 Hz).
^{13}C NMR (151 MHz, $CDCl_3$) δ 151.3, 149.3, 147.1 (q, *J* = 3.9 Hz), 144.7 (dddd, *J* = 248.5, 14.3, 6.6, 4.1 Hz), 144.4 (ddt, *J* = 251.7, 15.2, 4.4 Hz), 138.1, 134.1 (q, *J* = 3.4 Hz), 127.0, 126.7 (q, *J* = 33.5 Hz), 125.81 (t, *J* = 2.0 Hz), 125.75, 123.6 (t, *J* = 14.8 Hz), 123.3 (q, *J* = 272.6 Hz), 118.1 (t, *J* = 15.6 Hz), 68.2, 34.0, 24.04, 24.03.
HRMS (ESI-TOF): calc'd for $C_{22}H_{17}F_7NO$ $[M+H]^+$: 444.1194, found: 444.1193.

(4c) (4-isopropylphenyl)(2,3,5,6-tetrafluoro-4-(5-methylpyridin-2-yl)phenyl)methanol

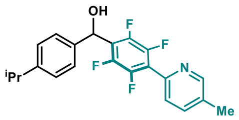

Following **General Procedure B** on 0.20 mmol scale. Purification by flash column chromatography on silica gel (eluent: hexanes/EtOAc = 5:1) afforded 45 mg (58%) of the title compound **4c** as pale yellow oil.

^{1}H NMR (300 MHz, $CDCl_3$) δ 8.54 (d, *J* = 2.2 Hz, 1H), 7.64 – 7.55 (m, 1H), 7.37 – 7.29 (m, 3H), 7.23 – 7.17 (m, 2H), 6.22 (d, *J* = 6.8 Hz, 1H), 3.47 (d, *J* = 7.5 Hz, 1H), 2.87 (hept, *J* = 6.9 Hz, 1H), 2.37 (s, 3H), 1.21 (d, *J* = 6.9 Hz, 6H).
^{19}F NMR (282 MHz, $CDCl_3$) δ -143.58 – -143.76 (m), -143.81 – -144.05 (m).
^{13}C NMR (75 MHz, $CDCl_3$) δ 150.6, 149.0, 146.6 – 145.8 (m), 144.7, 143.2 – 142.4 (m), 138.5, 137.4, 133.9, 126.9, 125.8, 125.7, 125.5, 122.5 (t, *J* = 14.9 Hz), 67.9, 34.0, 24.1, 18.5.
HRMS (ESI-TOF): calc'd for $C_{22}H_{20}F_4NO$ $[M+H]^+$: 390.1479, found: 390.1476.

(4d) (4-isopropylphenyl)(2,3,5,6-tetrafluoro-4-(pyrimidin-2-yl)phenyl)methanol

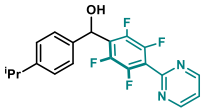

Following **General Procedure B** on 0.20 mmol scale. Purification by flash column chromatography on silica gel (eluent: hexanes/EtOAc = 3:1 ~ 1:1) afforded 70 mg (93%) of the title compound **4d** as pale yellow oil.

^{1}H NMR (300 MHz, $CDCl_3$) δ 9.28 (d, *J* = 4.9 Hz, 2H), 7.85 – 7.68 (m, 3H), 7.66 – 7.56 (m, 2H), 6.65 (d, *J* = 7.7 Hz, 1H), 3.56 (d, *J* = 7.9 Hz, 1H), 3.28 (hept, *J* = 6.8 Hz, 1H), 1.62 (d, *J* = 7.0 Hz, 6H).
^{19}F NMR (282 MHz, $CDCl_3$) δ -143.17 – -143.35 (m), -143.36 – -143.54 (m).
^{13}C NMR (75 MHz, $CDCl_3$) δ 158.3, 157.7, 149.1, 146.9 – 145.8 (m), 143.5 – 142.6 (m), 138.2, 126.9, 125.9, 125.7, 123.6 (t, *J* = 14.3 Hz), 120.6, 118.3, 68.0, 33.9, 24.04, 24.03.
HRMS (EI-TOF): calc'd for $C_{20}H_{16}N_2O_1F_4$ $[M]^+$: 376.1193, found: 376.1197.

(4e) (4-isopropylphenyl)(2,3,5,6-tetrafluoro-4-(thiophen-2-yl)phenyl)methanol

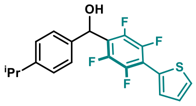

Following **General Procedure B** on 0.20 mmol scale. Purification by flash column chromatography on silica gel (eluent: hexanes/EtOAc = 3:1) afforded 46 mg (60%) of the title compound **4e** as pale yellow oil. (*Note*: 93% yield before TMS deprotection was determined by quantatitive ^{1}H NMR using trichloroethene as internal standard; See the quantatitive ^{1}H NMR spectra in NMR spectra section)

^{1}H NMR (400 MHz, $CDCl_3$) δ 7.63 – 7.58 (m, 1H), 7.55 (dd, *J* = 5.2, 1.1 Hz, 1H), 7.37 (d, *J* = 8.0 Hz, 2H), 7.26 – 7.23 (m, 2H),

7.19 (ddt, J = 4.9, 3.7, 1.0 Hz, 1H), 6.26 (d, J = 7.7 Hz, 1H), 2.91 (hept, J = 7.0 Hz, 1H), 2.69 (d, J = 8.0 Hz, 1H), 1.25 (d, J = 6.9 Hz, 6H).

^{19}F NMR (282 MHz, $CDCl_3$) δ -140.12 – -140.30 (m), -144.00 – -144.19 (m).

^{13}C NMR (101 MHz, $CDCl_3$) δ 149.2, 146.3 – 143.4 (m), 145.2 – 142.3 (m), 138.4, 130.4 (t, J = 5.8 Hz), 128.5 (t, J = 4.1 Hz), 127.4, 127.1, 127.0, 125.8, 120.2 (t, J = 15.2 Hz), 114.0, 68.3 – 68.2 (m), 34.0, 24.1.

HRMS (EI-TOF): calc'd for $C_{20}H_{16}S_1O_1F_4$ $[M]^+$: 380.0853, found: 380.0858.

(4f) (4-(4,5-dihydrooxazol-2-yl)-2,3,5,6-tetrafluorophenyl)(4-isopropylphenyl)methanol

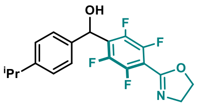

Following **General Procedure B** on 0.20 mmol scale. Purification by flash column chromatography on silica gel (eluent: hexanes/EtOAc = 3:1) afforded 55 mg (75%) of the title compound **4f** as pale yellow solid. (*Note*: 94% yield before TMS deprotection was determined by quantatitive ^{1}H NMR using trichloroethene as internal standard; See the quantatitive ^{1}H NMR spectra in NMR spectra section)

^{1}H NMR (300 MHz, $CDCl_3$) δ 7.32 (d, J = 8.0 Hz, 2H), 7.21 (d, J = 8.3 Hz, 2H), 6.21 (s, 1H), 4.47 (t, J = 9.7 Hz, 2H), 4.11 (t, J = 9.7 Hz, 2H), 3.83 (br, 1H), 2.89 (hept, J = 6.9 Hz, 1H), 1.23 (d, J = 6.9 Hz, 6H).

^{19}F NMR (282 MHz, $CDCl_3$) δ -137.55 – -137.85 (m), -142.38 – -142.60 (m).

^{13}C NMR (75 MHz, $CDCl_3$) δ 156.3, 149.1, 145.0– 143.1 (m), 146.3 – 142.5 (m), 138.0, 126.9, 125.7, 125.2 (t, J = 14.7 Hz), 108.0 (t, J = 15.0 Hz), 68.1, 67.7, 55.1, 33.9, 24.0.

HRMS (EI-TOF): calc'd for $C_{19}H_{17}N_1O_2F_4$ $[M]^+$: 367.1190, found: 367.1191.

(4g) Diphenyl(2,3,5,6-tetrafluoro-4-((4-isopropylphenyl)((trimethylsilyl)oxy)methyl)phenyl)phosphane

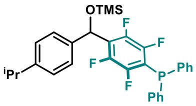

Following **General Procedure A** on 0.20 mmol scale. Purification by preparative LC (150 mm Zorbax Eclipse Plus C18; eluent: acetonitrile/water = 95:5) afforded 100 mg (90%) of the title compound **4g** as pale yellow oil. (*Note*: 95% yield before preparative separation determined by quantatitive ^{1}H NMR using trichloroethene as internal standard; see the quantatitive ^{1}H NMR spectra in NMR spectra section 7)

^{1}H NMR (400 MHz, $CDCl_3$) δ 7.49 – 7.30 (m, 12H), 7.22 – 7.15 (m, 2H), 6.21 (s, 1H), 2.90 (hept, J = 6.9 Hz, 1H), 1.24 (d, J = 6.8 Hz, 6H), 0.11 (s, 9H).

^{19}F NMR (282 MHz, $CDCl_3$) δ -128.06 – -128.99 (m), -141.76 – -142.45 (m).

^{31}P NMR (122 MHz, $CDCl_3$) δ -24.78 (t, J = 37.3 Hz).

^{29}Si NMR (60 MHz, $CDCl_3$) δ 20.54.

^{13}C NMR (75 MHz, $CDCl_3$) δ 148.5, 146.6 – 145.6 (m), 143.1 – 142.4 (m), 139.0, 133.2 (d, J = 21.3 Hz), 130.1, 129.4, 128.8 (d, J = 7.2 Hz), 126.5, 125.7, 125.6, 120.6, 67.6, 33.9, 24.1, -0.1.

HRMS (ESI-TOF): calc'd for $C_{31}H_{31}F_4OPSiNa$ $[M+Na]^+$: 577.1710, found: 577.1714.

(4g') (4-(diphenylphosphoryl)-2,3,5,6-tetrafluorophenyl)(4-isopropylphenyl)methanone

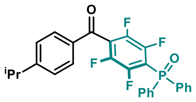

Following **General Procedure C** on 0.20 mmol scale. Purification by flash column chromatography on silica gel (eluent: hexanes/EtOAc = 3:1) afforded 53 mg (53%) of the title compound **4g'** as white solid.

^{1}H NMR (300 MHz, $CDCl_3$) δ 7.88 – 7.73 (m, 6H), 7.69 – 7.47 (m, 6H), 7.42 – 7.32 (m, 2H), 2.99 (hept, *J* = 6.9 Hz, 1H), 1.27 (d, *J* = 6.9 Hz, 6H).
^{19}F NMR (282 MHz, $CDCl_3$) δ -126.98 – -127.37 (m), -138.54 – -138.92 (m).
^{31}P NMR (122 MHz, $CDCl_3$) δ 20.71.
^{13}C NMR (75 MHz, $CDCl_3$) δ 185.1, 157.5, 149.0 – 145.3 (m), 145.3 – 141.4 (m), 133.4, 133.0 (d, *J* = 3.0 Hz), 132.3, 131.4 (d, *J* = 10.8 Hz), 130.8, 130.2, 129.0 (d, *J* = 13.3 Hz), 127.5, 123.5 (t, *J* = 21.0 Hz), 34.6, 23.6.
HRMS (ESI-TOF): calc'd for $C_{28}H_{21}PO_2F_4Na$ $[M+Na]^+$: 519.1108, found: 519.1113.

Suitable single crystals for X-ray diffraction were obtained by vapor diffusion of pentane into a saturated DCM solution of **4g'** at room temprature overnight (*ca.* 16 h).

(4h) (4-isopropylphenyl)(2,3,5,6-tetrafluoro-[1,1'-biphenyl]-4-yl)methanol

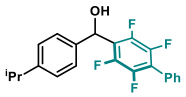

Following **General Procedure B** on 0.20 mmol scale with extended strring time up to 3 days. Purification by flash column chromatography on silica gel (eluent: hexanes/EtOAc = 5:1) afforded 29 mg (38%) of the title compound **4h** as pale yellow oil.

^{1}H NMR (300 MHz, $CDCl_3$) δ 7.60 – 7.50 (m, 5H), 7.47 (d, *J* = 8.1 Hz, 2H), 7.35 – 7.31 (m, 2H), 6.36 (d, *J* = 6.5 Hz, 1H), 3.00 (hept, *J* = 7.3 Hz, 1H), 2.81 (d, *J* = 7.7 Hz, 1H), 1.33 (d, *J* = 6.9 Hz, 6H).
^{19}F NMR (282 MHz, $CDCl_3$) δ -143.65 – -143.85 (m), -143.92 – -144.14 (m).
^{13}C NMR (101 MHz, $CDCl_3$) δ 149.1, 146.2 – 143.0 (m), 145.5 – 142.4 (m), 138.3, 130.1, 129.2, 128.6, 127.3, 126.9, 126.7 (t, *J* = 14.4 Hz), 125.7, 120.9 (t, *J* = 14.6 Hz), 68.2, 33.8, 24.0.
HRMS (EI-TOF): calc'd for $C_{22}H_{18}OF_4$ $[M]^+$: 374.1288, found: 374.1286.

(4i) (4-isopropylphenyl)(2,3,5,6-tetrafluoro-4-vinylphenyl)methanol

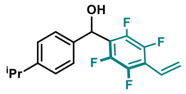

Following **General Procedure B** on 0.20 mmol scale with extended strring time to 3 days. Purification by flash column chromatography on silica gel (eluent: hexanes/EtOAc = 15:1) afforded 35 mg (54%) of the title compound **4i** as yellow oil.

^{1}H NMR (300 MHz, $CDCl_3$) δ 7.33 (d, *J* = 8.1 Hz, 2H), 7.25 – 7.19 (m, 2H), 6.68 (dd, *J* = 18.0, 11.9 Hz, 1H), 6.21 (d, *J* = 5.0 Hz, 1H), 6.11 (d, *J* = 18.0 Hz, 1H), 5.71 (d, *J* = 11.9 Hz, 1H), 2.90 (hept, *J* = 6.9 Hz, 1H), 2.64 (d, *J* = 6.5 Hz, 1H), 1.24 (d, *J* = 6.9 Hz, 6H).
^{19}F NMR (282 MHz, $CDCl_3$) δ -143.77 (dd, *J* = 20.8, 12.3 Hz), -145.03 (dd, *J* = 21.1, 12.4 Hz).
^{13}C NMR (101 MHz, $CDCl_3$) δ 149.2, 146.3 – 143.4 (m), 145.8 – 143.0 (m), 138.4, 127.0, 125.7, 124.0 (t, *J* = 7.8 Hz), 122.4, 120.5 (t, *J* = 15.0 Hz), 116.4 (t, *J* = 13.5 Hz), 68.2 (t, *J* = 2.1 Hz), 34.0, 24.1.
HRMS (EI-TOF): calc'd for $C_{18}H_{16}OF_4$ $[M]^+$: 324.1132, found: 324.1137.

(4j) (4-isopropylphenyl)(2,3,5,6-tetrafluoro-4-(phenylethynyl)phenyl)methanol

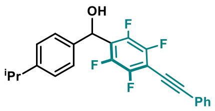

Following **General Procedure B** on 0.20 mmol scale with extended strring time to 3 days. Purification by flash column chromatography on silica gel (eluent: hexanes/EtOAc = 10:1) afforded 38 mg (47%) of the title compound **4j** as yellow oil.

^{1}H NMR (300 MHz, $CDCl_3$) δ 7.55 – 7.43 (m, 2H), 7.35 – 7.20 (m, 5H), 7.15 (d, *J* = 8.1 Hz, 2H), 6.15 (d, *J* = 7.4 Hz, 1H), 2.82 (hept, *J* = 7.0 Hz, 1H), 2.69 (d, *J* = 7.8 Hz, 1H), 1.15 (d, *J* = 7.0 Hz, 6H).
^{19}F NMR (282 MHz, $CDCl_3$) δ -136.43 – -136.71 (m), -143.75 – -143.99 (m).
^{13}C NMR (75 MHz, $CDCl_3$) δ 149.3, 146.9 (ddt, *J* = 253.4, 15.5, 3.7 Hz), 146.2 – 142.4 (m), 138.1, 132.1, 129.7, 128.6, 127.0, 125.7, 122.4 (t, *J* = 14.9 Hz), 121.8, 104.2, 102.0 (t, *J* = 3.8 Hz), 74.4 (t, *J* = 4.3 Hz), 68.3 (t, *J* = 2.1 Hz), 34.0, 24.0.
HRMS (EI-TOF): calc'd for $C_{24}H_{18}OF_4$ $[M]^+$: 398.1288, found: 398.1295.

(4k) (4-isopropylphenyl)(perfluoronaphthalen-2-yl)methanol

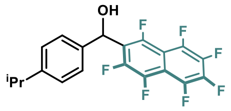

Following **General Procedure B** on 0.20 mmol scale under condition of 10 mol% of **5a**, 2.0 equiv. of **3a** and 5.0 equiv. of **2a** instead. Purification by flash column chromatography on silica gel (eluent: hexanes/EtOAc = 12:1) afforded 44 mg (55%) of the title compound **4k** as yellow oil.

^{1}H NMR (300 MHz, $CDCl_3$) δ 7.35 (d, *J* = 8.0 Hz, 2H), 7.24 (d, *J* = 8.1 Hz, 2H), 6.40 (d, *J* = 7.7 Hz, 1H), 2.91 (hept, *J* = 7.0 Hz, 1H), 2.73 (d, *J* = 8.1 Hz, 1H), 1.24 (d, *J* = 6.9 Hz, 6H).
^{19}F NMR (282 MHz, $CDCl_3$) δ -121.51 – -122.13 (m), -137.90 – -138.19 (m), -143.47 – -144.16 (m), -145.72 – -146.29 (m), -147.99 – -148.59 (m), -152.84 – -153.34 (m), -155.04 – -155.64 (m).
^{13}C NMR (101 MHz, $CDCl_3$) δ 151.4 – 147.2 (m), 149.3, 148.9 – 144.5 (m), 143.4 – 139.9 (m), 141.4 – 138.3 (m), 138.1, 140.6 – 137.3 (m), 127.0, 125.7, 121.1 – 120.6 (m), 111.5 – 107.8 (m), 68.5 – 67.8 (m), 34.0, 24.0.
HRMS (ESI-TOF): calc'd for $C_{20}H_{13}OF_7Na$ $[M+Na]^+$: 425.0747, found: 425.0746.

(4l) 2-(3-chloro-2,4,6-trifluoro-5-((4-isopropylphenyl)((trimethylsilyl)oxy)methyl)phenyl)pyridine

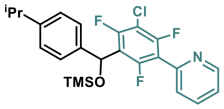

Following **General Procedure A** on 0.20 mmol scale under condition of 10 mol% of **5a**, 2.0 equiv. of **3a** and 5.0 equiv. of **2a** instead. Purification by preparative LC (150 mm Zorbax Eclipse Plus C18; eluent: methanol/water = 90:10) afforded 58 mg (62%) of the title compound **4l** as pale yellow oil.

^{1}H NMR (400 MHz, $CDCl_3$) δ 8.75 (ddd, *J* = 4.9, 1.9, 1.0 Hz, 1H), 7.79 (td, *J* = 7.7, 1.9 Hz, 1H), 7.43 (dt, *J* = 7.8, 1.2 Hz, 1H), 7.37 – 7.33 (m, 2H), 7.19 – 7.14 (m, 2H), 6.24 (s, 1H), 2.88 (hept, *J* = 6.9 Hz, 1H), 1.23 (d, *J* = 7.0 Hz, 6H), 0.12 (s, 9H).
^{19}F NMR (282 MHz, $CDCl_3$) δ -111.99 (t, *J* = 4.5 Hz), -114.68 – -114.95 (m), -117.27 (t, *J* = 5.3 Hz).
^{29}Si NMR (60 MHz, $CDCl_3$) δ 20.02.
^{13}C NMR (101 MHz, $CDCl_3$) δ 157.6 – 154.7 (m), 156.2 (dddd, *J* = 252.4, 76.6, 10.4, 5.5 Hz), 150.1, 148.4, 148.1, 139.5, 136.6, 126.9, 126.4, 126.2, 125.7, 125.5, 123.5, 1191 – 118.3 (m), 116.0 – 115.3 (m), 67.2, 33.9, 24.13, 24.10, -0.1.
HRMS (EI-TOF): calc'd for $C_{24}H_{25}ClF_3NOSiNa$ $[M+Na]^+$: 486.1238, found: 486.1239.

(4m) (3-chloro-2,4,6-trifluorophenyl)(4-isopropylphenyl)methanol

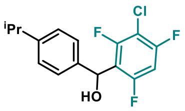

Following **General Procedure B** on 0.20 mmol scale under condition of 10 mol% of **5a**, 2.0 equiv. of **3a** and 5.0 equiv. of **2a** instead. Purification by flash column chromatography on silica gel (eluent: hexanes/EtOAc = 10:1) afforded 34 mg (54%) of the title compound **4m** as pale yellow oil.

^{1}H NMR (300 MHz, $CDCl_3$) δ 7.30 (d, *J* = 8.0 Hz, 2H), 7.22 (d, *J* = 8.3 Hz, 2H), 6.88 – 6.72 (m, 1H), 6.18 (d, *J* = 7.3 Hz, 1H), 2.90 (hept, *J* = 6.9 Hz, 1H), 2.62 (d, *J* = 8.1 Hz, 1H), 1.24 (d, *J* = 6.9 Hz, 6H).
^{19}F NMR (282 MHz, $CDCl_3$) δ -111.00 (t, *J* = 4.4 Hz, 1F), -113.04 – -113.24 (m, 2F).
^{13}C NMR (101 MHz, $CDCl_3$) δ 158.7 (ddd, *J* = 249.9, 13.7, 10.4 Hz), 159.7 – 156.7 (m), 157.1 (ddd, *J* = 251.4, 11.0, 5.3 Hz), 149.0, 138.7, 126.9, 125.7, 117.2 (td, *J* = 17.3, 4.5 Hz), 107.2 – 106.4 (m), 102.1 – 100.9 (m), 67.8, 33.9, 24.1.
HRMS (EI-TOF): calc'd for $C_{16}H_{14}OF_3Cl$ $[M]^+$: 314.0680, found: 314.0680.

(4n) 2-(2,3,5,6-tetrafluoro-4-((4-methoxyphenyl)((trimethylsilyl)oxy)methyl)phenyl)pyridine

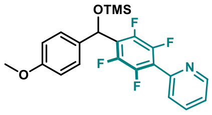

Following **General Procedure A** on 0.20 mmol scale. Purification by flash column chromatography on silica gel (eluent: hexanes/EtOAc = 10:1) afforded 70 mg (80%) of the title compound **4n** as yellow oil.

^{1}H NMR (300 MHz, $CDCl_3$) δ 8.81 – 8.72 (m, 1H), 7.82 (td, *J* = 7.8, 1.8 Hz, 1H), 7.50 – 7.44 (m, 1H), 7.42 – 7.29 (m, 3H), 6.97 – 6.83 (m, 2H), 6.25 (s, 1H), 3.80 (s, 3H), 0.14 (s, 9H).
^{19}F NMR (282 MHz, $CDCl_3$) δ -143.28 (dd, *J* = 22.3, 12.9 Hz), -144.20 (dd, *J* = 22.2, 12.8 Hz).
^{29}Si NMR (60 MHz, $CDCl_3$) δ 20.72.
^{13}C NMR (75 MHz, $CDCl_3$) δ 159.1, 150.2, 147.9, 146.7 – 142.9 (m), 146.1 – 142.5 (m), 136.8, 134.0, 130.1, 126.8 (t, *J* = 1.3 Hz), 126.0 (t, *J* = 1.9 Hz), 123.9 (t, *J* = 15.0 Hz), 123.8, 113.8, 67.1, 55.4, -0.1.
HRMS (ESI-TOF): calc'd for $C_{22}H_{22}N_1O_2Si_1F_4$ $[M+H]^+$: 436.1350, found: 436.1351.

(4o) 2-(2,3,5,6-tetrafluoro-4-((3-methoxyphenyl)((trimethylsilyl)oxy)methyl)phenyl)pyridine

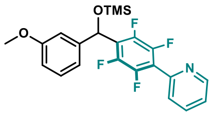

Following **General Procedure A** on 0.20 mmol scale. Purification by flash column chromatography on silica gel (eluent: hexanes/EtOAc = 10:1) afforded 56 mg (64%) of the title compound **4o** as yellow oil.

^{1}H NMR (300 MHz, $CDCl_3$) δ 8.76 (ddd, *J* = 4.9, 1.9, 1.0 Hz, 1H), 7.82 (td, *J* = 7.8, 1.8 Hz, 1H), 7.48 (dt, *J* = 7.8, 1.2 Hz, 1H), 7.36 (ddd, *J* = 7.7, 4.9, 1.2 Hz, 1H), 7.29 – 7.22 (m, 1H), 7.08 (s, 1H), 7.02 – 6.95 (m, 1H), 6.81 (dd, *J* = 8.0, 2.4 Hz, 1H), 6.26 (s, 1H), 3.82 (s, 3H), 0.16 (s, 9H).
^{19}F NMR (282 MHz, $CDCl_3$) δ -142.99 (dd, *J* = 22.1, 12.7 Hz), -144.12 (dd, *J* = 22.2, 12.8 Hz).
^{29}Si NMR (60 MHz, $CDCl_3$) δ 21.05.
^{13}C NMR (75 MHz, $CDCl_3$) δ 159.8, 150.2, 147.9, 146.7 – 145.7 (m), 143.5, 143.2 – 142.3 (m), 136.7, 129.5, 126.0 (t, *J* = 2.1 Hz), 123.8, 123.4 (t, *J* = 15.2 Hz), 119.3, 117.9, 112.9, 111.5 (t, *J* = 1.7 Hz), 67.1 (t, *J* = 2.0 Hz), 55.4, -0.1.
HRMS (ESI-TOF): calc'd for $C_{22}H_{22}N_1O_2F_4Si_1$ $[M+H]^+$: 436.1351, found: 436.1350.

(4p) 2-(2,3,5,6-tetrafluoro-4-((2-methoxyphenyl)((trimethylsilyl)oxy)methyl)phenyl)pyridine

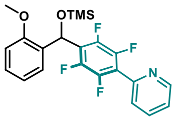

Following **General Procedure A** on 0.20 mmol scale. Purification by flash column chromatography on silica gel (eluent: hexanes/ EtOAc = 15:1) afforded 54 mg (62%) of the title compound **4p** as yellow oil.

^{1}H NMR (300 MHz, $CDCl_3$) δ 8.75 (ddd, *J* = 4.9, 1.8, 0.9 Hz, 1H), 7.89 – 7.74 (m, 2H), 7.48 (d, *J* = 7.8 Hz, 1H), 7.39 – 7.22 (m, 2H), 7.04 (t, *J* = 7.5 Hz, 1H), 6.79 (d, *J* = 7.8 Hz, 1H), 6.43 (s, 1H), 3.74 (s, 3H), 0.14 (s, 9H).
^{19}F NMR (282 MHz, $CDCl_3$) δ -143.14 (dd, *J* = 22.6, 13.0 Hz), -145.44 (dd, *J* = 21.4, 11.8 Hz).
^{29}Si NMR (60 MHz, $CDCl_3$) δ 20.52.
^{13}C NMR (75 MHz, $CDCl_3$) δ 155.6, 150.1, 148.2 – 148.0 (m), 147.1 – 143.1 (m), 146.2 – 142.1 (m), 136.7, 129.6, 128.7, 127.3 (t, *J* = 2.7 Hz), 126.1 (t, *J* = 2.2 Hz), 123.7, 123.1 (t, *J* = 12.8 Hz), 120.3, 118.7 (t, *J* = 16.5 Hz), 109.8, 62.7 (t, *J* = 2.3 Hz), 55.3, -0.1.
HRMS (ESI-TOF): calc'd for $C_{22}H_{22}N_1O_2Si_1F_4$ $[M+H]^+$: 436.1352, found: 436.1350.

(4q) 2-(2,3,5,6-tetrafluoro-4-((4-fluorophenyl)((trimethylsilyl)oxy)methyl)phenyl)pyridine

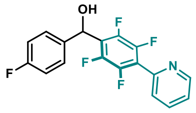

Following **General Procedure B** on 0.20 mmol scale. Purification by flash column chromatography on silica gel (eluent: hexanes/EtOAc = 20:1) afforded 52 mg (74%) of the title compound **4q** as colorless oil.

^{1}H NMR (300 MHz, $CDCl_3$) δ 8.75 (ddd, *J* = 4.9, 1.8, 0.9 Hz, 1H), 7.85 (td, *J* = 7.8, 1.8 Hz, 1H), 7.53 – 7.34 (m, 4H), 7.12 – 6.99 (m, 2H), 6.27 (s, 1H), 3.83 (br, 1H).
^{19}F NMR (282 MHz, $CDCl_3$) δ -114.12 (1F), -143.29 – -143.68 (m, 4F).
^{13}C NMR (75 MHz, $CDCl_3$) δ 162.5 (d, *J* = 246.1 Hz), 150.1, 147.5, 146.6 – 142.6 (m), 146.1 – 142.4 (m), 137.1, 136.9 (d, *J* = 3.4 Hz), 130.1, 127.5 (d, *J* = 9.2 Hz), 126.2, 124.1, 122.5, 115.7 (d, *J* = 21.6 Hz), 67.1.
HRMS (EI-TOF): calc'd for $C_{18}H_{10}NOF_5$ $[M]^+$: 351.0677, found: 351.0681.

(4r) (4-chlorophenyl)(2,3,5,6-tetrafluoro-4-(pyridin-2-yl)phenyl)methanol

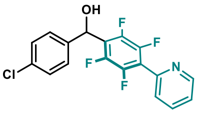

Following **General Procedure B** on 0.20 mmol scale with extended strring time to 3 days. Purification by flash column chromatography on silica gel (eluent: hexanes/EtOAc = 15:1) afforded 40 mg (54%) of the title compound **4r** as beige oil.

^{1}H NMR (600 MHz, $CDCl_3$) δ 8.76 (ddd, *J* = 4.9, 1.8, 1.0 Hz, 1H), 7.85 (td, *J* = 7.8, 1.8 Hz, 1H), 7.49 (dp, *J* = 7.9, 1.2 Hz, 1H), 7.43 – 7.37 (m, 3H), 7.37 – 7.32 (m, 2H), 6.27 (d, *J* = 7.3 Hz, 1H), 3.73 – 3.68 (m, 1H).
^{19}F NMR (565 MHz, $CDCl_3$) δ -143.29 – -143.39 (m), -143.39 – -143.49 (m).
^{13}C NMR (151 MHz, $CDCl_3$) δ 150.1, 147.5, 145.6 – 143.5 (m), 145.3 – 143.4 (m), 139.6, 137.1, 134.0, 128.9, 127.1, 126.2 (d, *J* = 1.7 Hz), 124.1, 122.3 (t, *J* = 14.1 Hz), 119.5 (t, *J* = 14.8 Hz), 67.0 (t, *J* = 2.1 Hz).
HRMS (EI-TOF): calc'd for $C_{18}H_{10}NOF_4Cl$ $[M]^+$: 367.0382, found: 367.0386.

(4s) 2-(2,3,5,6-tetrafluoro-4-(naphthalen-2-yl((trimethylsilyl)oxy)methyl)phenyl)pyridine

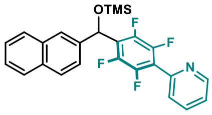

Following **General Procedure A** on 0.20 mmol scale. Purification by flash column chromatography on silica gel (eluent: hexanes/EtOAc = 20:1) afforded 67 mg (73%) of the title compound **4s** as colorless oil.

^{1}**H NMR** (300 MHz, $CDCl_3$) δ 8.76 (ddd, *J* = 4.9, 1.8, 0.9 Hz, 1H), 7.99 – 7.90 (m, 1H), 7.89 – 7.78 (m, 4H), 7.57 – 7.45 (m, 4H), 7.35 (ddd, *J* = 7.7, 4.9, 1.2 Hz, 1H), 6.47 (s, 1H), 0.20 (s, 9H).

^{19}**F NMR** (282 MHz, $CDCl_3$) δ -142.92 (dd, *J* = 22.3, 12.9 Hz), -144.03 (dd, *J* = 22.2, 12.9 Hz).

^{29}**Si NMR** (60 MHz, $CDCl_3$) δ 21.18.

^{13}**C NMR** (75 MHz, $CDCl_3$) δ 150.21, 150.17, 147.8, 146.6 – 142.9 (m), 146.1 – 142.4 (m), 139.2, 136.8, 136.7, 133.3, 133.0, 128.2, 127.8, 126.3, 126.1, 126.0, 124.3, 123.8 (t, *J* = 6.0 Hz), 123.5 (t, *J* = 13.9 Hz), 119.4 (t, *J* = 16.3 Hz), 67.5, -0.1.

HRMS (ESI-TOF): calc'd for $C_{25}H_{22}O_1N_1F_4Si_1$ $[M+H]^+$: 456.1401, found:456.1402.

(4t) (4-(dimethylamino)phenyl)(2,3,5,6-tetrafluoro-4-(pyridin-2-yl)phenyl)methanol

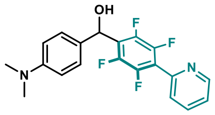

Following **General Procedure B** on 0.20 mmol scale. Purification by flash column chromatography on silica gel (eluent: hexanes/EtOAc = 15:1) afforded 51 mg (68%) of the title compound **4t** as pale yellow oil.

^{1}**H NMR** (400 MHz, $CDCl_3$) δ 8.80 – 8.73 (m, 1H), 7.83 (td, *J* = 7.7, 1.8 Hz, 1H), 7.51 – 7.44 (m, 1H), 7.40 – 7.27 (m, 3H), 6.71 (d, *J* = 8.8 Hz, 2H), 6.21 (s, 1H), 3.20 – 3.02 (br, 1H), 2.95 (s, 6H).

^{19}**F NMR** (282 MHz, $CDCl_3$) δ -143.65 – -143.85(m), -144.00 – -144.20(m).

^{13}**C NMR** (101 MHz, $CDCl_3$) δ 150.6, 150.2, 147.8, 145.8 – 143.2 (m), 145.6 – 143.0 (m), 136.8, 128.6, 126.9, 126.1, 123.8, 123.0 (t, *J* = 14.6 Hz), 119.0 (t, *J* = 16.1 Hz), 112.6, 68.3, 40.6.

HRMS (EI-TOF): calc'd for $C_{18}H_{10}NOF_4Cl$ $[M]^+$: 367.0382, found: 367.0386.

(4u) 2-(2,3,5,6-tetrafluoro-4-(phenanthren-9-yl((trimethylsilyl)oxy)methyl)phenyl)pyridine

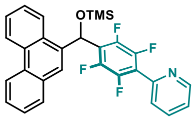

Following **General Procedure A** on 0.20 mmol scale. Purification by flash column chromatography on silica gel (eluent: hexanes/EtOAc = 20:1) afforded 65 mg (64%) of the title compound **4u** as yellow oil.

^{1}**H NMR** (300 MHz, $CDCl_3$) δ 8.81 – 8.64 (m, 3H), 8.42 (s, 1H), 8.09 – 7.91 (m, 2H), 7.81 – 7.59 (m, 5H), 7.43 (d, *J* = 7.9 Hz, 1H), 7.36 – 7.28 (m, 1H), 6.92 (s, 1H), 0.26 (s, 9H).

^{19}**F NMR** (282 MHz, $CDCl_3$) δ -142.09 (dd, *J* = 22.0, 12.6 Hz), -143.93 (dd, *J* = 22.0, 12.6 Hz).

^{29}**Si NMR** (60 MHz, $CDCl_3$) δ 21.52.

^{13}**C NMR** (75 MHz, $CDCl_3$) δ 150.1, 147.7, 147.2 – 143.1 (m), 146.3 – 142.5 (m), 136.7, 134.1, 131.5, 130.7, 130.3, 130.1, 129.3, 129.2, 127.1, 126.98, 126.96, 126.3, 126.1 (t, *J* = 2.7 Hz), 126.0 (d, *J* = 1.9 Hz), 123.8, 123.9, 122.62, 122.58, 119.6 (t, *J* = 16.6 Hz), 64.8, 0.0.

HRMS (ESI-TOF): calc'd for $C_{29}H_{24}N_1O_1Si_1F_4$ $[M+H]^+$: 506.1558, found: 506.1563.

(4v) benzo[b]thiophen-3-yl(2,3,5,6-tetrafluoro-4-(pyridin-2-yl)phenyl)methanol

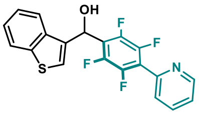

Following **General Procedure B** on 0.20 mmol scale. Purification by flash column chromatography on silica gel (eluent: hexanes/EtOAc = 12:1) afforded 50 mg (64%) of the title compound **4v** as beige oil.

^{1}**H NMR** (400 MHz, $CDCl_3$) δ 8.74 (dd, *J* = 4.9, 1.7 Hz, 1H), 7.92 – 7.79 (m, 3H), 7.52 – 7.45 (m, 2H), 7.44 – 7.33 (m, 3H), 6.59

(s, 1H), 4.05 (br, 1H).
^{19}F NMR (282 MHz, $CDCl_3$) δ -142.85 – -143.15(m), -143.18 – -143.48 (m).
^{13}C NMR (101 MHz, $CDCl_3$) δ 150.0, 147.4, 146.3 – 143.5 (m), 145.8 – 142.9 (m), 140.9, 137.1, 137.0, 135.2, 126.2, 124.8, 124.6, 124.3, 124.1, 123.1, 121.9, 121.4 (t, *J* = 14.5 Hz), 119.5 (t, *J* = 16.0 Hz), 63.6.
HRMS (EI-TOF): calc'd for $C_{20}H_{11}NOF_4S$ $[M]^+$: 389.0492, found: 389.0498.

(4w) (*E*)-3-phenyl-1-(2,3,5,6-tetrafluoro-4-(pyridin-2-yl)phenyl)prop-2-en-1-ol

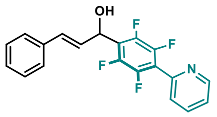

Following **General Procedure B** on 0.20 mmol scale. Purification by flash column chromatography on silica gel (eluent: hexanes/EtOAc = 10:1) afforded 38 mg (53%) of the title compound **4w** as yellow oil. (*Note*: 75% yield before TMS deprotection and oxidation was determined by quantatitive ^{1}H NMR using trichloroethene as internal standard; See the quantative ^{1}H NMR spectra in NMR spectra section)

^{1}H NMR (300 MHz, $CDCl_3$) δ 8.71 (ddd, *J* = 4.9, 1.9, 0.9 Hz, 1H), 7.77 (td, *J* = 7.8, 1.8 Hz, 1H), 7.43 (d, *J* = 7.9 Hz, 1H), 7.36 – 7.27 (m, 4H), 7.24 – 7.16 (m, 1H), 6.65 (d, *J* = 15.9 Hz, 1H), 6.50 (dd, *J* = 15.9, 6.5 Hz, 1H), 5.74 (d, *J* = 6.5 Hz, 1H), 3.07 (br, 1H).
^{19}F NMR (282 MHz, $CDCl_3$) δ -143.60 – -143.79 (m), -143.80 – -144.00 (m).
^{13}C NMR (101 MHz, $CDCl_3$) δ 150.0, 147.5, 146.0 – 143.2 (m), 145.7 – 142.8 (m), 136.9, 135.9, 132.5, 128.7, 128.3, 127.7, 126.8, 126.0, 123.9, 121.5 (t, *J* = 14.7 Hz), 119.2, 67.0 (t, *J* = 2.7 Hz).
HRMS (ESI-TOF): calc'd for $C_{20}H_{14}NOF_4$ $[M+H]^+$: 360.1006, found: 360.1003.

(4x) 2,2-dimethyl-1-(2,3,5,6-tetrafluoro-4-(pyridin-2-yl)phenyl)propan-1-one

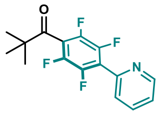

Following **General Procedure D** on 0.20 mmol scale with extended stirring time to 3 days. Purification by flash column chromatography on silica gel (eluent: hexanes/EtOAc = 25:1) afforded 20 mg (32%) of the title compound **4x** as pale yellow oil.

^{1}H NMR (400 MHz, $CDCl_3$) δ 8.78 (ddd, *J* = 4.9, 1.9, 1.0 Hz, 1H), 7.86 (td, *J* = 7.8, 1.8 Hz, 1H), 7.51 (dp, *J* = 7.9, 1.2 Hz, 1H), 7.40 (ddd, *J* = 7.7, 4.9, 1.2 Hz, 1H), 1.28 (t, *J* = 1.1 Hz, 9H).
^{19}F NMR (282 MHz, $CDCl_3$) δ -139.59 – -139.93 (m), -142.08 – -142.32 (m).
^{13}C NMR (101 MHz, $CDCl_3$) δ 203.2, 150.3, 147.3, 144.2 (ddt, *J* = 252.6, 15.2, 4.2 Hz), 142.1 (dddd, *J* = 247.3, 15.9, 7.7, 4.5 Hz), 136.9, 126.1 (t, *J* = 1.8 Hz), 124.1, 120.8 (t, *J* = 16.3 Hz), 120.5 (t, *J* = 24.2 Hz), 45.8, 26.0.
HRMS (EI-TOF): calc'd for $C_{16}H_{13}NOF_4$ $[M]^+$: 311.0928, found: 311.0933.

(4y) (1-methylcyclohexyl)(2,3,5,6-tetrafluoro-4-(pyridin-2-yl)phenyl)methanone

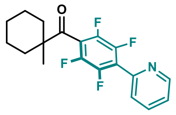

Following **General Procedure D** on 0.20 mmol scale with extended stirring time to 3 days. Purification by flash column chromatography on silica gel (eluent: hexanes/EtOAc = 25:1) afforded 24 mg (35%) of the title compound **4y** as pale yellow oil.

^{1}H NMR (600 MHz, $CDCl_3$) δ 8.79 (d, *J* = 4.9 Hz, 1H), 7.86 (td, *J* = 7.8, 1.8 Hz, 1H), 7.52 (d, *J* = 7.8 Hz, 1H), 7.43 – 7.38 (m, 1H), 1.89 – 1.80 (m, 2H), 1.55 (qt, *J* = 16.8, 4.1 Hz, 8H), 1.28 (s, 3H).

^{19}F NMR (565 MHz, $CDCl_3$) δ -138.97 – -139.29 (m), -142.20 – -142.47 (m).

^{13}C NMR (151 MHz, $CDCl_3$) δ 203.7, 150.3, 147.4, 144.2 (ddt, *J* = 252.5, 15.3, 4.1 Hz), 142.2 (dddd, *J* = 247.5, 15.9, 7.9, 4.3 Hz), 137.0, 126.1, 124.1, 121.0 – 120.6 (m), 120.8 – 120.5 (m), 49.1, 33.7, 25.7, 22.0, 21.4.

HRMS (EI-TOF): calc'd for $C_{19}H_{17}NOF_4$ $[M]^+$: 351.1241, found: 351.1243.

3. Mechanistic studies

3.1 Control experiments

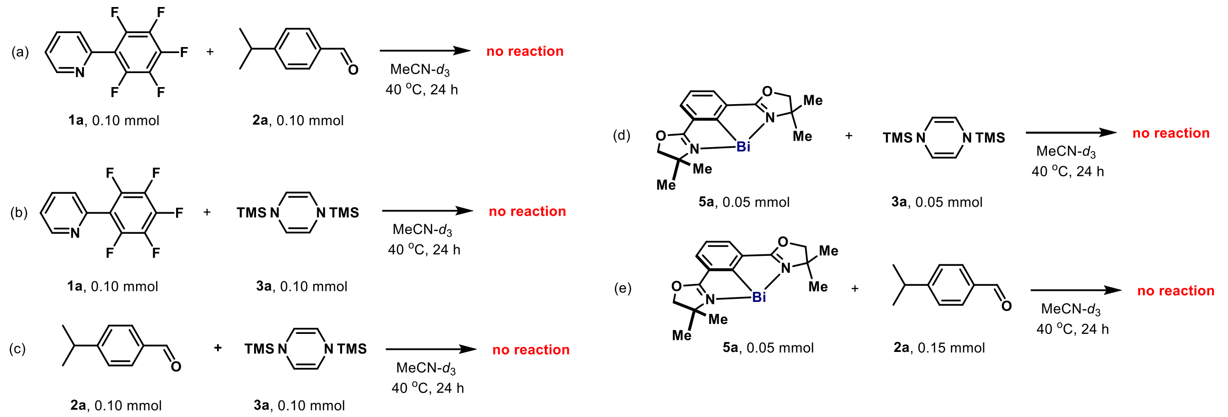

In an Argon-filled glovebox, an NMR tube was charged with the two components as indicated above in MeCN-d_3 (c = 0.20 M). The tube was capped with a septum cap, sealed with parafilm, and taken out of the glovebox. The sample was placed in a 40 °C oil bath for 24 h, and then ^{1}H NMR was recorded at ambient temperature.

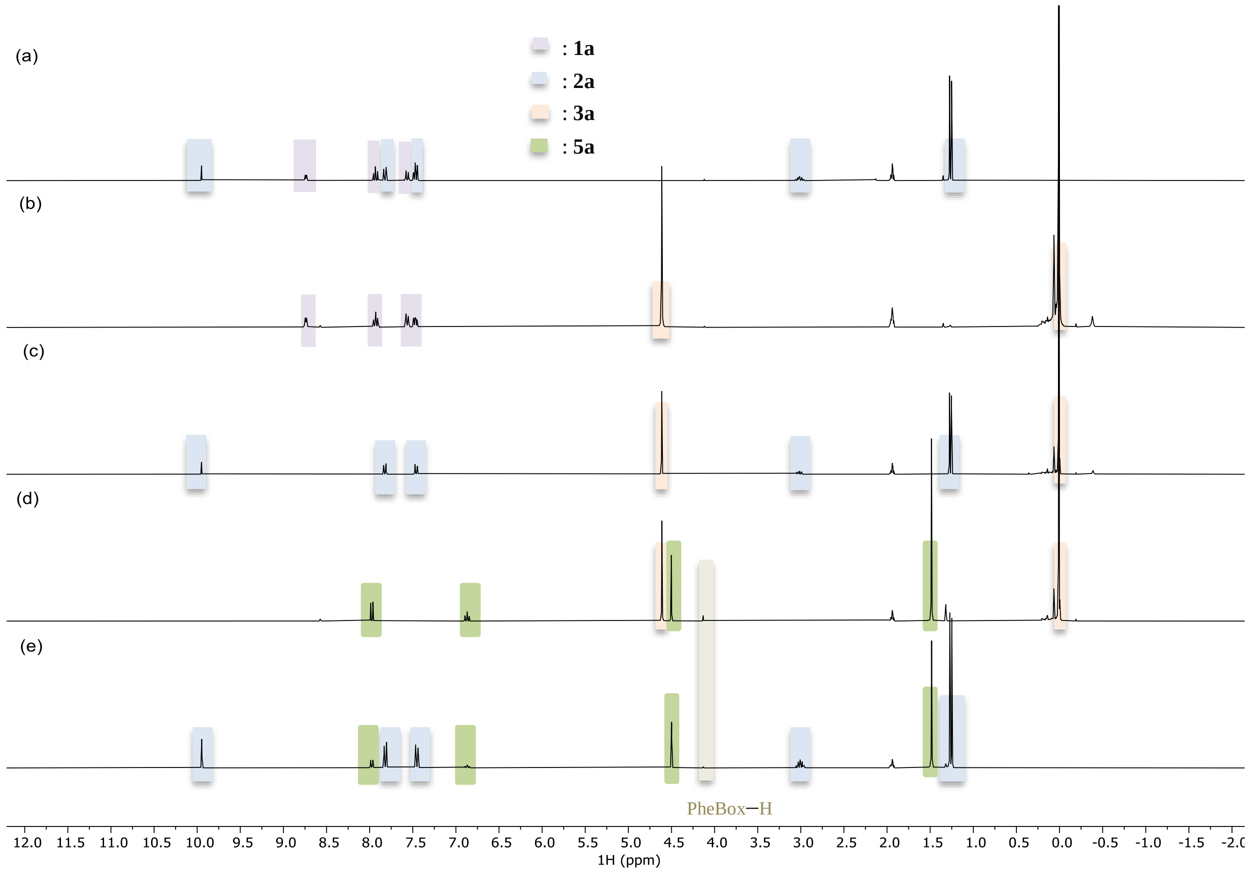

Figure S1. ^{1}H NMR spectra in MeCN-d_3 of control experiments.

<u>*Results*</u>: the cross-experiments of either two of the three reactants (**1a**, **2a** and **3a**) led to no conversion, which excludes pathways initiated by the reactivity among those reactants. Partial decomposition of PheBox-Bi(I) **5a** to protodebismuthylated PheBox–H and Bi metal can be observed when **5a** was mixed with **2a** or **3a** at 40 °C.[6] These results as well as the absence of reactivity when **1a**, **2a** and **3a** were mixed and heat at 40 °C in the absence of PheBox-Bi(I) **5a** (see Table S1, entry 4 – 6) suggest that the reaction is initiated by PheBox-Bi(I) **5a** reacting with polyfluoroarene **1a**.

3.2 Stochiometric reaction between Bi(I) and polyfluoroarene

3.2.1 Reactivtiy between 1a and 5a: kinetic profile

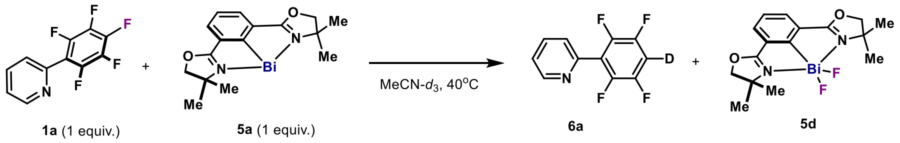

__Procedure__: In an argon-filled glovebox, a J-young NMR tube was charged with **1a** (6.1 mg, 25 μmol), **5a** (12 mg, 25 μmol) and MeCN-d_3 (0.50 mL). The tube was sealed with a Teflon screw cap, removed from the glovebox, then kept at 0 °C and quickly shaken before inserting it into the NMR probe which was prewarmed to 40 °C. After shimming, the NMR spectra were immediately recorded.

Based on the collected NMR data (*vide infra*), it is evident that the C(sp^2)–F bond of **1a** was cleaved by Phebox-Bi(I) **5a**, and the deuterium-defluoronation product **6a** and another Bi species **6x**, bearing the 4-(2-pyridiyl)tetrafluorophenyl moiety, were found to be the major products. In addition, PheBox-BiF$_2$ (**5d**) was formed and involved in an equilibriums between Phebox-Bi(I) (**5a**), Phebox-BiF$_2$ (**5d**) and the unidentified Bi-species. The exact mechanism of these equilibriums remains elusive, but might involve reversible OA and disproportionation processes.[1]

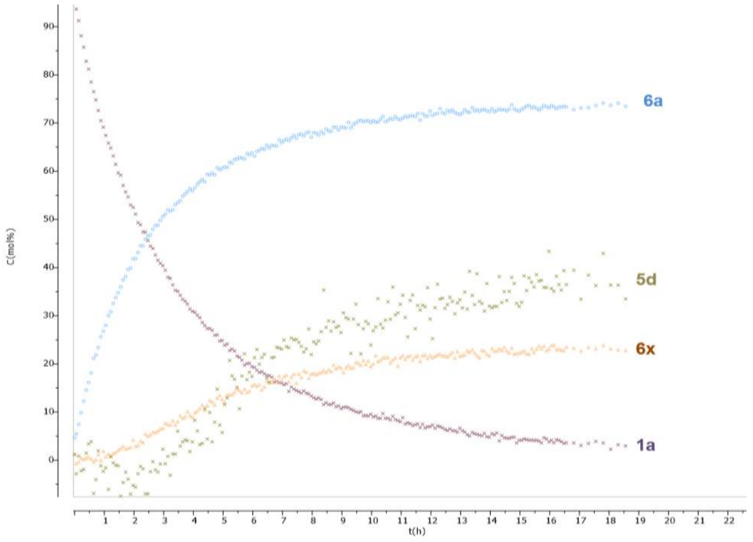

Figure S2. ^{19}F NMR kinetic profile of the reaction between **1a** and **5a** at 40 °C.

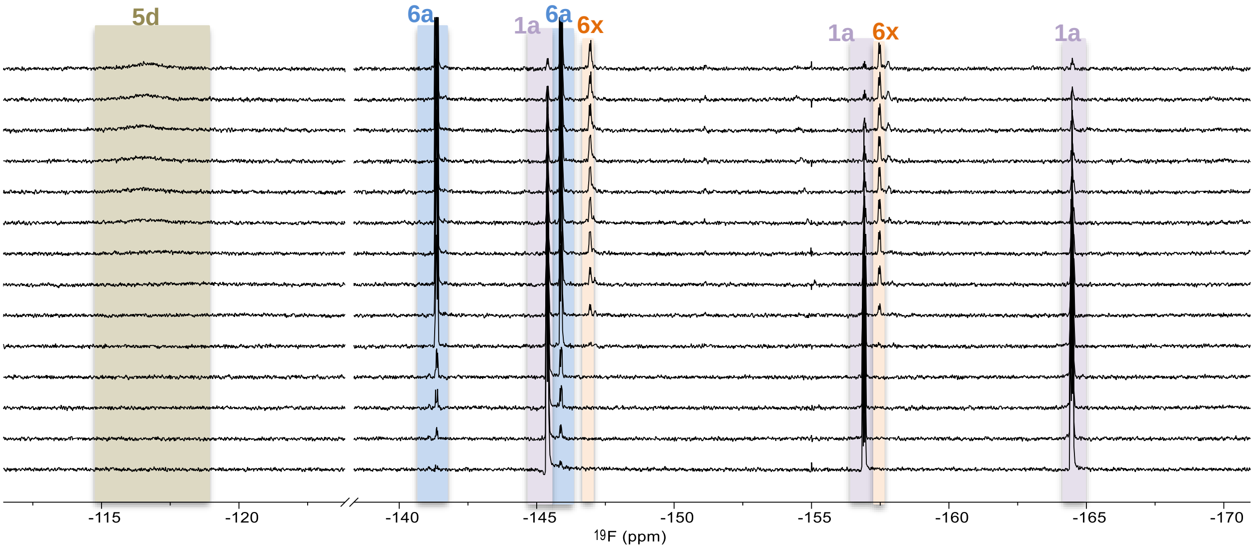

Figure S3. ^{19}F NMR spectra at different time points (600 MHz, MeCN-d_3, 313K).

Note: The profile data for **5d** of the ^{19}F NMR kinetics profile in the initial stage (first 2 h) is attributed to the broad and poorly

resolved signals of PheBox-BiF_2 (**5d**) which arises from an equilibrium involving Phebox-Bi(I) (**5a**), Phebox-BiF_2 (**5d**) and unidentified Bi-species (see below). In this case, the percentage of species shown in the plot represents the relative concentration within the mixture rather than accurate yields. To precisely determine the product yield, we have conducted a stoichiometric experiment of **1a** and **5a** in MeCN-d_3 and analyzed the mixture by quantitative ^{19}F NMR using PhCF_3 as an internal standard, which reveals a 50% of **5d** and 84% of **6a**.

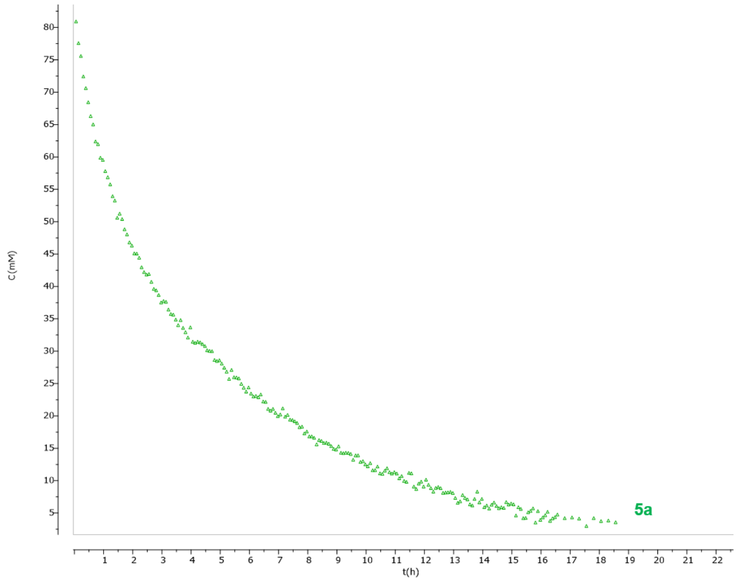

Figure S4. ^{1}H NMR kinetic plot of reaction between **1a** and **5a** at 40 °C; decay of **5a** over time.

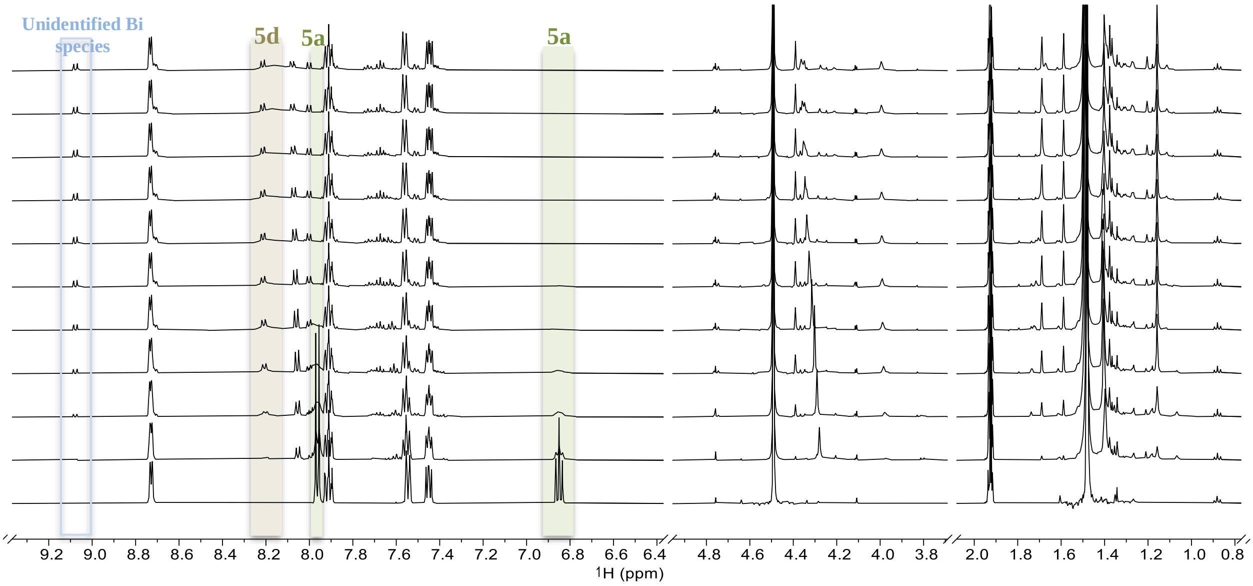

Figure S5. ^{1}H NMR spectra at different time points (600 MHz, MeCN-d_3, 313 K).

The noticeable signal broadening of **5d** and **5a** indicates chemical interconversions. Then we conducted variable temperature ^{1}H NMR for the reaction mixture from 25 °C to –40 °C (*vide infra*). The experiment shows signal sharpening of all species at lower temperatures probably due to a reduced exchange rate. At 263 K (–10.15 °C), **5a** reappeared and its signals sharpened up at 233 K (–40.15 °C), as well as the signals of **5d** which also increased. Yet, the unknown species decreased at lower temperatures, which indicates the equilibrium among **5a** and **5d** and other unidentified species as well.

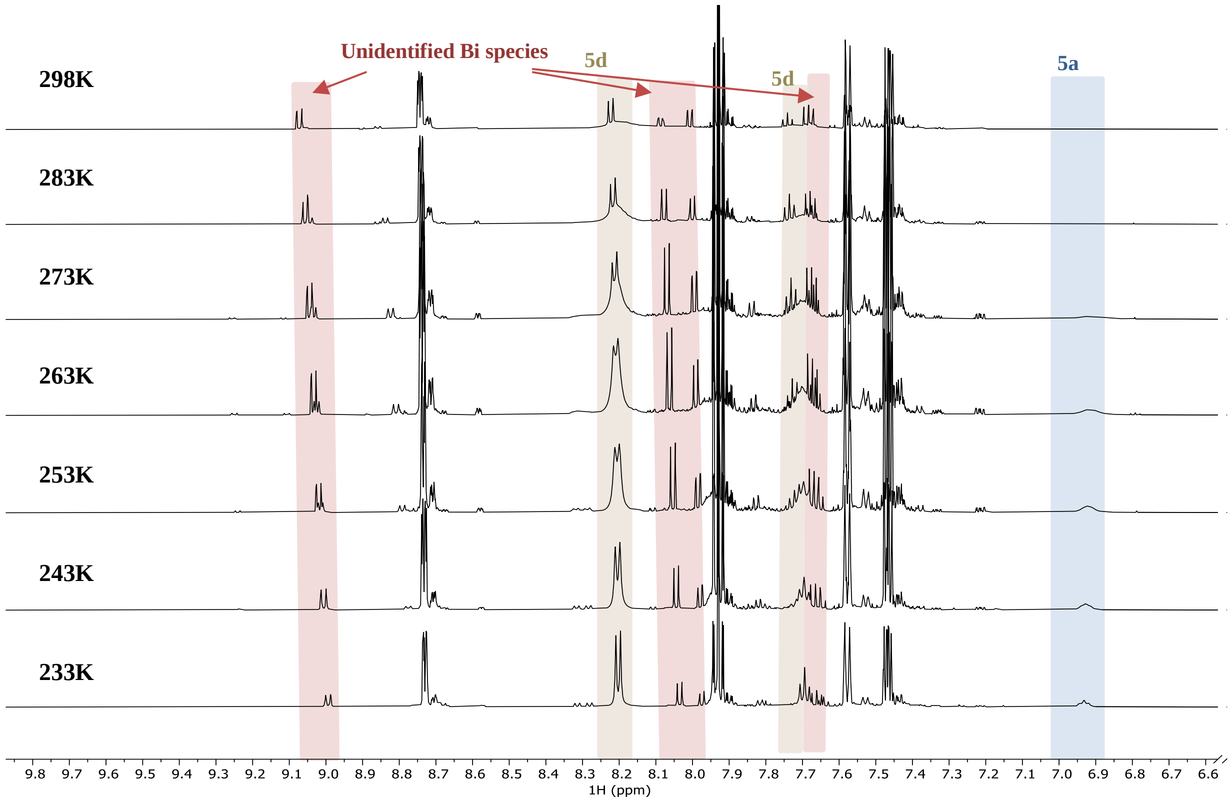

Figure S6. Variable temperature ^{1}H NMR data of the reaction mixture of **1a** and **5a**.

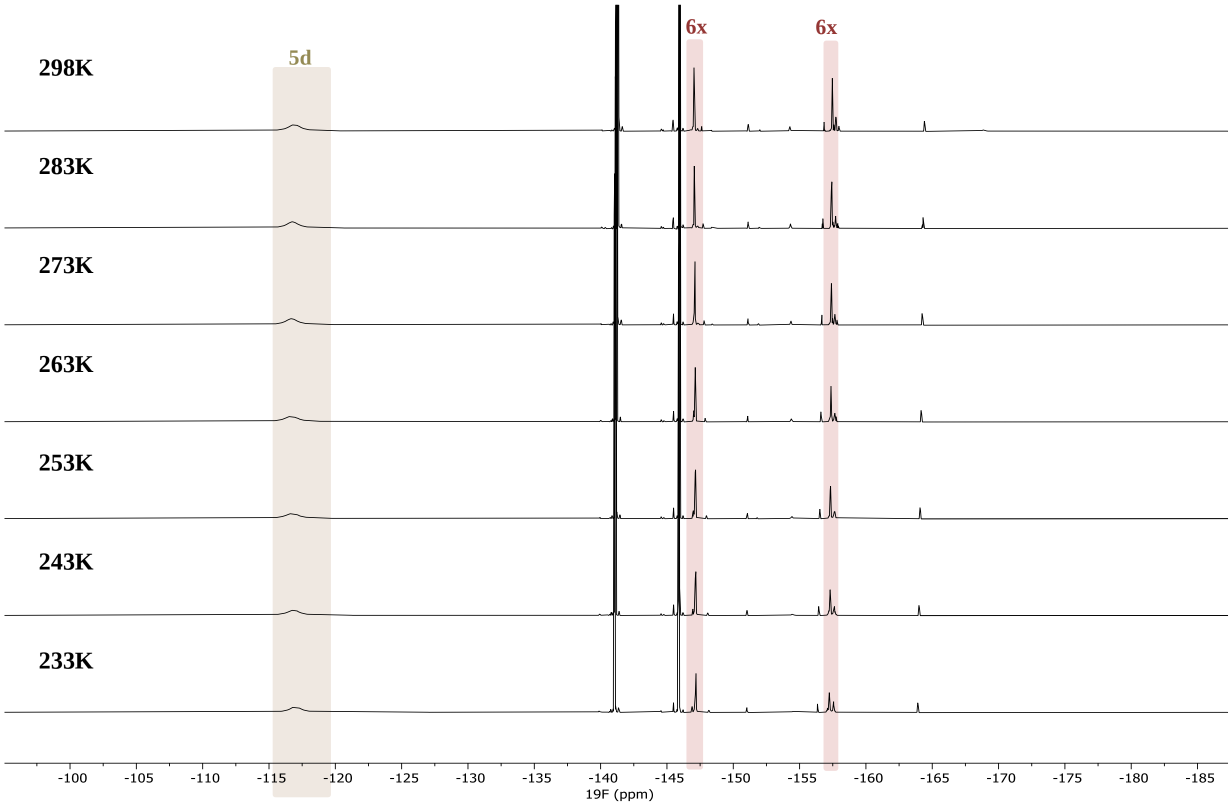

Figure S7. Variable temperature ^{19}F NMR data of the reaction mixture of **1a** and **5a**.

3.2.1.1 Characterization data

6a (extracted NMR spectra from the crude mixture):

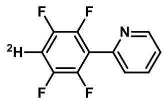

^{1}H NMR (600 MHz, CD_3CN) δ 8.74 (ddd, *J* = 4.8, 1.8, 1.0 Hz, 1H), 7.93 (td, *J* = 7.8, 1.8 Hz, 1H), 7.58 (dp, *J* = 7.8, 1.2 Hz, 1H), 7.46 (ddd, *J* = 7.7, 4.8, 1.2 Hz, 1H).

^{2}H NMR (92 MHz, CD_3CN) δ 7.45

^{19}F NMR (565 MHz, CD_3CN) δ -141.33 (dd, *J* = 21.5, 12.8 Hz), -145.93 (dd, *J* = 21.6, 12.9 Hz).

^{13}C NMR (151 MHz, CD_3CN) δ 151.1, 148.5 (t, *J* = 2.8 Hz), 148.1 – 146.2 (m), 146.1 – 144.1 (m), 138.0, 126.9 (t, *J* = 1.8 Hz), 125.1, 121.8 (t, *J* = 16.7 Hz), 107.0 (t, *J* = 25.2 Hz).

HRMS (ESI-TOF): calc'd for $C_{11}H_5F_4N_1D_1$ $[M+H]^+$: 229.0494, found: 229.0494.

Phebox-BiF_2 (**5d**) (extracted NMR spectra from the crude mixture):

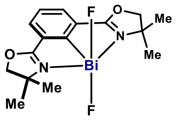

^{1}H NMR (600 MHz, CD_3CN, 233K) δ 8.20 (d, *J* = 7.7 Hz, 2H), 7.69 (t, *J* = 7.7 Hz, 1H), 4.50 (s, 4H), 1.47 (s, 12H).

^{19}F NMR (565 MHz, CD_3CN, 233K) δ -117.00 (br. s).

The spectral data matched with those reported in the literature.[1]

3.2.2 NMR analysis for the reaction between 1a and 5a (MeCN-d_3)

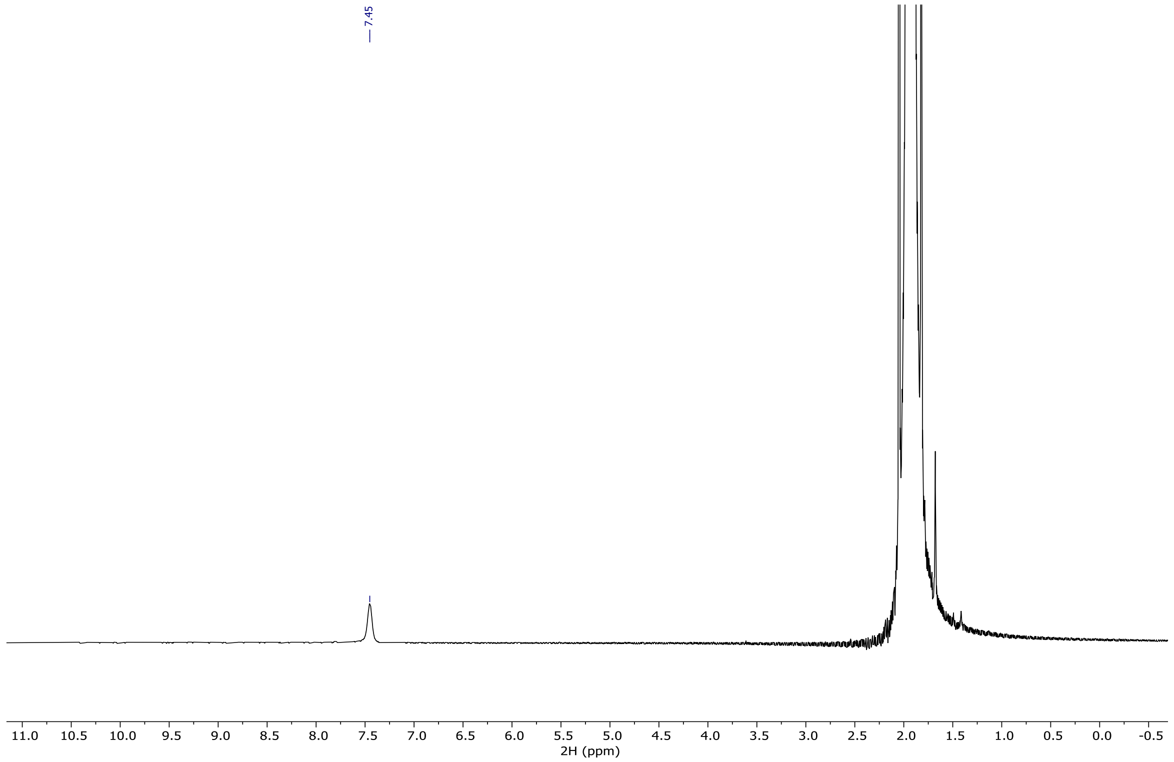

Figure S8. ^{2}H NMR spectrum of the crude mixture acquired after the reaction between **1a** and **5a** (92 MHz, MeCN-d_3, 298K).

The ^{2}H NMR spectrum shows the presence of deuterated aromatic compound which was further assigned to **6a** by NMR and HRMS.

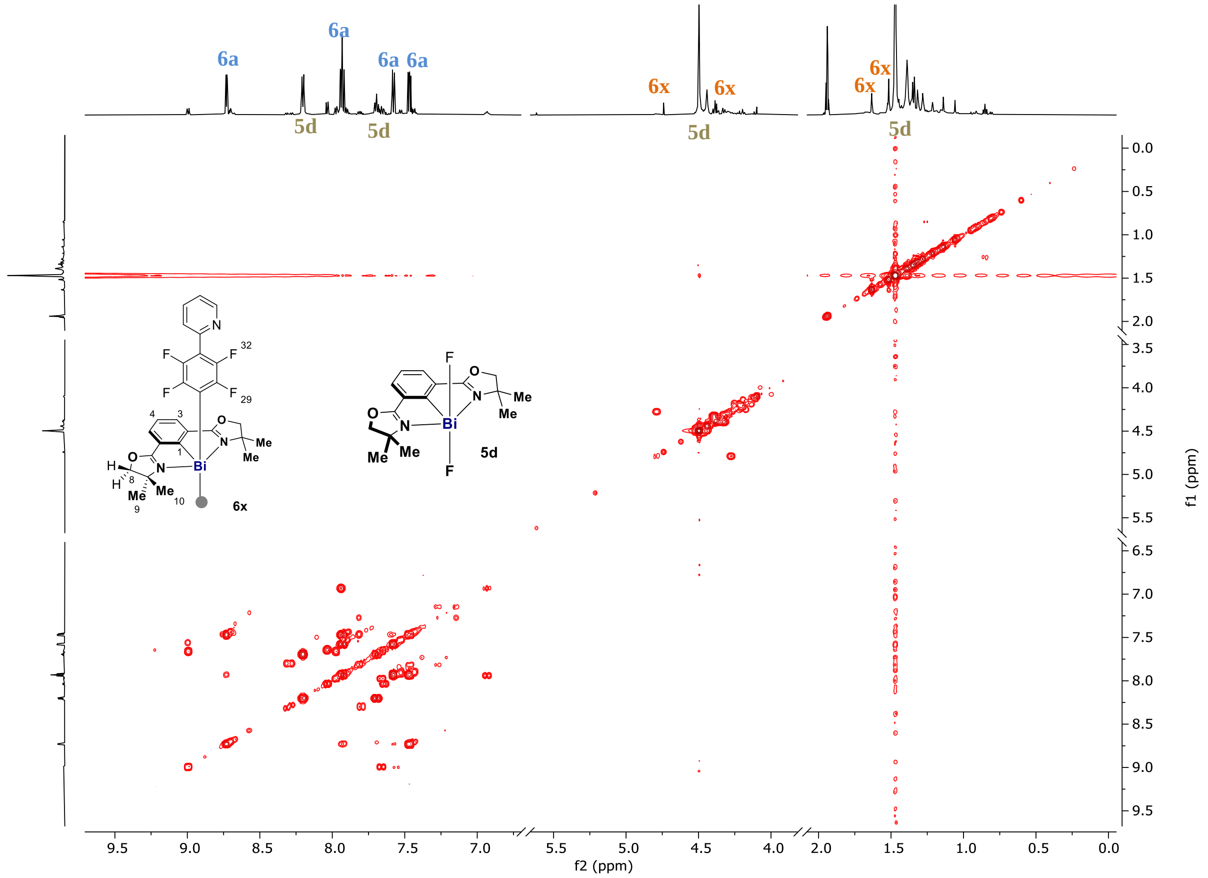

Figure S9. ^{1}H-^{1}H COSY spectrum of the crude mixture acquired after the reaction between **1a** and **5a** (600 MHz, MeCN-d_3, 233K).

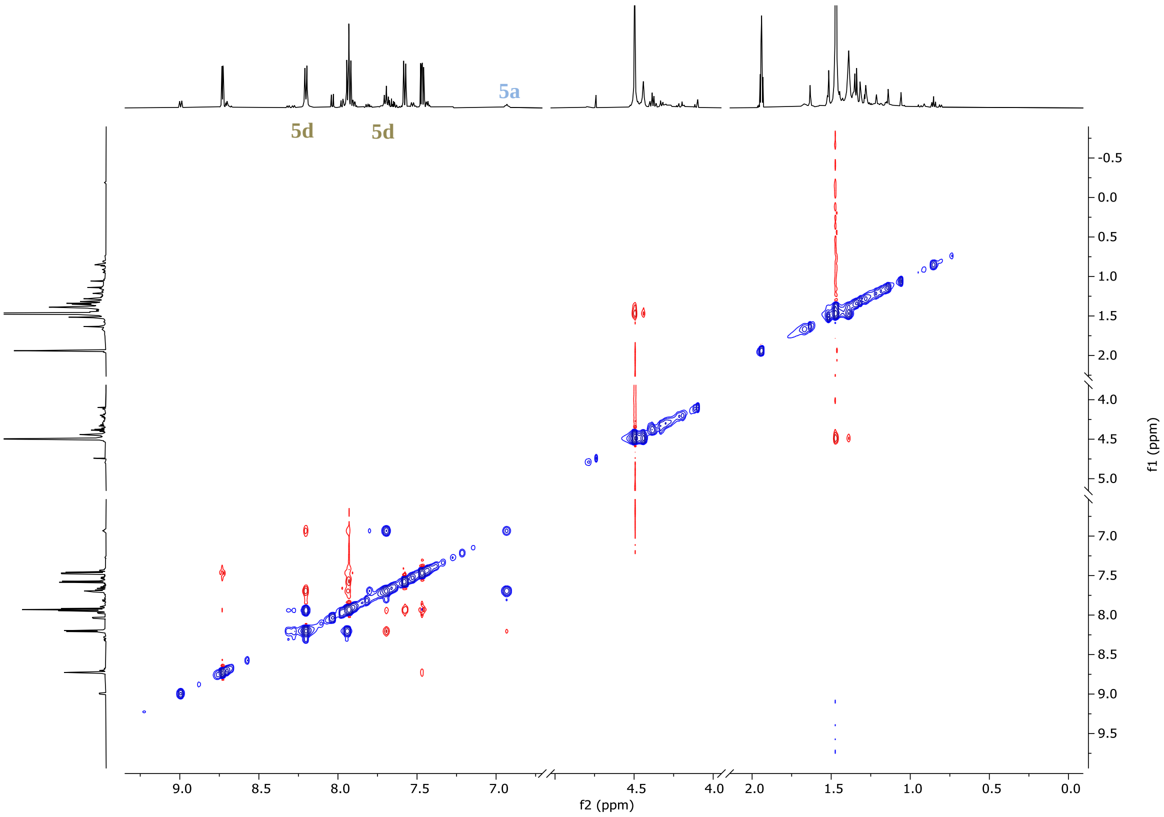

Figure S10. ^{1}H-^{1}H NOESY spectrum of the crude mixture acquired after the reaction between **1a** and **5a** (600 MHz, MeCN-d_3, 233K).

EXSY crosspeaks in the ROESY spectrum at 233K indicates the chemical interconversion between **5a** and **5d**.

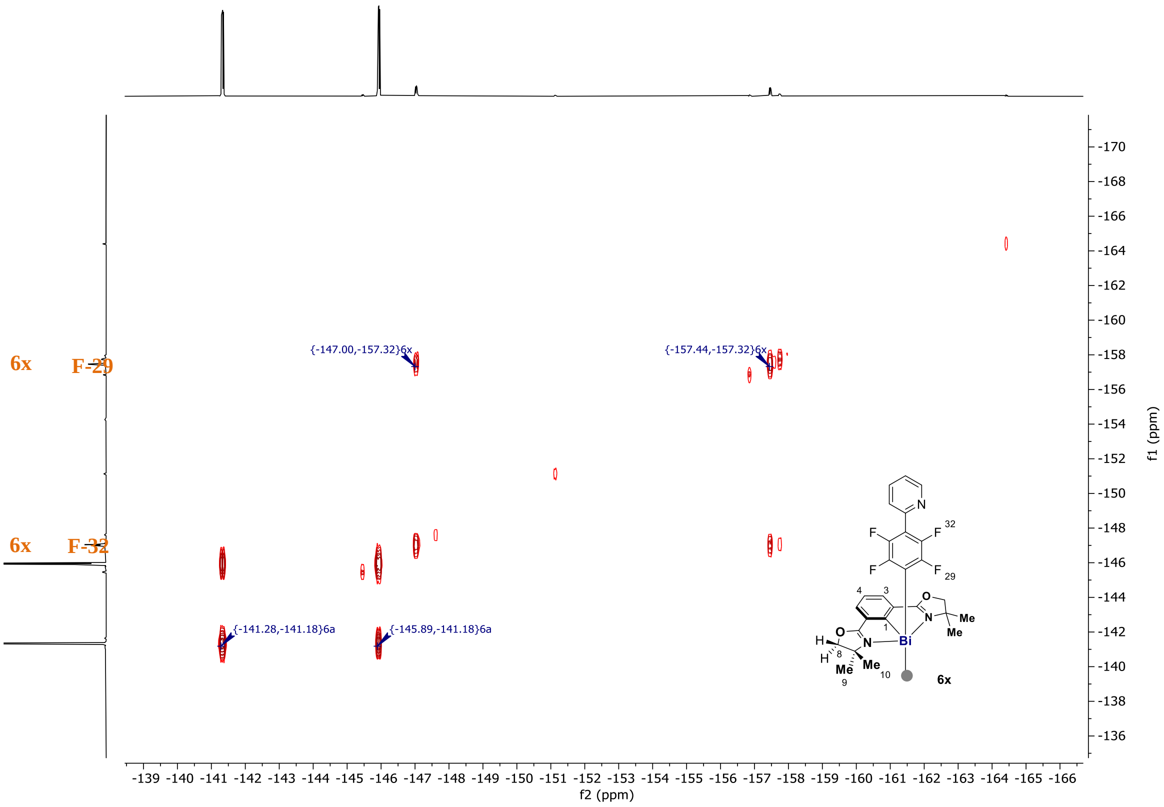

Figure S11. ^{19}F-^{19}F COSY spectrum of the crude mixture acquired after the reaction between **1a** and **5a** (565 MHz, MeCN-d_3, 298K).

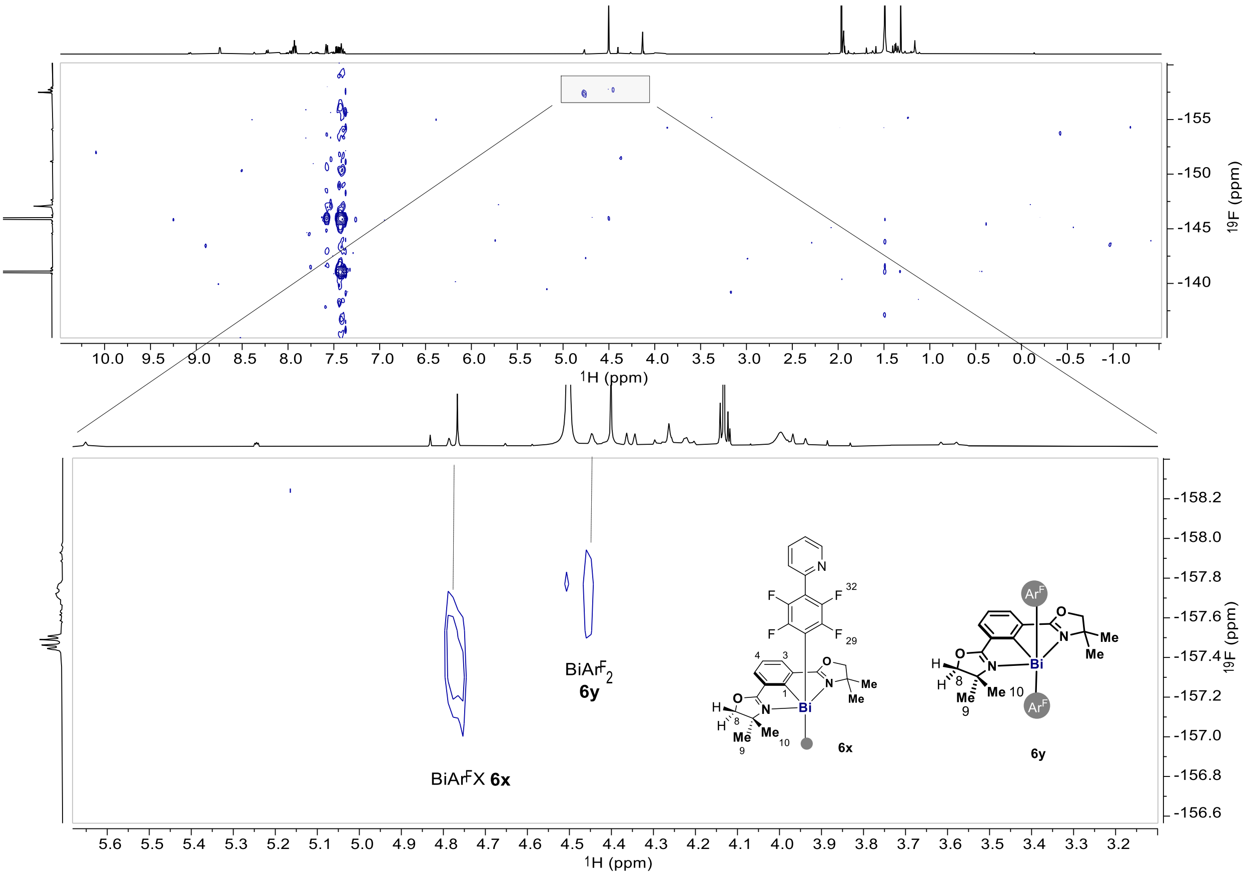

Figure S12. ^{1}H-^{19}F COSY spectrum of the crude mixture acquired after the reaction between **1a** and **5a** (600 HMz, MeCN-d_3, 298K).

The spectra reveals the spatial proximity of the 4-tetrafluorophenyl to the CH_2 of the oxazoline ligand as a through-space coupling between F-29 and H-8 is observed in the ^{1}H-^{19}F COSY spectrum, suggesting the connectivity of the 4-(2-pyridiyl)tetrafluorophenyl and PheBox-ligand through the Bi center in **6x**. ^{1}H-^{19}F COSY and ^{1}H-^{1}H COSY spectra shows that **6x** has asymmetric PheBox backbones as indicated by the inequivalence of CH_2 (H-8) and CH_3 (H-9/H-10) groups of the oxazolines, which is in line with proposed structure of **6x**. However, no unambiguous confirmation and assignment of the structure of **6x** could be made due to its low concentration, peak overlapping and moisture sensitivity. Nevertheless, it could be concluded that the C(sp^{2})–F bond of pentafluoroarene **1a** was cleaved by Bi(I) **5a**.

3.2.3 Stochiometric treatment of the OA mixture with 2a and 3a:

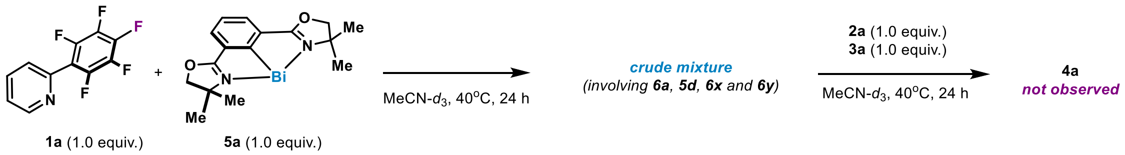

The crude mixture acquired after the reaction of **1a** and **5a** was treated with stochiometric amount of **2a** and **3a** inside an NMR tube, removed from the glovebox, heated at 40 °C for 24 h, and then analyzed by NMR.

The NMR data (Fig S13) shows that treatment with **2a** and **3a** led no formation of the model product **4a** which excludes potential pathways through **6x**. In addition, ^{19}F NMR spetra indicates that the formed Bi-based species **6x** and **5d** were fully consumed in the presence of **3a**, whereas deutero/hydro-defluorination product **6a** and **6b** were observed as major products. This is consistent with the proposed structure of Bi(III)(Ar^{F})X species **6x**. It is assumed that **6x** is generated from the postulated oxidative addition intermediate **5c**.

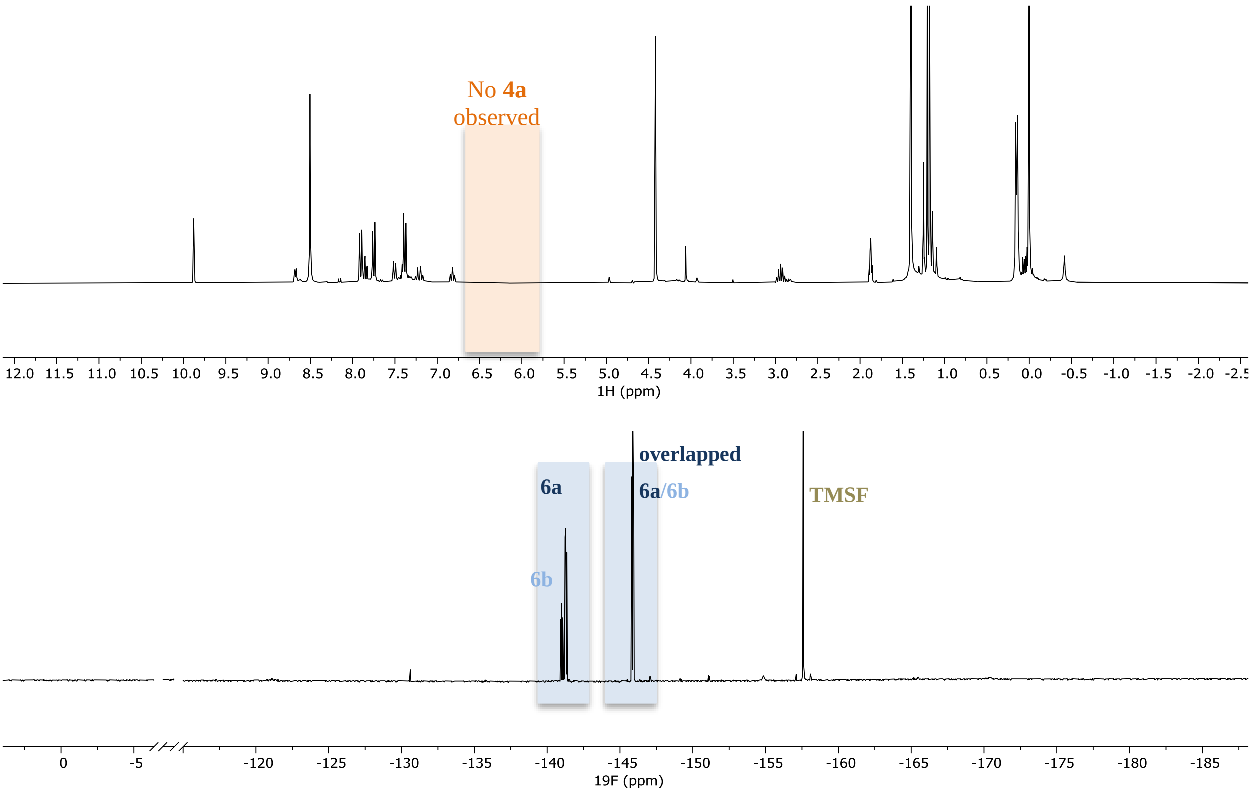

Figure S13. ^{1}H NMR (*upper*) and ^{19}F NMR (*bottom*) spectra for the reaction of the OA mixture (**1a** + **5a**) with **2a** and **3a** (300 MHz, MeCN-d_3, 298 K).

3.2.4 Reaction of 1a with 5a in the presence of LiOTf

The complexity derived for the equilibrium for intermediate **5c** and the sensitivity towards MeCN prevented complete characterization of the intermediates during the C(sp^2)–F bond cleavage. We speculated that fluoride abstraction with LiOTf could lead to the cationic species Phebox-Bi(4-(2-pyridyl)-tetrafluorophenyl) triflate (**5f**) with enhanced stability, as previsouly observed in other work from our laboratory.[1] Thus we conducted a stochiometric reaction of **1a** with **5a** in the presence of LiOTf. NMR analysis fo the mixture reveals PheBox-Bi(III)-CH$_2$CN adduct **5e** as the major Bi-based species. Interestingly, HRMS analysis of the mixture identified the proposed PheBox-Bi(III)-Ar^F **5f** (*vide infra*).

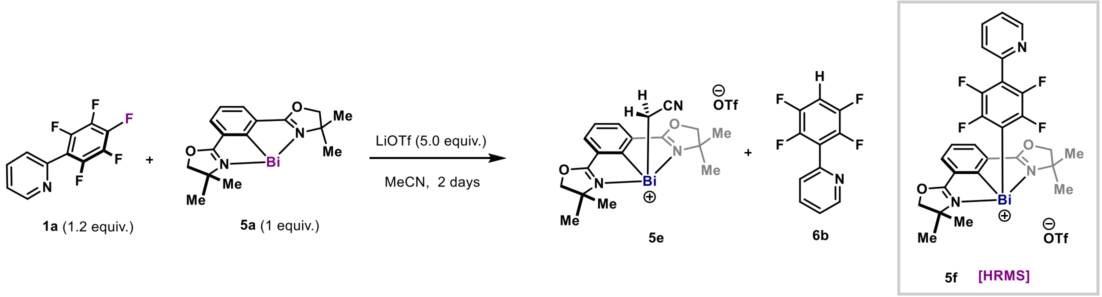

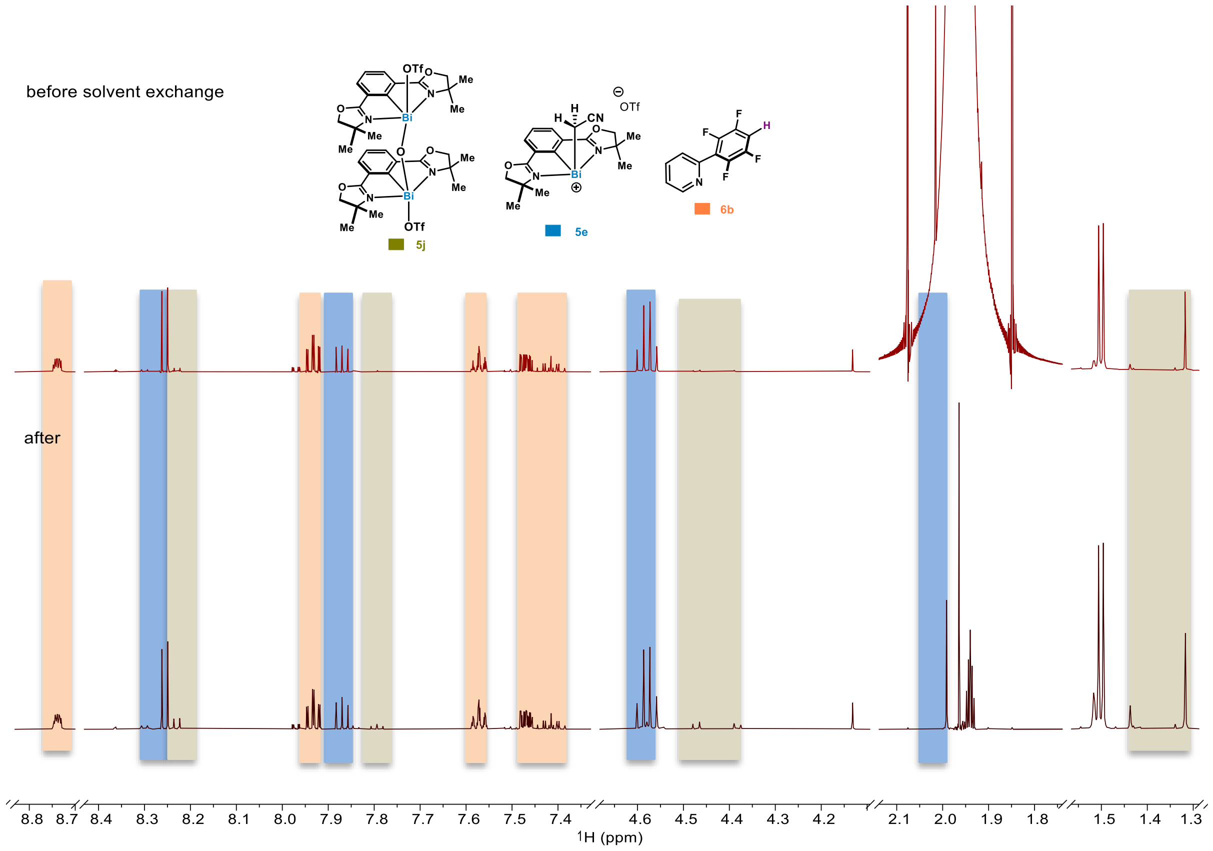

Figure S14. ^{1}H NMR spectra: upper spectra in MeCN, bottom spectra in MeCN-d_3 (600 MHz, 298 K).

NMR indicates that PheBox-Bi(CH_2CN)OTf **5e** was found to be the major Bi-based species accompanied by the formation of HDF product **6b**. In addition, partial hydrolysis of **5e** occured and formed the oxo-bismuth byproduct **5j** during the solvent exchange, which indicates that **5e** is highly moisture sensitive. (See detailed 2D NMR speatra and assignments for **5e** in NMR spectra section)

Nontheless, the crude reaction mixture was concentrated in a Schlenk tube and submited to HRMS analysis under argon. The proposed PheBox-Bi(III)–Ar^F **5f** was observed by HRMS analysis albeit with low intensity. It is believed that the deprotonation of MeCN by the polarized and basic Bi(III)–Ar^F species leads to **5e** and releases **6b**.

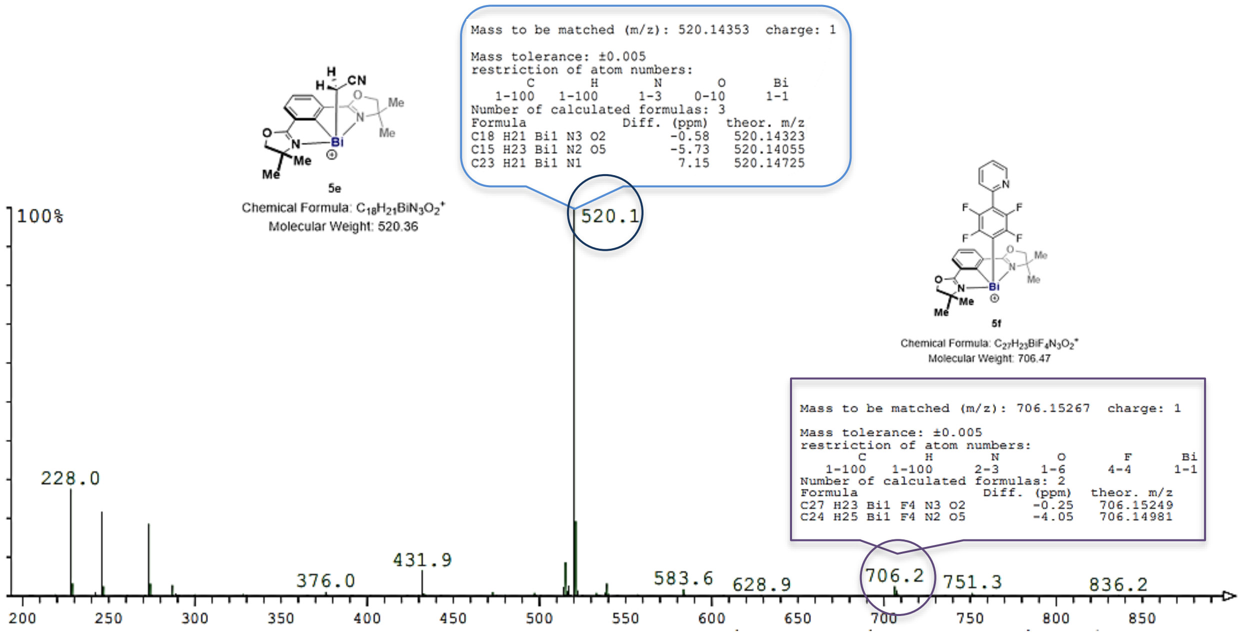

Figure S15. HRMS data of the crude mixture of **1a** and **5a** in presence of LiOTf.

3.2.4.1 Characterization data

6b (extracted NMR spectra from the crude mixture):

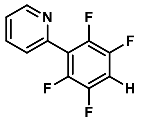

^{1}H NMR (600 MHz, CD_3CN) δ 8.74 (dtt, *J* = 4.3, 1.8, 0.9 Hz, 1H), 7.93 (tdd, *J* = 7.8, 1.8, 0.4 Hz, 1H), 7.57 (tp, *J* = 7.8, 1.2 Hz, 1H), 7.47 (dddd, *J* = 7.7, 4.9, 2.3, 1.2 Hz, 1H), 7.41 (tt, *J* = 10.3, 7.5 Hz, 1H).
^{19}F NMR (565 MHz, CD_3CN) δ -140.98 – -141.09 (m), -145.80 – -145.92 (m).
The spectral data matched with those reported in the literature.[1]

PheBox-Bi(CH_2CN)OTf **5e** (extracted NMR spectra from the crude mixture):

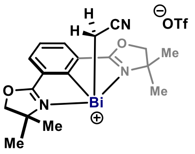

^{1}H NMR (600 MHz, CD_3CN) δ 8.26 (d, *J* = 7.7 Hz, 2H), 7.90 – 7.85 (m, 1H), 4.59 (d, *J* = 8.8 Hz, 2H), 4.57 (d, *J* = 8.8 Hz, 2H), 1.99 (s, 2H), 1.51 (s, 6H), 1.50 (s, 6H).
^{13}C NMR (151 MHz, CD_3CN) δ 192.2, 180.8, 135.2, 133.4, 131.4, 121.7 (q, *J* = 319.5 Hz), 120.0, 84.2, 68.0, 30.7, 28.6, 27.7.
^{19}F NMR (565 MHz, CD_3CN) δ -79.42.
HRMS (ESIpos, m/z): calc'd for $C_{18}H_{21}BiN_3O_2$ $[M-CF_3O_3S]^+$: 520.1432, found: 520.1435.

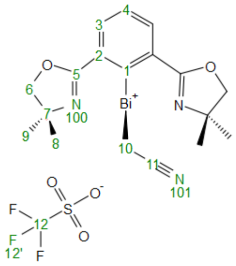

Atom	δ (ppm)	J	COSY	HSQC	HMBC	NOESY
1 C	192.200				3,10	
2 C	133.416				4	
3 C	135.176			3	3	
H	8.256	7.70(4)	4	3	1, 3, 5	6', 6"
4 C	131.387			4		
H	7.870	7.70(3)	3	4	2	
5 C	180.752				3, 6', 6"	
6 C	84.165			6', 6"	8, 9	
H'	4.594	8.80(6")	6"	6	5, 8, 9	3
H"	4.566	8.80(6')	6'	6	5, 8, 9	3
7 C	67.983				8, 9	
8 C	27.679			8	6', 6", 9	
H3	1.506			8	6, 7, 9, 100	10
9 C	28.610			9	6', 6", 8	
H3	1.496			9	6, 7, 8, 100	
10 C	30.712			10		
H2	1.991			10	1, 11, 100, 101	8
11 C	119.971				10	
12 C	121.722					
12' F	-79.416					
100 N	-165.276				8, 9, 10	
101 N	-132.978				10	

5j (extracted NMR spectra from the crude mixture):

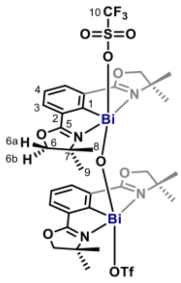

^{1}H NMR (600 MHz, CD_3CN) δ 8.23 (d, *J* = 7.6 Hz, 2H), 7.79 (t, *J* = 7.6 Hz, 1H), 4.47 (d, *J* = 8.7 Hz, 4H), 4.38 (d, *J* = 8.7 Hz, 4H), 1.44 (s, 6H), 1.32 (s, 6H).

The spectral data matched with those reported in the literature.[1]

3.3 Radical trapping experiments

Considering the recent reports of organobismuth(I) complexes engaging in radical processes,[7] as well as the observation of deuterated product **6a**, we also evaluated the possibility of radical involved single electron transfer (SET) pathway.

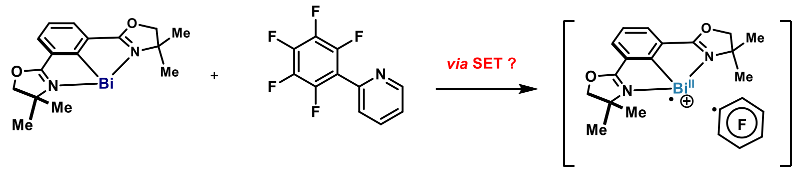

Procedure: A culture tube with a Teflon screw-cap equipped with a Teflon-coated stir bar was used. 2-(Perfluorophenyl) pyridine **1a** (12 mg, 0.05 mmol, 1.0 equiv.) and 4-isopropylbenzaldehyde **2a** (23 μL, 0.15 mmol, 3.0 equiv.) were introduced into the culture tube. Then, **5a** (1.2 mg, 2.5 μmol, 0.05 equiv.), **3a** (0.05 mmol, 1.0 equiv.), radical scavenger (0.05 mmol, 1.0 equiv.) and MeCN (0.25 mL) were added inside the glovebox. The reaction tube was taken outside the glovebox and the mixture was stirred at 40 °C for 16 h. After completion of the reaction, the solvent was removed under reduced pressure. The crude mixture was diluted with 0.50 mL of $CDCl_3$, trichloroethene (9.0 μL, 0.10 mmol, 2.0 equiv.) was added as the internal standard, and quantitive ^1H NMR was recorded to determine the yield.

(a) TEMPO as radical scavenger

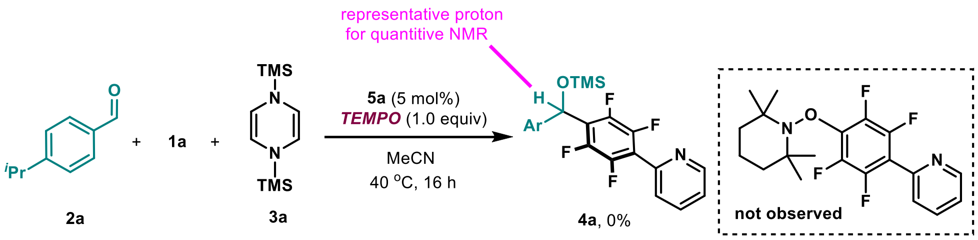

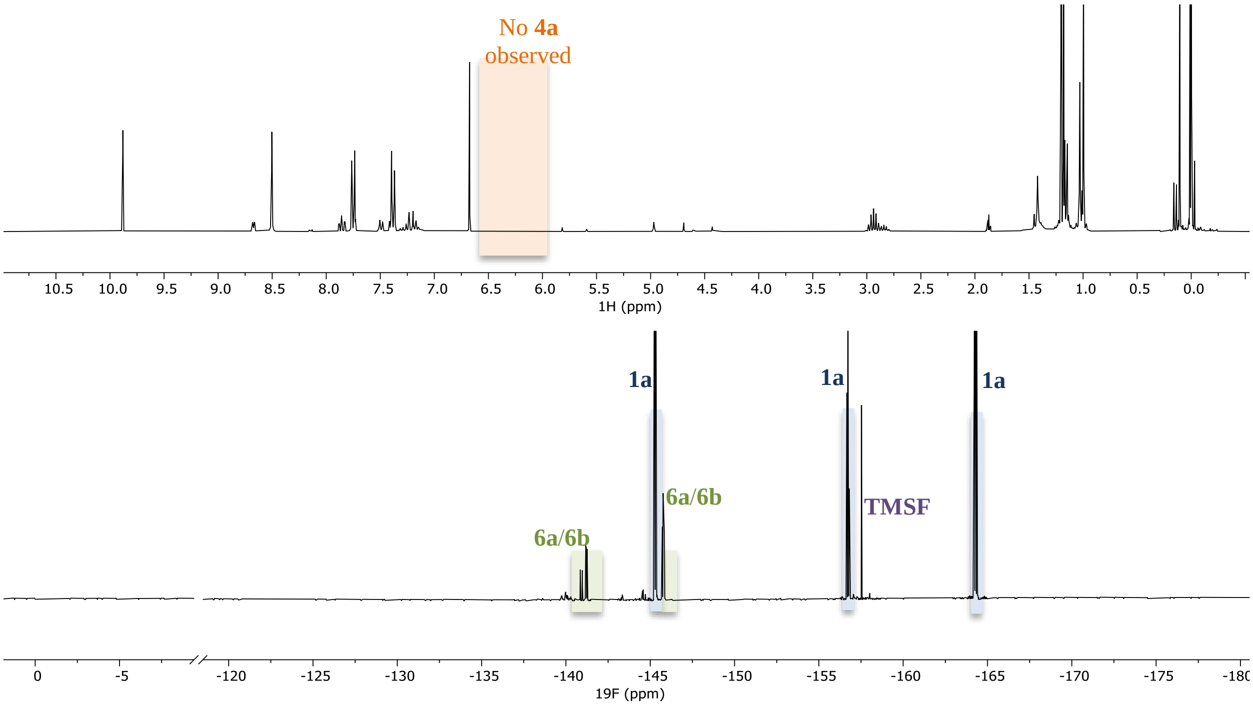

Figure S16. Crude ^1H NMR (*upper*) and ^{19}F NMR (*bottom*) spectra of the radical trapping experiment (**a**).

The NMR spectra shows no formation of product **4a** nor radical trapping product; most of **1a** remained unreactive due to the side reactitivity between TEMPO and Bi(I) **5a**.

(b) BHT as radical scavenger

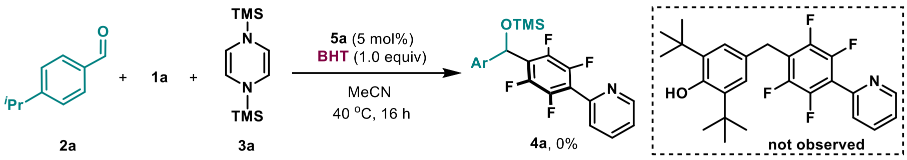

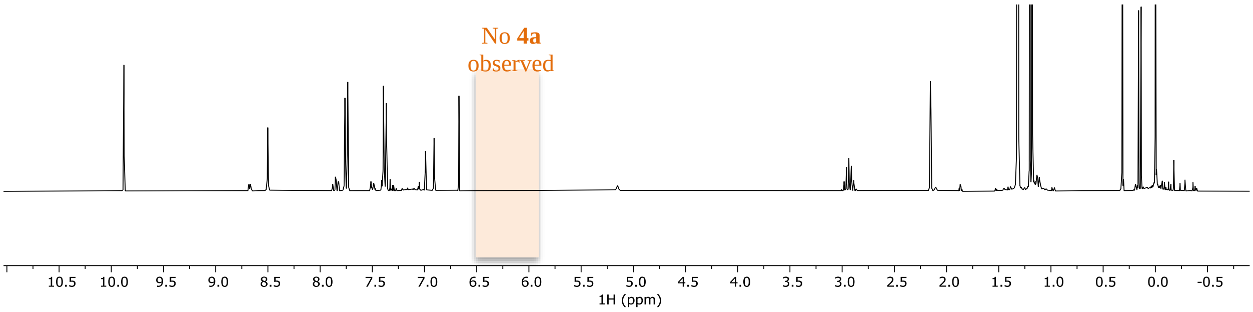

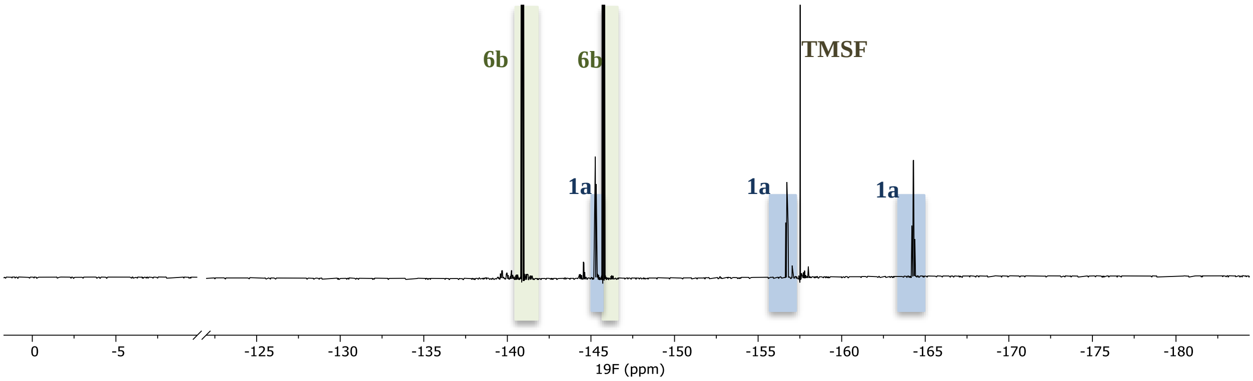

Figure S17. Crude ^{1}H NMR (*upper*) and ^{19}F NMR (*bottom*) spectra of the radical trapping experiments **(b)**.

The NMR spectra shows no formation of product **4a** nor radical traping product; **6b** was found to be the major product, which was attributed to the incompatibility between TMS-DHP **3a** and the oxidant scavenger.

c) DPE as radical scavenger

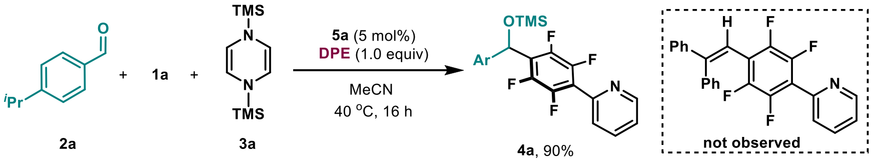

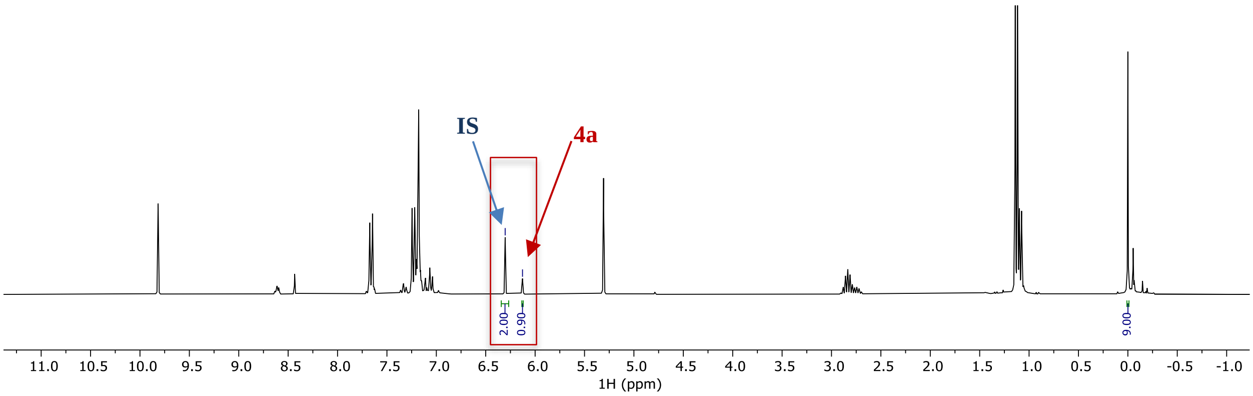

Figure S18. Crude ^{1}H NMR of the radical trapping experiment (**c**).

3.4 Cyclic voltammetry

Cyclic voltammograms were collected using a 3-electrode cell consisting of a 1.6 mm diameter glassy carbon disc working electrode, platinum wire as the counter electrode, and a silver wire pseudoreference electrode at ambient temperature in an argon-filled glovebox equipped with electrochemical outlets (all CVs are scanned by starting at open circuit potential). Sublimed ferrocene was added at the end of each experiment as the internal reference.

Though electrochemically irreversible, the reduction onset potential of **1a** (–2.39 V vs $Fc^{+/0}$) differs by *ca.* 1.4 V from the quasi-reversible $E_{1/2}$ of **5a**. This translates to SET event that is approximately 32 kcal/mol uphill, which allows us to exclude an SET-based oxidative addition process.

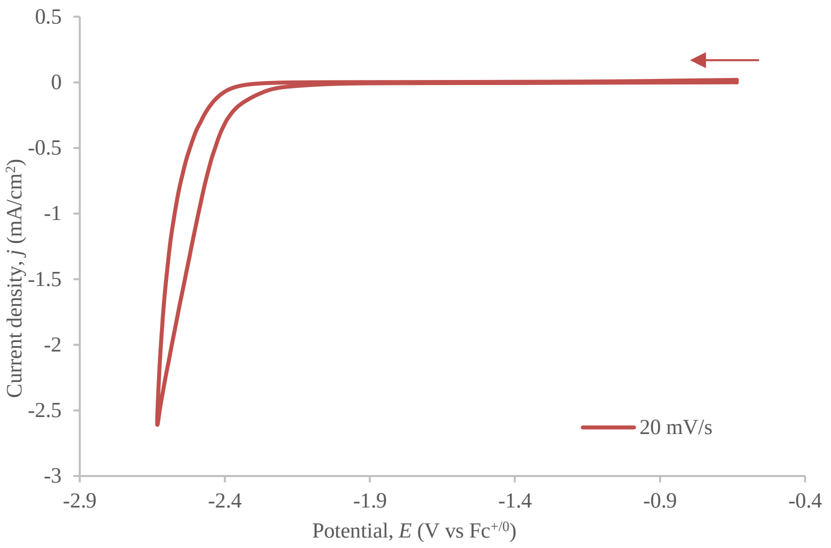

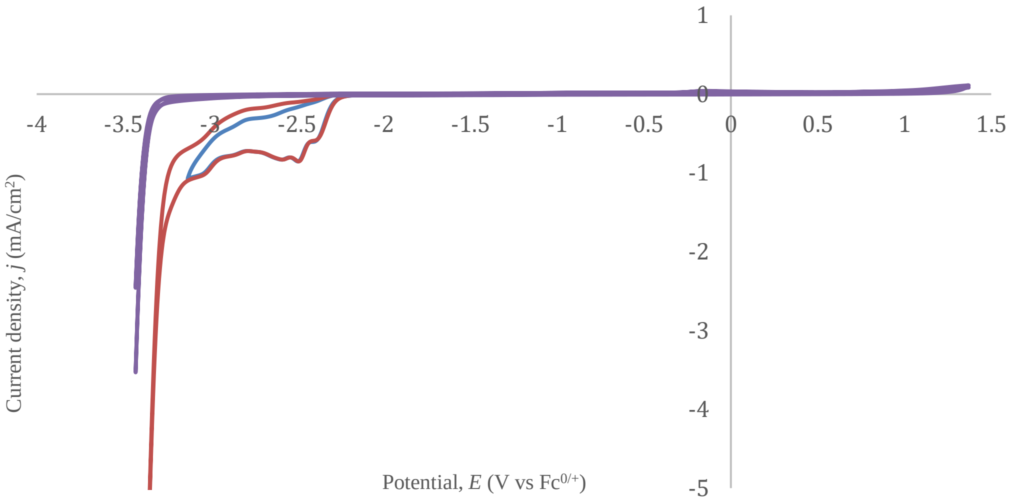

Figure S19. Cyclic voltammogram of **1a** in MeCN (*upper*) with E_{onset} = –2.39 V vs $Fc^{+/0}$ (1.0 mM **1a** , 0.10 M [*n*Bu_4N][PF_6] supporting electrolyte, T = 298 K, scan rate = 20 mV/s); Cyclic voltammogram comparison of **1a** and MeCN in a broader potential window (*bottom*)

The cyclic voltammogram (Fig S19, bottom) clearly differentiates the electrochemical behavior of MeCN (purple) from that of the substrate (blue and red). As shown, the substrate exhibits a well-defined cathodic feature at approximately –2.39 V vs $Fc^{0/+}$, while MeCN shows no noticeable reduction wave within this range. Upon extending the scan to a broader potential window, the onset of MeCN reduction occurs consistently around –3.0 V vs $Fc^{0/+}$. These observations unambiguously attribute the cathodic feature at –2.39 V vs $Fc^{0/+}$ to the substrate, thereby excluding solvent decomposition as its origin.

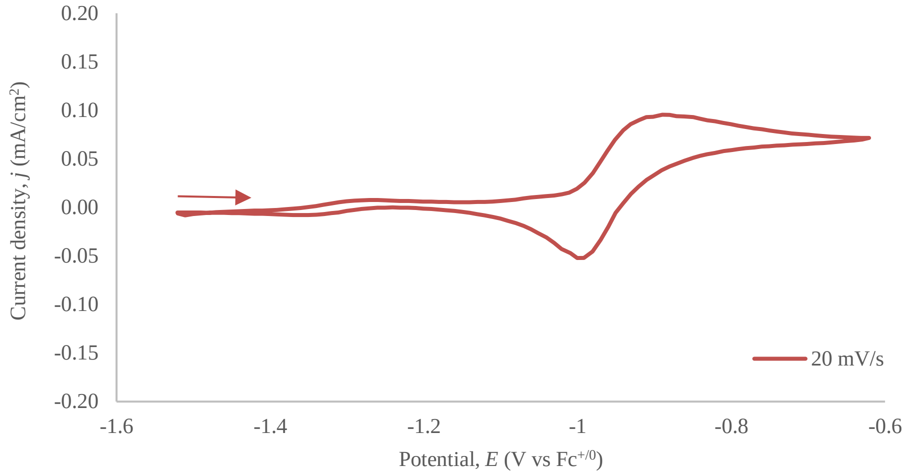

Figure S20. Cyclic voltammogram of **5a** in MeCN with $E_{1/2}$ = –0.97 V vs $Fc^{+/0}$ (1.0 mM **5a**, 0.10 M [$n$$Bu_4N$][$PF_6$] supporting electrolyte, T = 298 K, scan rate = 20 mV/s).

3.5 Studying the migratory insertion

3.5.1 Stoichiometric reaction of 1a, 5a and 2a

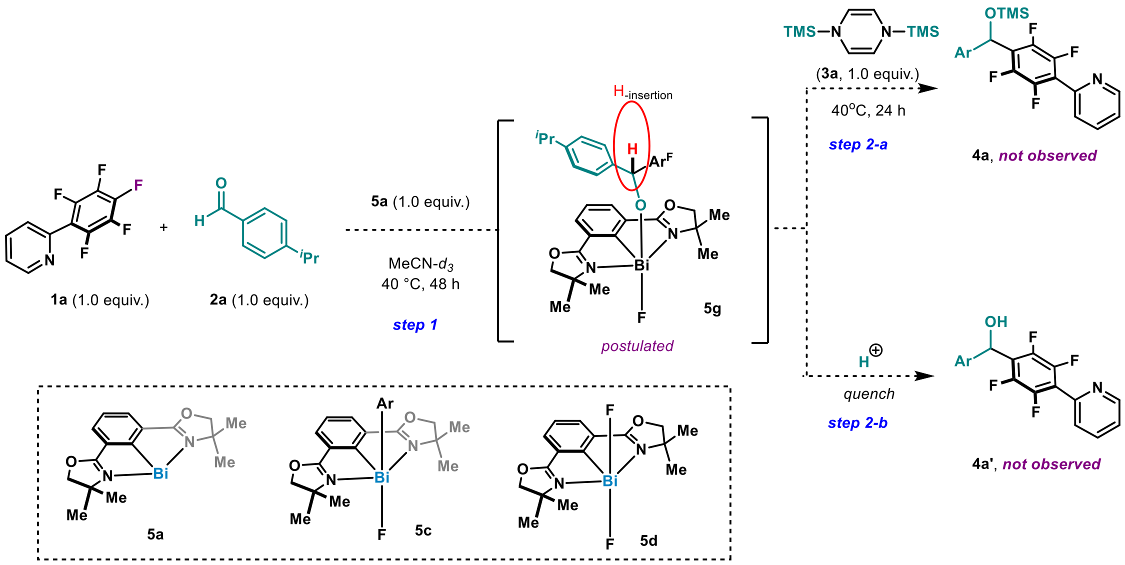

Step 1: In an argon-filled glovebox, a J-young tube was charged with Phebox-Bi(I) **5a** (12 mg, 25 µmol), **1a** (6.1 mg, 25 µmol, 1.0 equiv.), **2a** (3.8 µL, 25 µmol, 1.0 equiv.) and MeCN-d_3 (0.50 mL). The tube was closed, removed from the glovebox, and put in a 40 °C oil bath for 48 h. Then NMR analysis was performed at 25 °C.

The NMR spectra (*vide infra*) indicates the absence of the postulated insertion intermediate **5g** (it is assumed the C-H signal $\delta_{H\text{-insertion}}$ in **5g** to appear between 6.0 – 7.0 ppm according to the products and reported alkoxy bismuth(III) skeletons[8]) or product **4a'**. The aldehyde **2a** was remained unreacted after heating for 48 h. Instead, **6a** as well as PheBox-BiF_2 **5d** were found to be the major products, which shares similarities to the stoichiometric reaction between **1a** and **5a** in the absence of **2a**. In spite of the minor unknown species observed in the ^{19}F NMR, aldehyde remains unreactive towards the postulated OA intermediate **5c**.

Step 2-a: The mixture obtained in ***step 1*** was treated with **3a** (5.7 mg, 25 µmol, 1.0 equiv.) inside the glovebox, and the sample was analysed by NMR. **5a** was regenerated and no desired reductive arylation product **4a** was observed. This result further precludes pathways *via* possible intermediates from the reaction between **2a** and **5c**.

Step 2-b: To the mixture of ***step 1*** was added HCl (25 µL, 2.0 M in water, 2.0 equiv.) under argon, then NMR was recorded. The

data shows that **4a'** was not formed under acidic conditions, which indicates that migratory insertion into aldehyde did not occur.

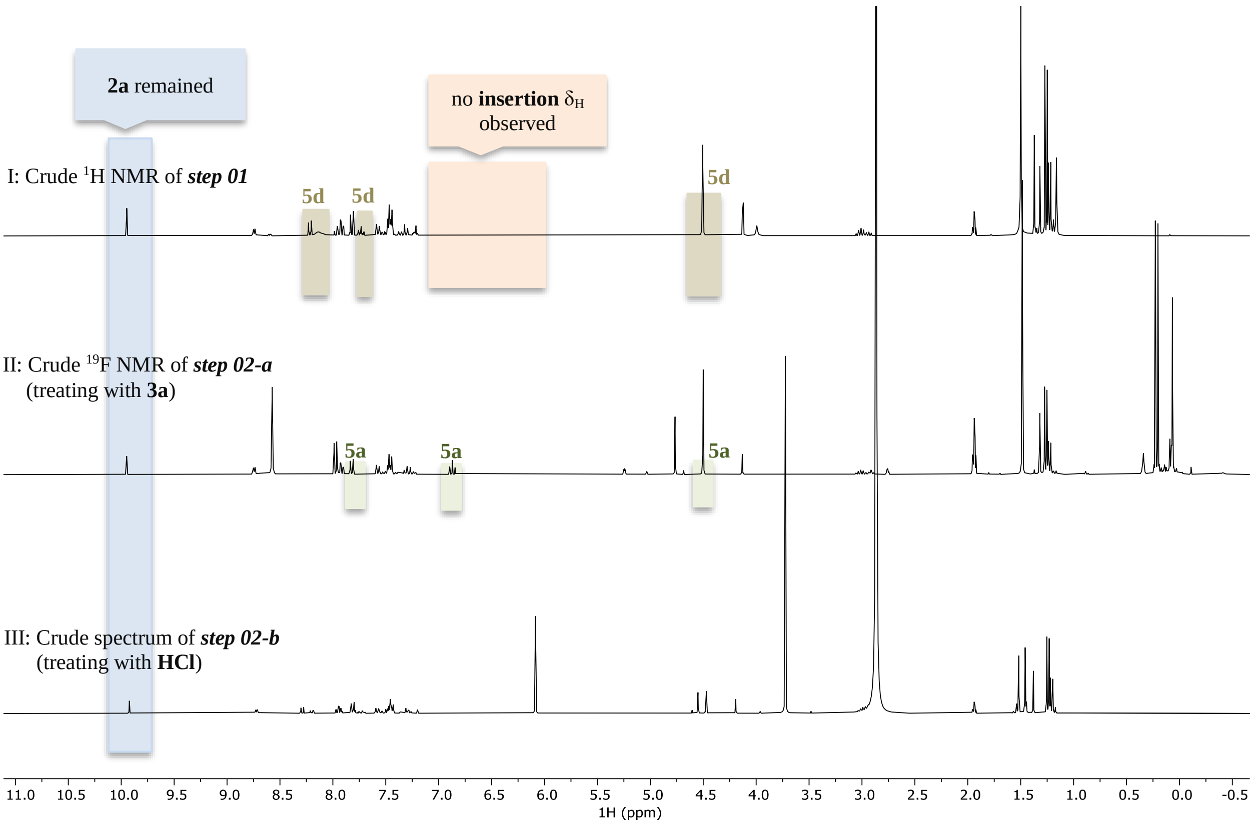

Figure S21. ^{1}H NMR spectra of reactions of **1a**, **2a** and **5a** (300 MHz, MeCN-d_3, 298K).

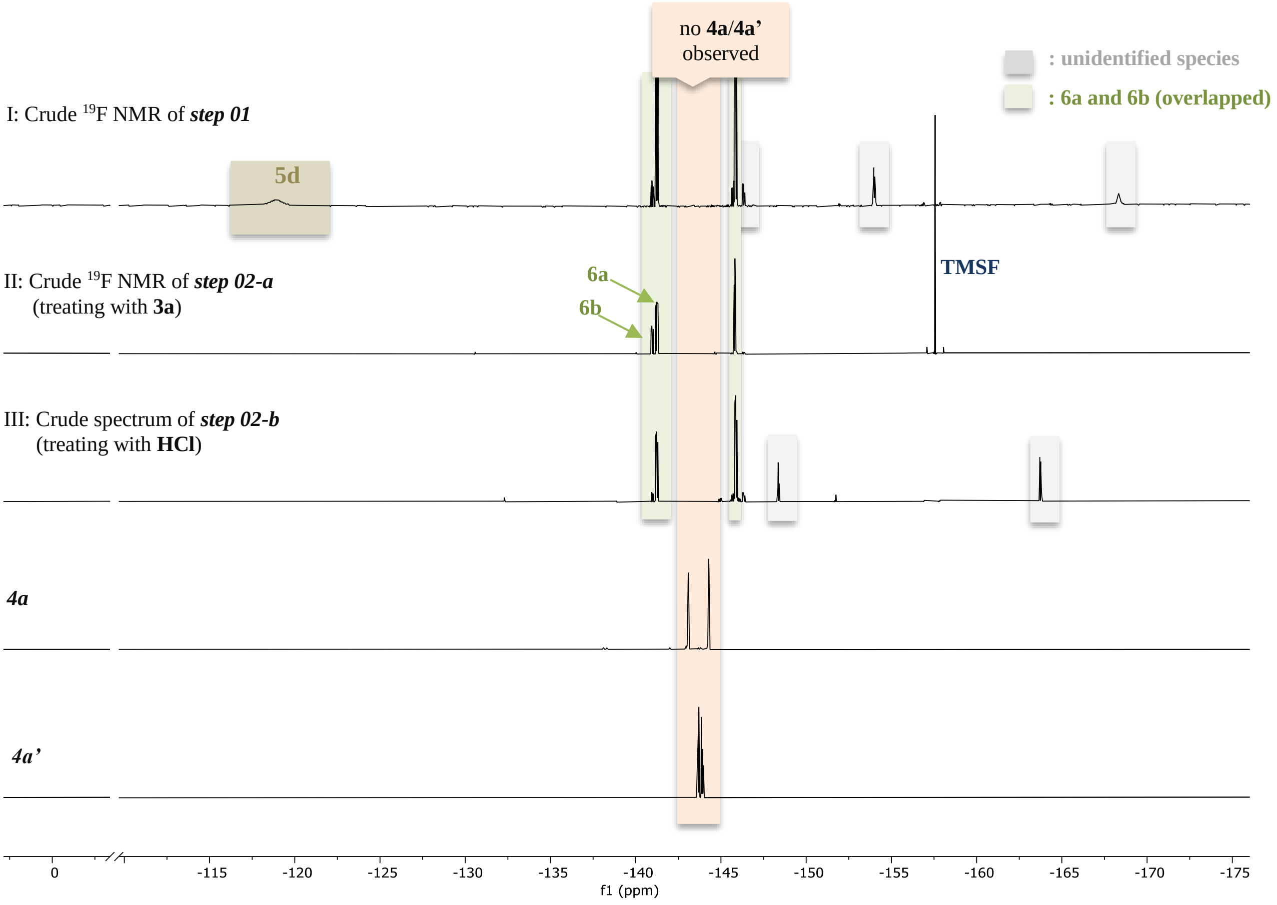

Figure S22. ^{19}F NMR spectra of reactions of **1a**, **2a** and **5a** (282 MHz, MeCN-d_3, 298K).

3.5.2 Kinetic experiment of 1a, 5a and 3a

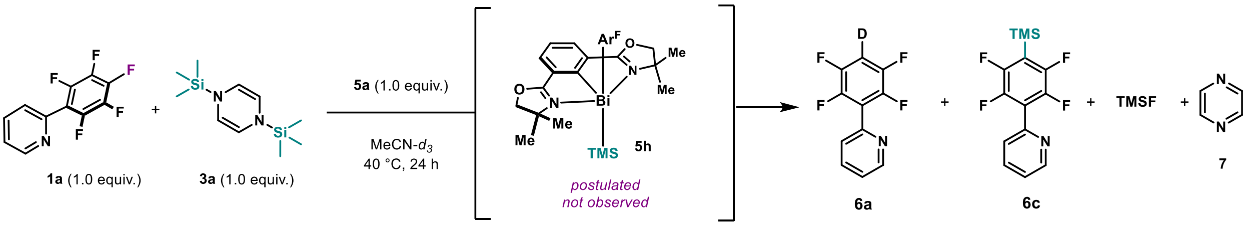

Procedure: In an argon-filled glovebox, a J-young NMR tube was charged with **1a** (6.1 mg, 25 µmol), **5a** (12 mg, 25 µmol) and MeCN-d_3 (0.50 mL). After adding **3a** (5.7 mg, 25 µmol), the tube was quickly capped and removed from the glovebox, then kept at 0 °C and quickly shaken before recording the NMR spectra at 40 °C.

Based on the NMR data (*vide infra*), it was concluded that the deutero-defluorination product **6a** and TMS-Ar^F **6c** was formed; Bi(I) **5a** was the major Bi-based species and its concentration remained relatively stable during the reaction. It is proposed that **6c** was formed through the C–Si reductive elimination *via* the putative intermediate Bi(III)(Ar^F)TMS **5h**, which is generated after ligand metathesis from **5c**.

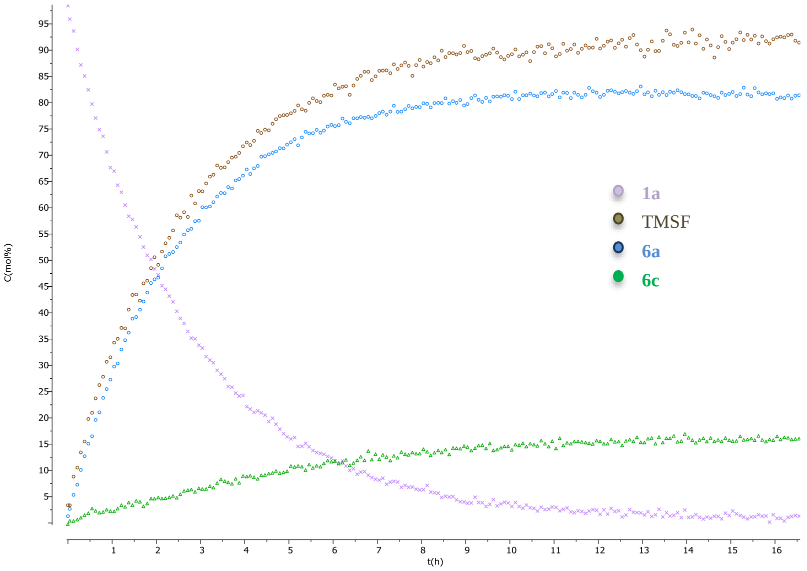

Figure S23. ^{19}F NMR kinetic plot of reaction of **1a**, **3a** and **5a** in MeCN-d_3.

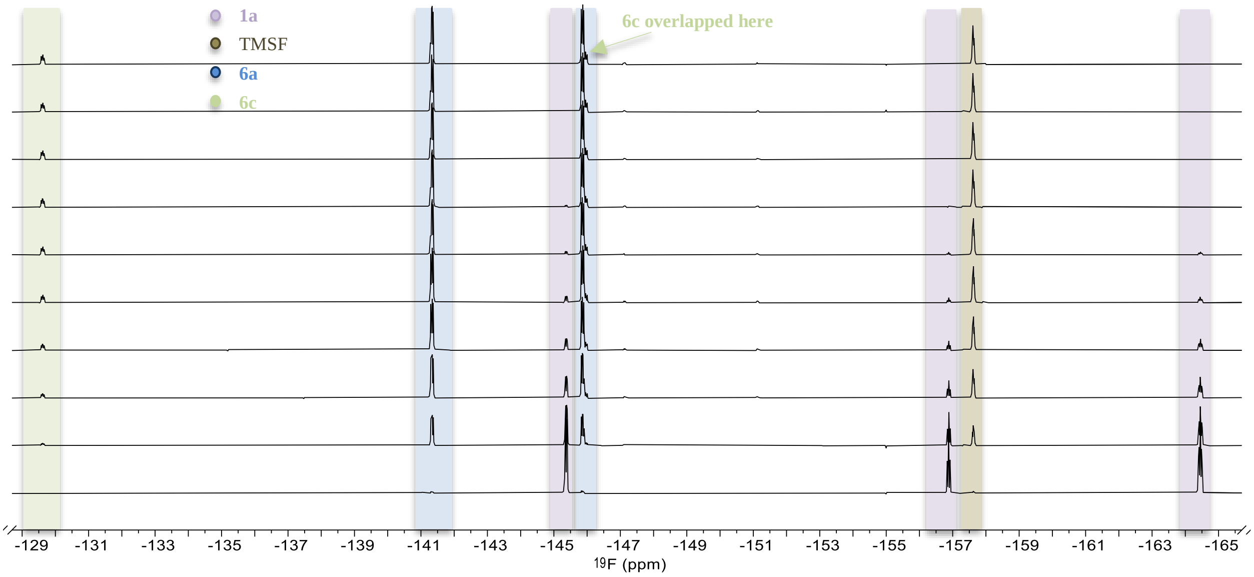

Figure S24. ^{19}F NMR spectra at different time points during the kinetic reaction of **1a**, **3a** and **5a** (600 MHz, MeCN-d_3, 313K).

In this experiment, **1a** decayed faster than the stoichiometric reaction of **1a** and **5a** in the absence of **3a**. (Compared to plot of **1a**

in Figure S2). We believe that the postulated OA intermediate **5c** reacted with **3a** to form the Bi(III)(Ar^F)TMS **5h** and release **6c**, which accelerated the reaction by pushing the equilibrium among OA step towards the C–F bond cleavage.

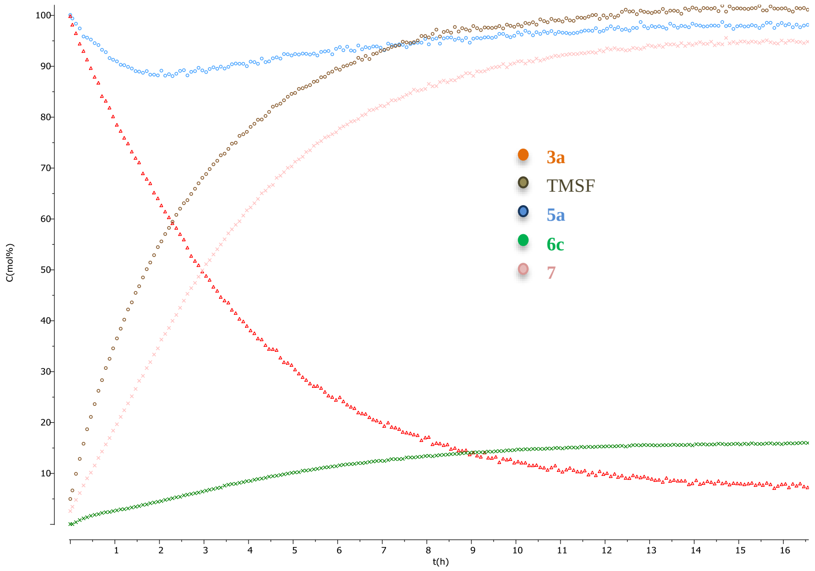

Figure S25. ^{1}H NMR kinetic profile of reaction of **1a**, **3a** and **5a** in MeCN-d_3.

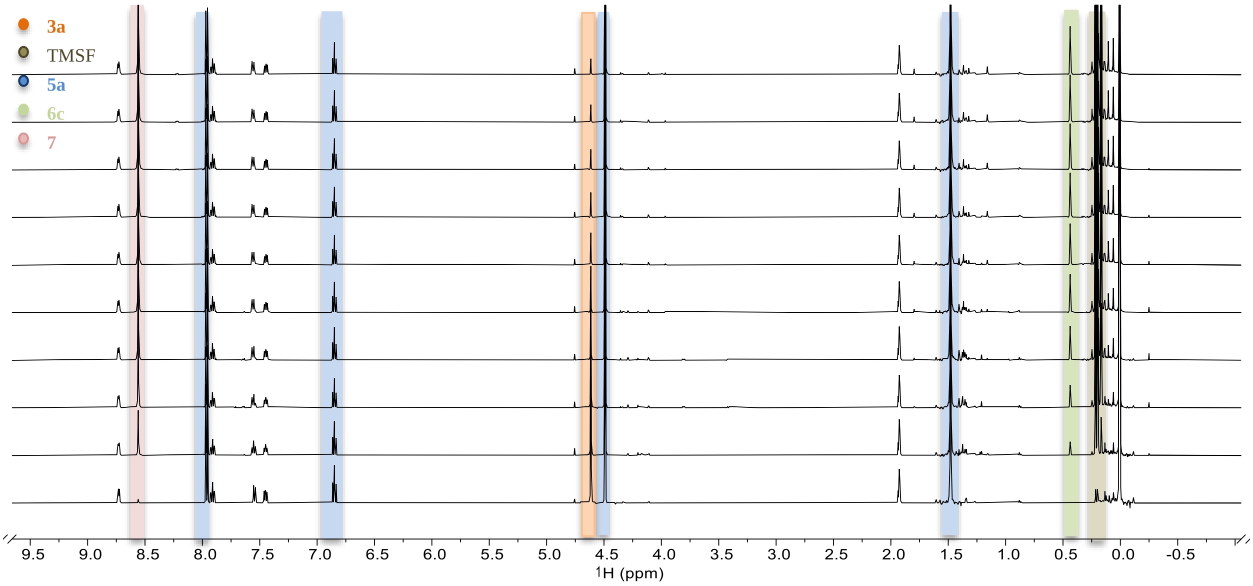

Figure S26. ^{1}H NMR spectra at different time points during the kinetic reaction of **1a**, **3a** and **5a** (600 MHz, MeCN-d_3, 313K).

The ^{1}H NMR kinetic profile shows that the concentration of **5a** dropped slightly at the beginning and then remained constant, which probably attributes to the formation of some unidentified active intermediate.

3.5.3 Characterization data of compound 6c

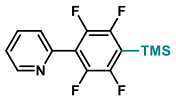

^{1}H NMR (600 MHz, $CDCl_3$) δ 8.78 (ddd, J = 4.9, 1.8, 1.0 Hz, 1H), 7.83 (td, J = 7.7, 1.8 Hz, 1H), 7.50 (dt, J = 7.8, 1.2 Hz, 1H), 7.36 (ddd, J = 7.7, 4.8, 1.2 Hz, 1H), 0.44 (t, J = 1.4 Hz, 9H).

^{19}F NMR (565 MHz, $CDCl_3$) δ -128.13 (dd, J = 24.2, 15.1 Hz), -144.40 (dd, J = 24.0, 14.9 Hz).

^{29}Si NMR (119 MHz, $CDCl_3$) δ -1.26 (tt, J = 3.2, 2.1 Hz).

^{13}C NMR (151 MHz, $CDCl_3$) δ 150.2, 150.1 – 148.4 (m), 148.4 – 148.3 (m), 143.9 (ddt, J = 251.8, 18.9, 3.7 Hz), 136.7, 126.0 (t, J = 1.9 Hz), 123.7, 121.4 – 121.1 (m), 117.4 (tt, J = 32.7, 2.3 Hz), 0.1 (t, J = 2.9 Hz).

HRMS (ESI-TOF): calc'd for $C_{14}H_{14}F_4N_1Si_1$ $[M+H]^+$: 300.0826, found: 300.0825.

3.5.4 Control experiments of 6c and 2a

Based on the previous report of catalyst-free nucleophilic fluoroalkylation of aldehydes,[9] we evaluated the reactivity between **2a** and TMS−ArF **6c** either with or without fluorine anion reagent as below.

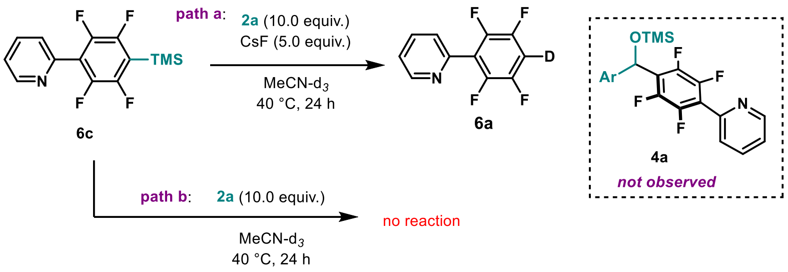

NMR data indicates that **6c** remained unreactive towards excess of **2a** and no arylation product **4a** was observed with or without CsF, which ruled out the side-pathways between **6c** and **2a**, suggesting the necessity of Bi-based active intermediates.

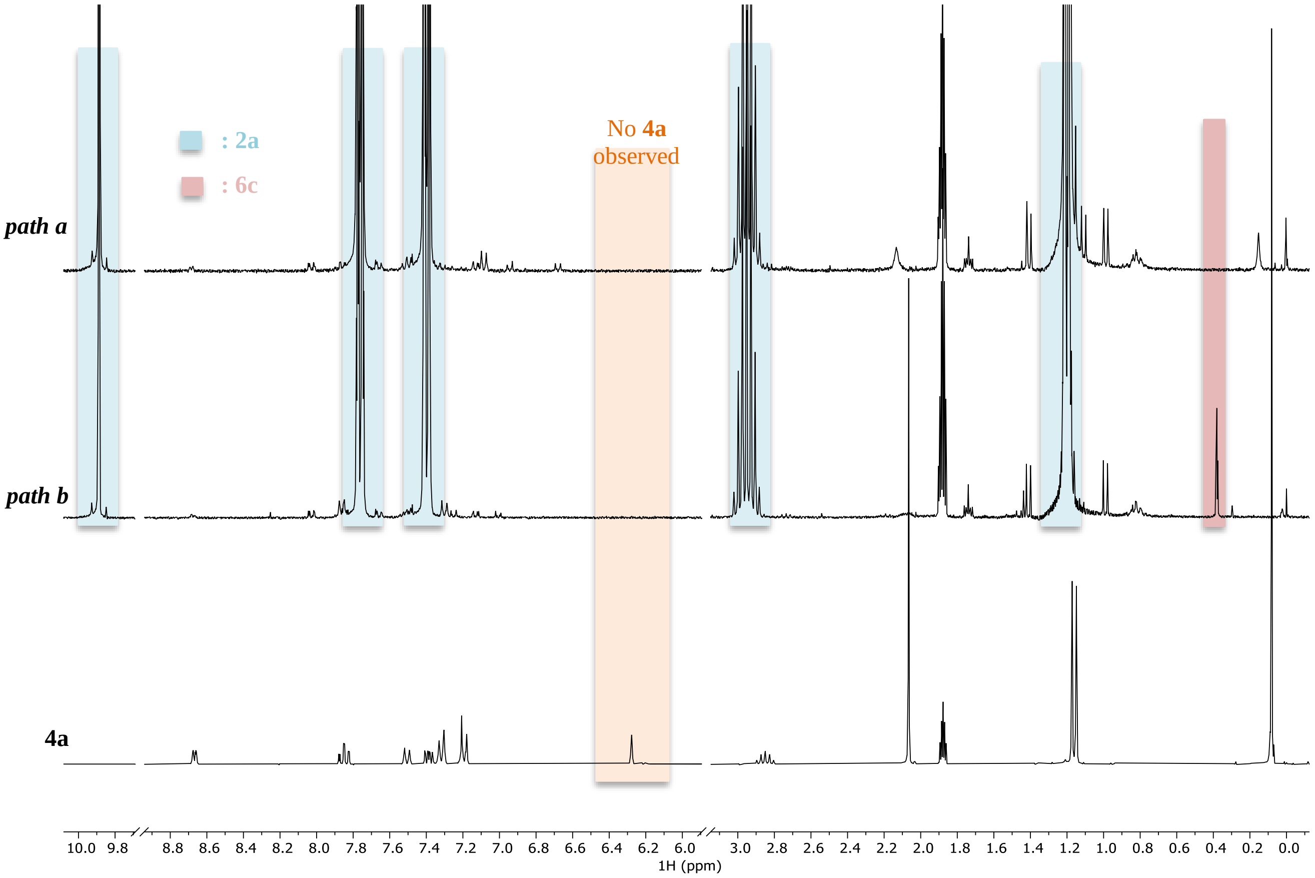

Figure S27. ^{1}H NMR spectra of reaction of **6c** and **2a** with or *w/o* CsF (300 MHz, MeCN-d_3, 298K).

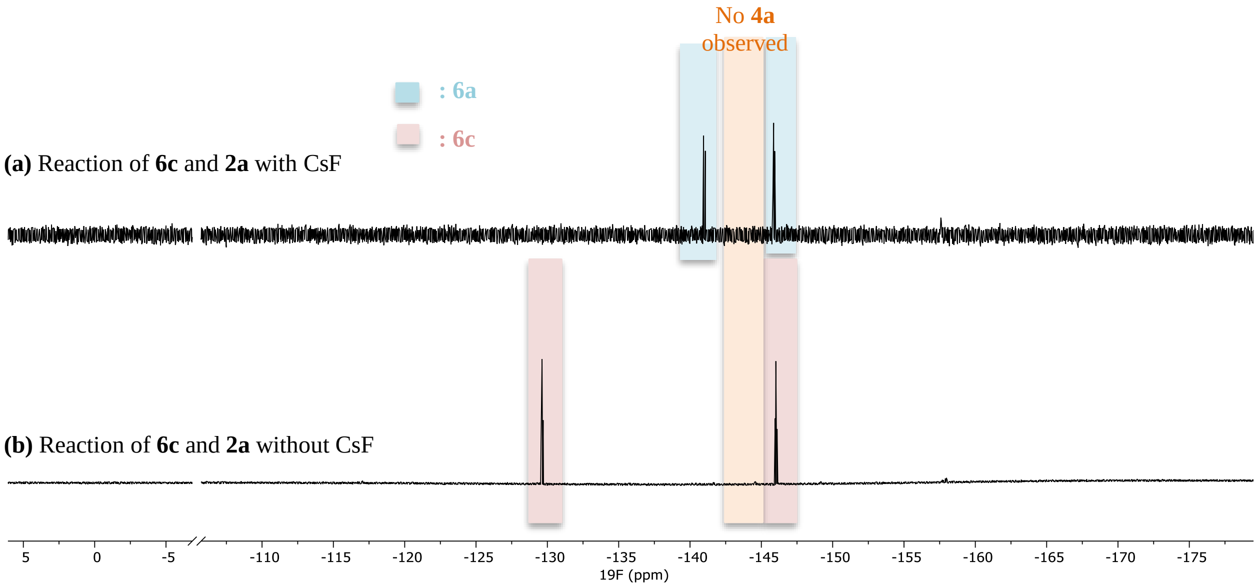

Figure S28. ^{19}F NMR spectra of reaction of **6c** and **2a** with or *w/o* CsF (282 MHz, MeCN-d_3, 298K).

3.5.5 Stoichiometric experiments of 5d and 3a

To probe the possibility of F/TMS interaction, we independently prepared PheBox-BiF_2 **5d**[1] and subject it to a stoichiometric amount of TMS–DHP **3a** in MeCN-d_3. Within 5 min at room temperature, the formation of Bi(I) and TMSF were observed, indicating the strong ability of TMS–DHP to cleave a Bi–F bond and supporting the feasibility of F/TMS interaction during this process with **5h**. Other than this, similar Bi–X bonds cleavage (X = I, Br) by Mashima's reagent has been reported to proceed efficiently,[10] further supporting the viability of this transformation and its potential to facilitate subsequent reactivity based on this process.

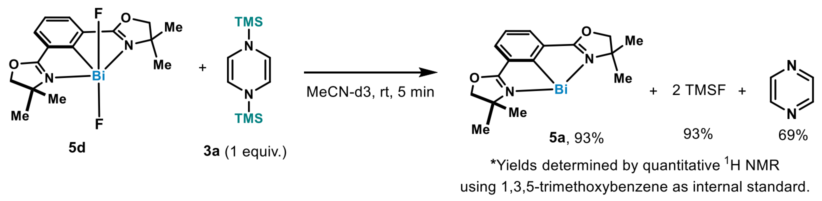

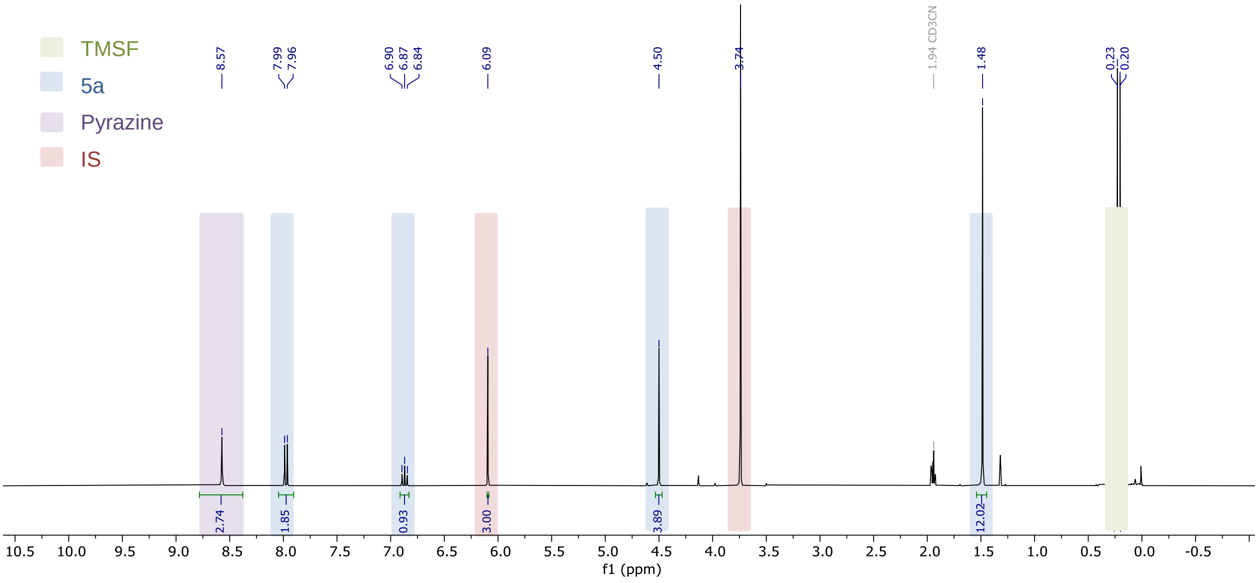

Figure S29. ^{1}H NMR spectra of reaction of **5d** and **3a** (300 MHz, MeCN-d_3, 298K).

3.6 Reductive elimination of Bi(III) with 3a

3.6.1 Synthesis of PheBox-Bi(III)-oxides

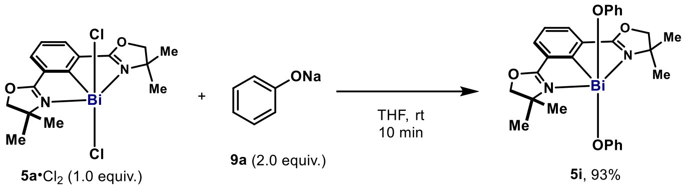

In an Argon-filled glovebox, a 20 mL vial was charged with **5a**•Cl_2 (110 mg, 0.20 mmol), sodium phenoxide (47 mg, 0.40 mmol, 2.0 equiv), and THF (5.0 mL). The mixture was stirred for 10 min at 25 °C and filtered through celite. The filtrate was evaporated under vacuum to afford yellow solid **5i** (124 mg, 93% yield).

^{1}H NMR (400 MHz, THF-d_8) δ 8.08 (d, *J* = 7.6 Hz, 2H), 7.56 (t, *J* = 7.7 Hz, 1H), 6.80 (t, *J* = 7.5 Hz, 4H), 6.23 – 6.13 (m, 6H), 4.47 (s, 4H), 1.48 (s, 12H).

^{13}C NMR (101 MHz, THF-d_8) δ 212.3, 181.4, 166.9, 134.2, 132.6, 128.94, 128.86, 120.7, 115.3, 83.4, 68.3, 28.7.

HRMS (ESIpos, m/z): calculated for $C_{22}H_{24}BiN_2O_3$ $[M–C_6H_5O]^+$ 573.1585; found 573.1593.

Anal. Calc. for $C_{28}H_{29}BiN_2O_4$: C, 50.46; H, 4.39; N, 4.20; Found: C, 50.21; H, 4.42; N, 4.18.

Suitable single crystals for X-ray diffraction were obtained by vapor diffusion of pentane into a saturated THF solution of **5i** at -35 °C overnight (*ca.* 12 h) in the glovebox.

3.6.2 Reductive O–Si bond formation from PheBox-Bi(III) alkoxides

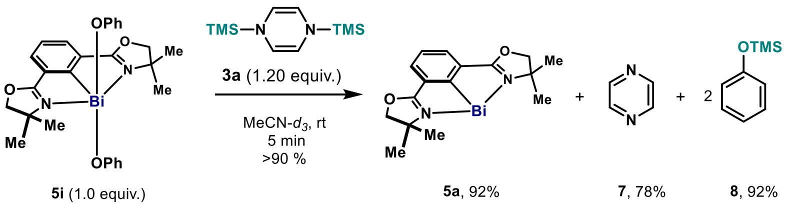

In an Argon-filled glovebox, an NMR tube was charged with TMS-DHP **3a** (14 mg, 0.06 mmol) and Bi(III) complex **5i** (33 mg, 0.05 mmol) and MeCN-d_3 (0.50 mL). A septum cap was fitted and the tube was shaked for 5 min. Then 1,3,5-trimethoxybenzene (0.05 mmol) was added and then the mixture was submitted to quantitive ^{1}H NMR for determining the yield.

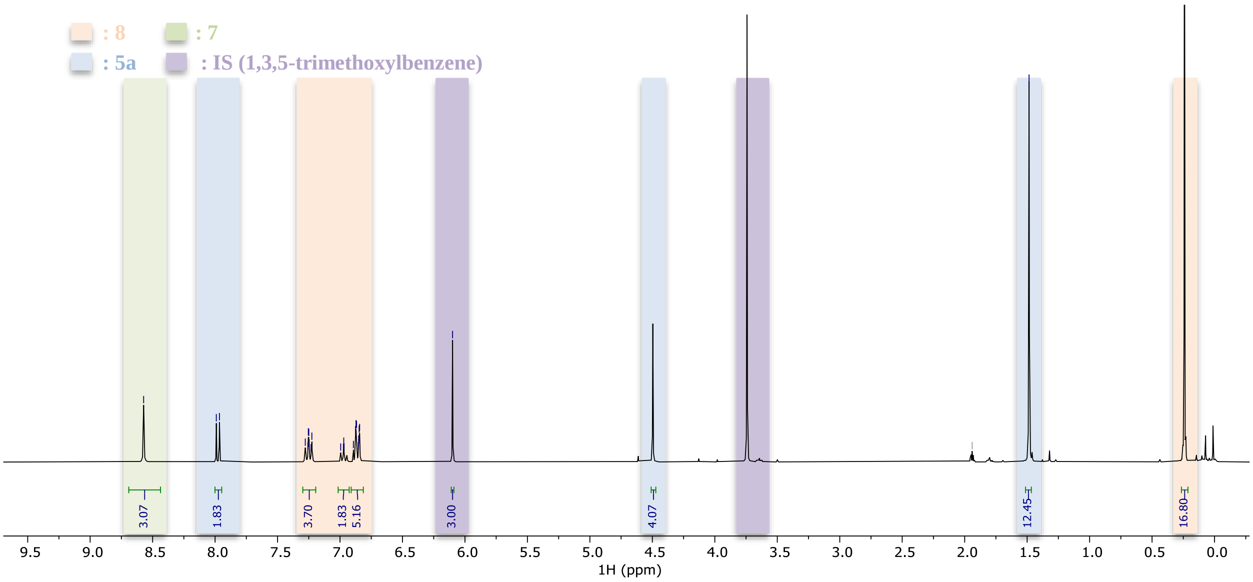

Figure S30. Crude quantitative ^{1}H NMR spectra of the crude mixture of **3a** and **5i** (300 MHz, MeCN-d_3, 298K).

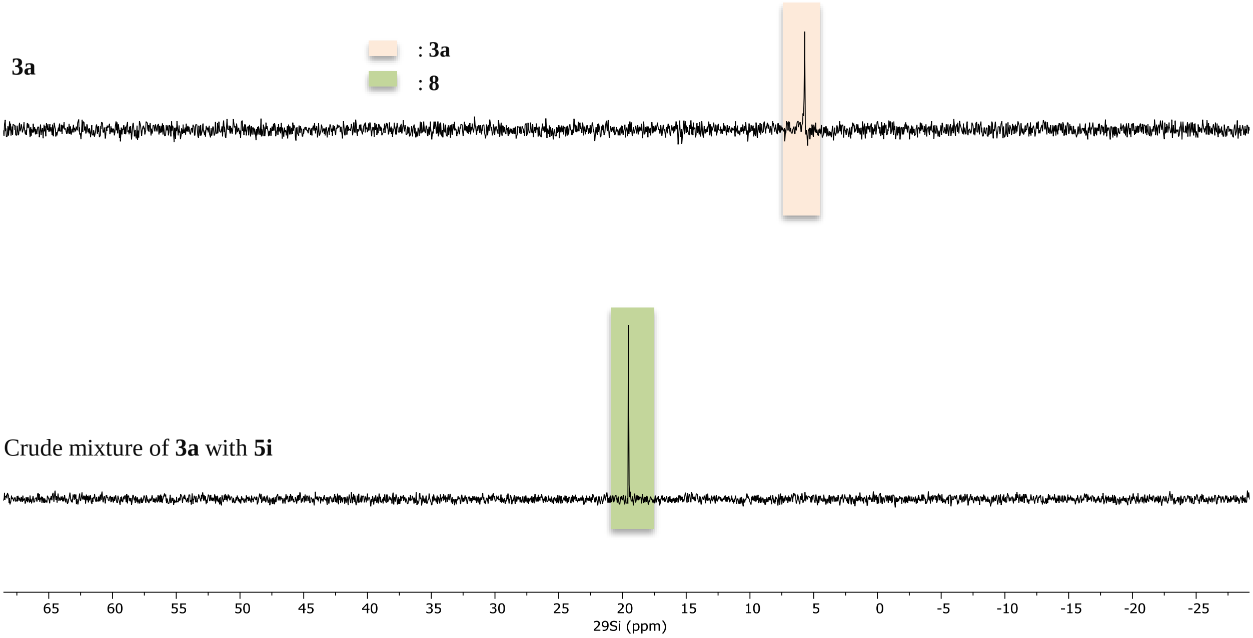

Figure S31. ^{29}Si NMR spectra of **3a** (*upper*) and the crude mixture acquired after the reaction of **3a** and **5i** (*bottom*) (60 MHz, MeCN-d_3, 298K).

3.7 Kinetic experiments of the catalytic arylation reaction

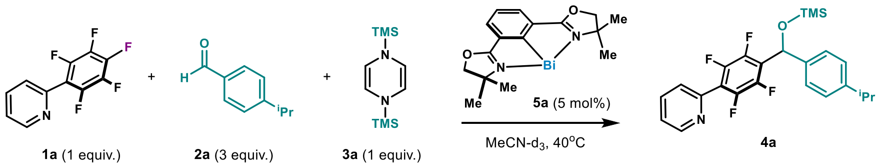

<u>*Procedure*</u>*:* In an argon-filled glovebox, a J-young NMR tube was charged with **1a** (25 mg, 0.10 mmol), **2a** (45 mg, 0.3 mmol), **3a** (23 mg, 0.10 mmol), **5a** (12 mg, 5.0 µmol) and MeCN-d_3 (0.50 mL). The tube was sealed with a Teflon screw cap, removed from the glovebox, then kept at −40 °C and quickly shaken before inserting it into the NMR probe which was prewarmed to 40 °C. After shimming, the NMR spectra were immediately recorded.

Note: The NMR-minitored reaction under static (non-stirred) conditions furnished a yield comparable to that of the standard stirred reaction, albeit with prolonged reaction time.

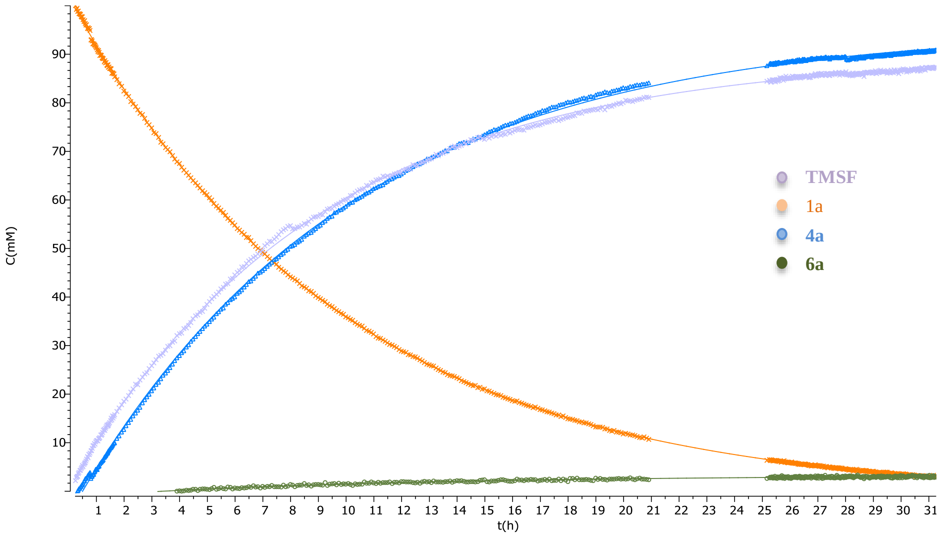

Figure S32. ^{19}F NMR kinetic profile of the catalytic reaction at 40 °C.

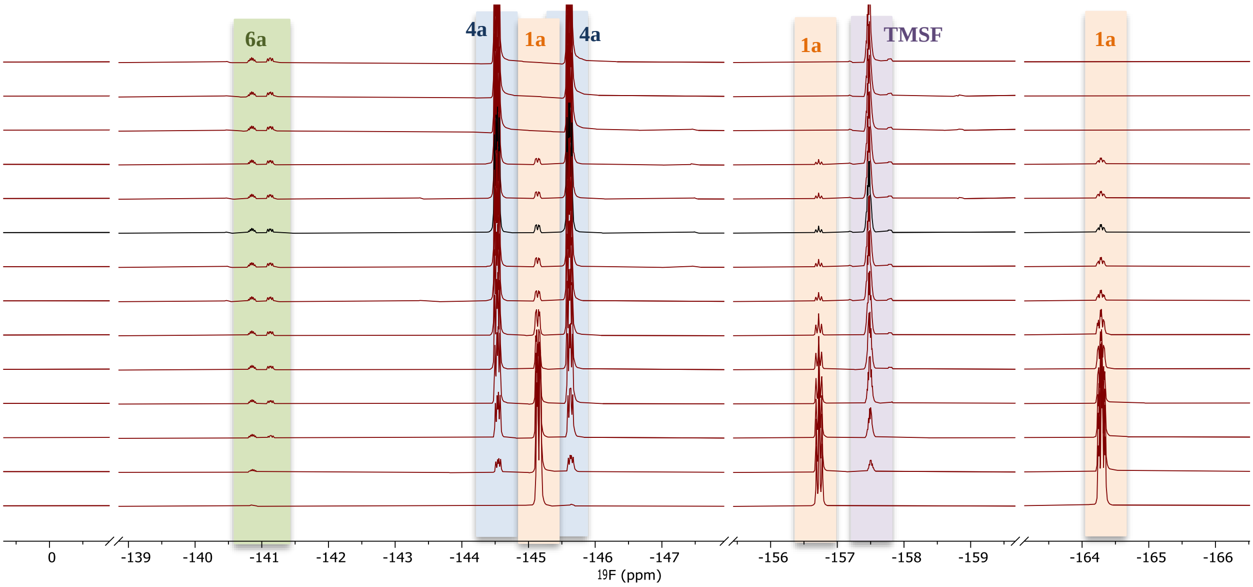

Figure S33. ^{19}F NMR spectra at different time points (600 MHz, MeCN-d_3, 313K).

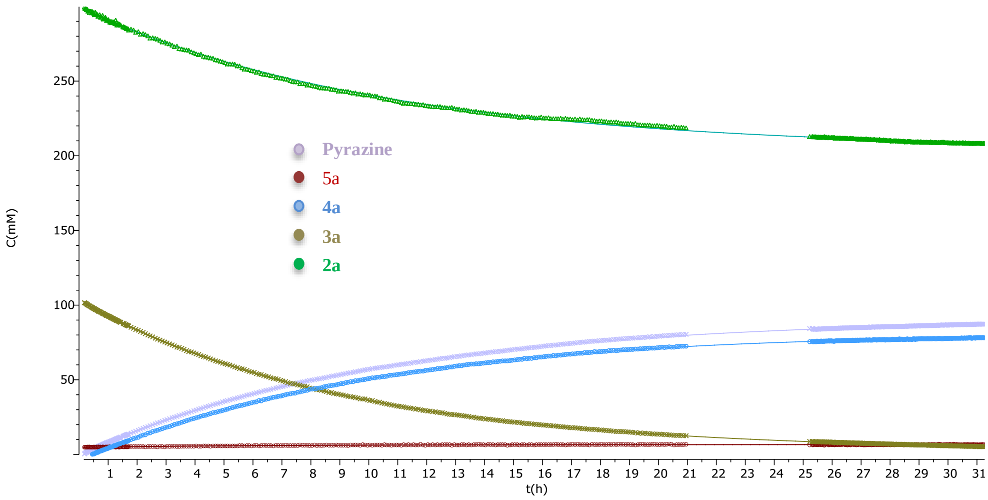

Figure S34. ^{1}H NMR kinetic plot of the catalytic model reaction at 40 °C

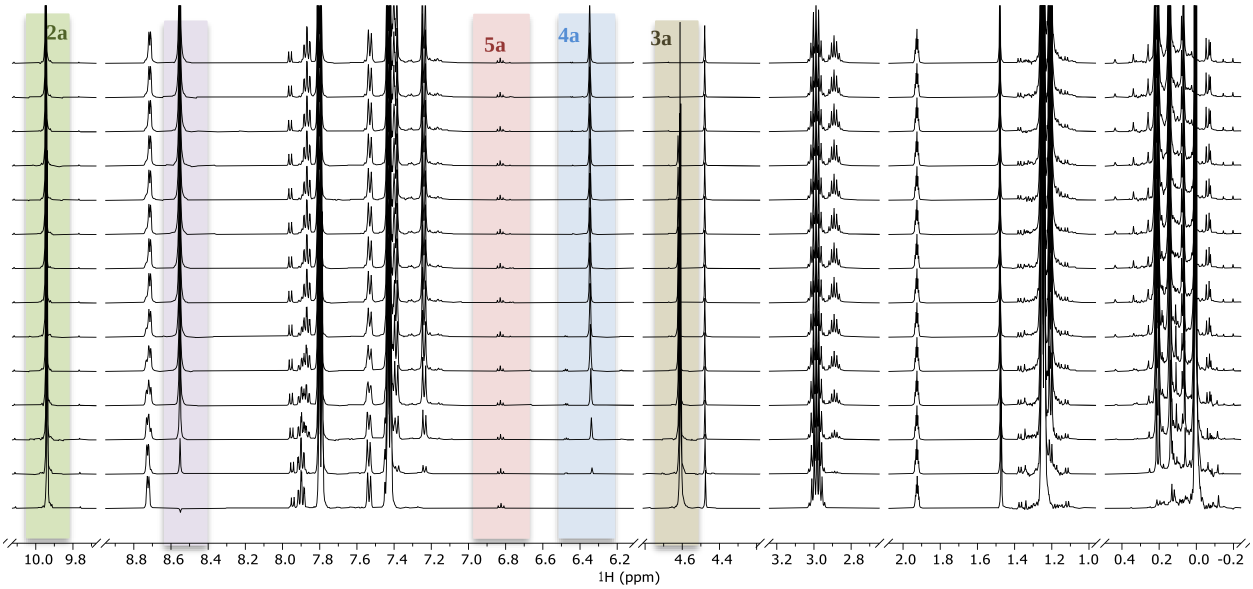

Figure S35. ^{1}H NMR spectra at different time points (600 MHz, MeCN-d_3, 313 K).

It is important to emphasize the fact that Bi(I) complex **5a** is observed as the resting state throughout the entire catalytic profile, suggesting the proposed mechanism in which the oxidative addition (OA) step is the turnover-limiting and most energetically demanding step of the reaction.

4. Computational details and discussion

4.1 General procedure

All quantum chemical calculations were carried out using the ORCA 5.0.1 program[11] Geometries of intermediates and transition states were optimized using the PBE0 density functional[12] with the D3BJ[13] dispersion correction and the Def2-SVP[14] basis set and in acetonitrile solvent with the CPCM solvation model.[15] The relativistic effective core potential of Bi was represented using Def2-ECP (Bi).[16] Vibrational frequency calculations were performed for all stationary points to confirm if each optimized structure is a local minimum or a transition state structure. All optimized transition state structures have only one imaginary (negative) frequency, and all minima (reactants, products, and intermediates) have no imaginary frequencies. For transition state structures, intrinsic reaction coordinate (IRC) calculations were carried out to ensure transit from reactants to products. The PBE0 functional with the D3BJ correction, the Def2-TZVP basis set, and Def2-ECP (Bi) was used for single-point energy calculations. The reported Gibbs free energies were calculated at the standard conditions (298 K, 1 atm). Cartesian coordinates for stationary points and the absolute thermochemical data are given in Sect. 8.

4.2 Insertion by 5c isomers

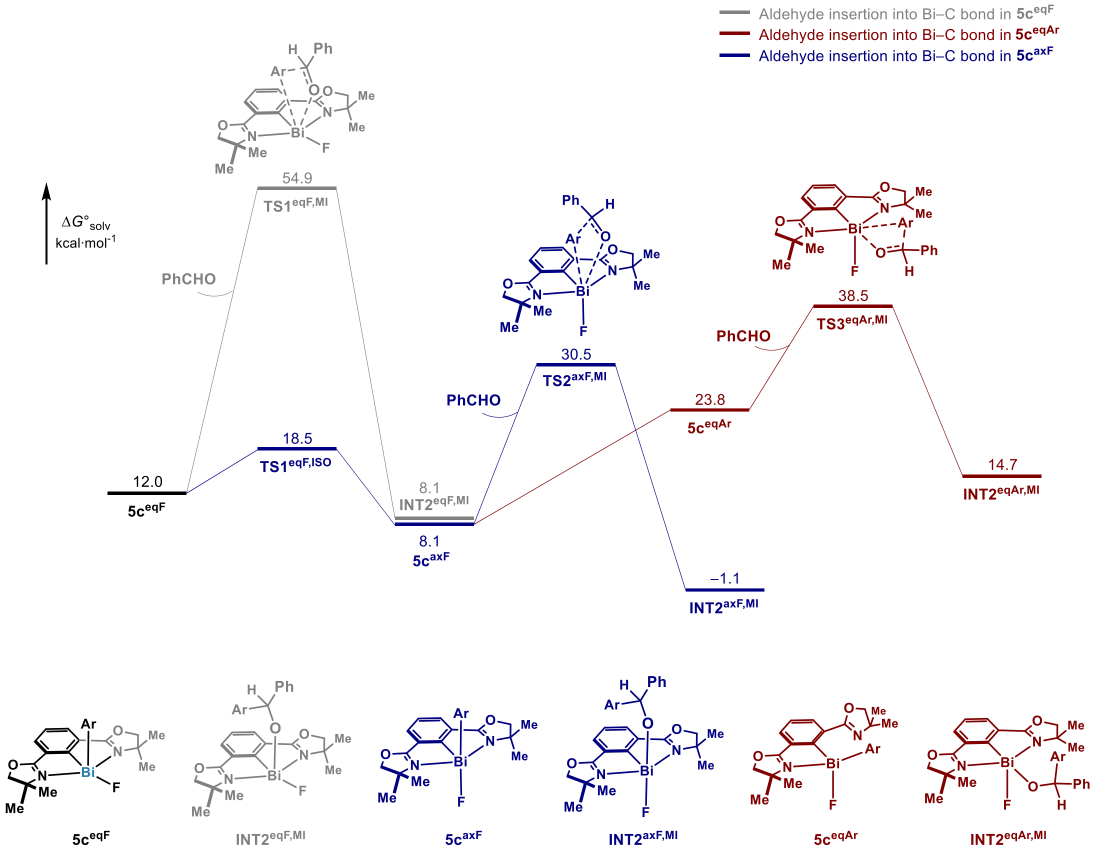

Potential insertion pathways via **5c** isomers (**5c**$^{\mathbf{eqF}}$, **5c**$^{\mathbf{axF}}$, and **5c**$^{\mathbf{eqAr}}$) were considered. Direct insertion of benzaldehyde by isomer **5c**$^{\mathbf{eqF}}$ was found to be unlikely due to its high kinetic barrier (**TS1**$^{\mathbf{eqF,MI}}$, $\Delta G^{\ddagger}$ = 42.9 kcal/mol). Isomer **5c**$^{\mathbf{axF}}$ was found to be the most stable isomer that can be accessed from **5c**$^{\mathbf{eqF}}$ with a low barrier for isomerization (**TS1**$^{\mathbf{eqF,ISO}}$, $\Delta G^{\ddagger}$ = 6.5 kcal/mol). Insertion of benzaldehyde by **5c**$^{\mathbf{axF}}$ is predicted to be exergonic (formation of **INT2**$^{\mathbf{axF,MI}}$, ΔG = –9.2 kcal/mol) with a kinetic barrier (**TS2**$^{\mathbf{ax}}$) of 22.4 kcal/mol. However, compared to the insertion by **5h** (**TS1**$^{\mathbf{BiC}}$, $\Delta G^{\ddagger}$ = 15.1 kcal/mol), that by **5c**$^{\mathbf{axF}}$ is kinetically less accessible. The other isomer **5c**$^{\mathbf{eqAr}}$ was found to be the least stable isomer with the insertion transition state **TS3**$^{\mathbf{eqAr,MI}}$ located at ΔG_{rel} = 38.5 kcal/mol. The overall process via **5c**$^{\mathbf{eqAr}}$ displays a relatively large energy span between **5c**$^{\mathbf{axF}}$ and **TS3**$^{\mathbf{eqAr,MI}}$ (30.4 kcal/mol), suggesting that insertion via **5c**$^{\mathbf{eqAr}}$ would be less favorable compared to that via **5c**$^{\mathbf{axF}}$. These results suggest that insertion by **5c** isomers would be less favorable compared to the proposed process by **5h**.

4.3 5h isomer (5h^{ax} with axial Ar)

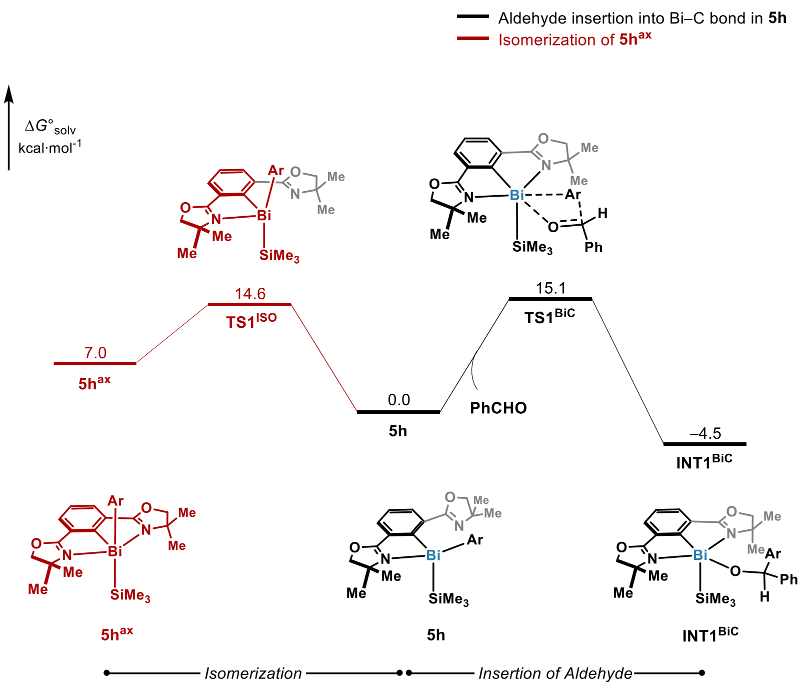

Compound **5h** was found to be a stable isomer of **5a**·[Ar][$SiMe_3$] (Ar = 2,3,5,6-tetrafluoro-4-(2-pyridyl)-phenyl). The isomer of **5h**, compound **5h^{ax}** (with axial Ar), was also examined. The isomerization of **5h** into **5h^{ax}** is found to be endergonic (ΔG = +7.0 kcal/mol). Given the low electronegativity of Si atom, 3-centered-4-electron bonding (C–Bi–Si) would have a smaller stabilization effect compared to that in **5c^{axF}** (C–Bi–F). This accounts for the computed thermodynamic preference of compound **5h** to **5h^{ax}**. The transition state for the isomerization (**TS1^{ISO}**) is located at ΔG_{rel} = +14.6 kcal/mol, suggesting that the isomerization of **5h^{ax}** to **5h** would be rapid under the standard reaction conditions. Given the relative stability of **5h**, the low kinetic barrier of isomerization (**TS1^{ISO}**) and subsequent insertion (**TS1^{BiC}**), the overall computational results suggest that insertion of benzaldehyde by **5h** is highly feasible.

Note: A concerted pathway via 5-membered ring (**5h** + PhCHO → **5a** + **4z**) was considered, but no corresponding saddle point was found. Instead, potential coordinates for the concerted TS were converged to **TS2** by TS optimization or were not converged.

5. References

1. Y. Pang, M. Leutzsch, N. Nöthling, F. Katzenburg, J. Cornella, *J. Am. Chem. Soc.* **2021**, *143*, 12487–12493.
2. F. Wang, O. Planas, J. Cornella, *J. Am. Chem. Soc.* **2019**, *141*, 4235–4240.
3. M. M. Parsutkar, C. E. Moore, T. V. RajanBabu, *Dalton Trans.* **2022,** *51*, 10148–10159.
4. H.-Q. Do, O. Daugulis, *J. Am. Chem. Soc.* **2008,** *130*, 1128–1129.
5. A. F. Palermo, B. S. Y. Chiu, P. Patel, S. A. L. Rousseaux, *J. Am. Chem. Soc.* **2023,** *145*, 24981–24989.
6. D. Zhao, J. A. Krause, W. B. Connick, *Inorg. Chem.* **2015,** *54*, 8339–8347.
7. a) S. Ni, D. Spinnato, J. Cornella, *J. Am. Chem. Soc.* **2024**, *146*, 22140–22144; b) M. Mato, P. C. Bruzzese, F. Takahashi, M. Leutzsch, E. J. Reijerse, A. Schnegg, J. Cornella, *J. Am. Chem. Soc.* **2023**, *145*, 18742–18747; c) M. Mato, D. Spinnato, M. Leutzsch, H. W. Moon, E. J. Reijerse, J. Cornella, *Nat. Chem.* **2023**, *15*, 1138–1145.
8. X. Yang, E. J. Reijerse, K. Bhattacharyya, M. Leutzsch, M. Kochius, N. Nöthling, J. Busch, A. Schnegg, A. A. Auer, J. Cornella, *J. Am. Chem. Soc.* **2022,** *144*, 16535–16544.
9. K. Komoda, A. Shimokawa, H. Amii, *ChemistrySelect.* **2019**, *4*, 2374–2378.
10. a) Z. Turner, *Inorg. Chem.* **2019**, *58*, 14212–14227; b) P. K. Majhi, H. Ikeda, T. Sasamori, H. Tsurugi, K. Mashima, N. Tokitoh, *Organometallics*. **2017**, *36*, 1224-1226.
11. a) F. Neese, *Wiley Interdiscip. Rev.: Comput. Mol. Sci.* **2012**, *2*, 73–78; b) F. Neese, *Wiley Interdiscip. Rev.: Comput. Mol. Sci.* **2022**, *12*, e1606.
12. a) J. Perdew, M. Ernzerhof, K. Burke, *J. Chem. Phys.* **1996**, *105*, 9982–9985; b) C. Adamo, V. Barone, *J. Chem. Phys.* **1999**, *110*, 6158–6170.
13. a) S. Grimme, S. Ehrlich, L. Goerigk, *J. Comput. Chem.* **2011**, *32*, 1456–1465; b) S. Grimme, J. Antony, S. Ehrlich, H. Krieg, *J. Chem. Phys.* **2010**, *132*, 154104.
14. F. Weigend, R. Ahlrichs, *Phys. Chem. Chem. Phys.* **2005**, *7*, 3297–3305.
15. a) V. Barone, M. Cossi, *J. Phys. Chem. A.* **1998**, *102*, 1995–2001; b) M. Garcia-Ratés, F. Neese, *J. Comput. Chem.* **2020**, *41*, 922–939.
16. a) W. Küchle, M. Dolg, H. Stoll, H. Preuss, *Mol. Phys.* **1991**, *74*, 1245–1263; b) ECP parameters for Bi [SD(78,MWB)] have been obtained from: pseudopotential library of the Stuttgart/Cologne group http://www.theochem.uni-stuttgart.de/pseudopotentials/

6. Crystallographic data

Single crystal structure analysis of complex 4g’

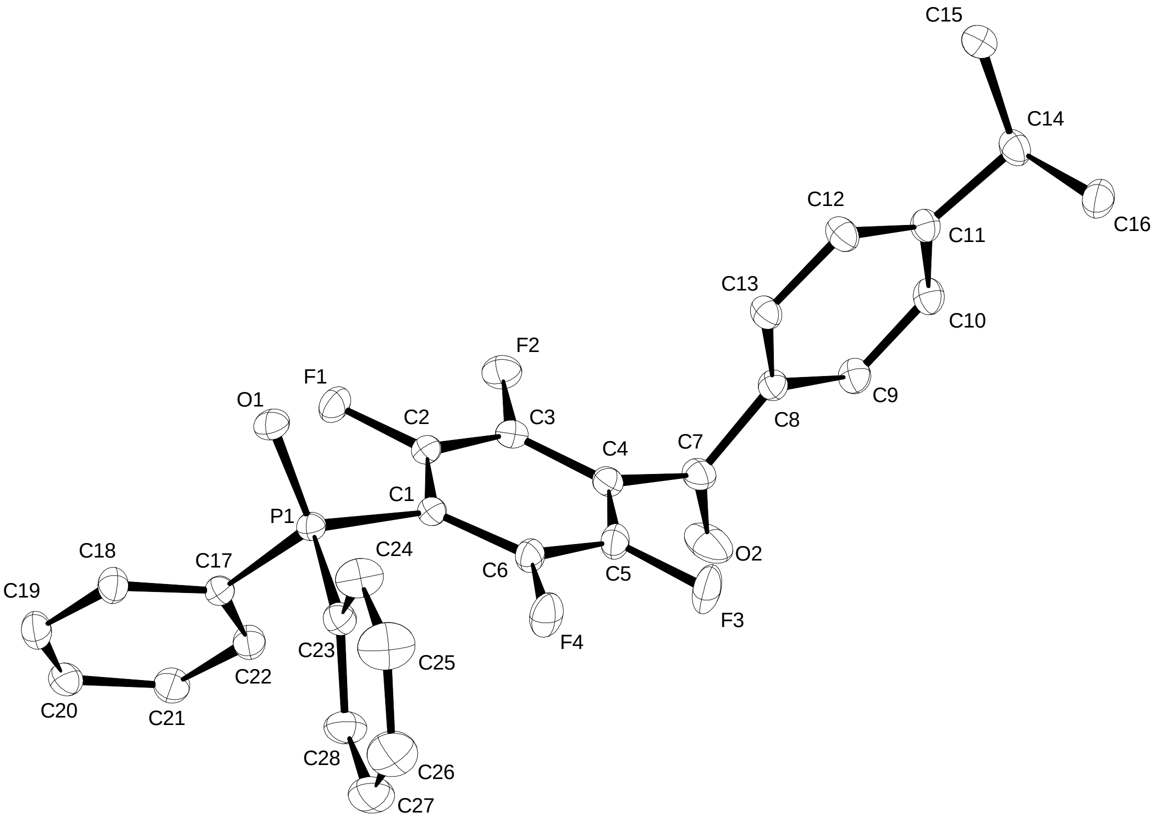

Figure S36. The solid state structure of **4g’**. H atoms have been removed for clarity.

X-ray Crystal Structure Analysis of complex **4g’**:

$C_{28}H_{21}F_4O_2P$, M_r = 496.42 g mol^{-1}, clear colourless plate, crystal size 0.361 x 0.204 x 0.120 mm^3, monoclinic, space group $P2_1/n$ [14], a = 19.7149(12) Å, b = 5.7656(3) Å, c = 21.4357(13) Å, β= 103.491(2)°, V = 2369.3(2) Å^3,T = 100(2) K, Z = 4, D_{calc} = 1.392 g·cm^3, λ = 0.71073 Å, $\mu(Mo\text{-}K_\alpha)$ = 0.172 mm^{-1}, Gaussian correction (T_{min} = 0.9334, T_{max} = 1.0000), Bruker-AXS D8 Venture with Photon III detector and IµS Diamond microfocus Mo-anode X-ray source, 3.188 < θ < 38.568°. 192509 measured reflections, 13235 independent reflections, 11898 reflections with $I > 2\sigma(I)$, R_{int} = 0.0329. The structure was solved by *SHELXT* and refined by full-matrix least-squares (*SHELXL*) against F^2 to R_1 = 0.0353 [$I > 2\sigma(I)$], wR_2 = 0.0353 [all data] with 318 parameters and 0 restraints.

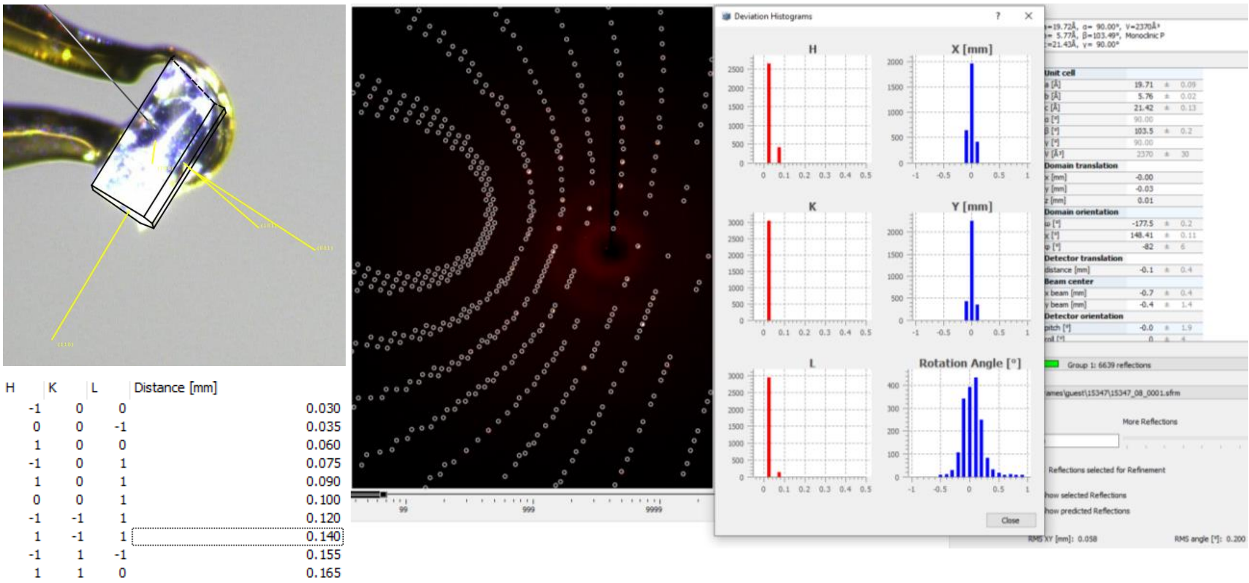

Figure S37. Crystal faces and unit cell determination/refinement of **4g'**.

INTENSITY STATISTICS FOR DATASET

Resolution	#Data	#Theory	%Complete	Redundancy	Mean I	Mean I/s	Rmerge	Rsigma
Inf - 2.14	228	326	69.9	15.65	48.42	83.94	0.0248	0.0132
2.14 - 1.50	525	551	95.3	22.34	63.13	103.40	0.0253	0.0111
1.50 - 1.21	744	747	99.6	19.95	56.58	94.00	0.0231	0.0094
1.21 - 1.06	759	760	99.9	18.40	42.46	81.52	0.0260	0.0098
1.06 - 0.96	766	766	100.0	17.49	27.21	72.59	0.0309	0.0094
0.96 - 0.89	796	796	100.0	17.51	20.13	64.93	0.0336	0.0101
0.89 - 0.84	700	700	100.0	16.79	14.89	57.91	0.0334	0.0113
0.84 - 0.80	705	705	100.0	15.17	12.27	50.85	0.0357	0.0132
0.80 - 0.76	864	864	100.0	14.89	10.78	46.96	0.0365	0.0142
0.76 - 0.73	760	760	100.0	14.10	9.34	41.26	0.0425	0.0160
0.73 - 0.71	586	586	100.0	13.59	8.02	37.83	0.0453	0.0181
0.71 - 0.68	1060	1060	100.0	13.34	7.67	34.39	0.0475	0.0191
0.68 - 0.66	758	758	100.0	13.22	6.18	30.43	0.0546	0.0217
0.66 - 0.65	423	423	100.0	12.90	6.29	29.48	0.0562	0.0223
0.65 - 0.63	939	939	100.0	12.09	5.20	26.27	0.0664	0.0265
0.63 - 0.61	1077	1077	100.0	11.08	4.16	20.50	0.0894	0.0334
0.61 - 0.60	571	571	100.0	10.69	3.58	18.16	0.1090	0.0387
0.60 - 0.59	642	642	100.0	10.34	3.89	18.87	0.1082	0.0374
0.59 - 0.58	624	624	100.0	8.54	3.27	15.25	0.1318	0.0486
0.58 - 0.57	707	724	97.7	3.60	2.80	8.58	0.1385	0.0893
0.57 - 0.55	627	1207	51.9	0.97	2.86	6.23	0.1229	0.1279
0.65 - 0.55	5187	5784	89.7	7.80	3.80	17.10	0.0933	0.0485
Inf - 0.55	14861	15586	95.3	12.98	15.53	43.32	0.0329	0.0148

Complete .cif-data of the compound are available under the CCDC number CCDC-**2377885**.

Table S5: Crystal data and structure refinement of **4g'**.

Identification code	15347	
Empirical formula	$C_{28} H_{21} F_4 O_2 P$	
Color	clear colourless	
Formula weight	496.42 g · mol^{-1}	
Temperature	100(2) K	
Wavelength	0.71073 Å	
Crystal system	MONOCLINIC	
Space group	$P2_1/n$, (no. 14)	
Unit cell dimensions	a = 19.7149(12) Å	α= 90°.
	b = 5.7656(3) Å	β= 103.491(2)°.
	c = 21.4357(13) Å	γ = 90°.
Volume	2369.3(2) $Å^3$	
Z	4	
Density (calculated)	1.392 Mg · m^{-3}	
Absorption coefficient	0.172 mm^{-1}	
F(000)	1024 e	
Crystal size	0.361 x 0.204 x 0.120 mm^3	
θ range for data collection	3.188 to 38.568°.	
Index ranges	$-34 \le h \le 34$, $-10 \le k \le 9$, $-37 \le l \le 37$	
Reflections collected	192509	
Independent reflections	13235 [R_{int} = 0.0329]	
Reflections with I>2σ(I)	11898	
Completeness to θ = 25.242°	96.9 %	
Absorption correction	Numerical	
Max. and min. transmission	1.00 and 0.93	
Refinement method	Full-matrix least-squares on F^2	
Data / restraints / parameters	13235 / 0 / 318	
Goodness-of-fit on F^2	1.050	
Final R indices [I>2σ(I)]	R_1 = 0.0353	wR^2 = 0.1036
R indices (all data)	R_1 = 0.0409	wR^2 = 0.0353
Largest diff. peak and hole	0.6 and -0.3 e · $Å^{-3}$	

Table S6: Bond lengths [Å] and angles [°] of **4g’**.

P(1)-O(1)	1.4843(5)	P(1)-C(1)	1.8311(6)
P(1)-C(17)	1.8057(6)	P(1)-C(23)	1.7943(6)
F(1)-C(2)	1.3355(7)	F(2)-C(3)	1.3411(7)
F(3)-C(5)	1.3454(7)	F(4)-C(6)	1.3374(7)
O(2)-C(7)	1.2168(8)	C(1)-C(2)	1.3967(8)
C(1)-C(6)	1.3927(8)	C(2)-C(3)	1.3811(8)
C(3)-C(4)	1.3843(9)	C(4)-C(5)	1.3807(9)
C(4)-C(7)	1.5162(9)	C(5)-C(6)	1.3857(9)
C(7)-C(8)	1.4722(8)	C(8)-C(9)	1.4016(8)
C(8)-C(13)	1.3958(8)	C(9)-C(10)	1.3841(9)
C(10)-C(11)	1.3999(9)	C(11)-C(12)	1.3993(8)
C(11)-C(14)	1.5152(8)	C(12)-C(13)	1.3920(8)
C(14)-C(15)	1.5257(10)	C(14)-C(16)	1.5344(10)
C(17)-C(18)	1.3990(8)	C(17)-C(22)	1.3965(8)
C(18)-C(19)	1.3926(9)	C(19)-C(20)	1.3909(10)
C(20)-C(21)	1.3899(10)	C(21)-C(22)	1.3963(9)
C(23)-C(24)	1.3942(10)	C(23)-C(28)	1.3981(10)
C(24)-C(25)	1.3929(11)	C(25)-C(26)	1.3851(15)
C(26)-C(27)	1.3889(14)	C(27)-C(28)	1.3912(10)

O(1)-P(1)-C(1)	112.32(3)	O(1)-P(1)-C(17)	113.09(3)
O(1)-P(1)-C(23)	112.56(3)	C(17)-P(1)-C(1)	104.41(3)
C(23)-P(1)-C(1)	107.23(3)	C(23)-P(1)-C(17)	106.66(3)
C(2)-C(1)-P(1)	117.57(4)	C(6)-C(1)-P(1)	126.57(4)
C(6)-C(1)-C(2)	115.86(5)	F(1)-C(2)-C(1)	119.43(5)
F(1)-C(2)-C(3)	118.05(5)	C(3)-C(2)-C(1)	122.52(5)
F(2)-C(3)-C(2)	119.60(5)	F(2)-C(3)-C(4)	119.44(5)
C(2)-C(3)-C(4)	120.96(5)	C(3)-C(4)-C(7)	121.44(5)
C(5)-C(4)-C(3)	117.12(5)	C(5)-C(4)-C(7)	121.31(5)
F(3)-C(5)-C(4)	119.26(6)	F(3)-C(5)-C(6)	118.67(6)
C(4)-C(5)-C(6)	122.07(5)	F(4)-C(6)-C(1)	121.83(5)
F(4)-C(6)-C(5)	116.78(5)	C(5)-C(6)-C(1)	121.39(5)
O(2)-C(7)-C(4)	118.44(6)	O(2)-C(7)-C(8)	122.87(6)
C(8)-C(7)-C(4)	118.68(5)	C(9)-C(8)-C(7)	118.68(5)
C(13)-C(8)-C(7)	121.76(5)	C(13)-C(8)-C(9)	119.53(5)
C(10)-C(9)-C(8)	119.86(5)	C(9)-C(10)-C(11)	121.21(5)
C(10)-C(11)-C(14)	119.49(5)	C(12)-C(11)-C(10)	118.47(5)
C(12)-C(11)-C(14)	121.91(5)	C(13)-C(12)-C(11)	120.76(5)
C(12)-C(13)-C(8)	120.09(5)	C(11)-C(14)-C(15)	113.63(5)

C(11)-C(14)-C(16)	108.40(6)	C(15)-C(14)-C(16)	110.72(6)
C(18)-C(17)-P(1)	116.06(4)	C(22)-C(17)-P(1)	124.07(4)
C(22)-C(17)-C(18)	119.86(5)	C(19)-C(18)-C(17)	120.04(6)
C(20)-C(19)-C(18)	120.00(6)	C(21)-C(20)-C(19)	120.12(6)
C(20)-C(21)-C(22)	120.26(6)	C(21)-C(22)-C(17)	119.68(6)
C(24)-C(23)-P(1)	117.34(5)	C(24)-C(23)-C(28)	120.28(6)
C(28)-C(23)-P(1)	122.35(5)	C(25)-C(24)-C(23)	119.64(8)
C(26)-C(25)-C(24)	120.00(8)	C(25)-C(26)-C(27)	120.54(7)
C(26)-C(27)-C(28)	119.99(8)	C(27)-C(28)-C(23)	119.55(7)

Single crystal structure analysis of complex 5i

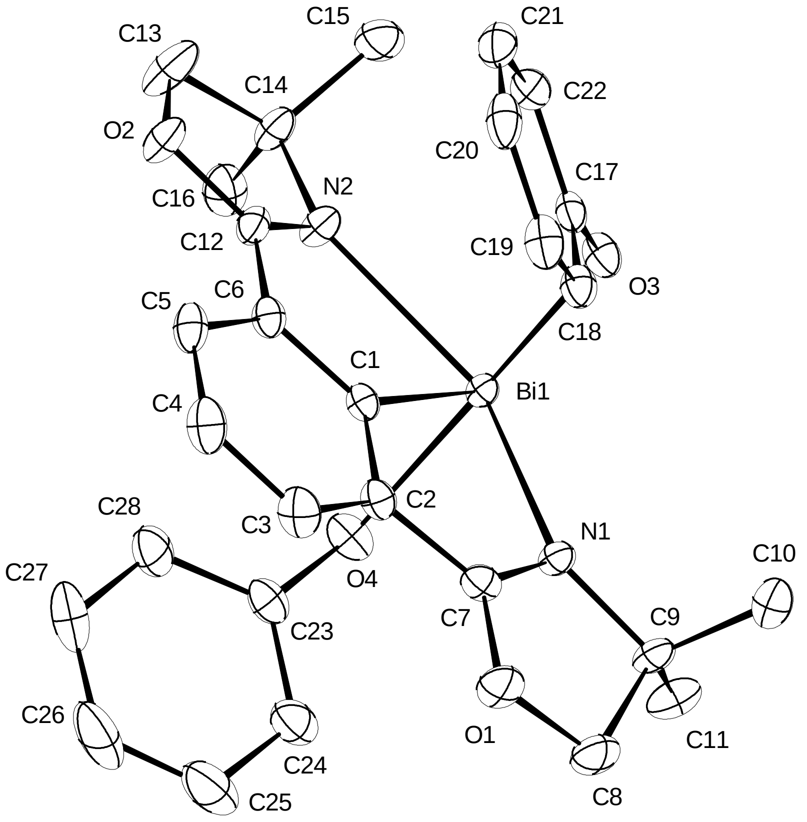

Figure S38. The solid state structure of **5i**. H atoms have been removed for clarity.

X-ray Crystal Structure Analysis of **5i**:

$C_{28}H_{29}BiN_2O_4$, M_r = 666.51 g mol^{-1}, yellow prism, crystal size 0.241 x 0.24 x 0.14 mm^3, orthorhombic, space group $P2_12_12_1$ [19], a = 12.3967(9) Å, b = 14.1332(10) Å, c = 14.9439(10) Å, V = 2618.2(3) Å^3,T = 100(2) K, Z = 4, D_{calc} = 1.691 g·cm^3, λ = 0.71073 Å, $\mu(Mo\text{-}K_\alpha)$ = 6.769 mm^{-1}, Gaussian correction ($T_{\min}$ = 0.30992, $T_{\max}$ = 0.47965), Bruker-AXS Kappa Mach3 with APEX-II detector and IµS micro focus Mo-anode X-ray source, 1.983 < θ < 33.794°. 97416 measured reflections, 10468 independent reflections, 10085 reflections with $I > 2\sigma(I)$, R_{int} = 0.0276. The structure was solved by *SHELXT* and refined by full-matrix least-squares (*SHELXL*) against F^2 to R_1 = 0.0123 [$I > 2\sigma(I)$], wR_2 = 0.0270 [all data] with 320 parameters, 0 restraints and an absolute structure parameter x = -0.0338(11).

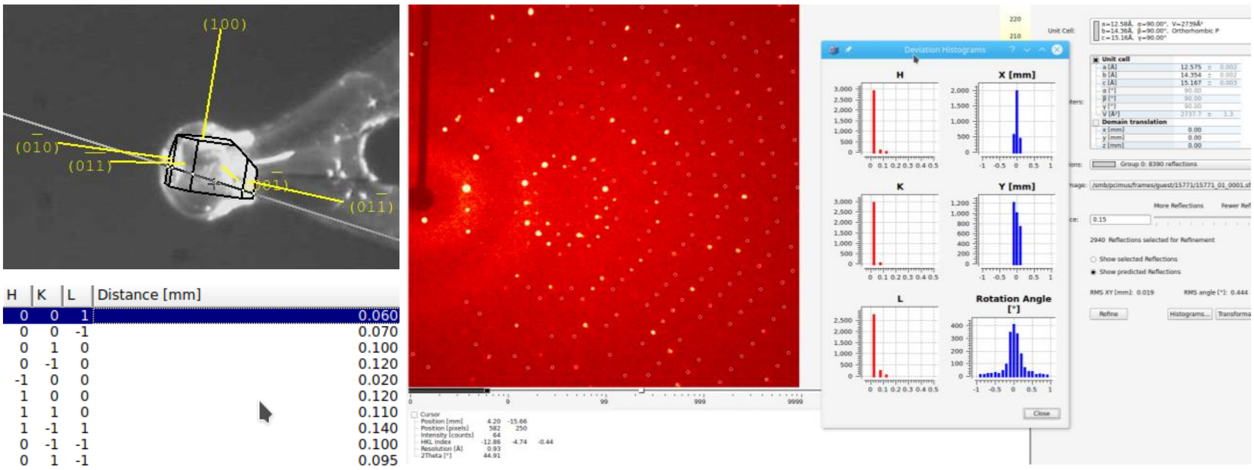

Figure S39. Crystal faces and unit cell determination/refinement of **5i**.

```
INTENSITY STATISTICS FOR DATASET
```

Resolution	#Data	#Theory	%Complete	Redundancy	Mean I	Mean I/s	Rmerge	Rsigma
Inf - 2.64	158	158	100.0	17.09	163.82	137.15	0.0232	0.0063
2.64 - 1.75	367	367	100.0	18.25	122.22	144.13	0.0191	0.0062
1.75 - 1.38	526	526	100.0	18.29	83.19	134.59	0.0187	0.0066
1.38 - 1.20	554	554	100.0	17.95	64.10	123.83	0.0204	0.0071
1.20 - 1.09	526	526	100.0	16.71	52.88	108.27	0.0229	0.0080
1.09 - 1.01	542	542	100.0	12.36	48.45	84.57	0.0253	0.0102
1.01 - 0.95	540	540	100.0	10.24	38.38	67.26	0.0304	0.0128
0.95 - 0.91	465	465	100.0	8.81	36.40	57.37	0.0320	0.0146
0.91 - 0.87	520	520	100.0	8.07	29.92	47.72	0.0381	0.0172
0.87 - 0.83	615	615	100.0	7.67	27.24	43.45	0.0421	0.0191
0.83 - 0.80	580	580	100.0	7.32	24.26	38.32	0.0457	0.0216
0.80 - 0.78	402	402	100.0	7.08	22.87	35.97	0.0474	0.0234
0.78 - 0.75	716	716	100.0	6.80	21.17	32.54	0.0518	0.0256
0.75 - 0.73	565	565	100.0	6.43	19.70	29.35	0.0547	0.0287
0.73 - 0.72	290	290	100.0	6.46	17.44	26.80	0.0613	0.0315
0.72 - 0.70	650	650	100.0	6.16	17.49	25.68	0.0626	0.0335
0.70 - 0.68	744	744	100.0	5.94	14.30	21.71	0.0683	0.0396
0.68 - 0.67	387	387	100.0	5.77	13.82	20.69	0.0722	0.0425
0.67 - 0.66	437	437	100.0	5.57	13.40	19.22	0.0765	0.0451
0.66 - 0.65	419	419	100.0	5.48	12.99	18.96	0.0770	0.0473
0.65 - 0.64	499	559	89.3	3.17	11.32	12.49	0.0811	0.0782
0.74 - 0.64	3703	3763	98.4	5.53	14.85	21.45	0.0675	0.0420
Inf - 0.64	10502	10562	99.4	9.24	35.51	55.14	0.0275	0.0160

Complete .cif-data of the compound are available under the CCDC number CCDC-**2377884**.

Table S7: Crystal data and structure refinement of **5i**.

Identification code	15771	
Empirical formula	$C_{28} H_{29} Bi N_2 O_4$	
Color	yellow	
Formula weight	666.51 g · mol^{-1}	
Temperature	100(2) K	
Wavelength	0.71073 Å	
Crystal system	ORTHORHOMBIC	
Space group	**$P2_12_12_1$, (no. 19)**	
Unit cell dimensions	a = 12.3967(9) Å	α= 90°.
	b = 14.1332(10) Å	β= 90°.
	c = 14.9439(10) Å	γ = 90°.
Volume	2618.2(3) Å^3	
Z	4	
Density (calculated)	1.691 Mg · m^{-3}	
Absorption coefficient	6.769 mm^{-1}	
F(000)	1304 e	
Crystal size	0.241 x 0.24 x 0.14 mm^3	
θ range for data collection	1.983 to 33.794°.	
Index ranges	$-19 \le h \le 19$, $-21 \le k \le 22$, $-23 \le l \le 23$	
Reflections collected	97416	
Independent reflections	10468 [R_{int} = 0.0276]	
Reflections with I>2σ(I)	10085	
Completeness to θ = 25.242°	100.0 %	
Absorption correction	Gaussian	
Max. and min. transmission	0.48 and 0.31	
Refinement method	Full-matrix least-squares on F^2	
Data / restraints / parameters	10468 / 0 / 320	
Goodness-of-fit on F^2	1.027	
Final R indices [I>2σ(I)]	R_1 = 0.0123	wR^2 = 0.0265
R indices (all data)	R_1 = 0.0142	wR^2 = 0.0270
Absolute structure parameter	-0.0338(11)	
Largest diff. peak and hole	0.9 and -0.5 e · Å^{-3}	

Table S8: Bond lengths [Å] and angles [°] of **5i**.

Bi(1)-O(3)	2.2882(13)	Bi(1)-O(4)	2.2991(13)
Bi(1)-N(1)	2.4416(15)	Bi(1)-N(2)	2.4958(16)
Bi(1)-C(1)	2.2221(16)	O(1)-C(7)	1.339(2)
O(1)-C(8)	1.473(3)	O(2)-C(12)	1.336(2)
O(2)-C(13)	1.455(3)	O(3)-C(17)	1.331(2)
O(4)-C(23)	1.336(2)	N(1)-C(7)	1.282(2)
N(1)-C(9)	1.483(2)	N(2)-C(12)	1.282(2)
N(2)-C(14)	1.483(3)	C(1)-C(2)	1.383(3)
C(1)-C(6)	1.384(2)	C(2)-C(3)	1.394(3)
C(2)-C(7)	1.463(3)	C(3)-C(4)	1.387(3)
C(4)-C(5)	1.385(3)	C(5)-C(6)	1.396(2)
C(6)-C(12)	1.465(3)	C(8)-C(9)	1.541(3)
C(9)-C(10)	1.522(3)	C(9)-C(11)	1.518(3)
C(13)-C(14)	1.551(3)	C(14)-C(15)	1.520(3)
C(14)-C(16)	1.519(3)	C(17)-C(18)	1.403(3)
C(17)-C(22)	1.406(3)	C(18)-C(19)	1.388(3)
C(19)-C(20)	1.383(3)	C(20)-C(21)	1.382(3)
C(21)-C(22)	1.396(3)	C(23)-C(24)	1.401(3)
C(23)-C(28)	1.402(3)	C(24)-C(25)	1.393(3)
C(25)-C(26)	1.381(4)	C(26)-C(27)	1.379(4)
C(27)-C(28)	1.384(3)		
O(3)-Bi(1)-O(4)	170.41(5)	O(3)-Bi(1)-N(1)	89.23(5)
O(3)-Bi(1)-N(2)	88.19(5)	O(4)-Bi(1)-N(1)	84.79(5)
O(4)-Bi(1)-N(2)	91.93(6)	N(1)-Bi(1)-N(2)	142.06(5)
C(1)-Bi(1)-O(3)	86.60(5)	C(1)-Bi(1)-O(4)	84.38(6)
C(1)-Bi(1)-N(1)	71.12(6)	C(1)-Bi(1)-N(2)	70.95(6)
C(7)-O(1)-C(8)	104.77(15)	C(12)-O(2)-C(13)	106.08(15)
C(17)-O(3)-Bi(1)	123.78(11)	C(23)-O(4)-Bi(1)	124.63(12)
C(7)-N(1)-Bi(1)	112.03(12)	C(7)-N(1)-C(9)	108.47(15)
C(9)-N(1)-Bi(1)	138.57(11)	C(12)-N(2)-Bi(1)	111.11(13)
C(12)-N(2)-C(14)	108.86(16)	C(14)-N(2)-Bi(1)	140.03(12)
C(2)-C(1)-Bi(1)	119.42(13)	C(2)-C(1)-C(6)	120.32(15)
C(6)-C(1)-Bi(1)	120.15(12)	C(1)-C(2)-C(3)	120.07(18)
C(1)-C(2)-C(7)	115.00(16)	C(3)-C(2)-C(7)	124.93(18)
C(4)-C(3)-C(2)	119.35(19)	C(5)-C(4)-C(3)	120.84(17)
C(4)-C(5)-C(6)	119.35(18)	C(1)-C(6)-C(5)	120.04(17)
C(1)-C(6)-C(12)	115.60(15)	C(5)-C(6)-C(12)	124.22(17)
O(1)-C(7)-C(2)	121.43(17)	N(1)-C(7)-O(1)	117.40(18)
N(1)-C(7)-C(2)	121.16(16)	O(1)-C(8)-C(9)	104.20(15)

N(1)-C(9)-C(8)	101.07(15)	N(1)-C(9)-C(10)	108.93(16)
N(1)-C(9)-C(11)	109.34(15)	C(10)-C(9)-C(8)	111.68(17)
C(11)-C(9)-C(8)	113.95(17)	C(11)-C(9)-C(10)	111.30(18)
O(2)-C(12)-C(6)	120.17(16)	N(2)-C(12)-O(2)	117.78(18)
N(2)-C(12)-C(6)	121.99(17)	O(2)-C(13)-C(14)	105.60(16)
N(2)-C(14)-C(13)	101.58(16)	N(2)-C(14)-C(15)	109.96(17)
N(2)-C(14)-C(16)	108.96(17)	C(15)-C(14)-C(13)	113.23(19)
C(16)-C(14)-C(13)	111.96(19)	C(16)-C(14)-C(15)	110.72(18)
O(3)-C(17)-C(18)	121.54(18)	O(3)-C(17)-C(22)	121.14(18)
C(18)-C(17)-C(22)	117.32(17)	C(19)-C(18)-C(17)	121.47(19)
C(20)-C(19)-C(18)	120.5(2)	C(21)-C(20)-C(19)	119.17(19)
C(20)-C(21)-C(22)	120.9(2)	C(21)-C(22)-C(17)	120.6(2)
O(4)-C(23)-C(24)	120.93(19)	O(4)-C(23)-C(28)	121.66(19)
C(24)-C(23)-C(28)	117.40(19)	C(25)-C(24)-C(23)	120.9(2)
C(26)-C(25)-C(24)	120.6(2)	C(27)-C(26)-C(25)	119.1(2)
C(26)-C(27)-C(28)	120.9(2)	C(27)-C(28)-C(23)	121.0(2)

7. NMR spectra

^{1}H NMR of **1a'** (400 MHz, $CDCl_3$, 298K)

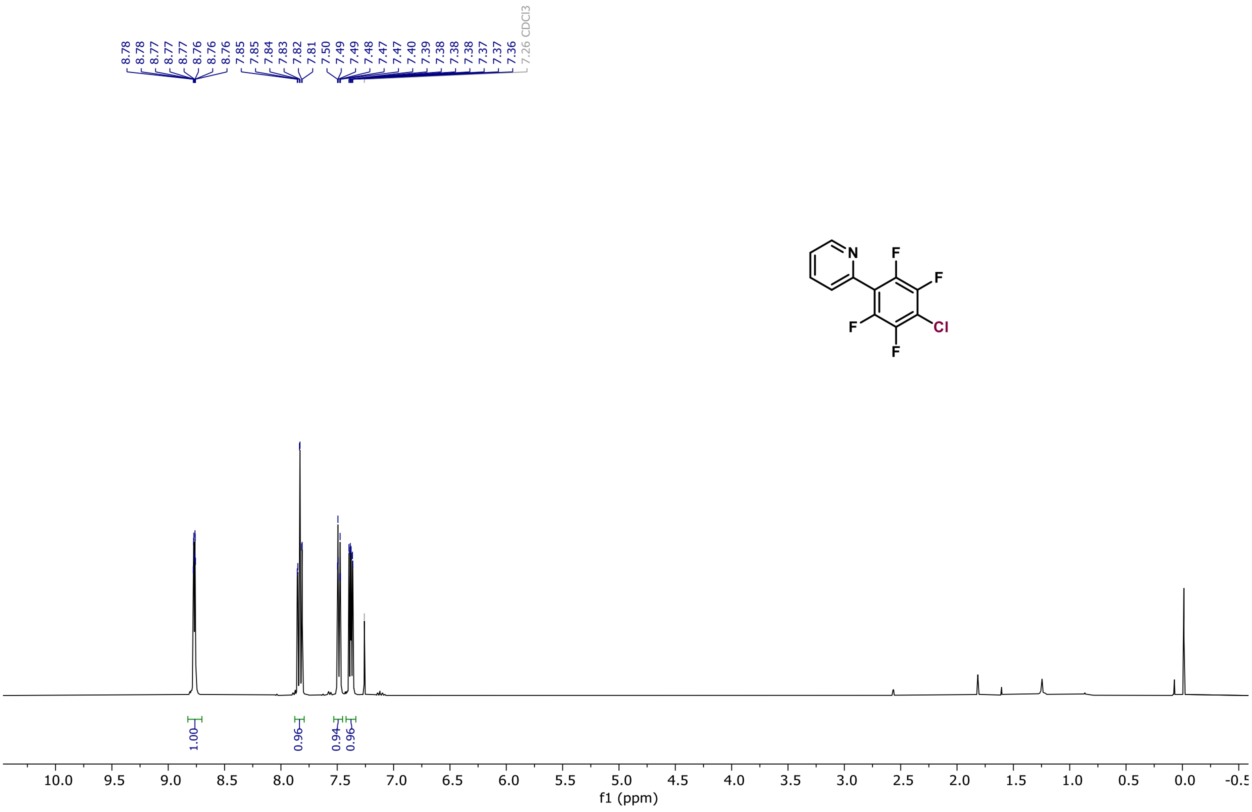

^{19}F NMR of **1a'** (282 MHz, $CDCl_3$, 298K)

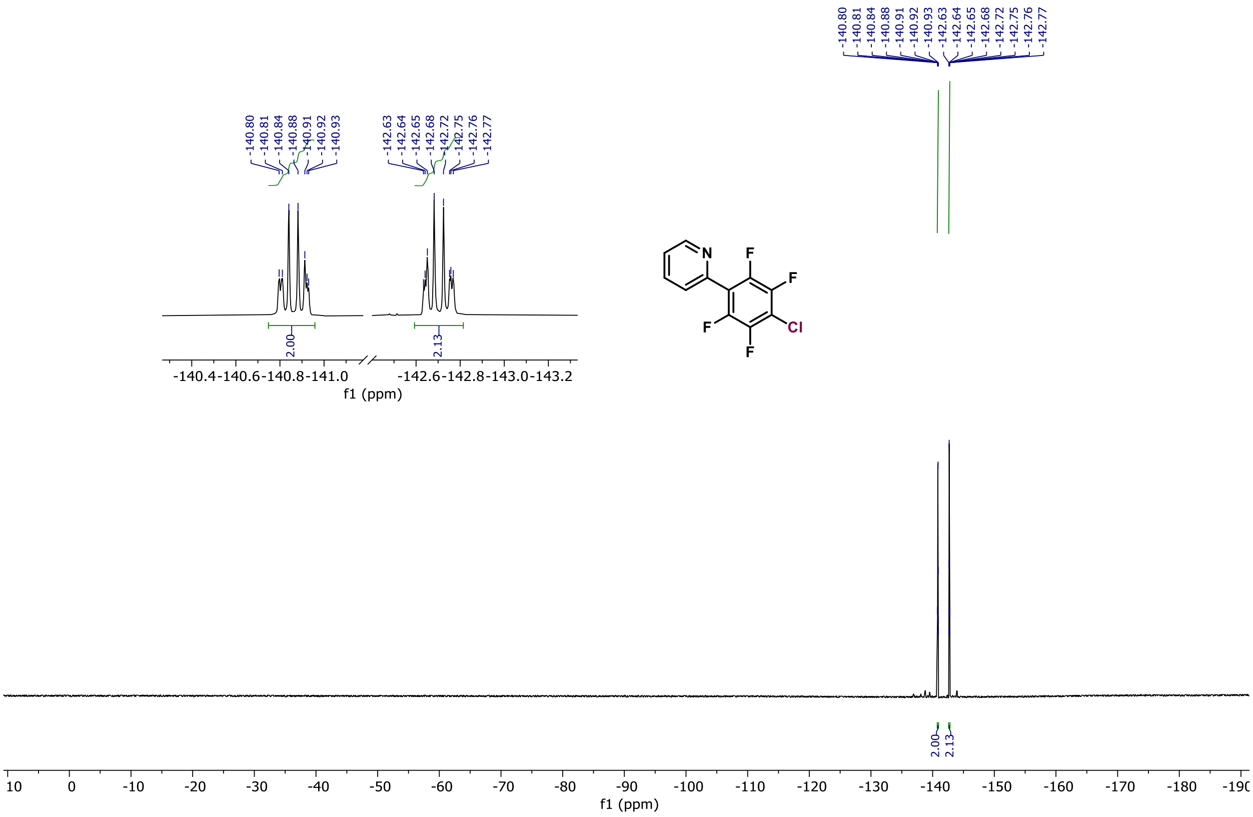

^{13}C NMR of **1a’** (101 MHz, $CDCl_3$, 298K)

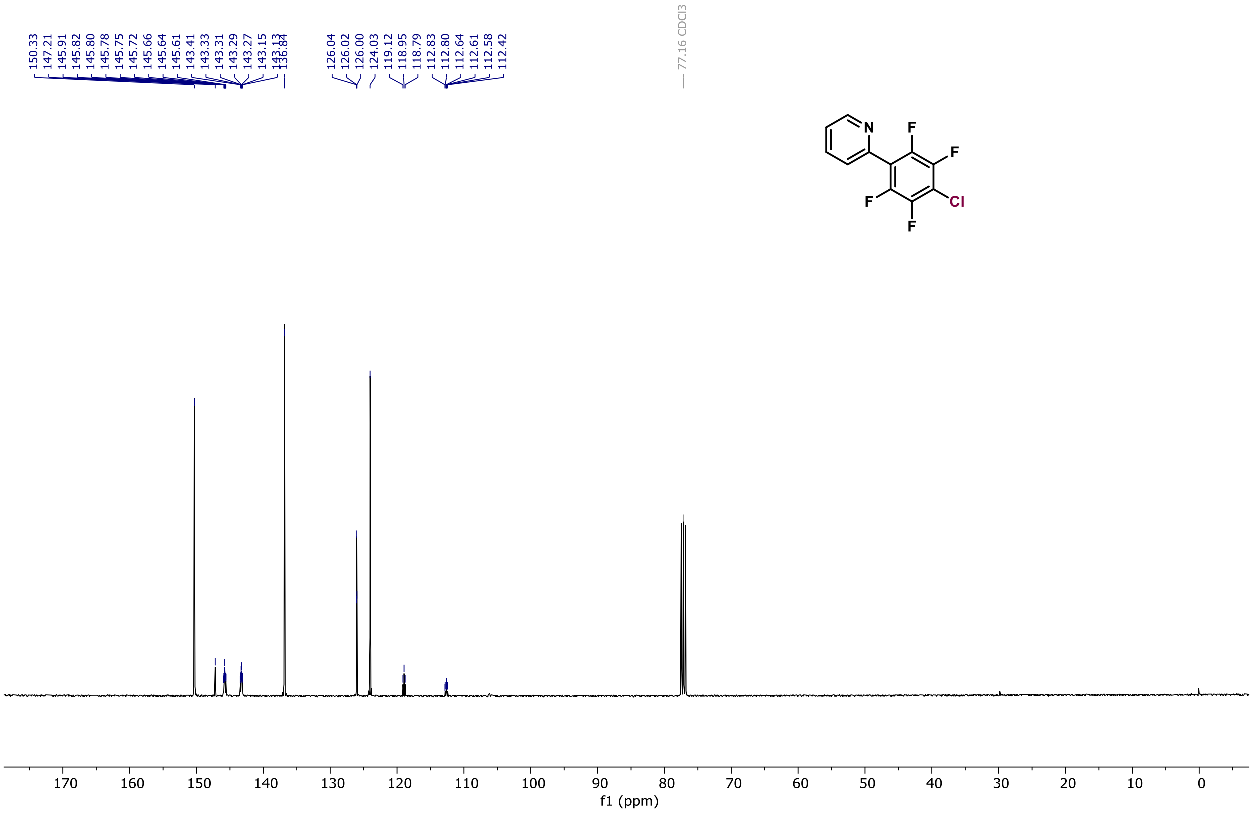

^{1}H NMR of **1l** (400 MHz, $CDCl_3$, 298K)

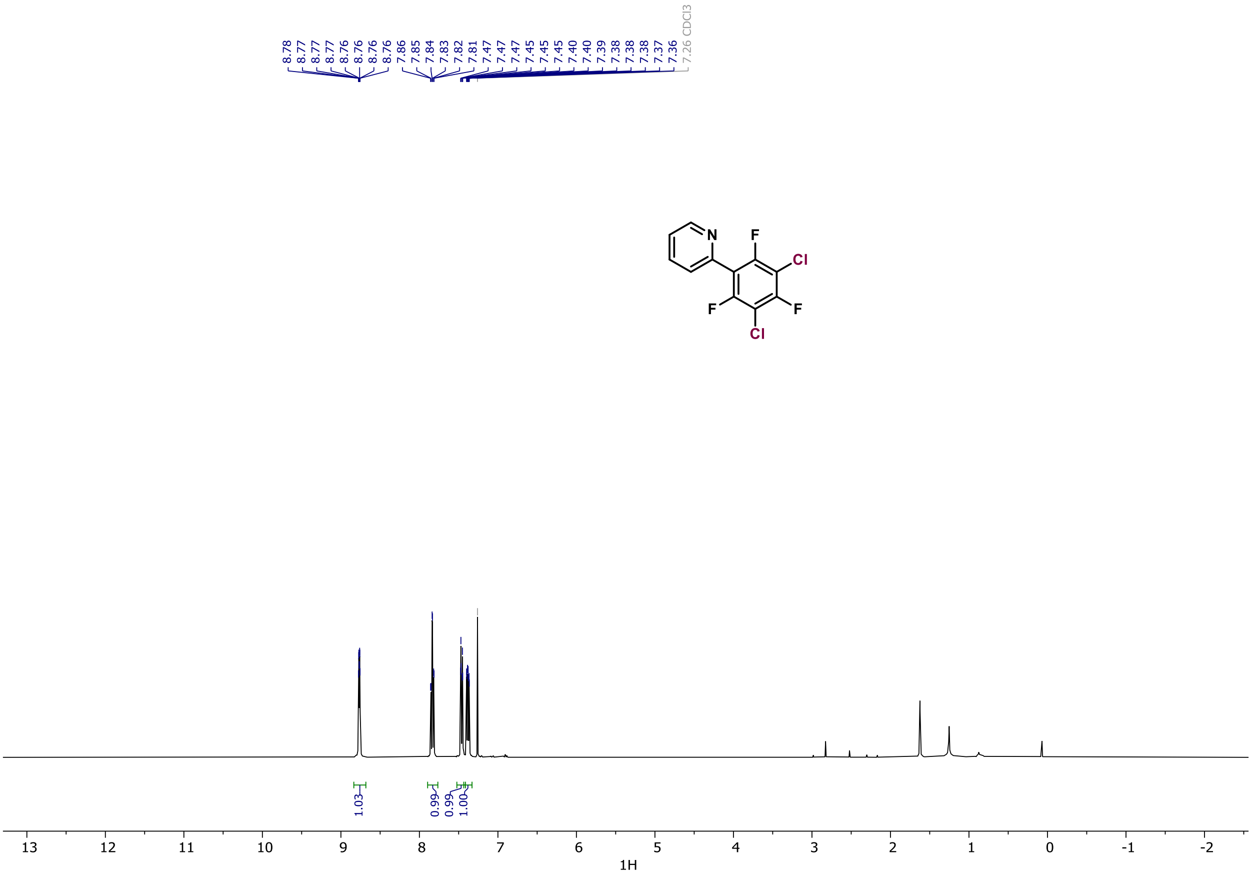

^{19}**F NMR** of **1l** (282 MHz, $CDCl_3$, 298K)

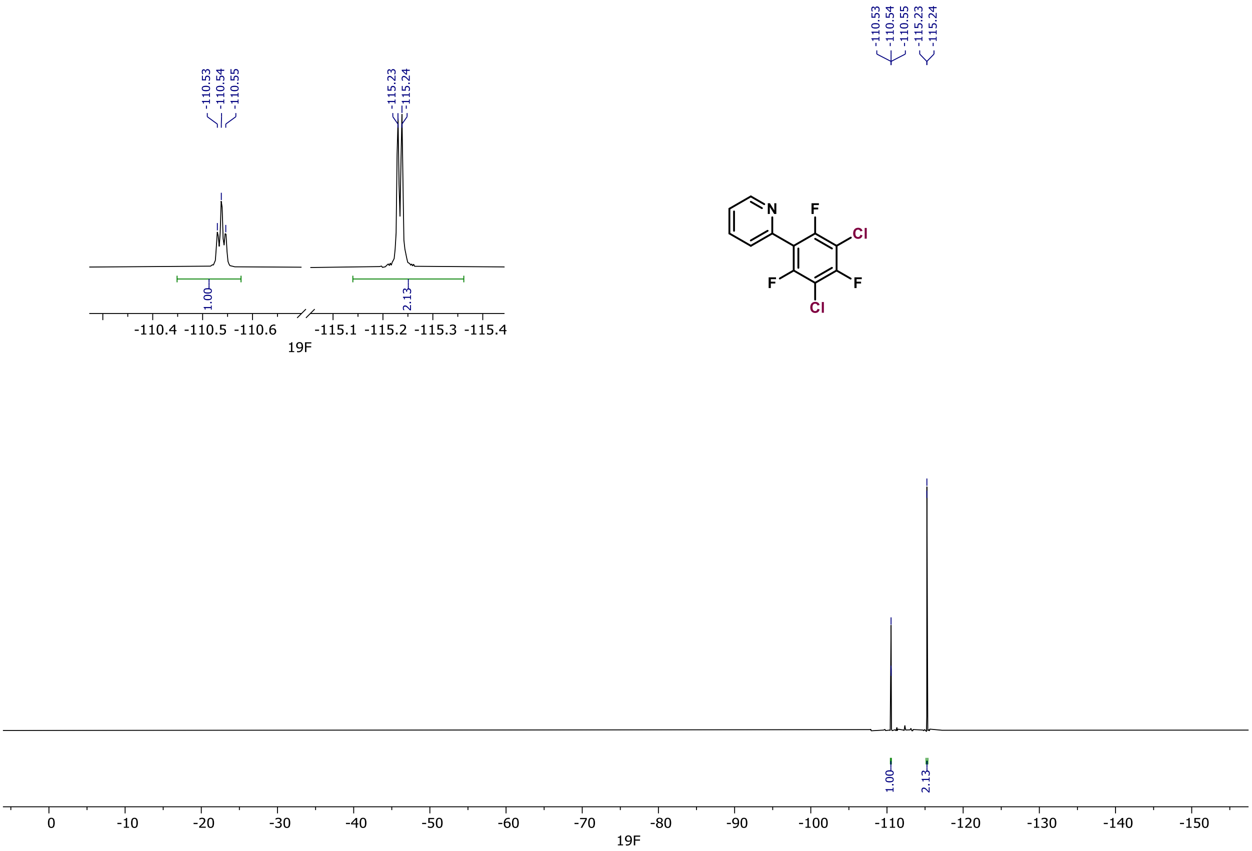

^{13}**C NMR** of **1l** (101 MHz, $CDCl_3$, 298K)

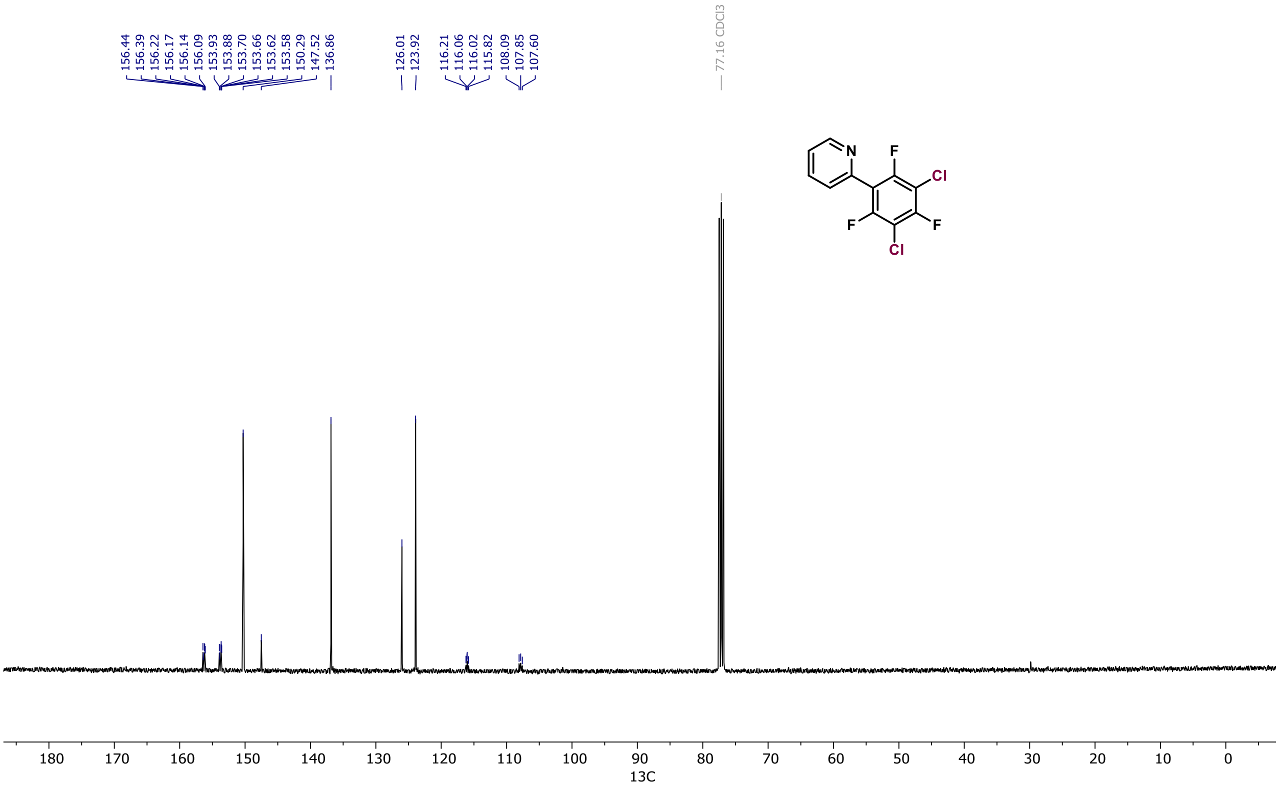

^{1}H NMR of **4a** (600 MHz, $CDCl_3$, 298K)

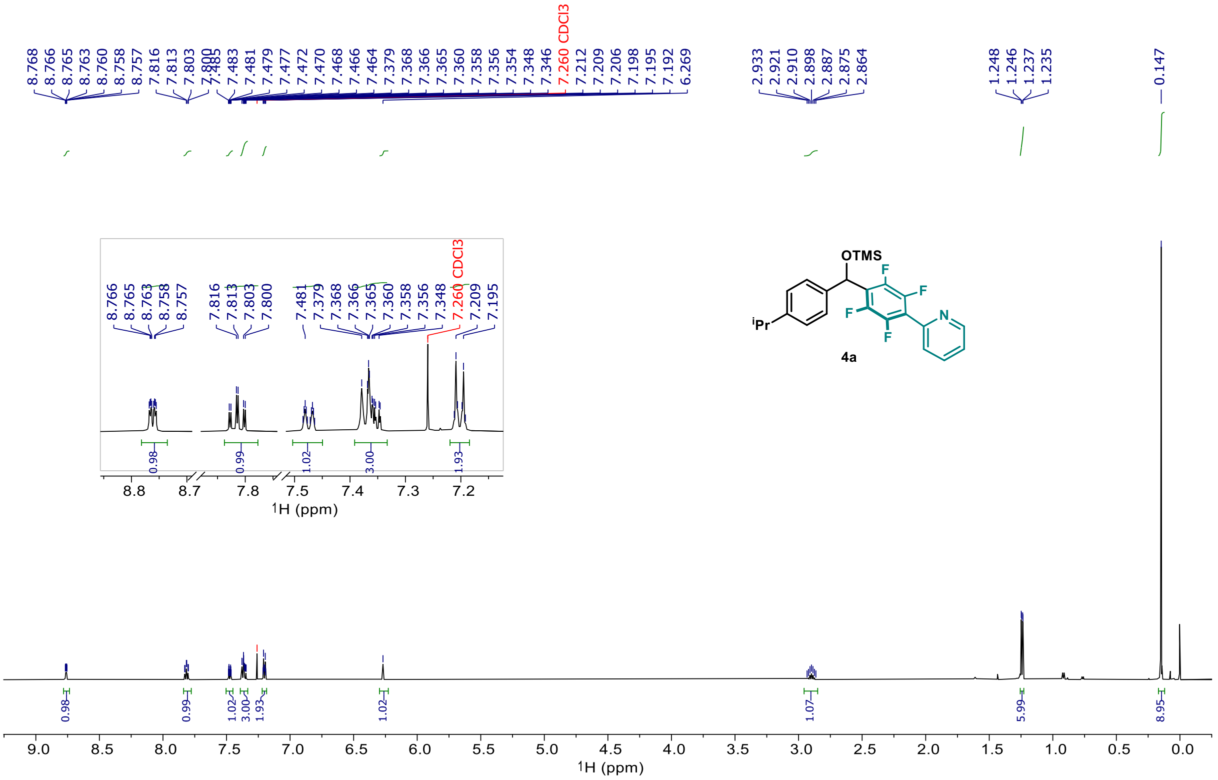

^{19}F NMR of **4a** (565 MHz, $CDCl_3$, 298K)

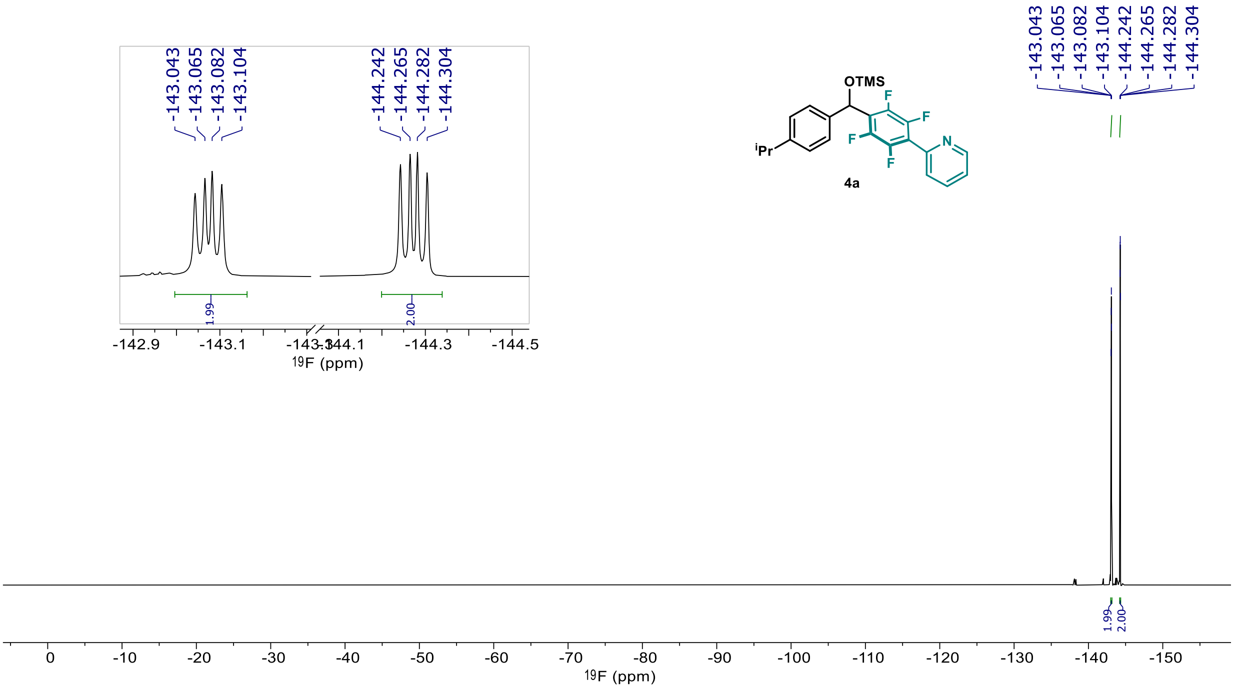

^{13}C NMR of **4a** (151 MHz, $CDCl_3$, 298K)

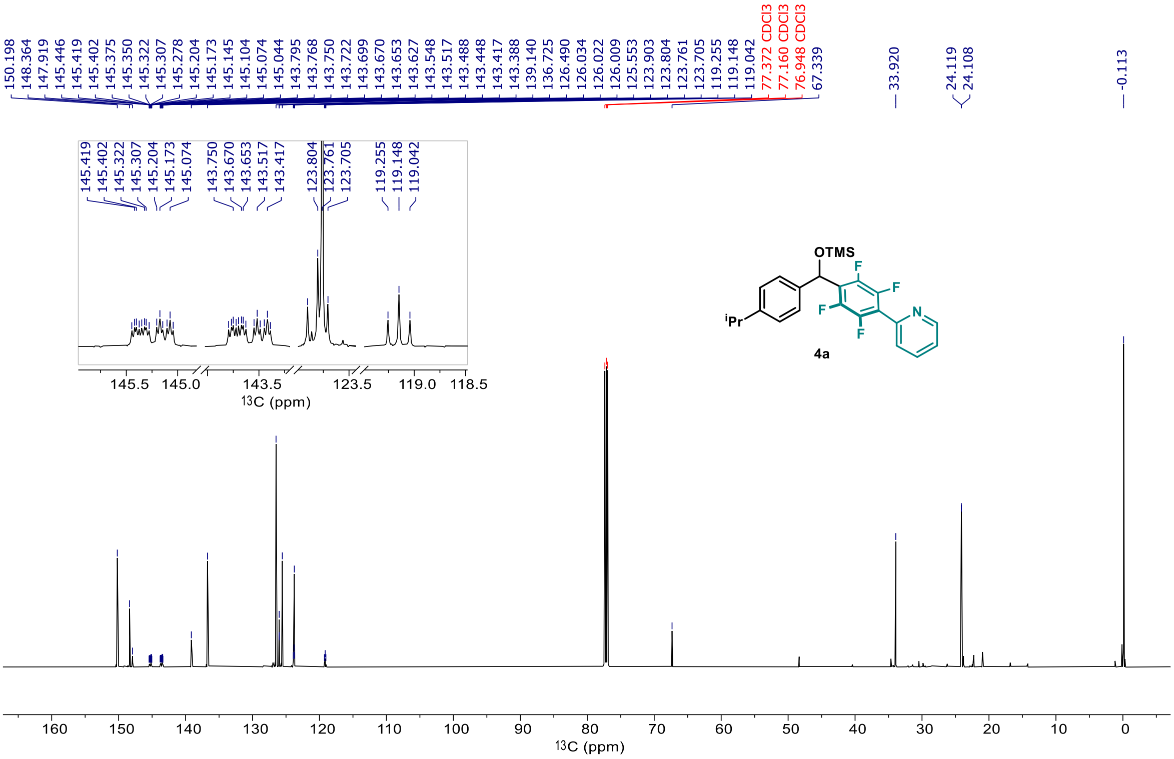

^{29}Si NMR of **4a** (119 MHz, $CDCl_3$, 298K)

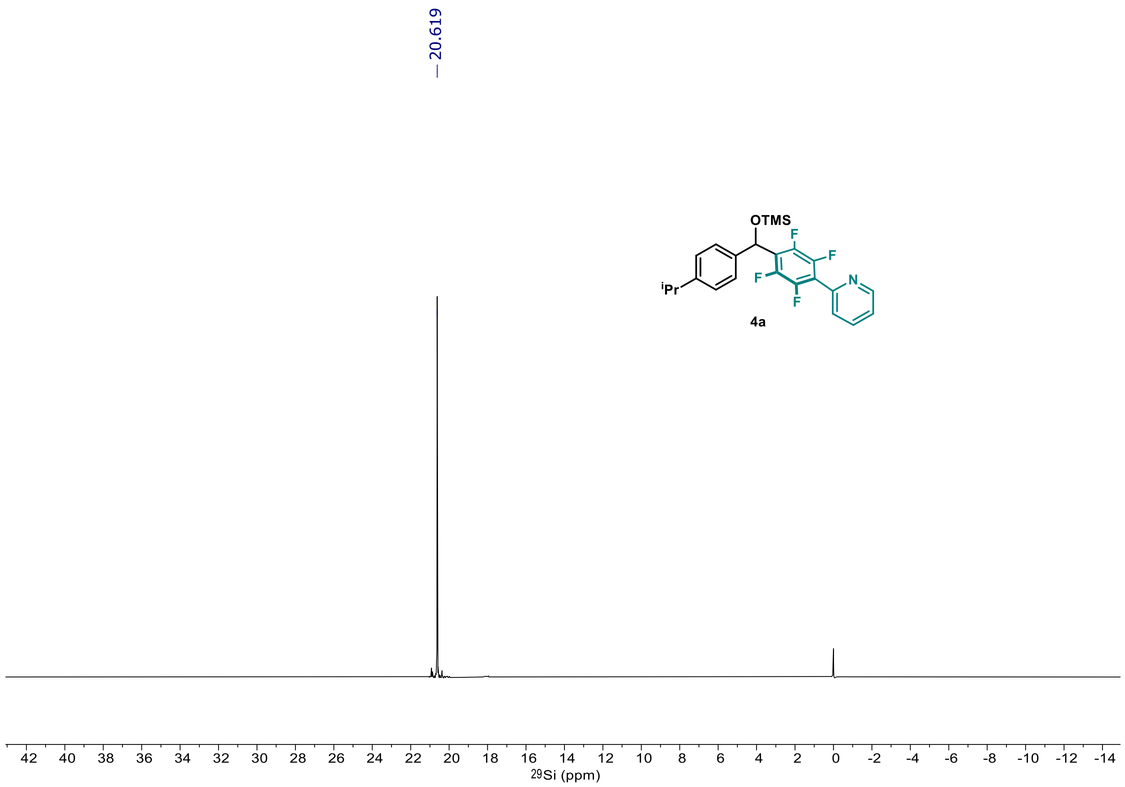

^{1}H-^{1}H COSY of **4a** (600 MHz, $CDCl_3$, 298K)

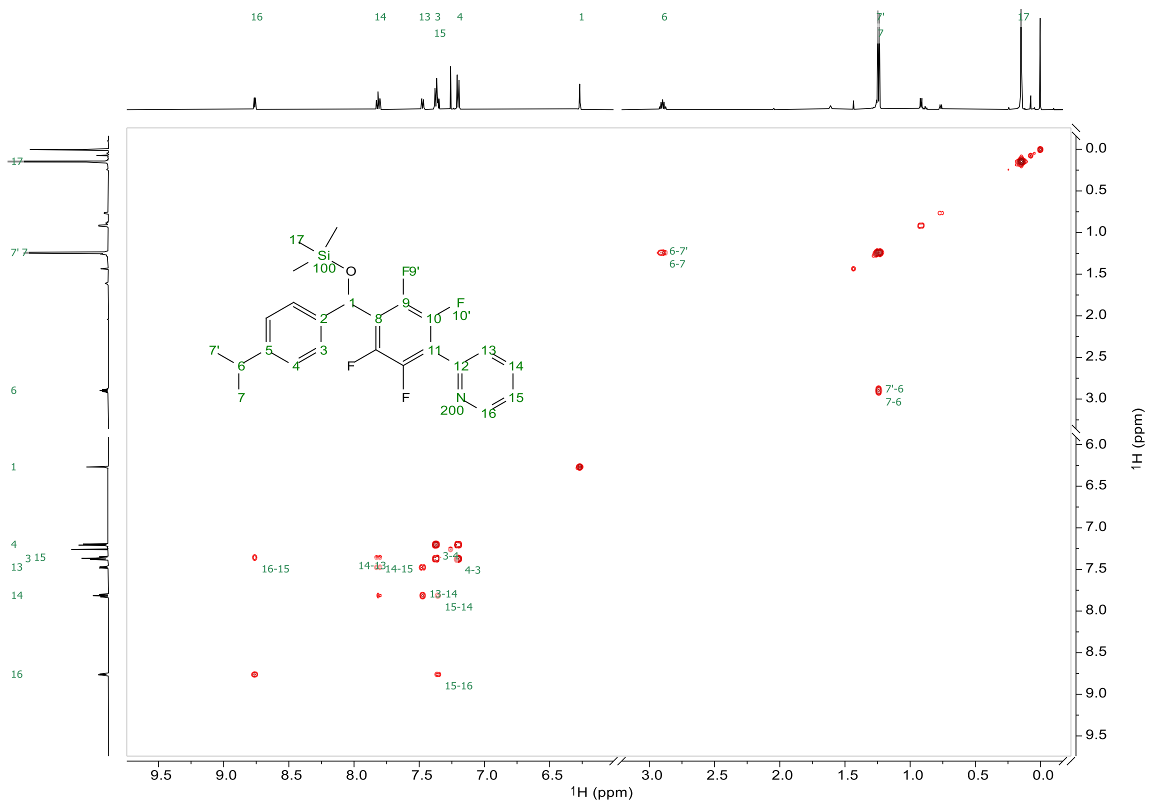

[1]H-^{1}H NOESY of **4a** (600 MHz, $CDCl_3$, 298K)

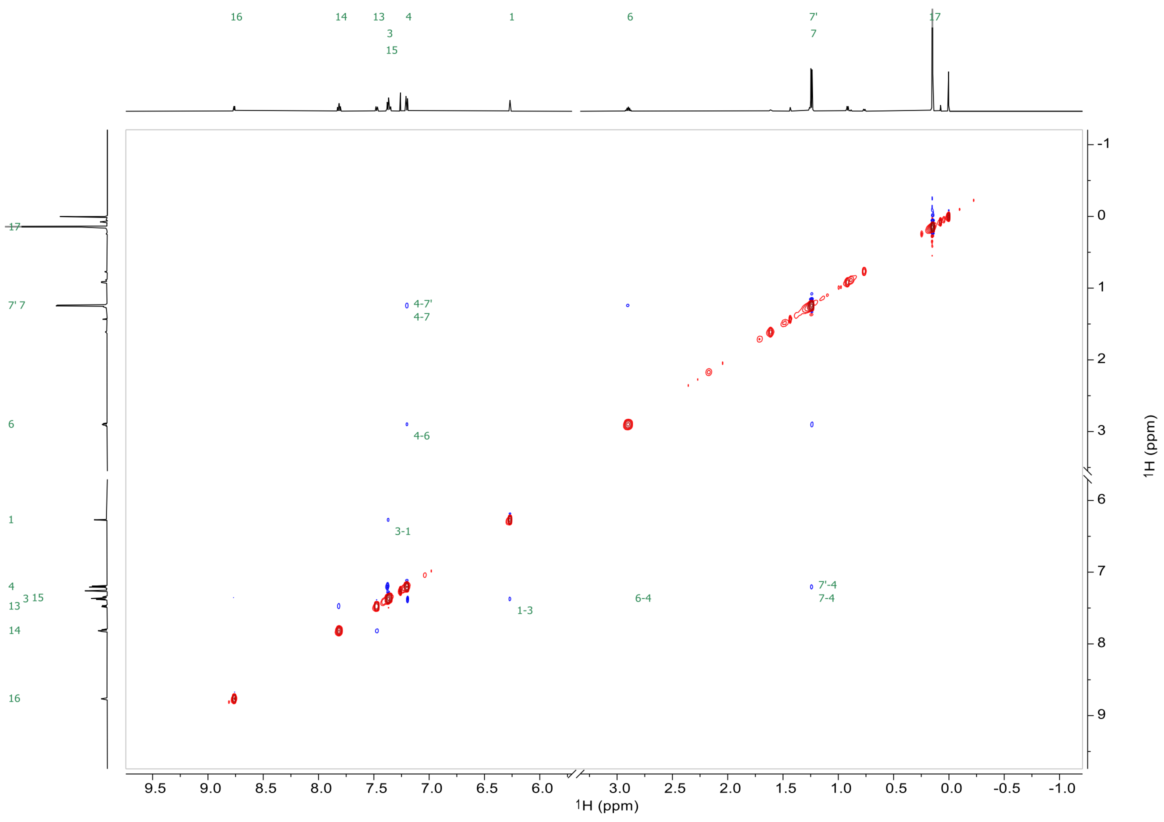

^{1}H-^{13}C HSQC of **4a** (600 MHz, $CDCl_3$, 298K)

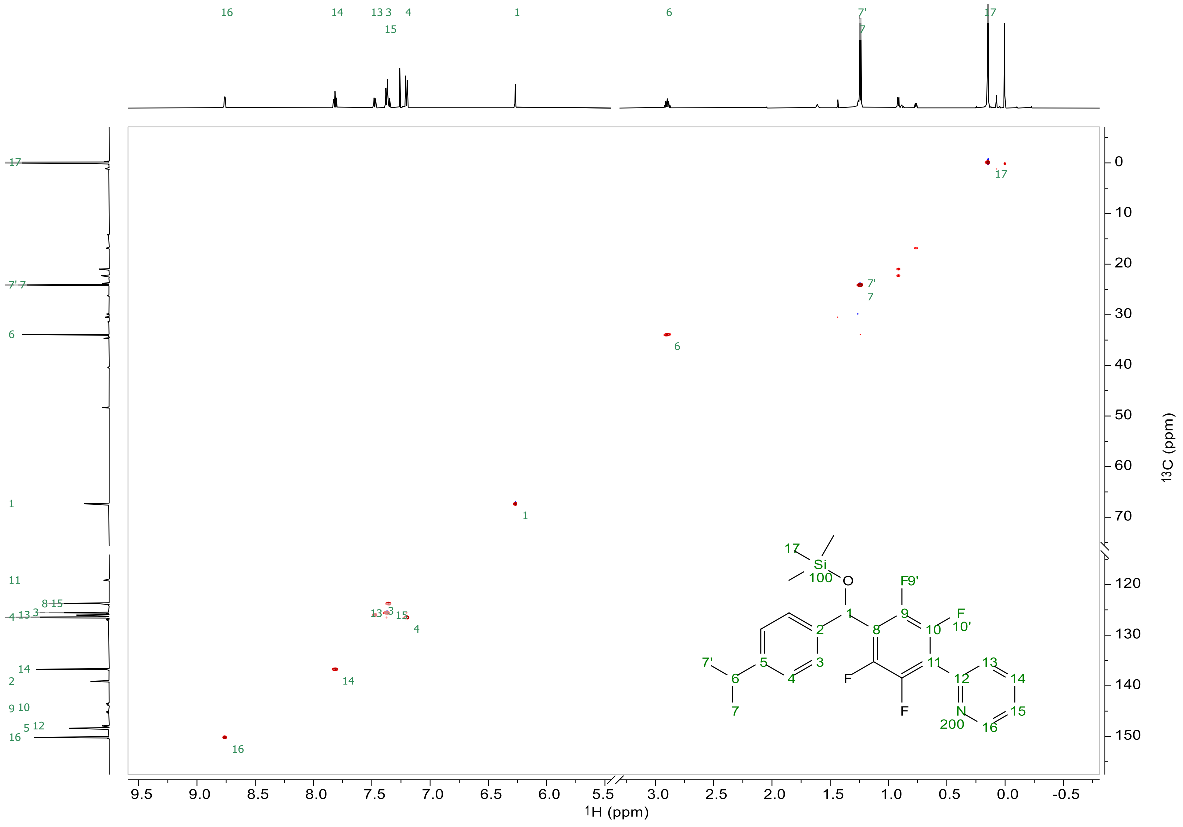

^{1}H-^{13}C HMBC of **4a** (600 MHz, $CDCl_3$, 298K)

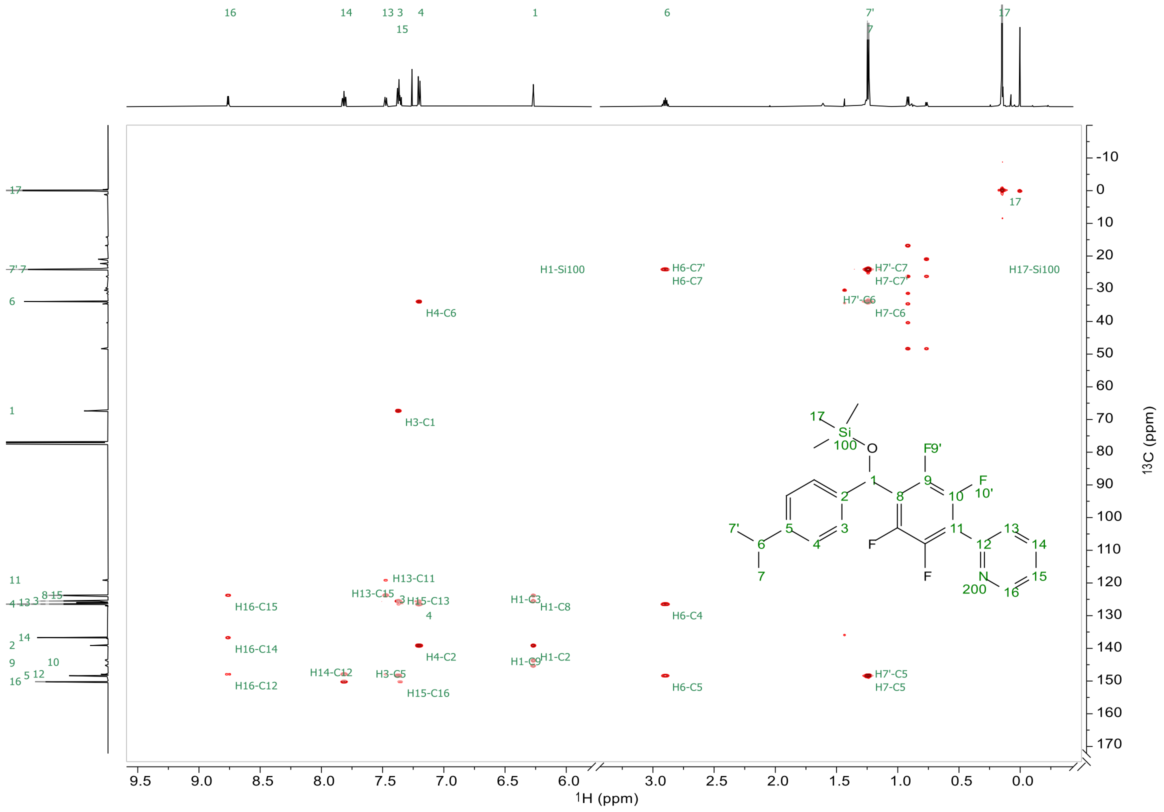

^{1}H NMR of 4b (600 MHz, $CDCl_3$, 298K)

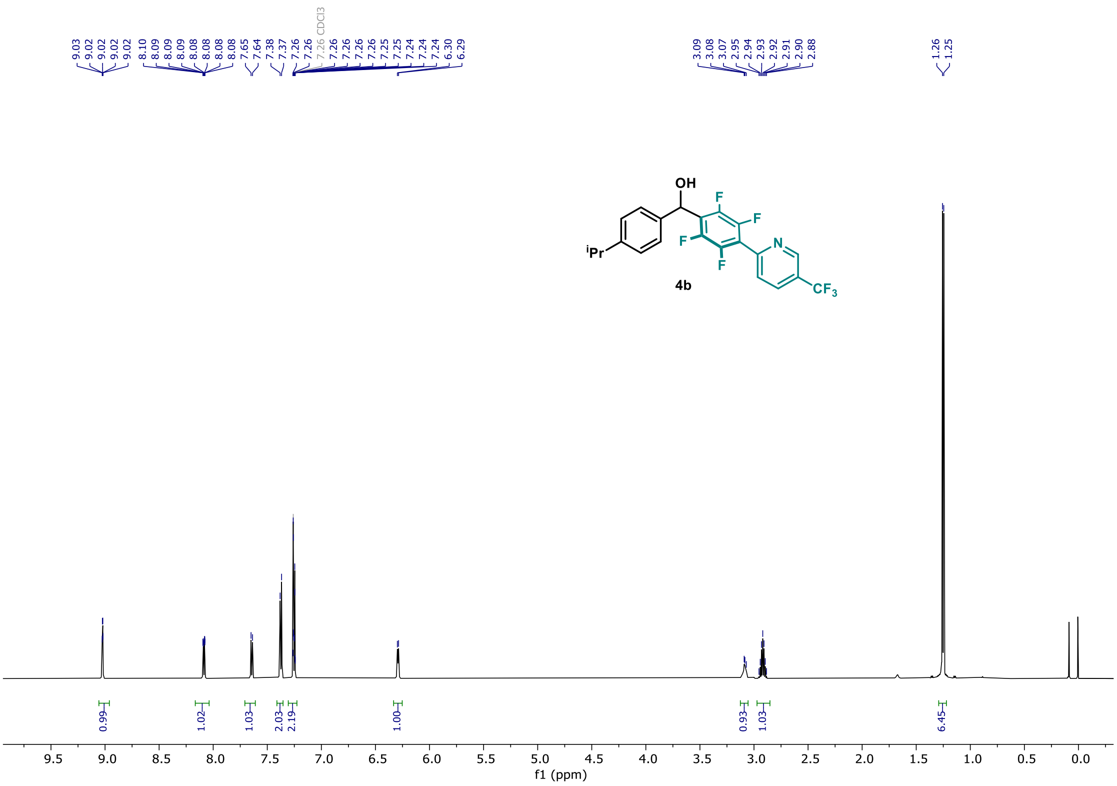

^{19}F NMR of **4b** (565 MHz, $CDCl_3$, 298K)

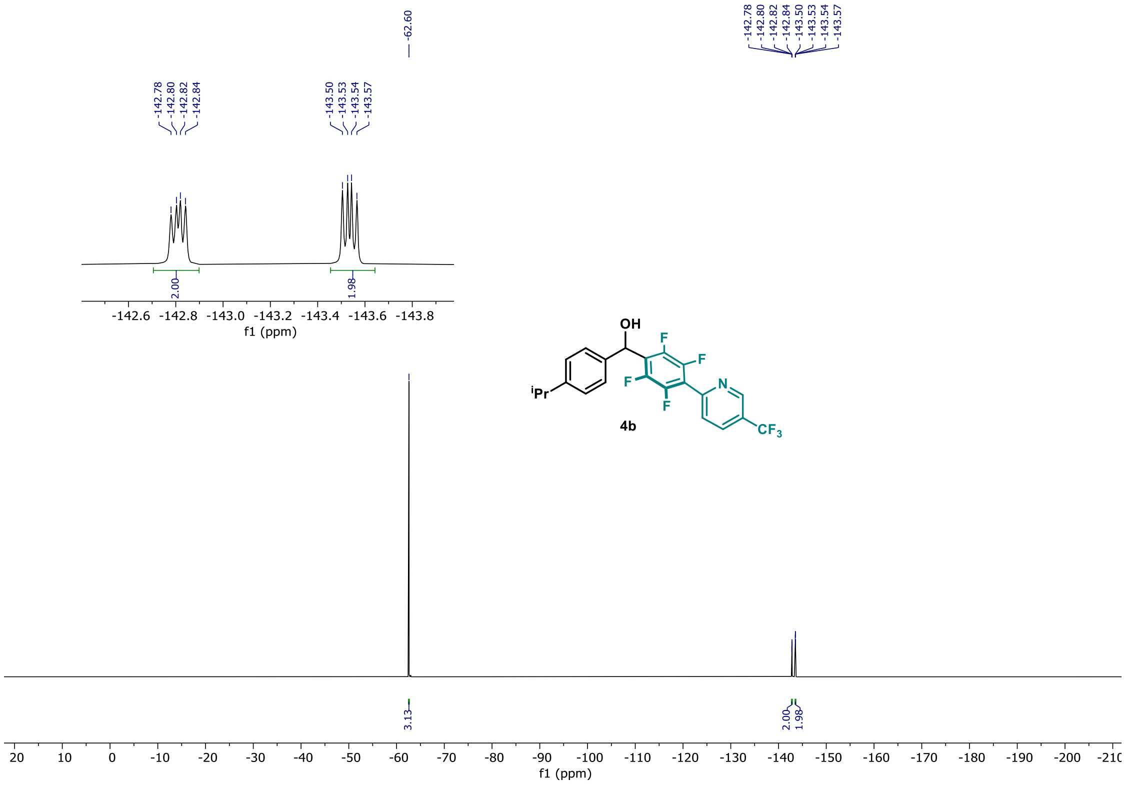

13C NMR of **4b** (151 MHz, $CDCl_3$, 298K)

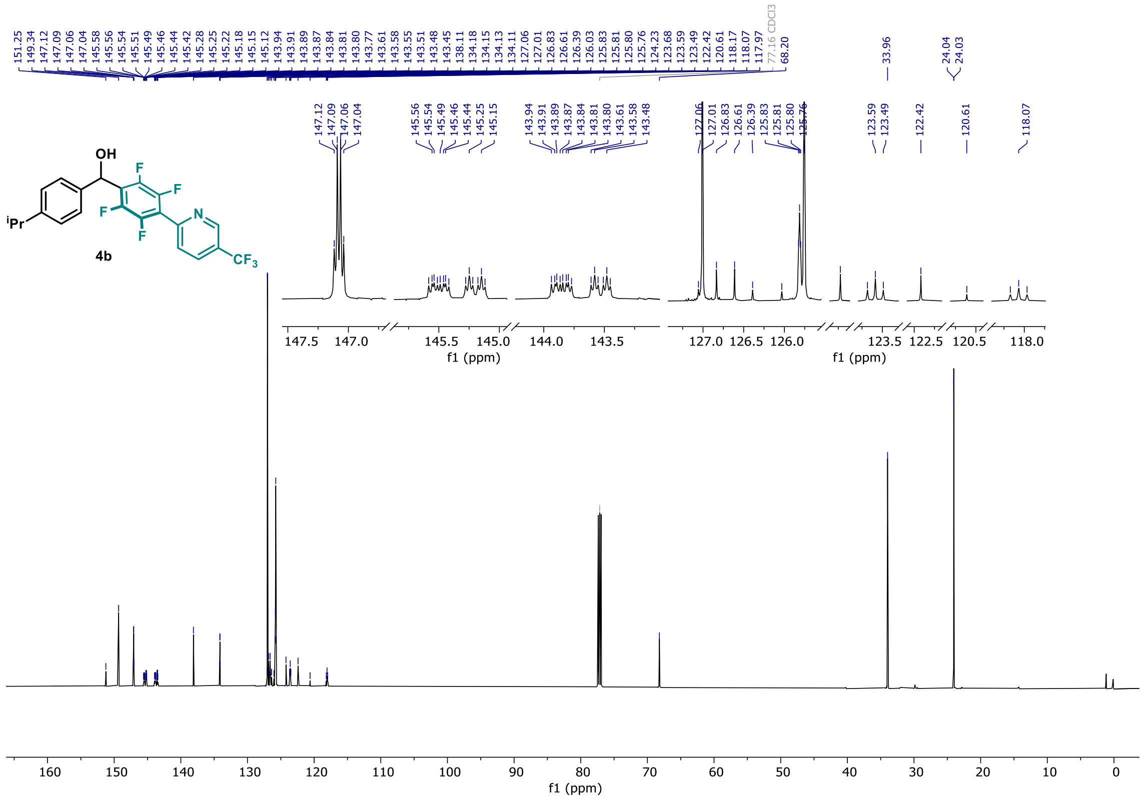

1H NMR of **4c** (300 MHz, $CDCl_3$, 298K)

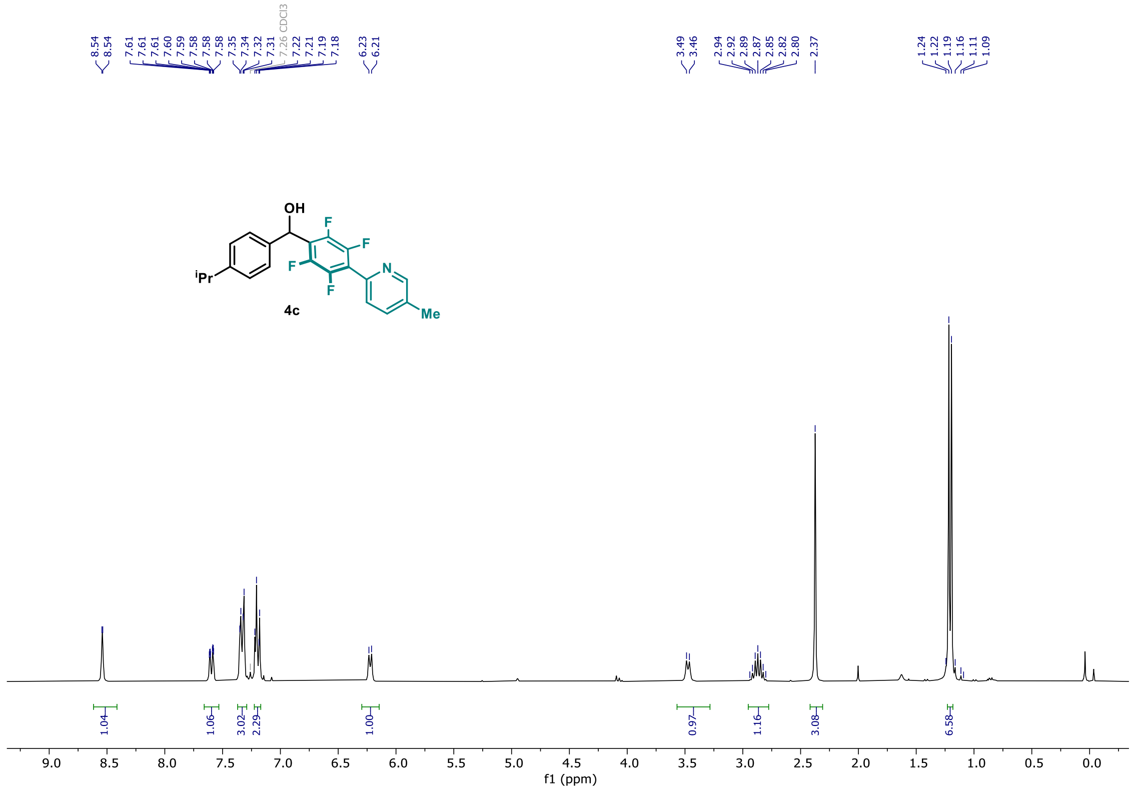

^{19}F NMR of 4c (282 MHz, $CDCl_3$, 298K)

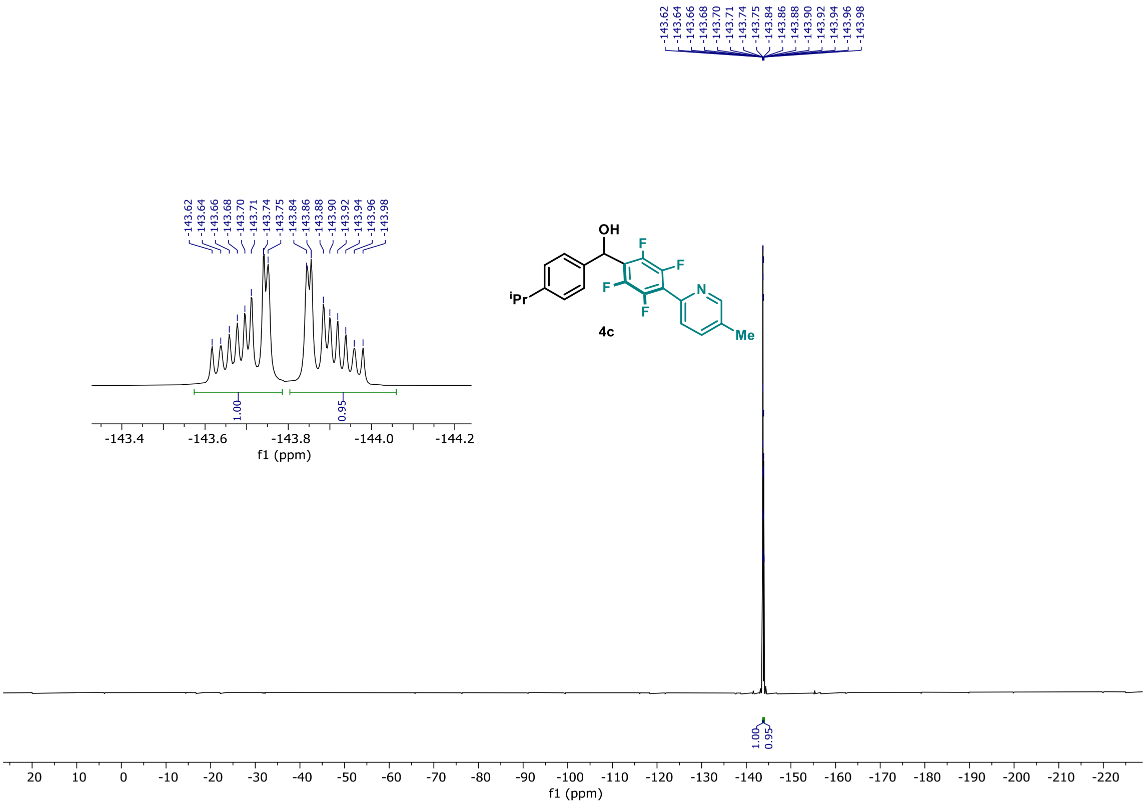

^{13}C NMR of 4c (75 MHz, $CDCl_3$, 298K)

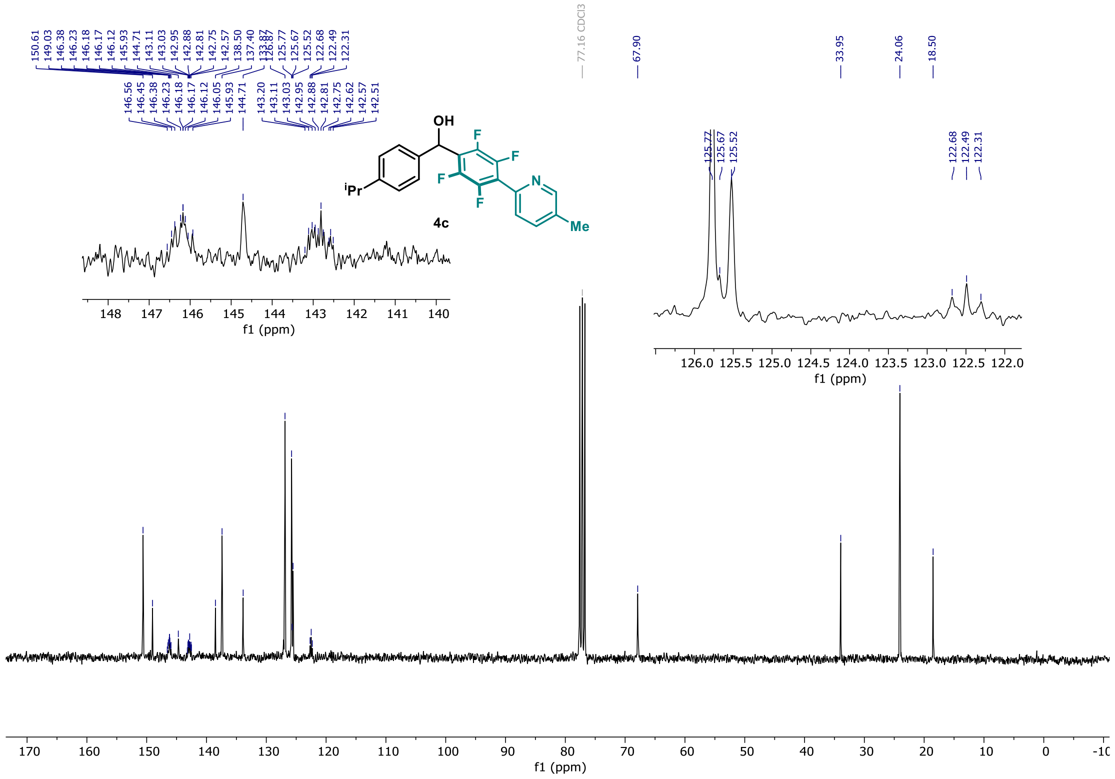

^{1}**H NMR** of **4d** (300 MHz, $CDCl_3$, 298K)

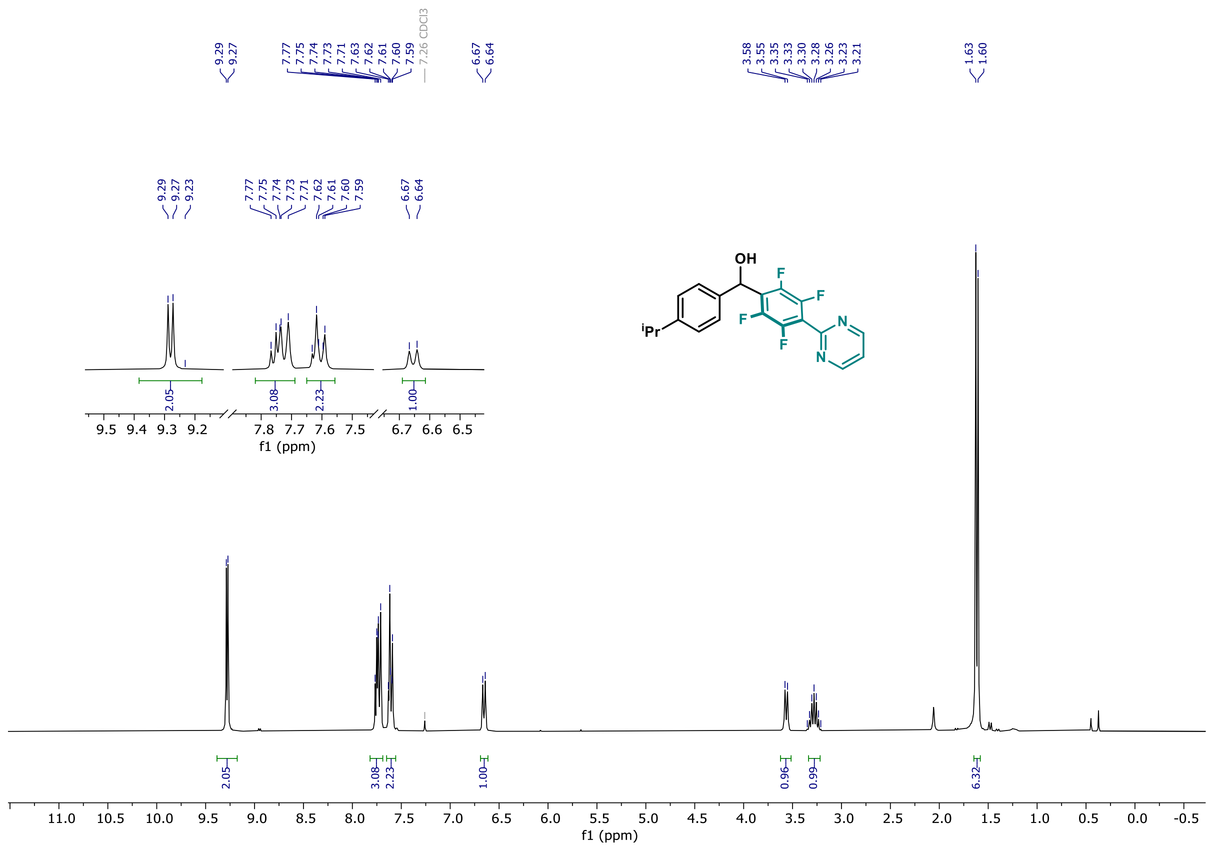

^{19}**F NMR** of **4d** (282 MHz, $CDCl_3$, 298K)

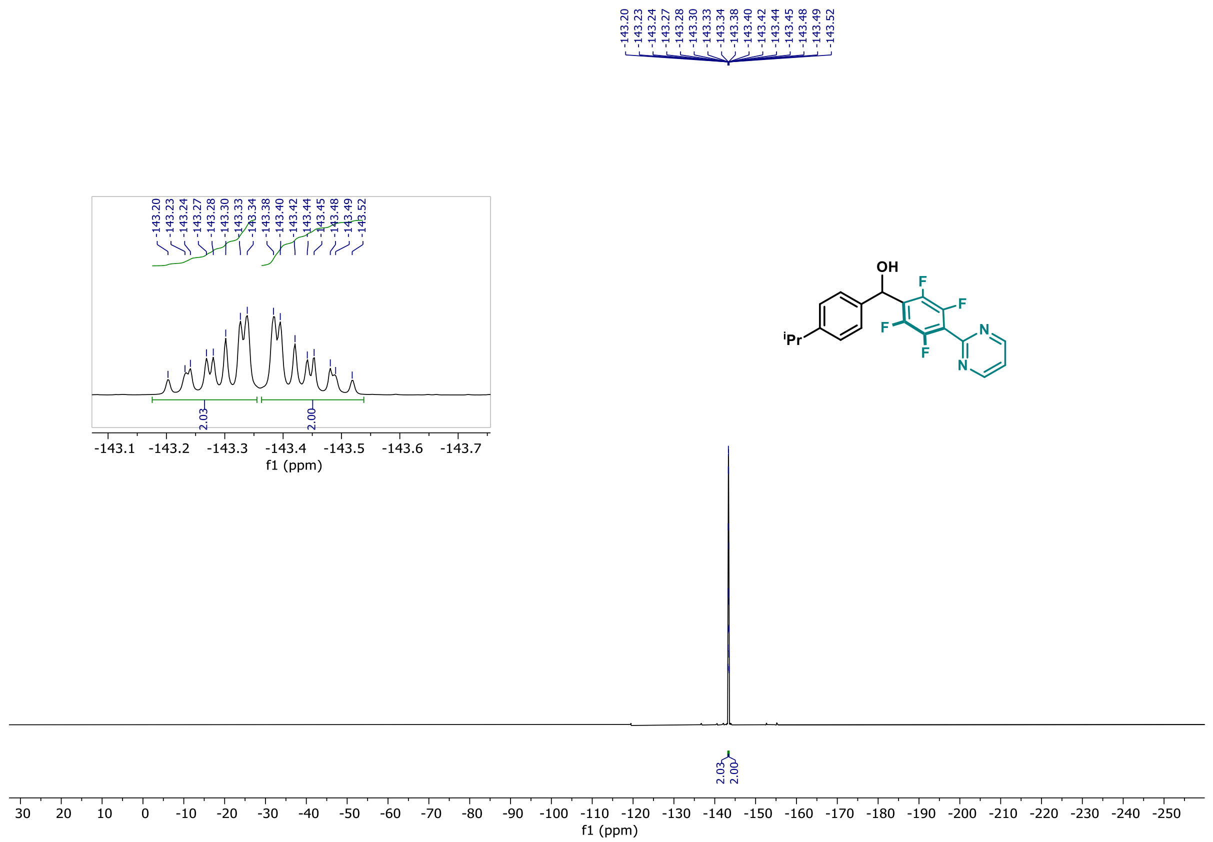

^{13}**C NMR** of **4d** (75 MHz, $CDCl_3$, 298K)

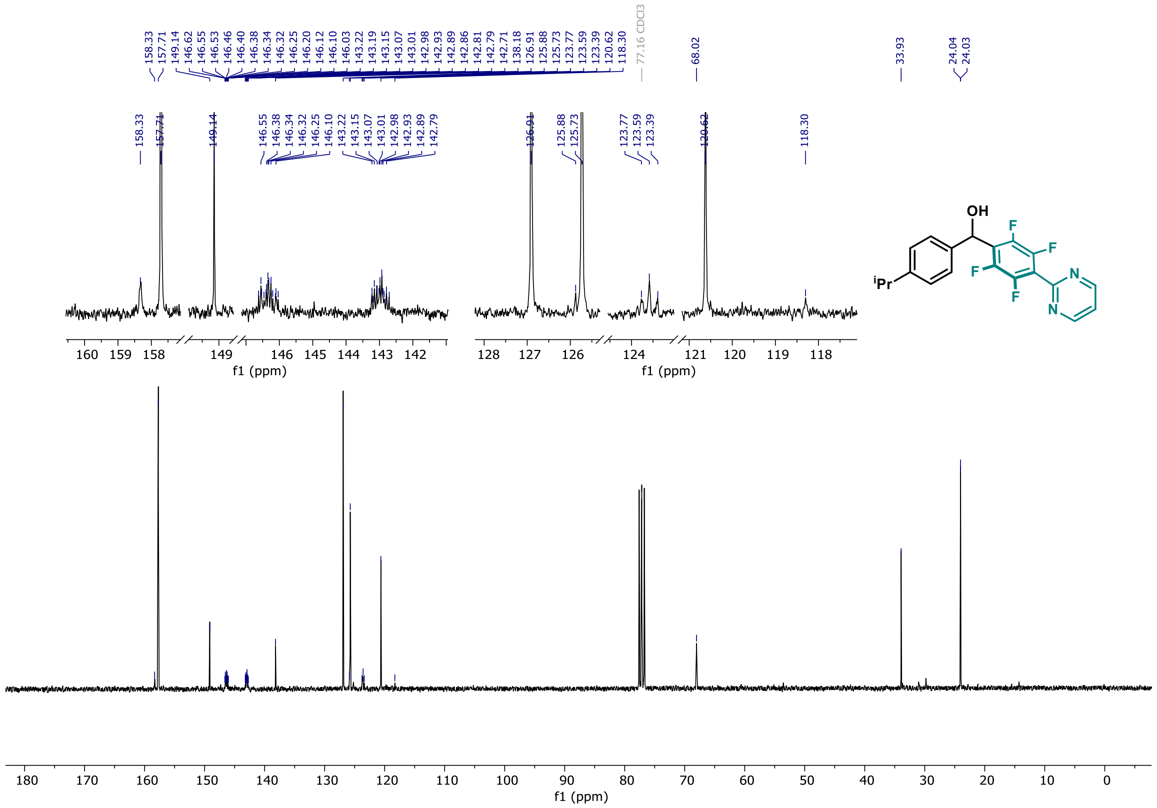

Crude ^{1}H NMR for 4e•TMS before deprotection (300 MHz, $CDCl_3$, 298K)

Crude ^{1}H NMR yield of **4e**•TMS before TMS deprotection using trichloroethene as internal standard.

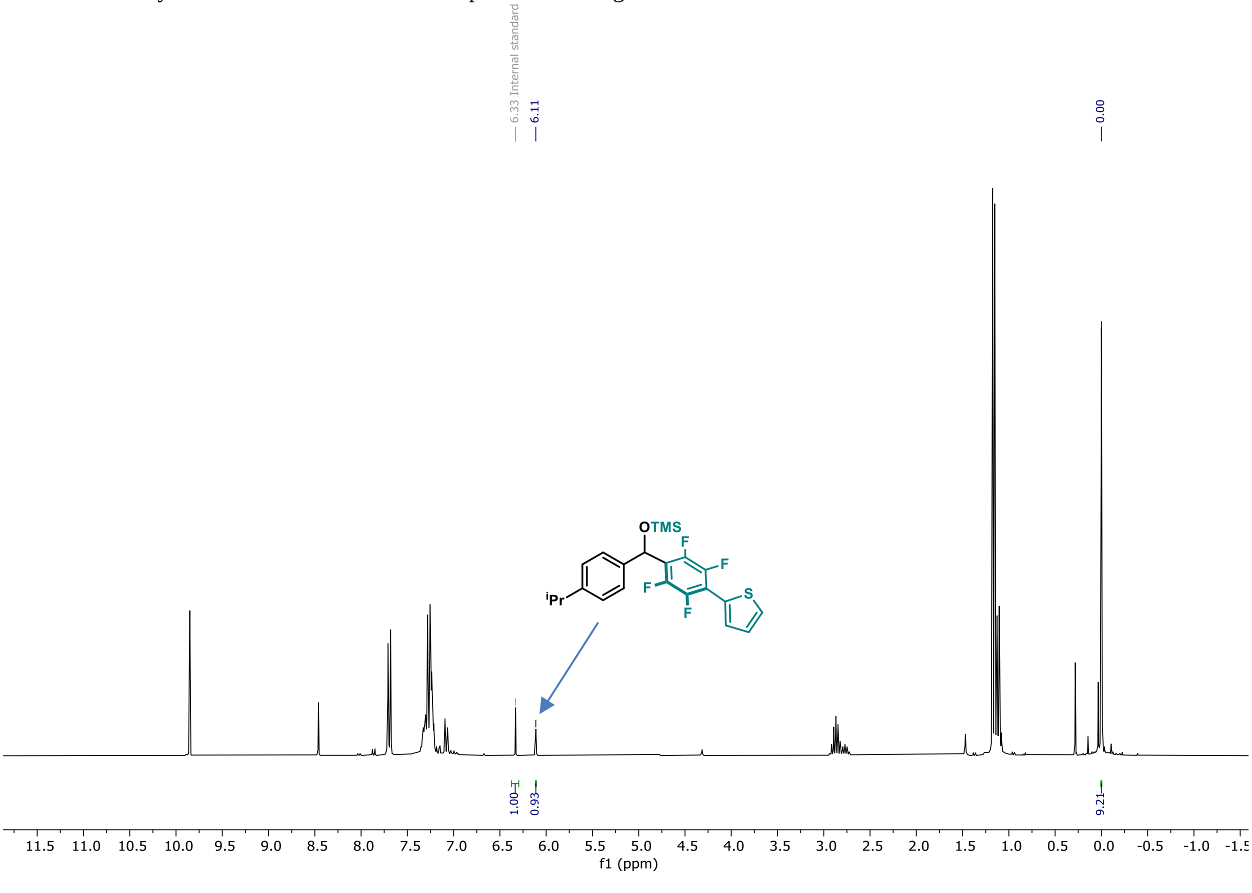

^{1}**H NMR** of **4e** (300 MHz, $CDCl_3$, 298K)

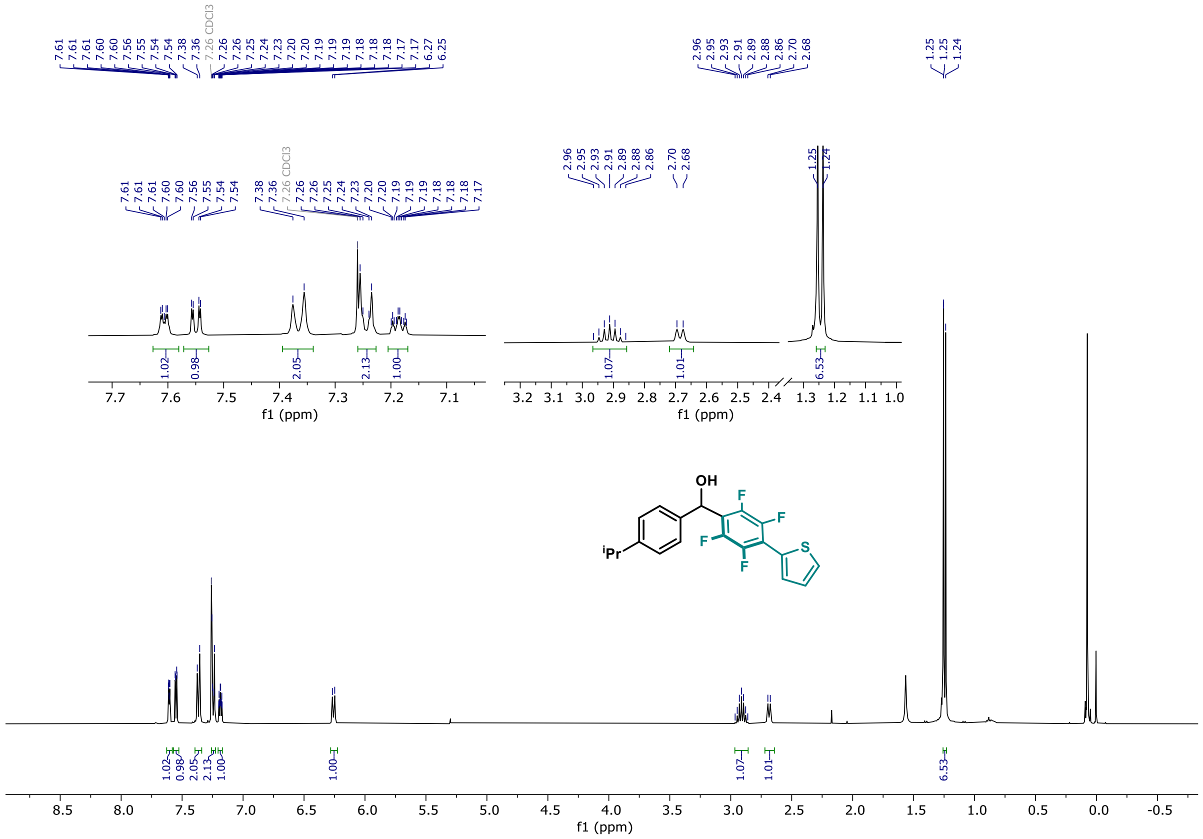

^{19}**F NMR** of **4e** (282 MHz, $CDCl_3$, 298K)

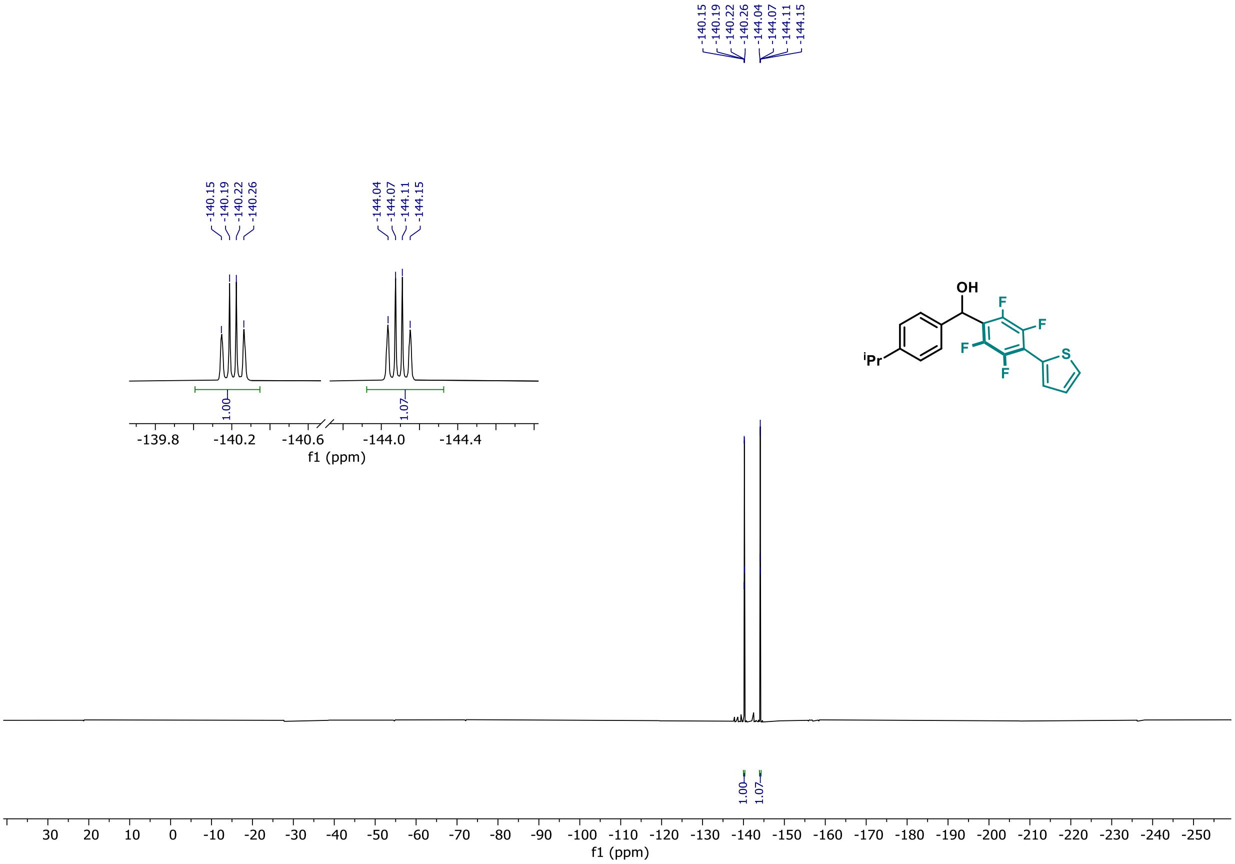

^{13}C NMR of **4e** (101 MHz, $CDCl_3$, 298K)

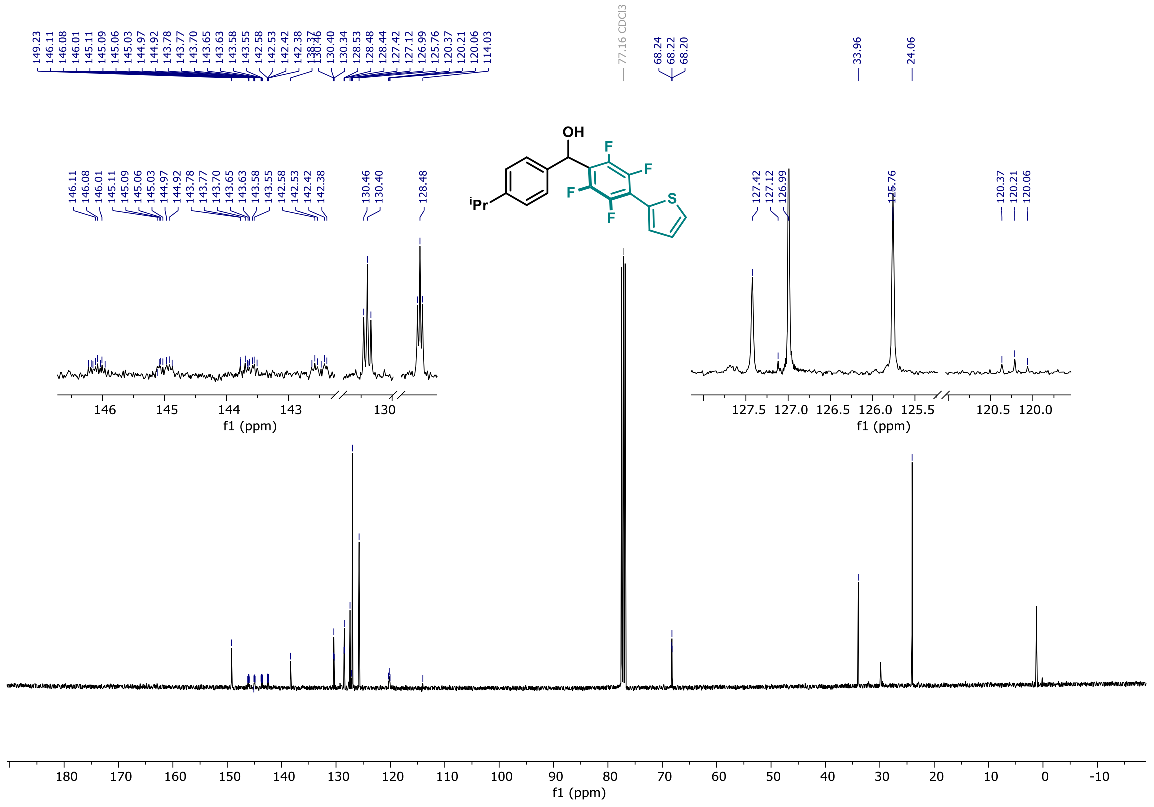

Crude ^{1}H NMR of 4f•TMS (300 MHz, $CDCl_3$, 298K)

Crude ^{1}H NMR yield of **4f**•TMS before TMS deprotection using trichloroethene as internal standard.

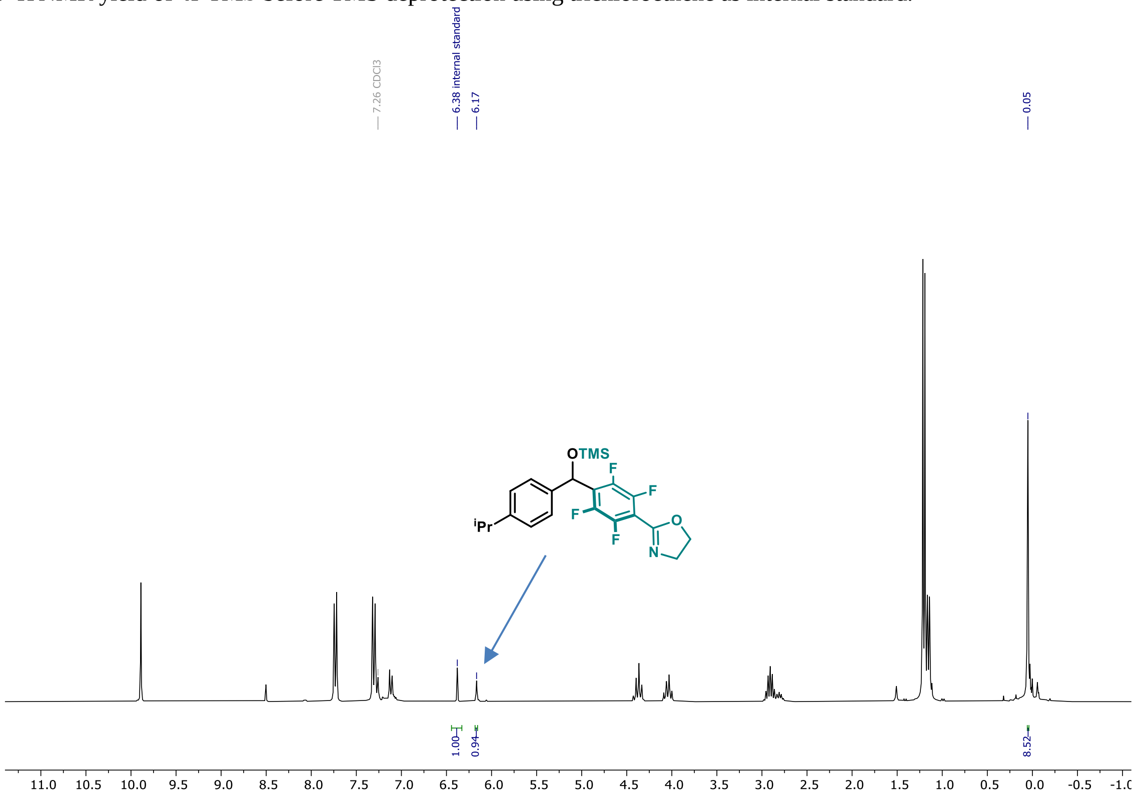

^{1}H NMR of **4f** (300 MHz, $CDCl_3$, 298K)

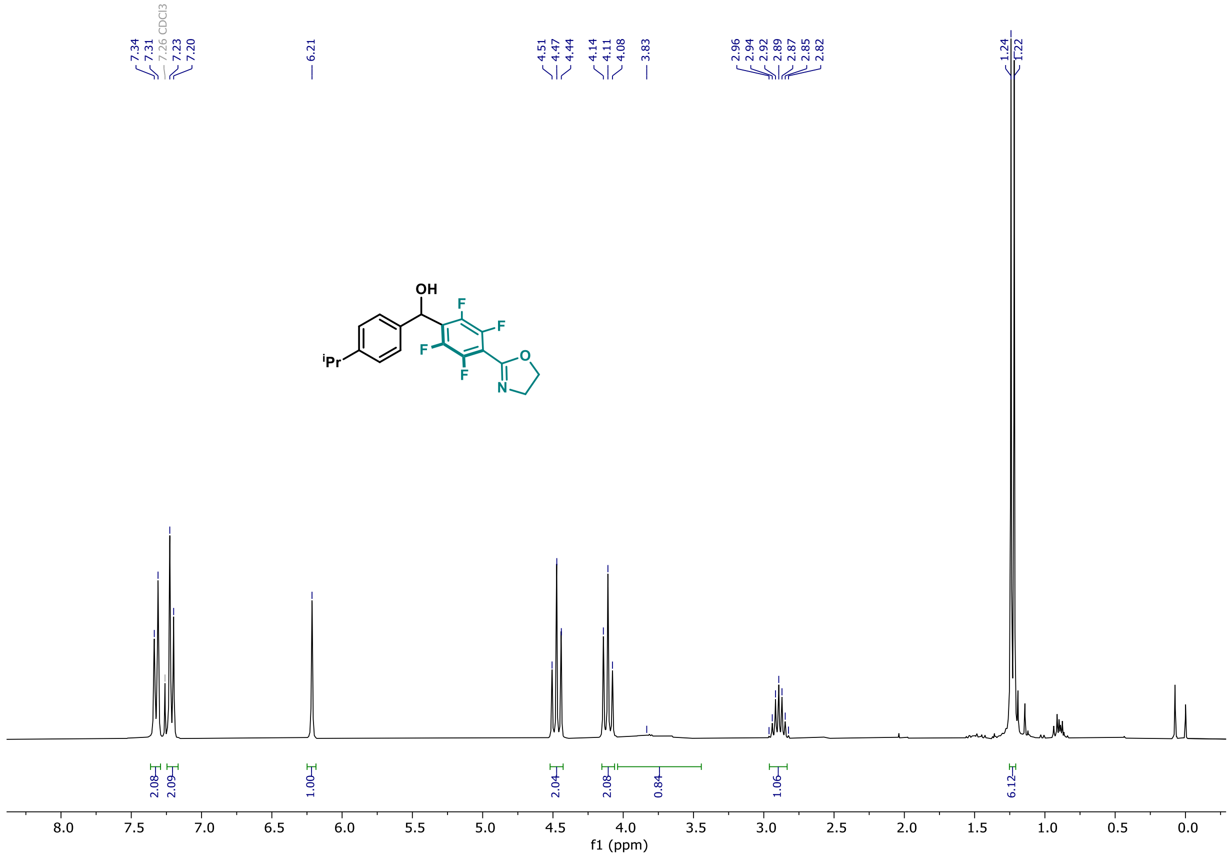

^{19}F NMR of **4f** (282 MHz, $CDCl_3$, 298K)

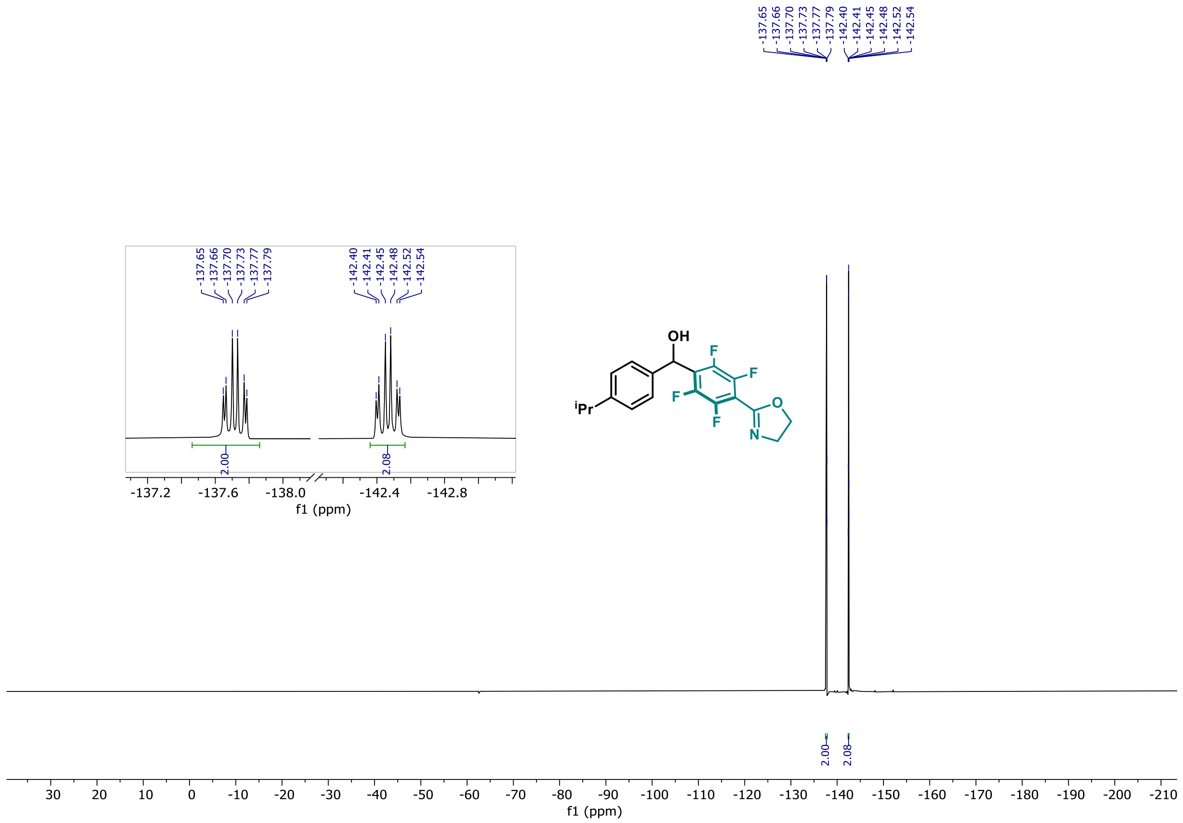

^{13}C NMR of **4f** (75 MHz, $CDCl_3$, 298K)

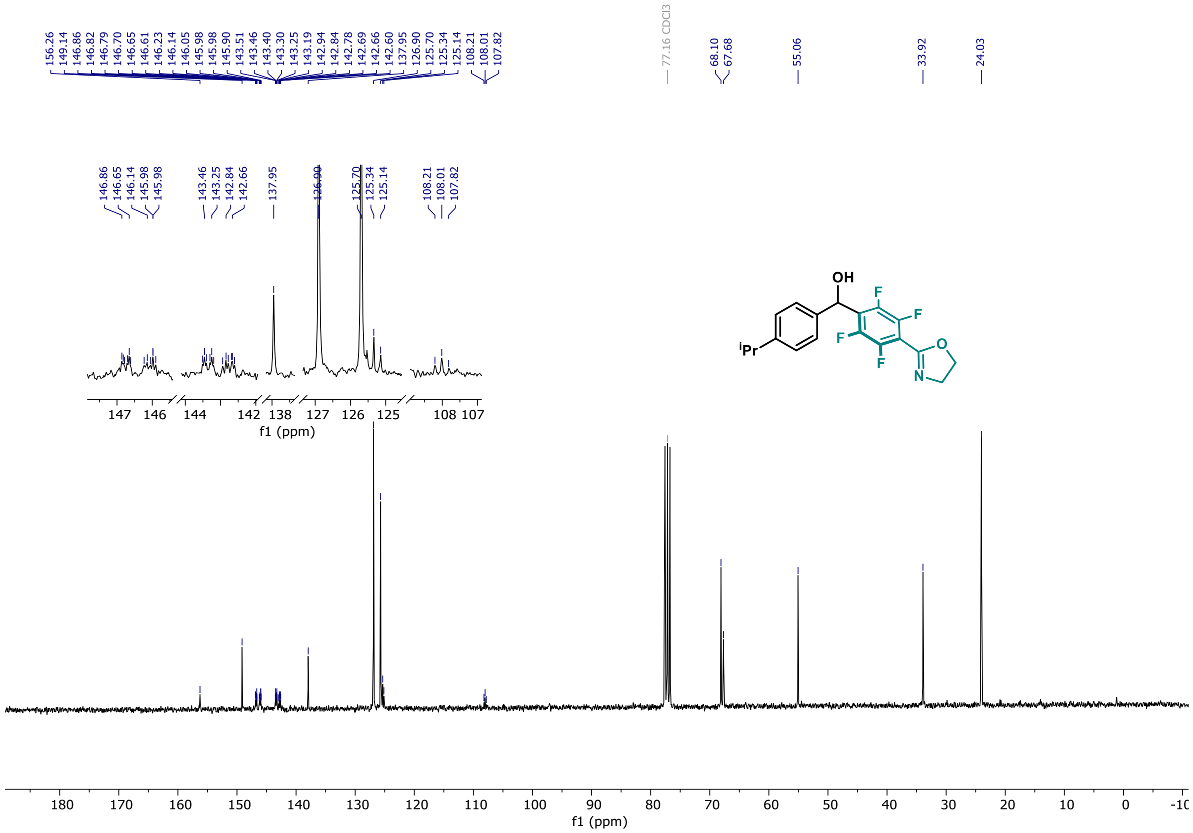

Crude ^{1}H NMR of 4g (300 MHz, $CDCl_3$, 298K)

Crude ^{1}H NMR yield of **4g** using trichloroethene as internal standard

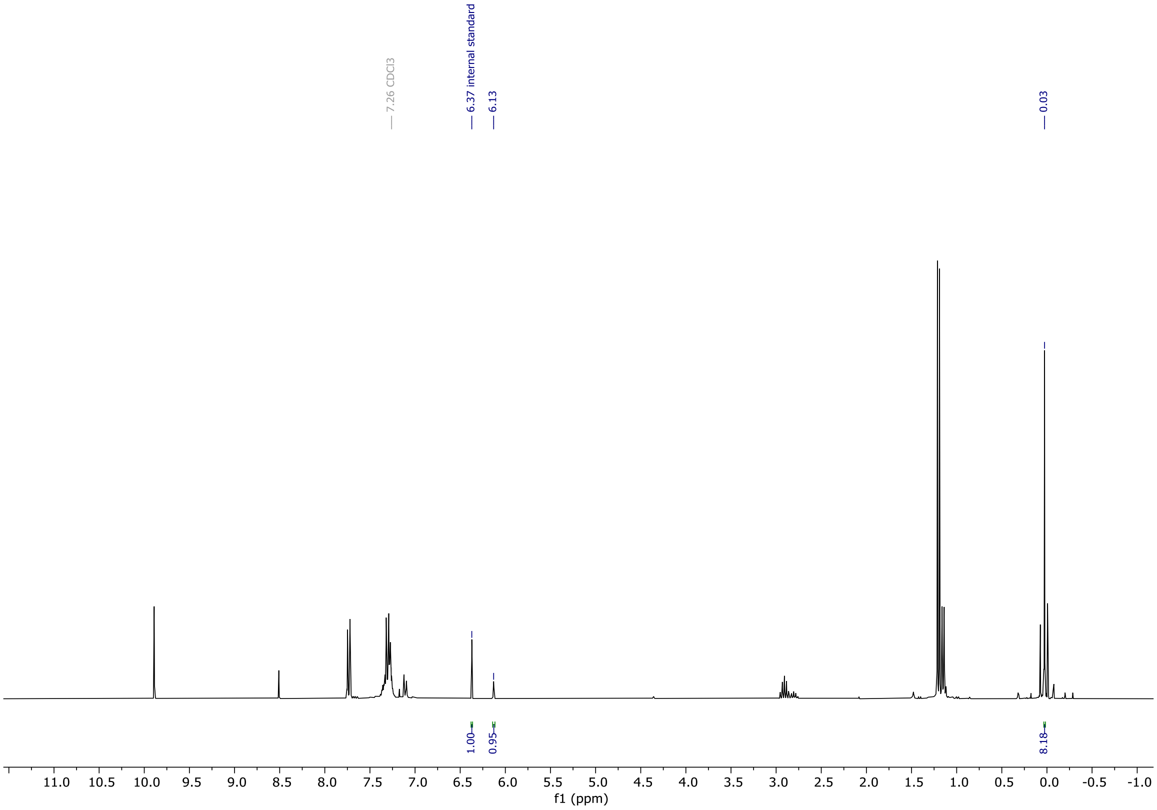

^{1}H NMR of **4g** (400 MHz, $CDCl_3$, 298K)

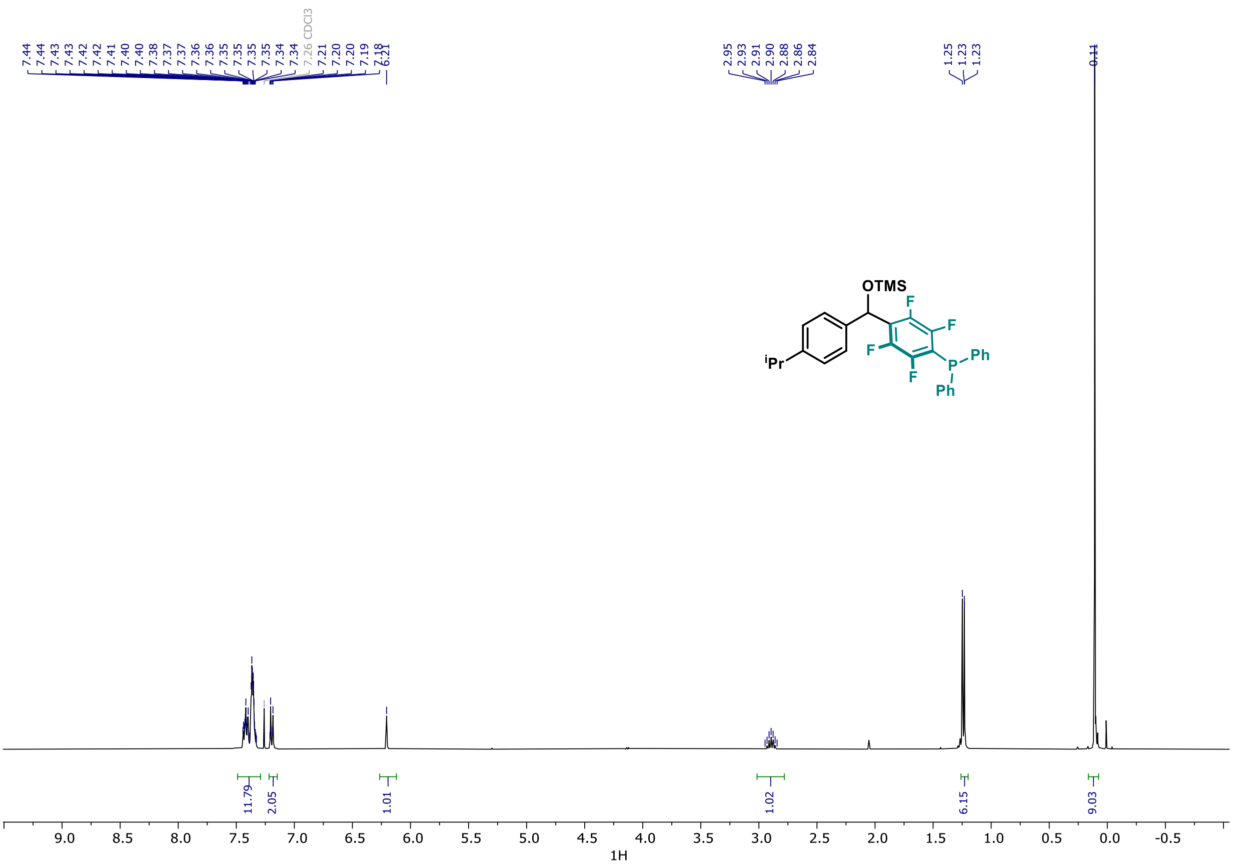

^{19}F NMR of **4g** (282 MHz, $CDCl_3$, 298K)

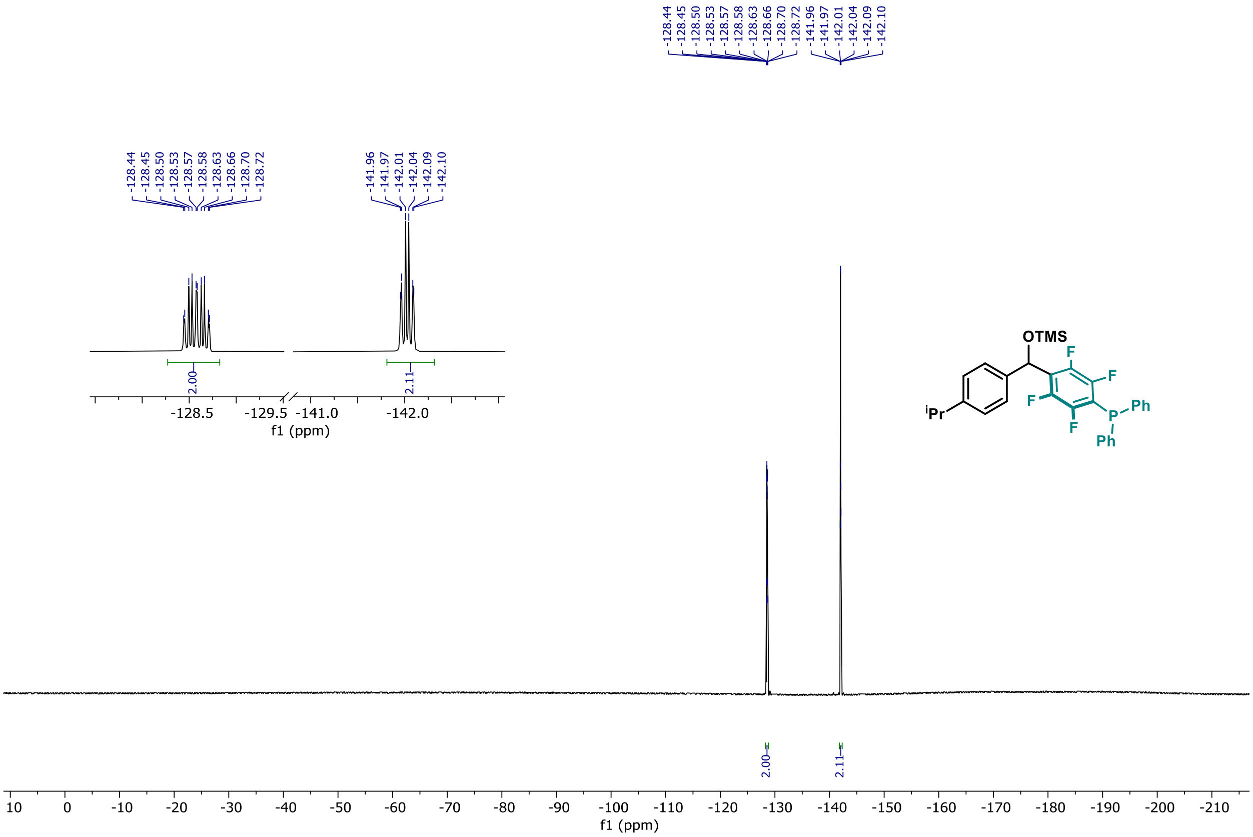

^{31}P NMR of **4g** (122 MHz, $CDCl_3$, 298K)

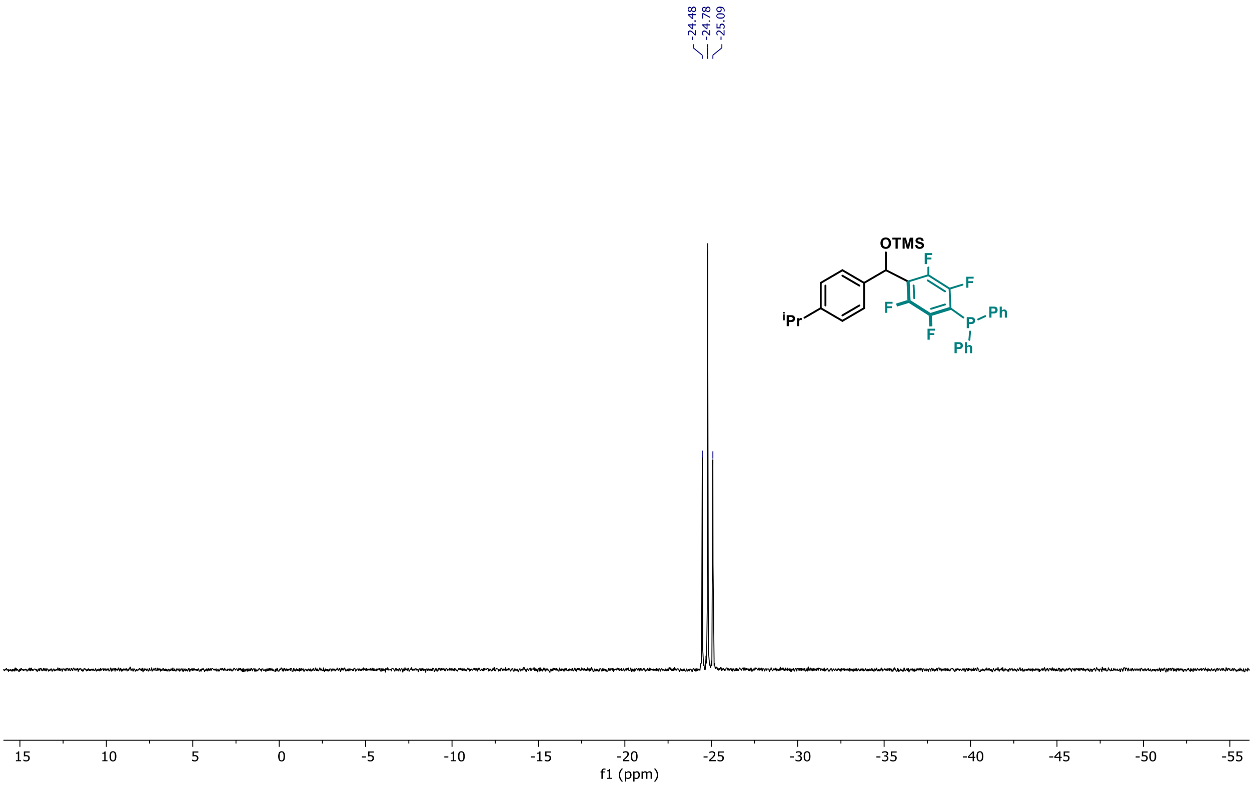

^{29}Si NMR of **4g** (60 MHz, $CDCl_3$, 298K)

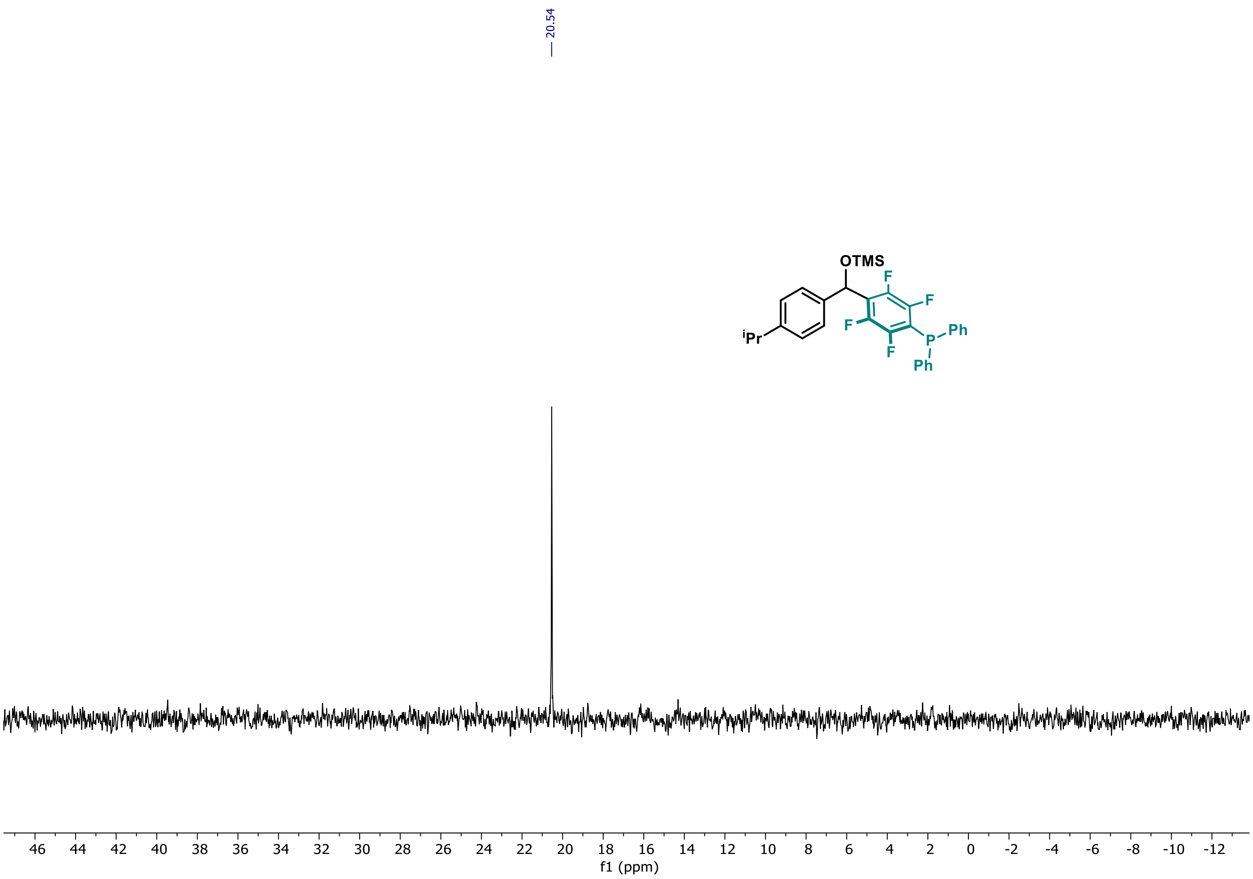

^{13}**C NMR** of **4g** (75 MHz, $CDCl_3$, 298K)

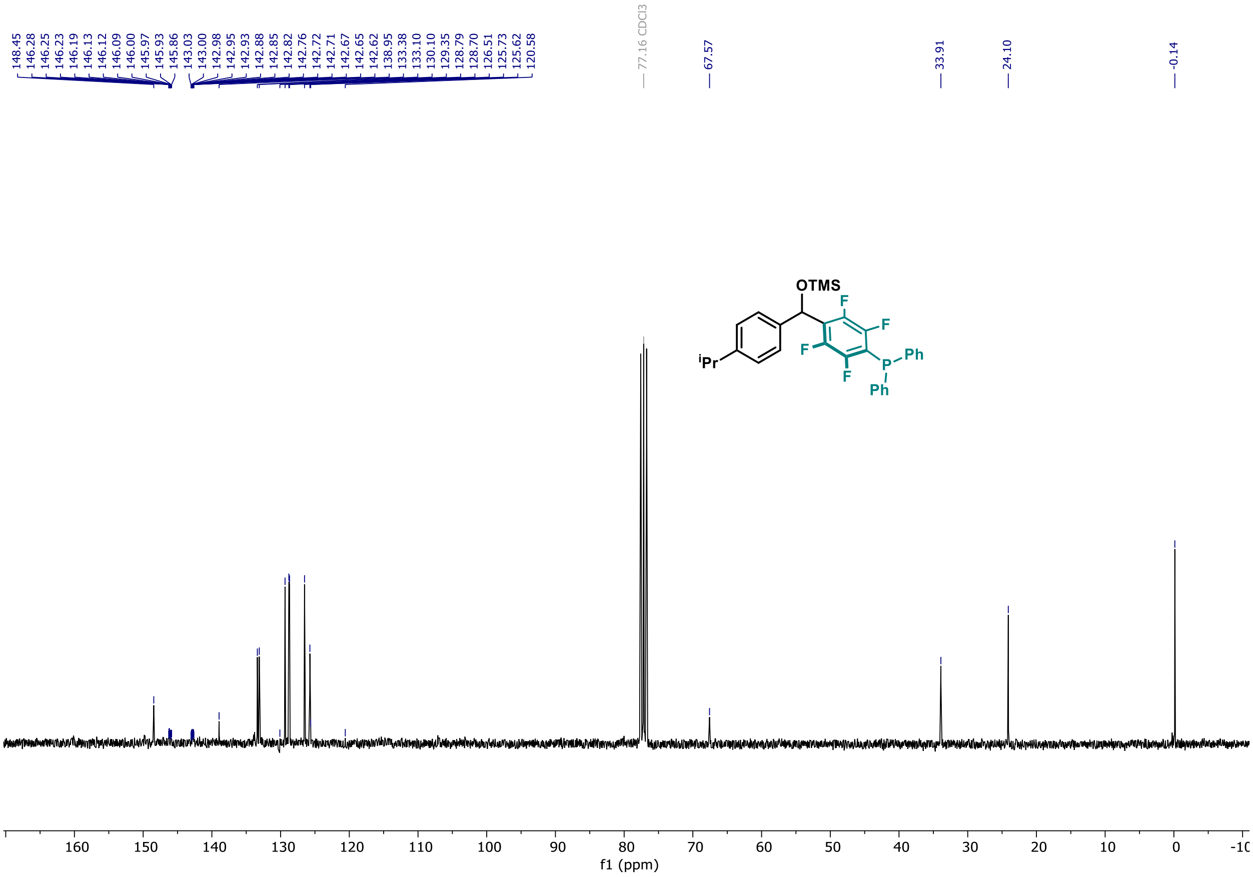

^{1}**H NMR** of **4g'** (300 MHz, $CDCl_3$, 298K)

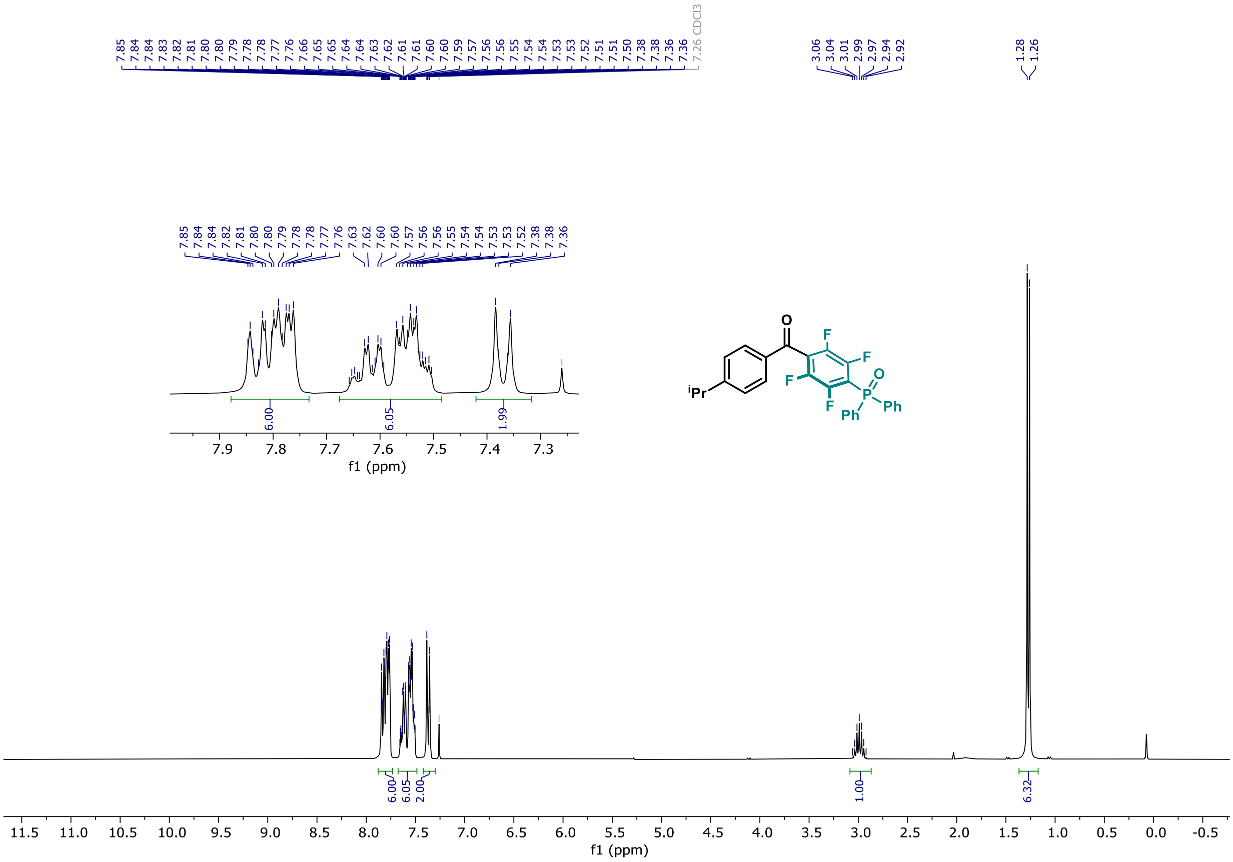

^{19}F NMR of **4g'** (282 MHz, $CDCl_3$, 298K)

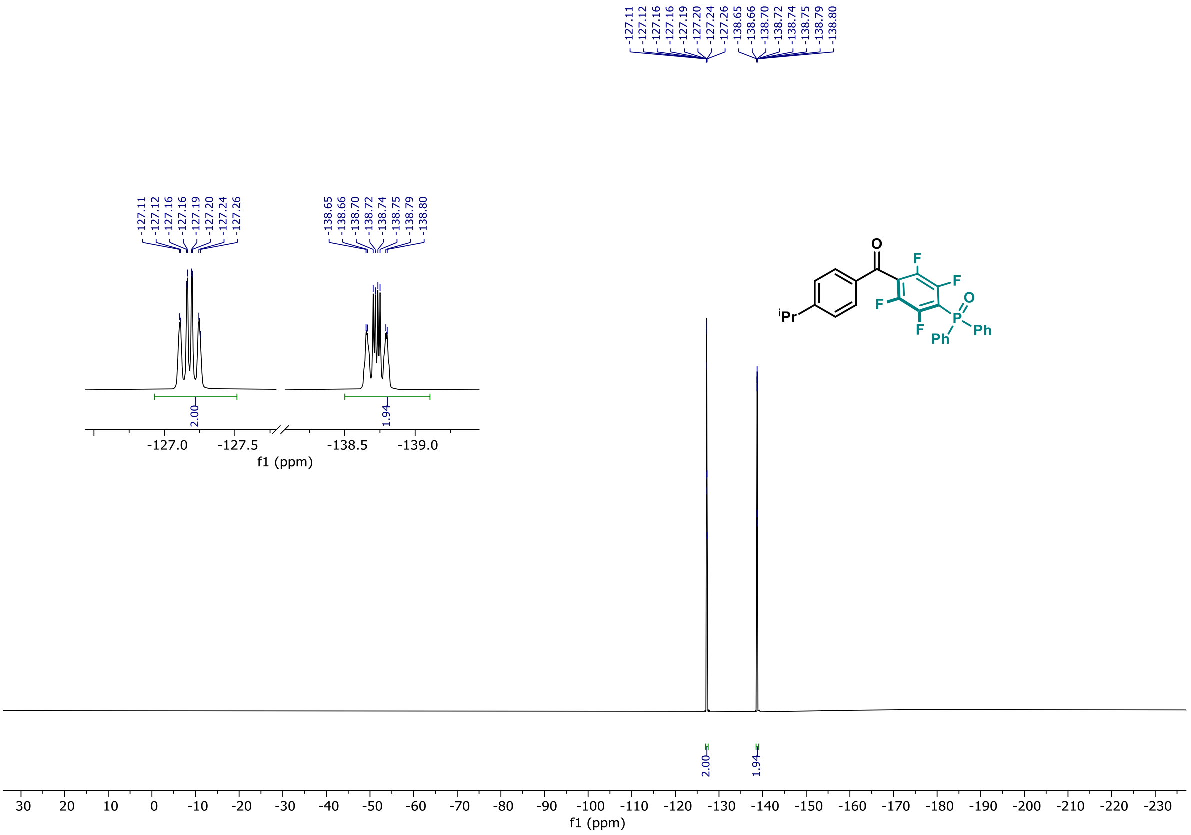

^{31}P NMR of **4g'** (122 MHz, $CDCl_3$, 298K)

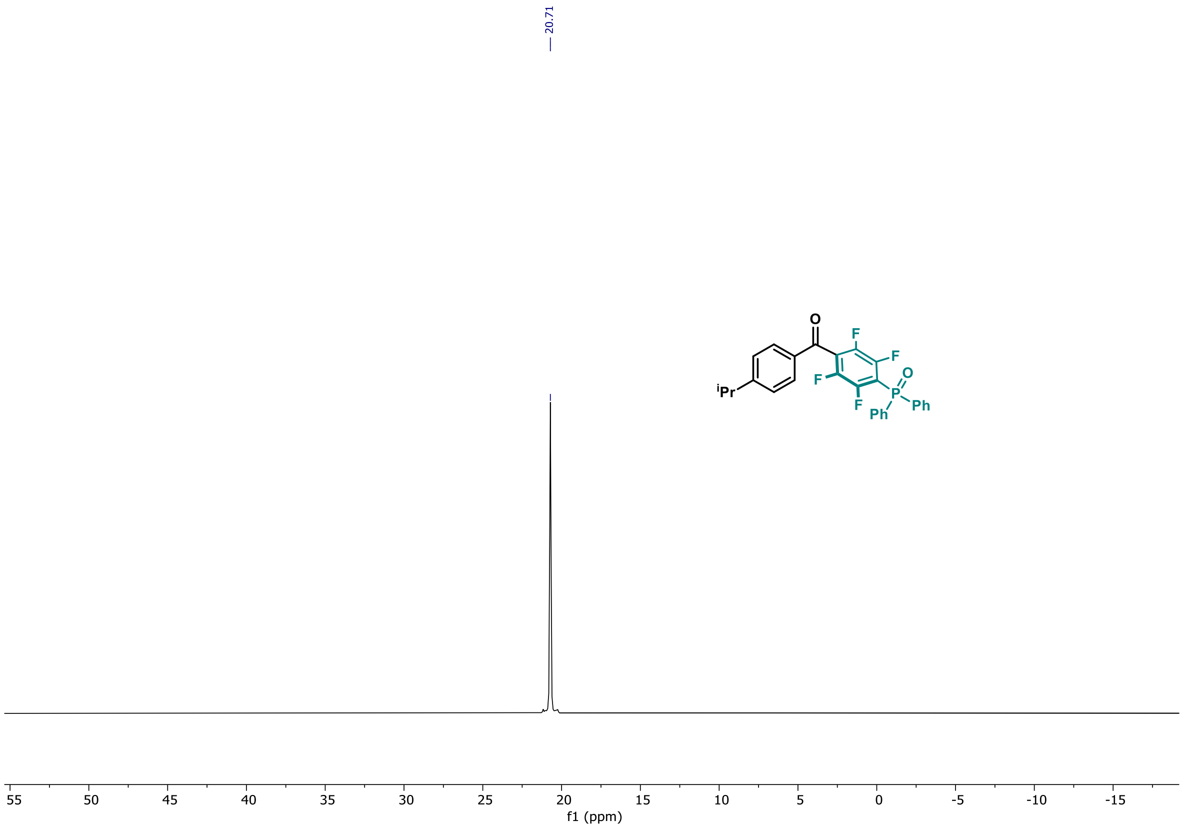

^{13}**C NMR** of **4g'** (75 MHz, $CDCl_3$, 298K)

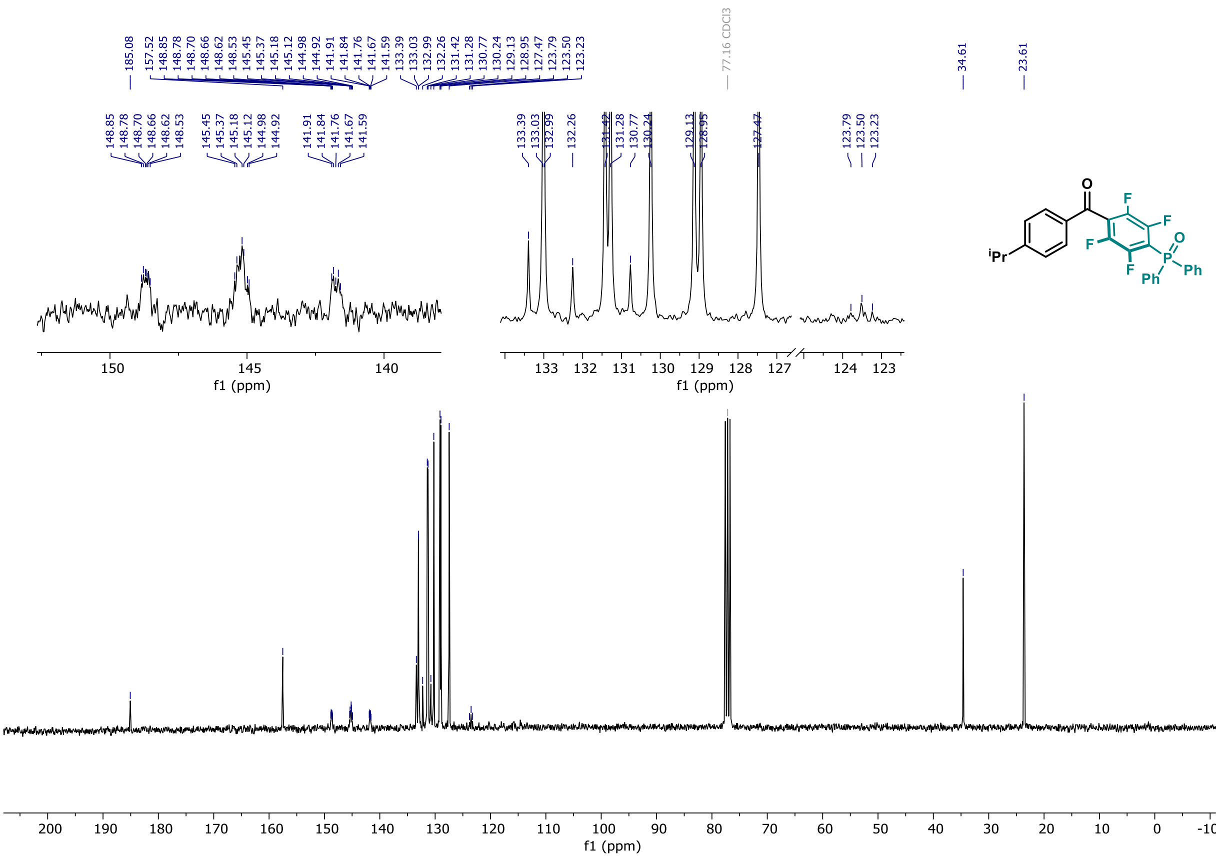

^{1}**H NMR** of **4h** (300 MHz, $CDCl_3$, 298K)

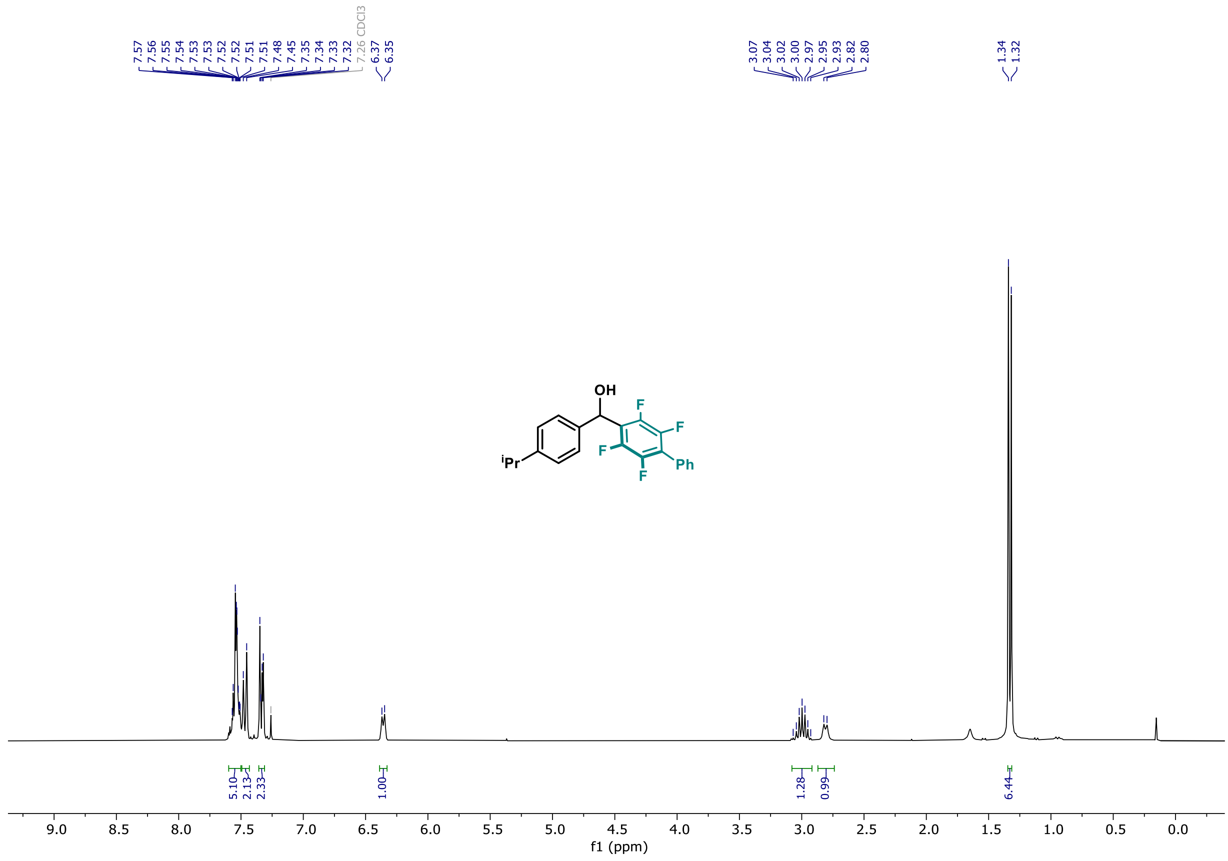

^{19}F NMR of **4h** (282 MHz, $CDCl_3$, 298K)

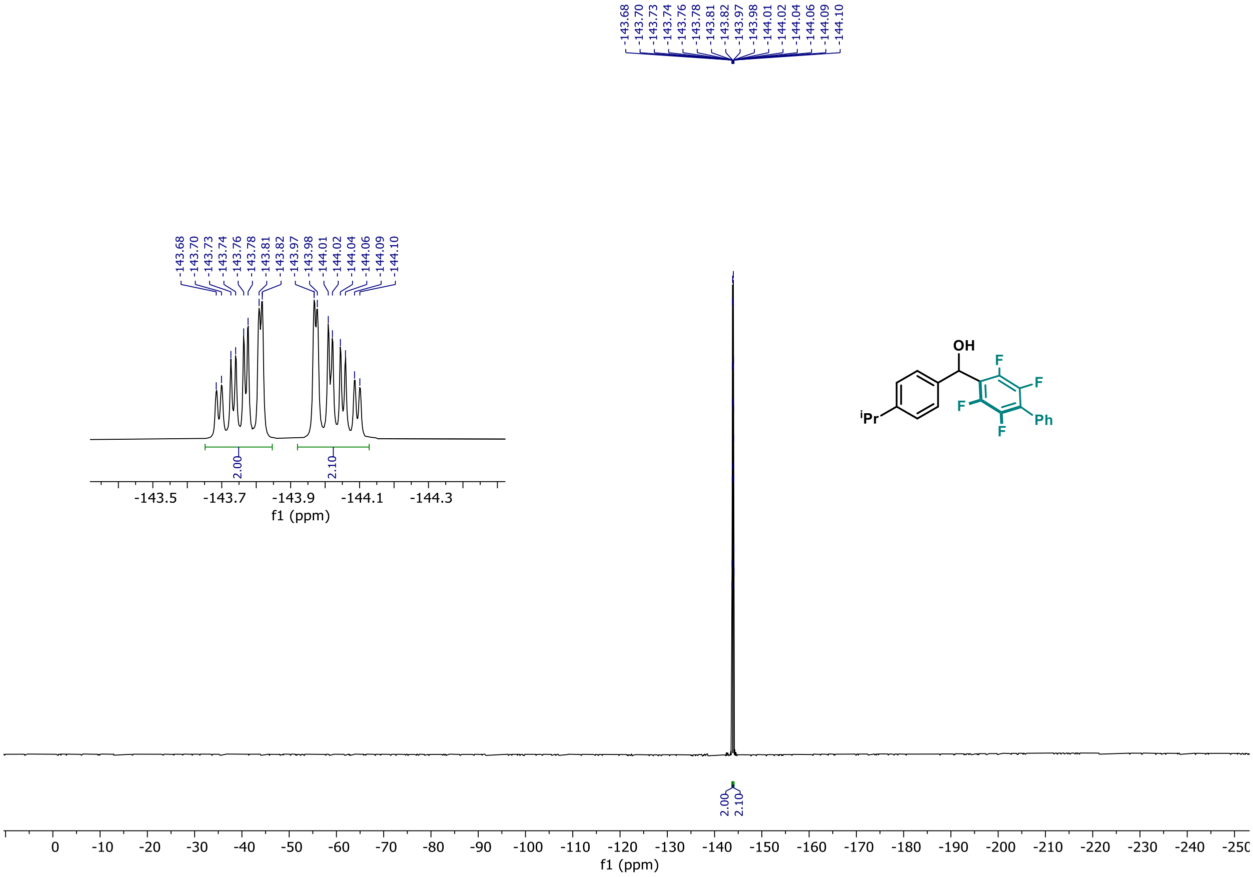

^{13}C NMR of **4h** (101 MHz, $CDCl_3$, 298K)

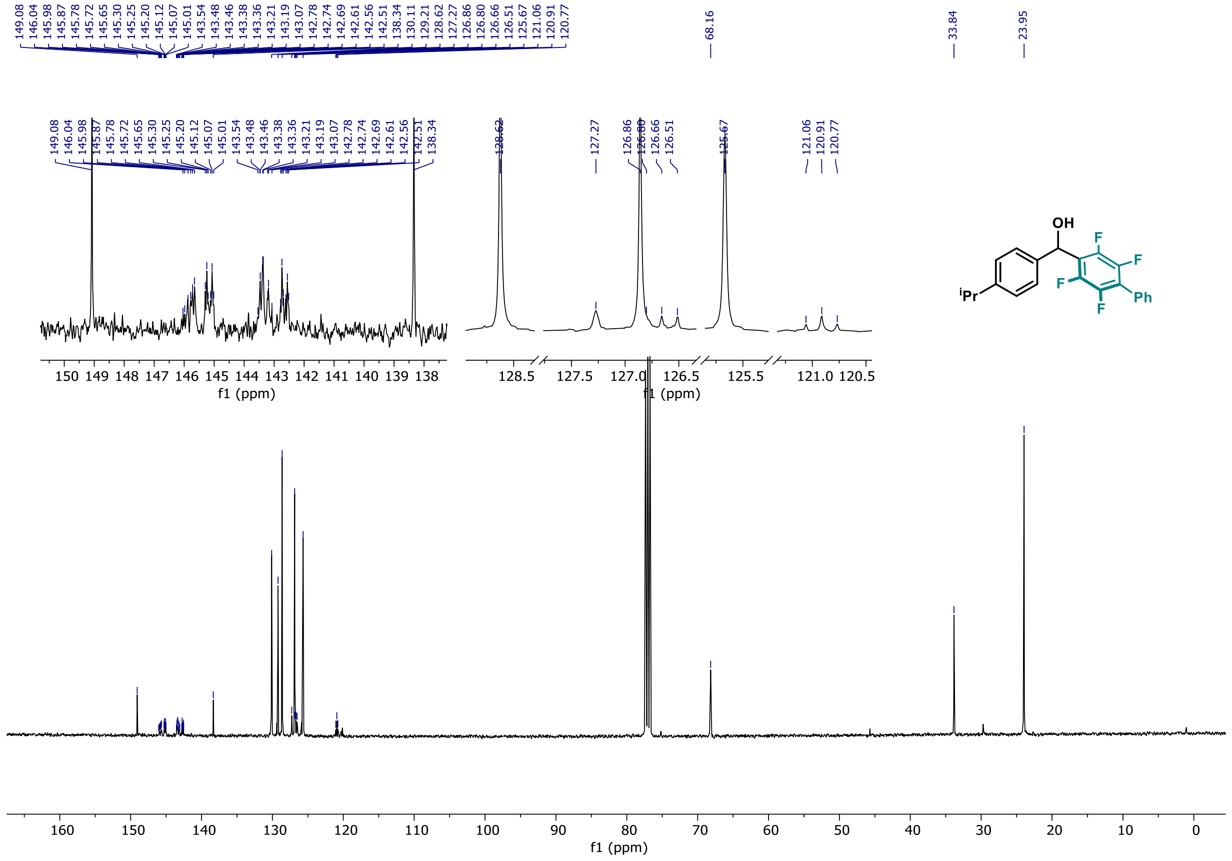

^{1}H NMR of 4i (300 MHz, $CDCl_3$, 298K)

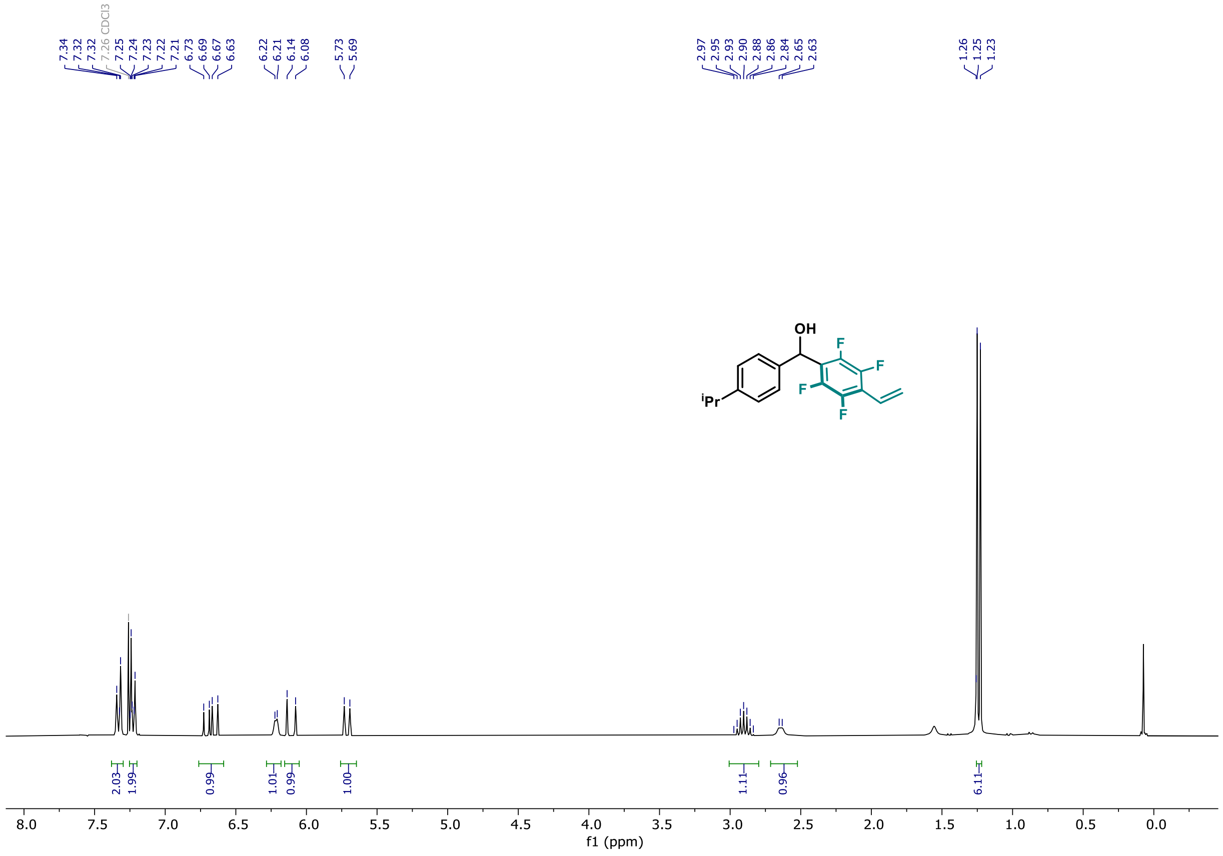

^{19}F NMR of 4i (282 MHz, $CDCl_3$, 298K)

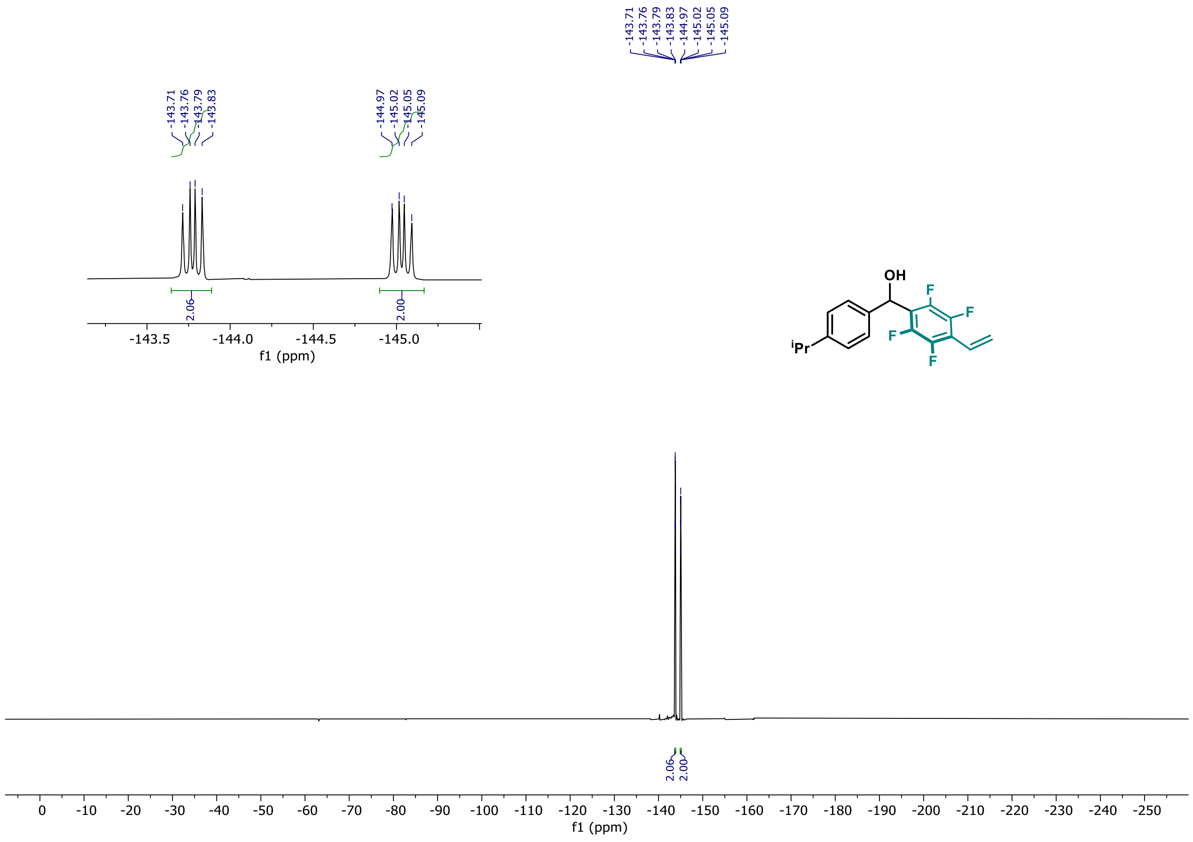

^{13}C NMR of **4i** (101 MHz, $CDCl_3$, 298K)

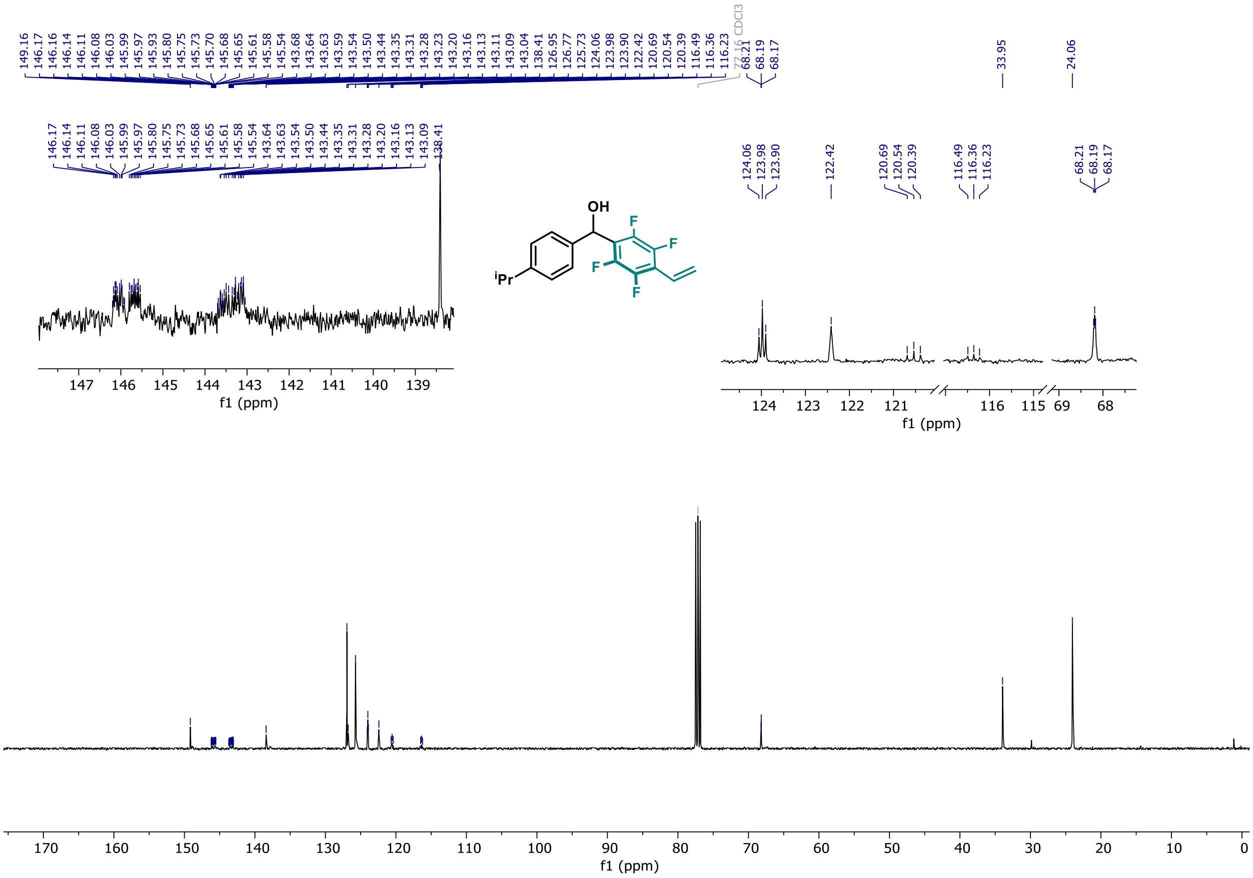

^{1}H NMR of **4j** (300 MHz, $CDCl_3$, 298K)

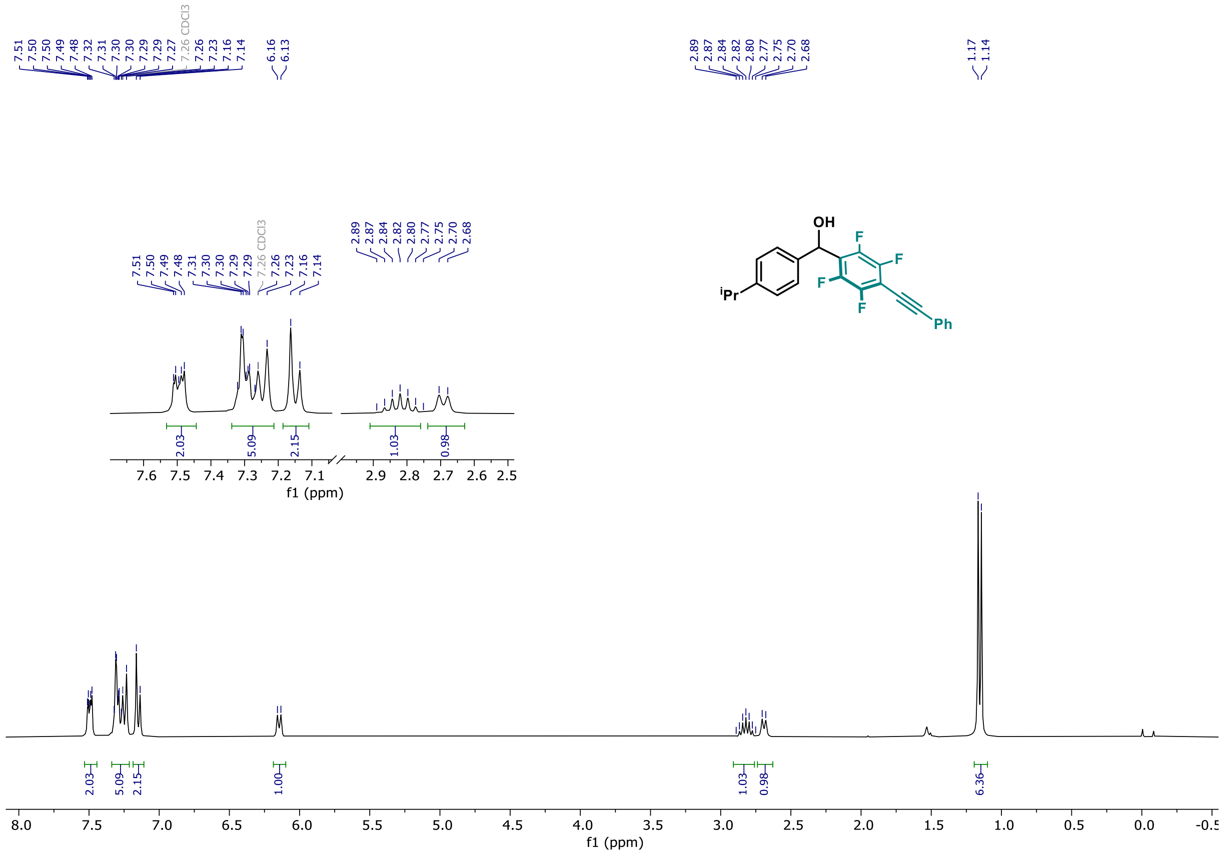

^{19}F NMR of **4j** (282 MHz, $CDCl_3$, 298K)

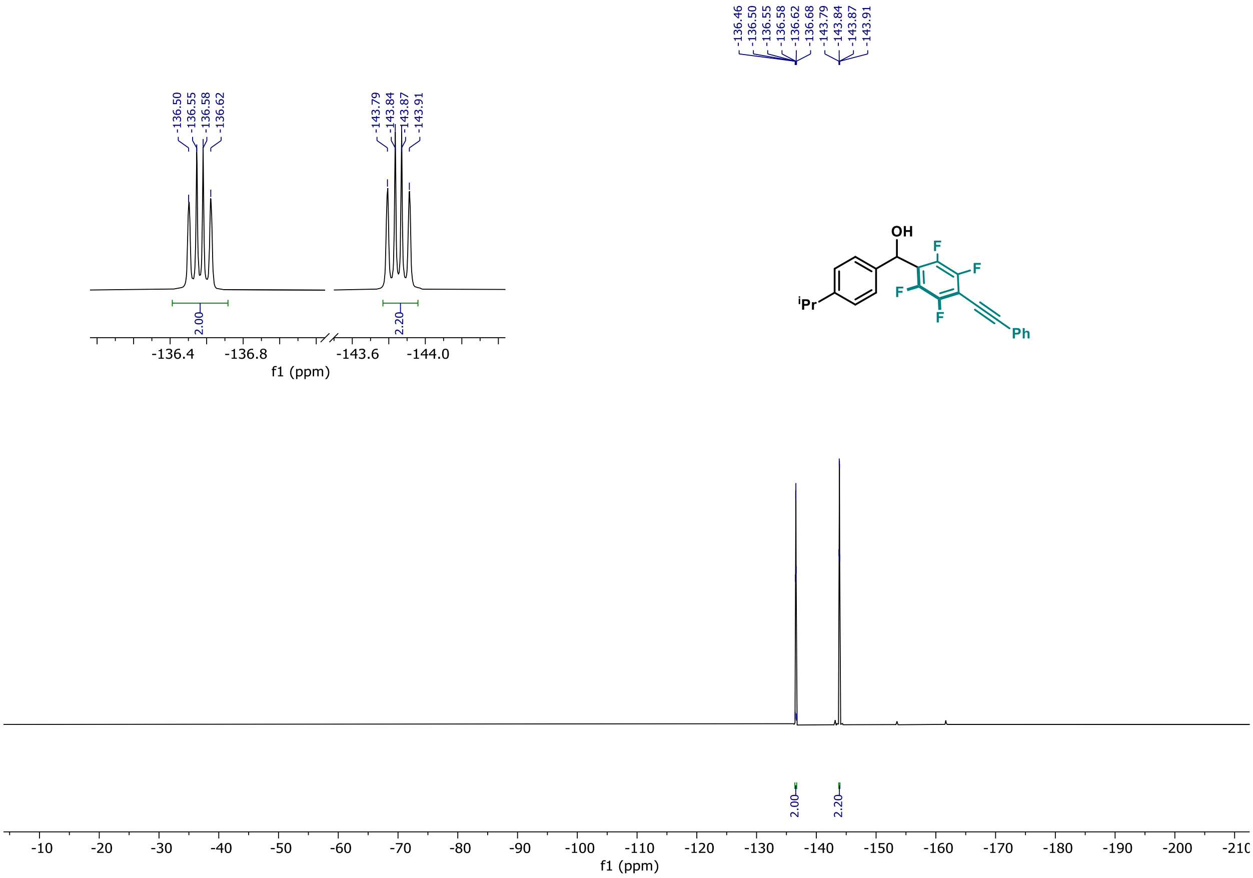

^{13}C NMR of **4j** (75 MHz, $CDCl_3$, 298K)

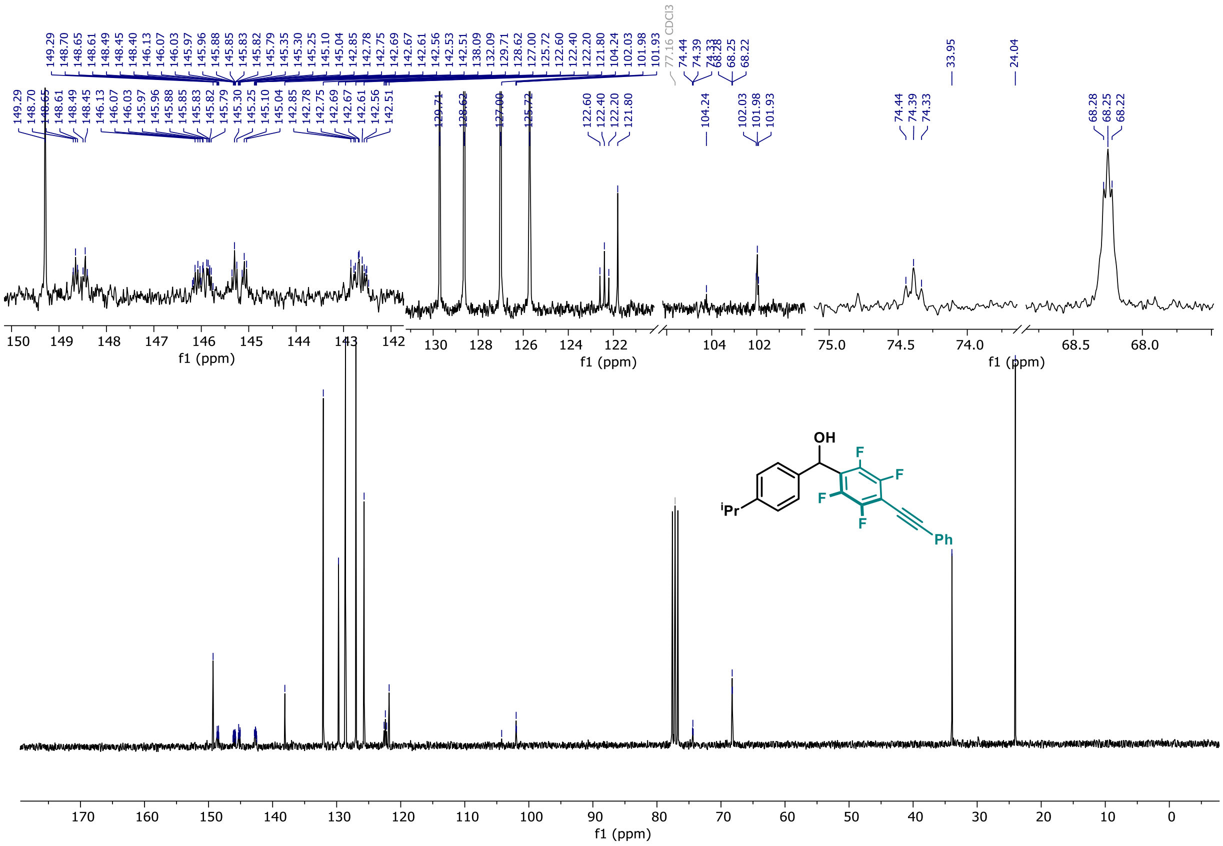

^{1}H NMR of **4k** (300 MHz, $CDCl_3$, 298K)

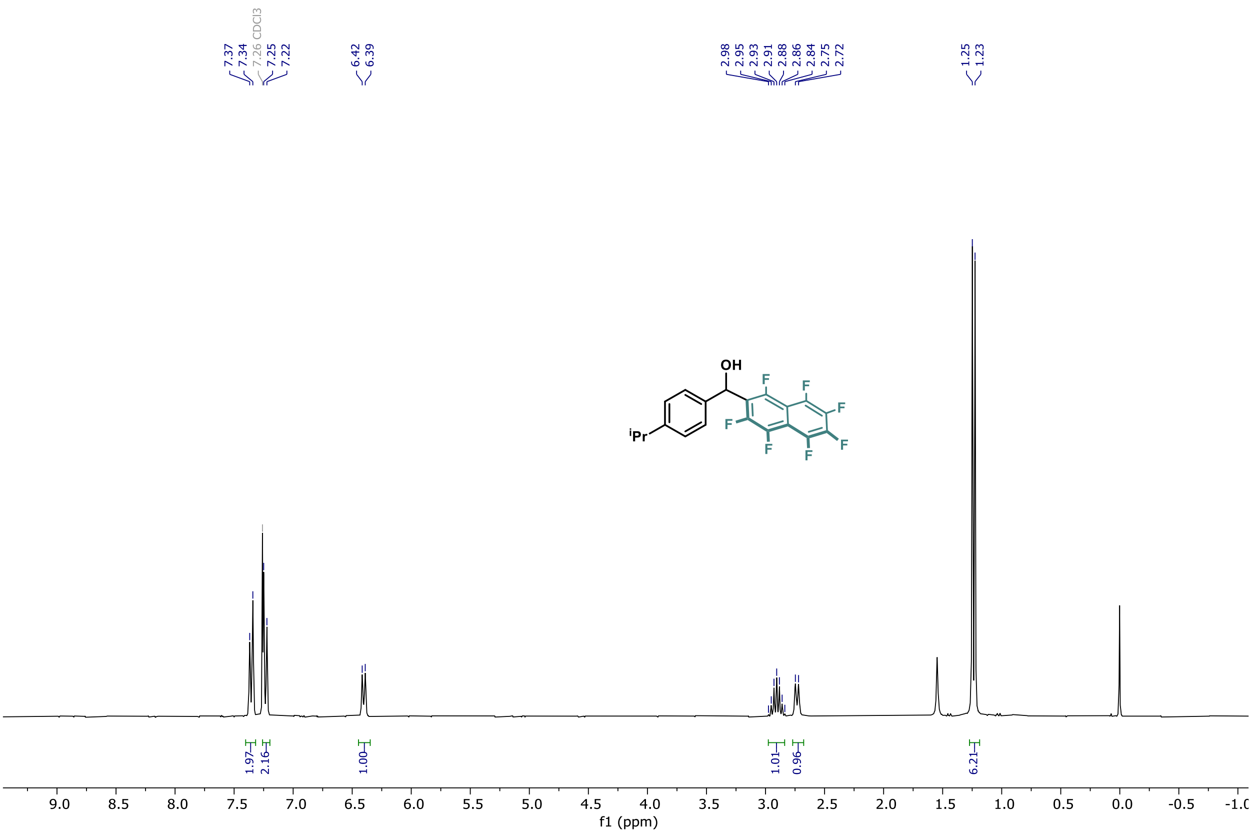

^{19}F NMR of **4k** (282 MHz, $CDCl_3$, 298K)

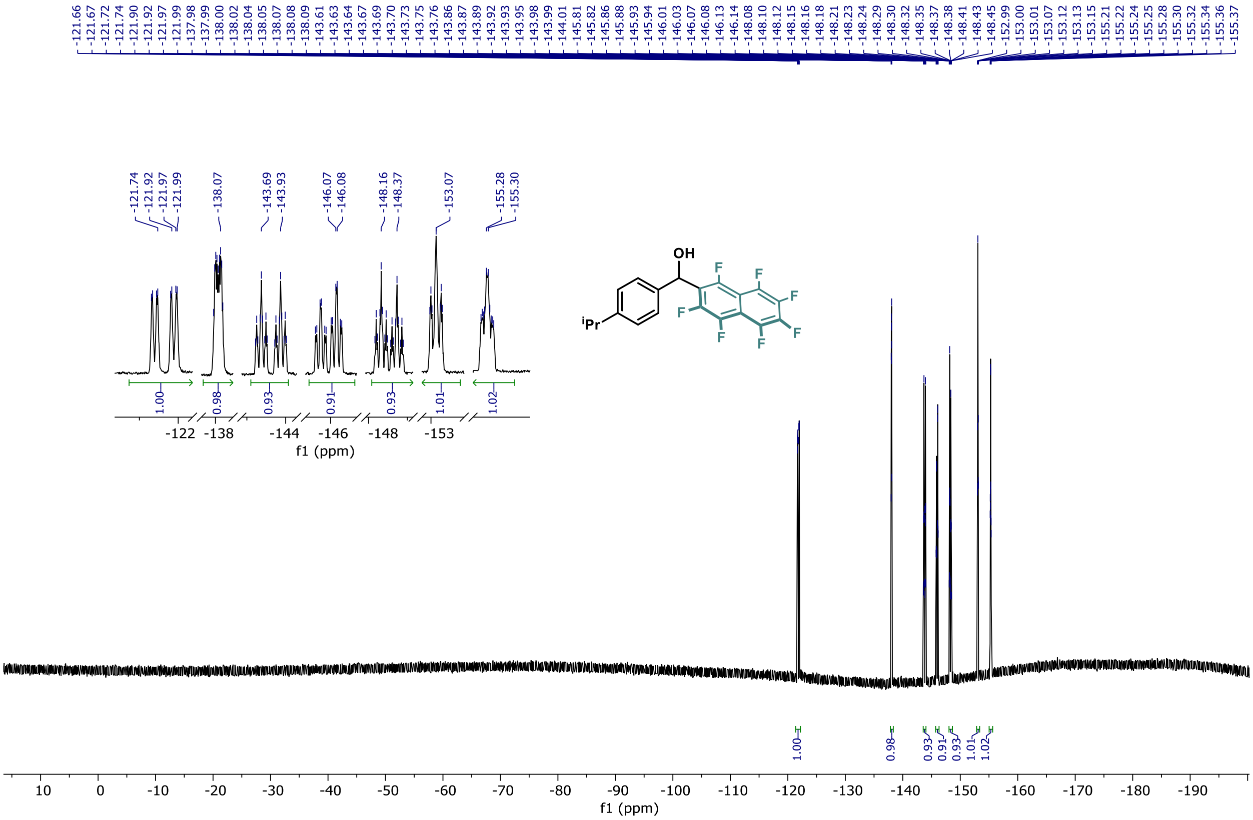

^{13}C NMR of **4k** (101 MHz, $CDCl_3$, 298K)

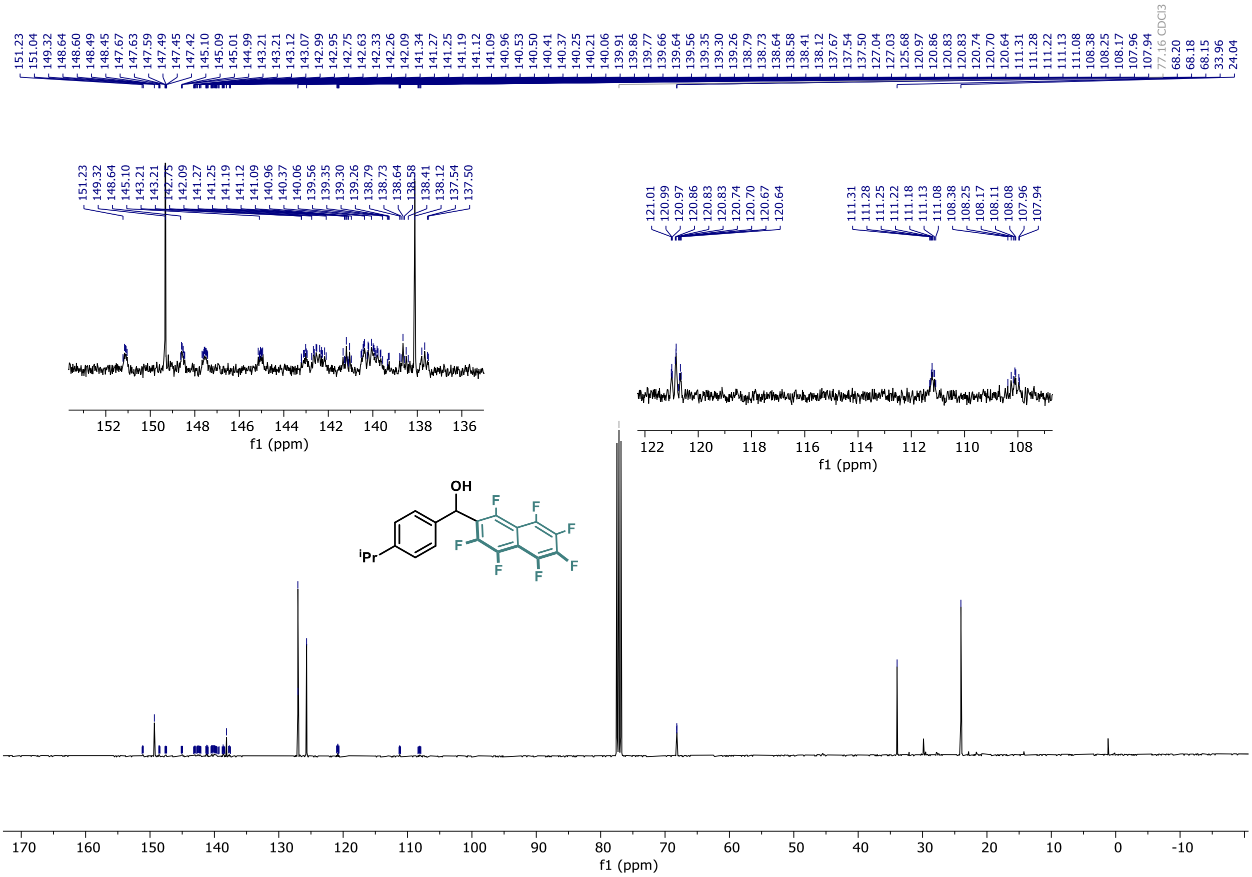

^{1}H NMR of **4l** (282 MHz, $CDCl_3$, 298K)

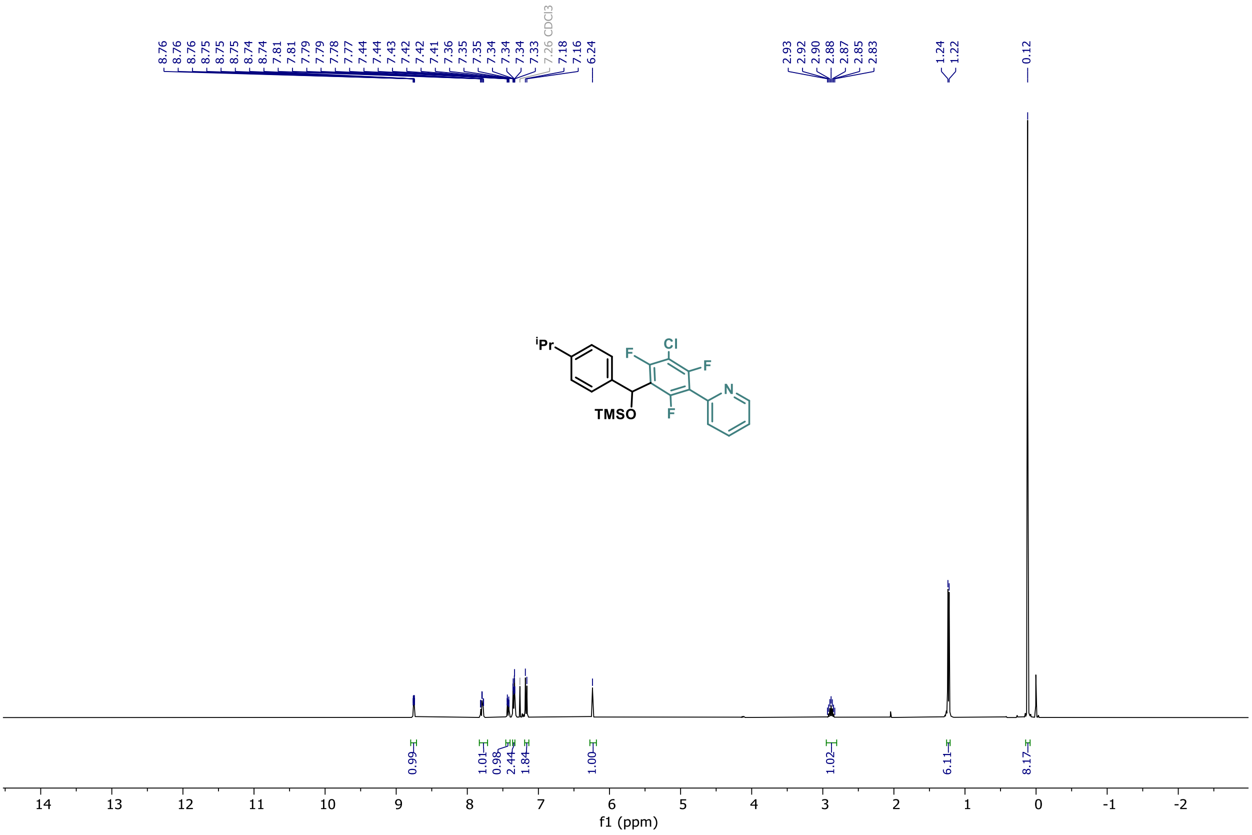

^{19}F NMR of **4l** (282 MHz, $CDCl_3$, 298K)

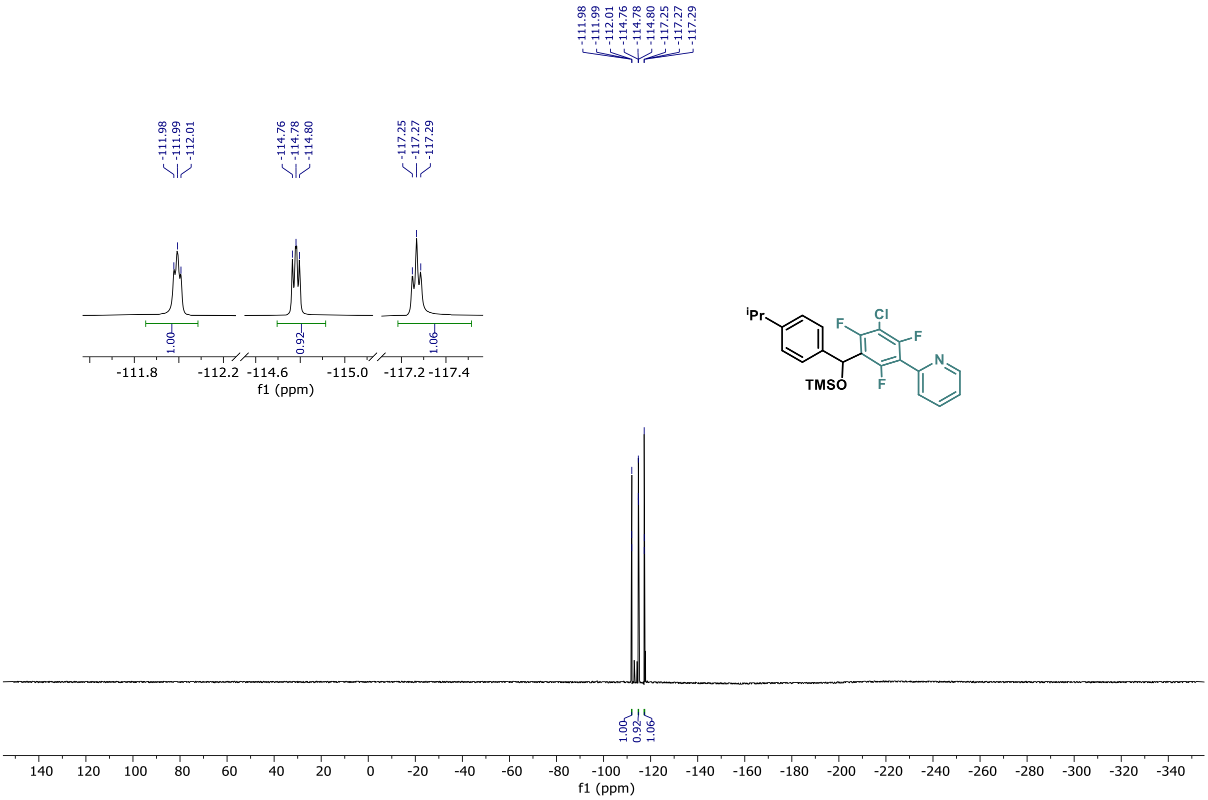

^{29}Si NMR of **4l** (60 MHz, $CDCl_3$, 298K)

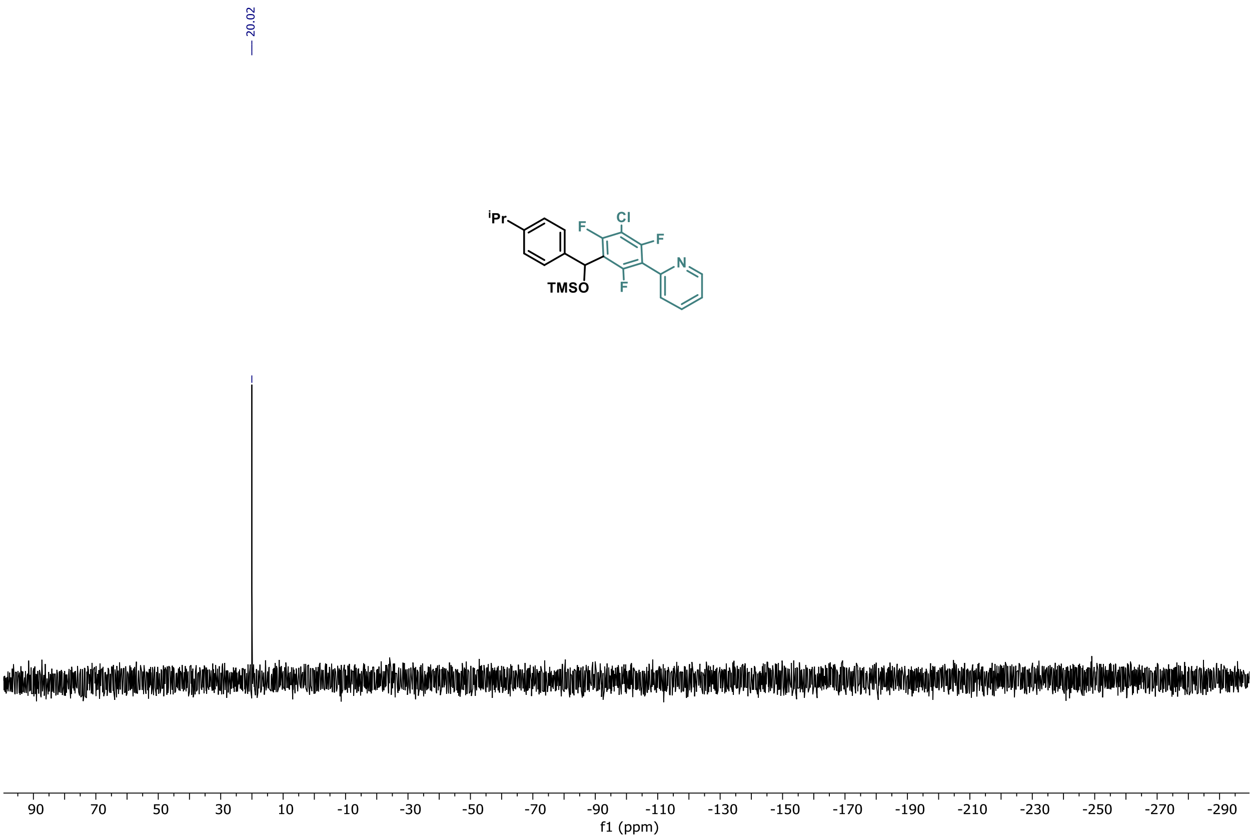

^{13}C NMR of **4l** (101 MHz, $CDCl_3$, 298K)

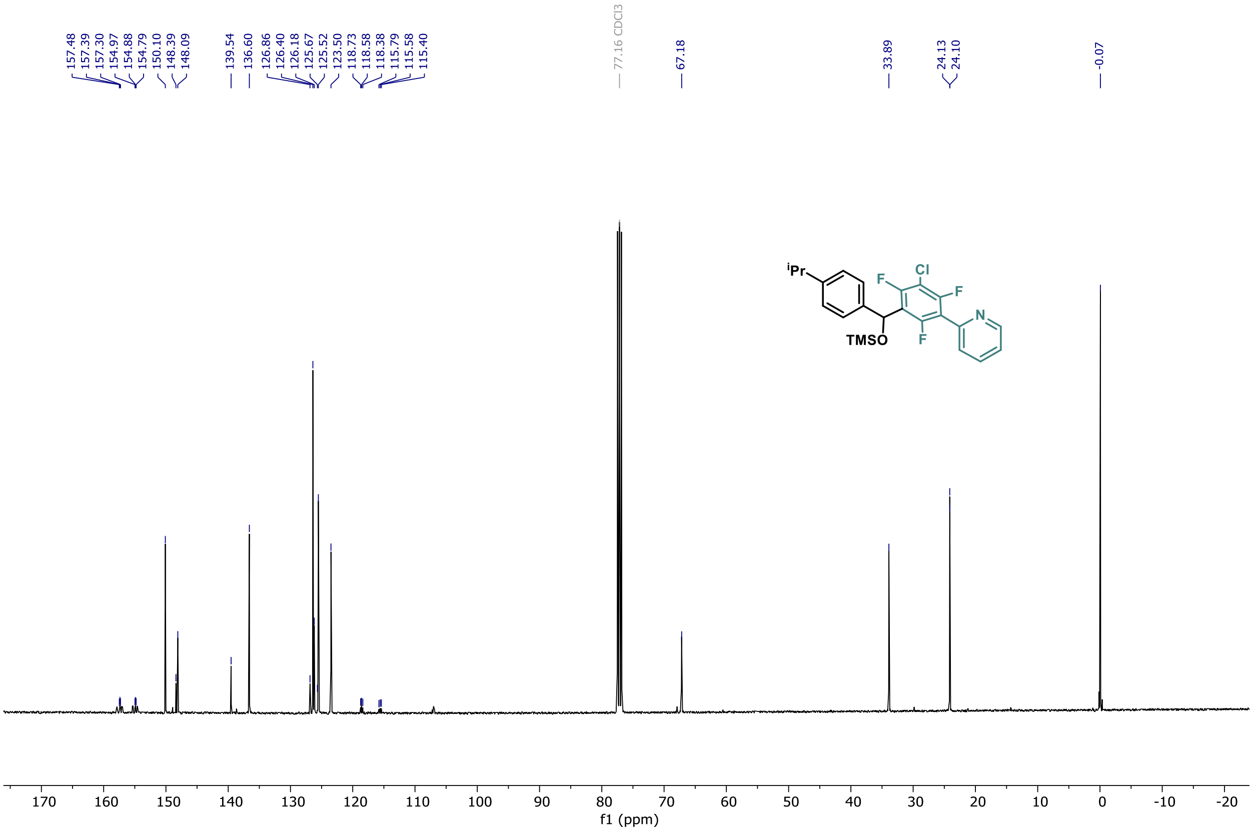

^{1}H NMR of **4m** (300 MHz, $CDCl_3$, 298K)

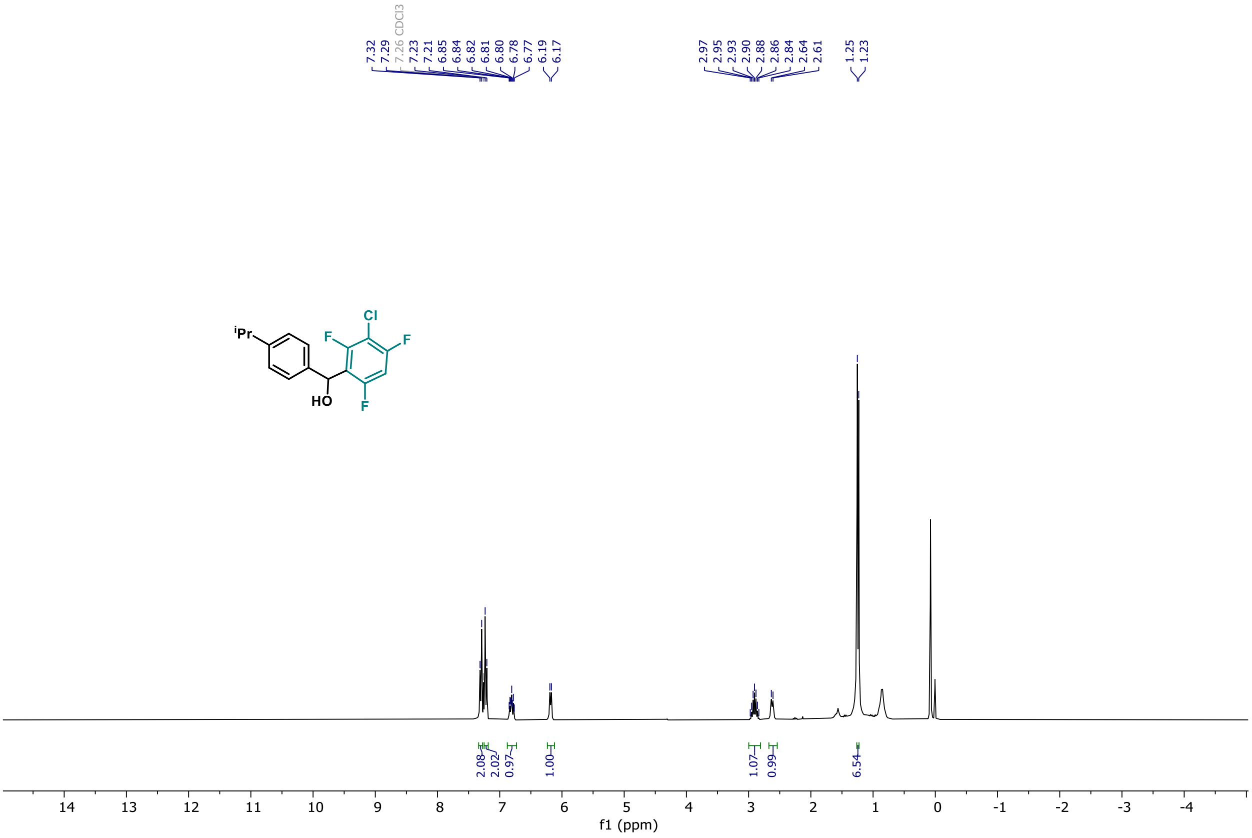

^{19}**F NMR** of **4m** (282 MHz, $CDCl_3$, 298K)

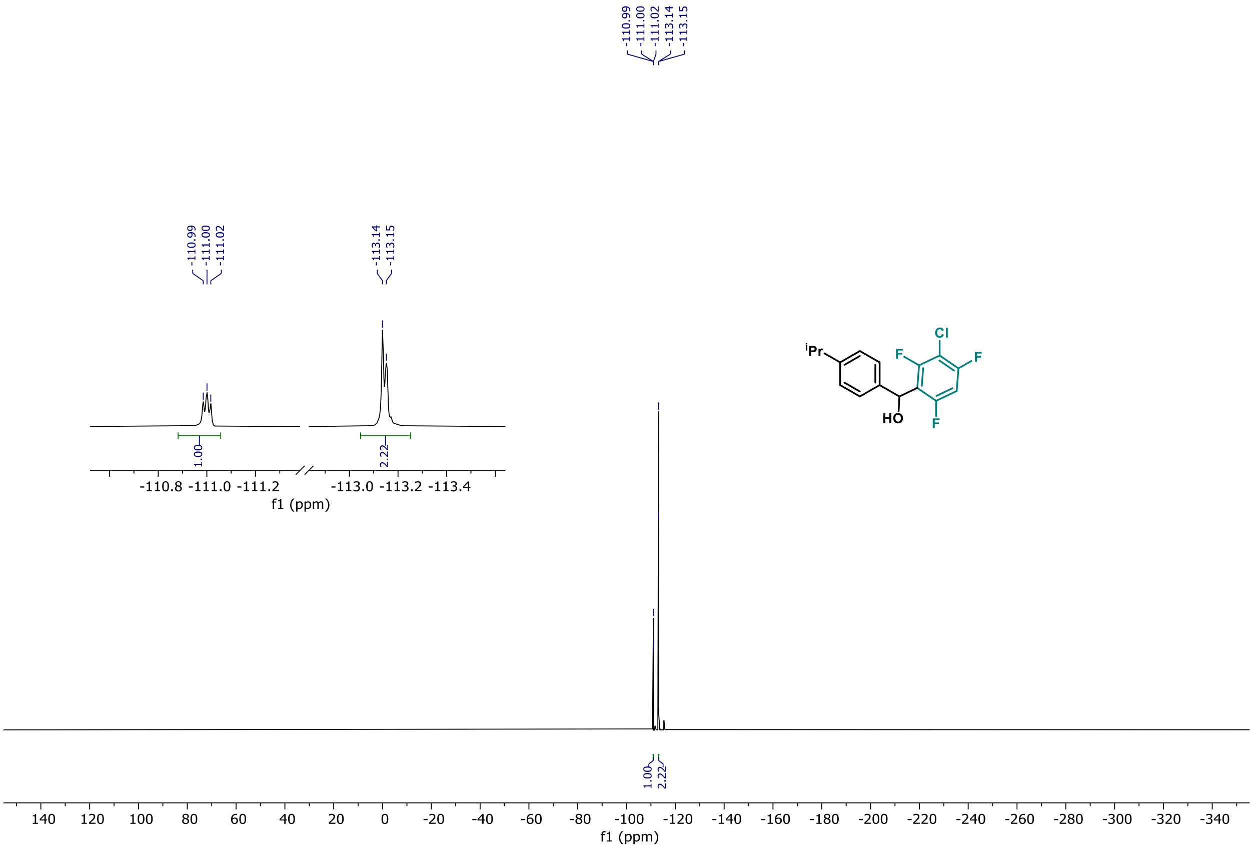

^{13}**C NMR** of **4m** (101 MHz, $CDCl_3$, 298K)

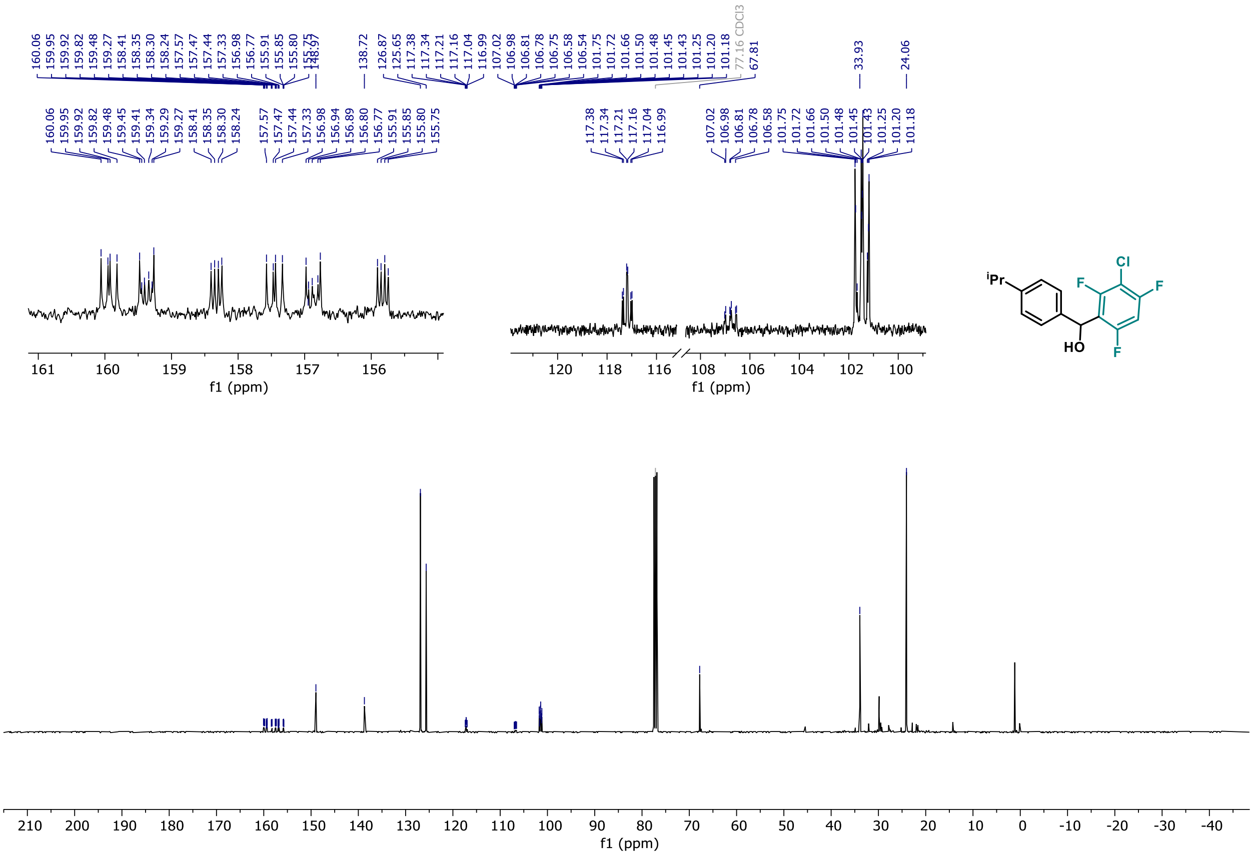

^{1}H NMR of **4n** (300 MHz, $CDCl_3$, 298K)

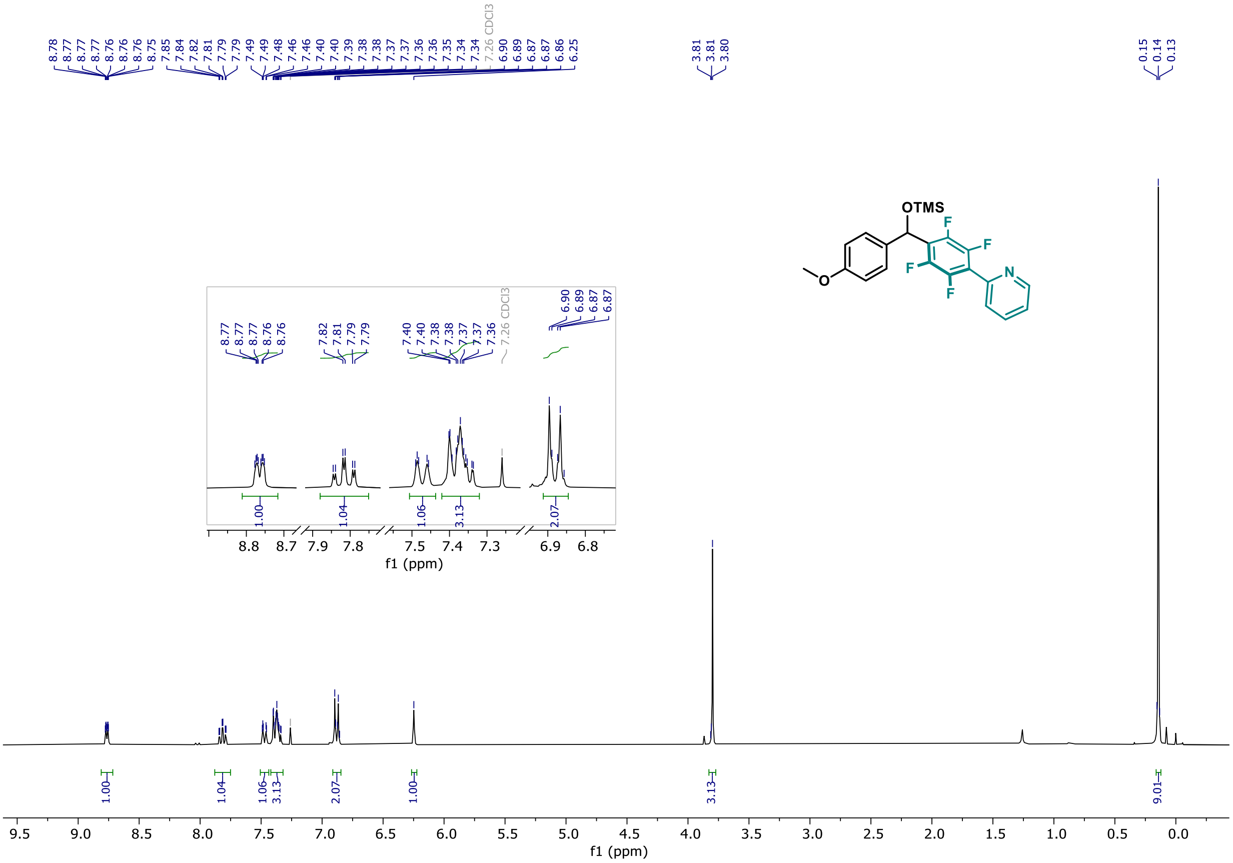

^{19}F NMR of **4n** (282 MHz, $CDCl_3$, 298K)

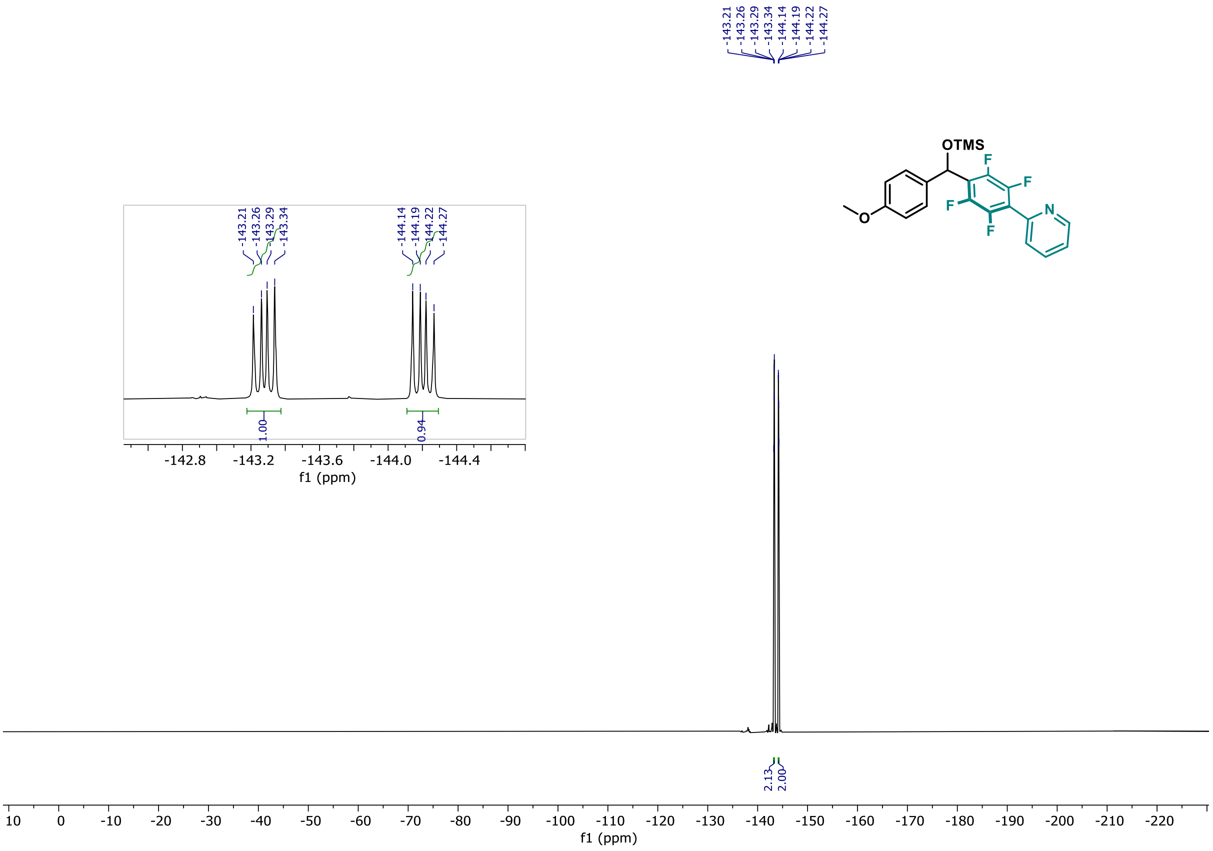

^{29}**Si NMR** of **4n** (60 MHz, $CDCl_3$, 298K)

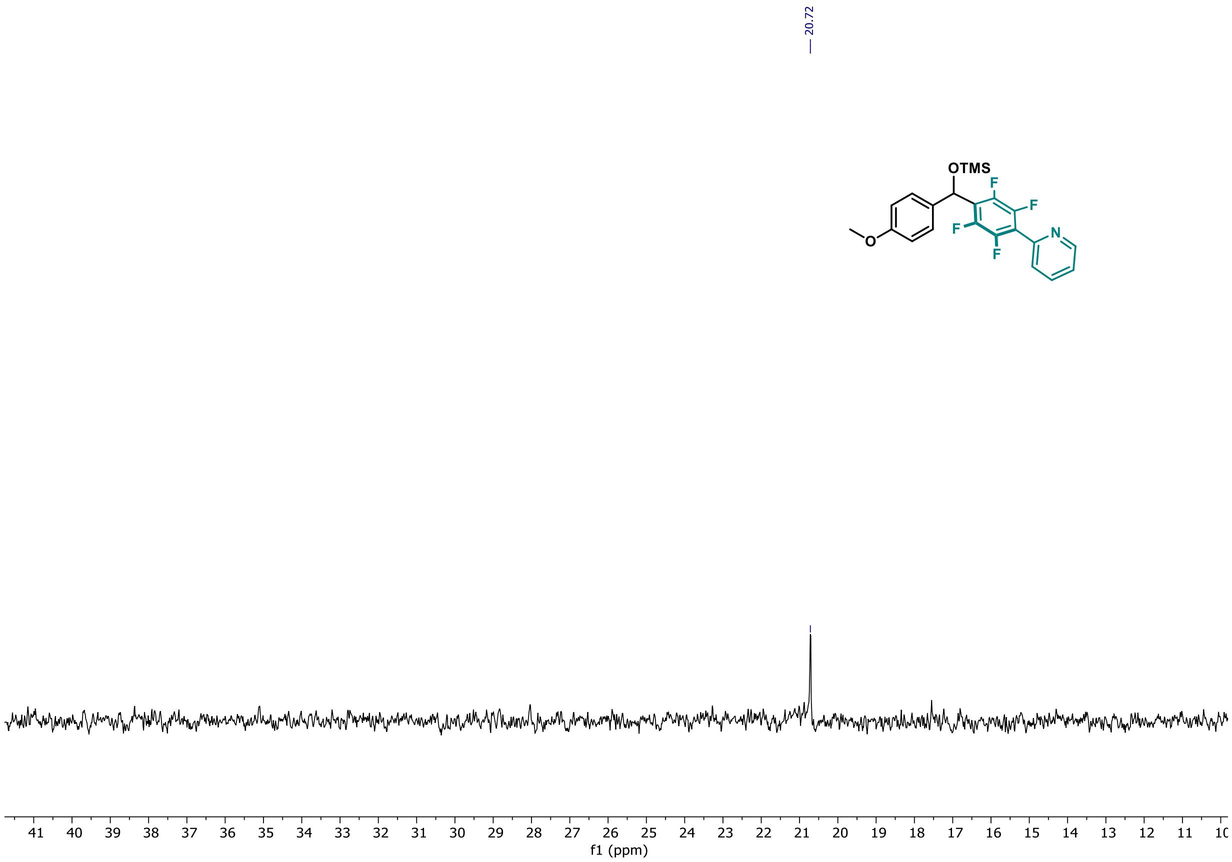

^{13}**C NMR** of **4n** (75 MHz, $CDCl_3$, 298K)

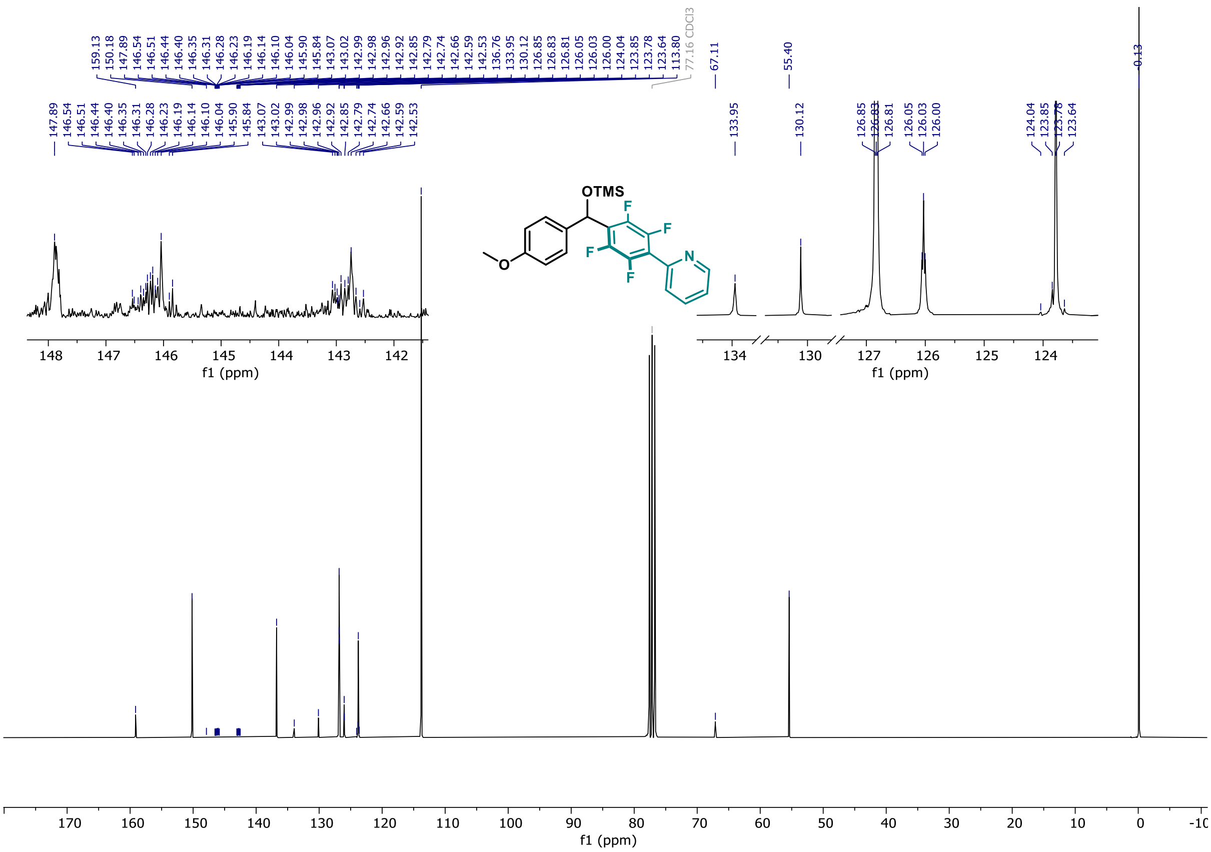

^{1}H NMR of 4o (300 MHz, $CDCl_3$, 298K)

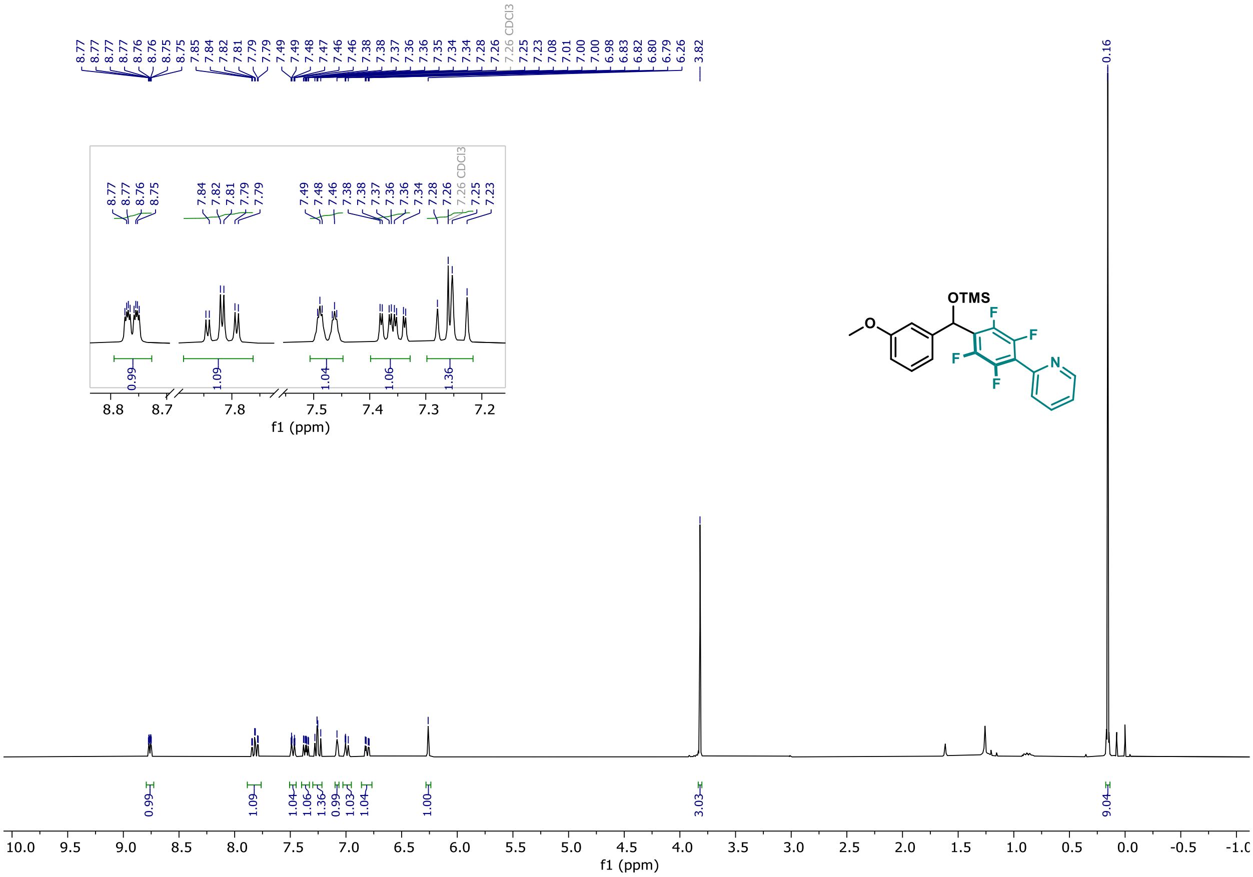

^{19}F NMR of **4o** (282 MHz, $CDCl_3$, 298K)

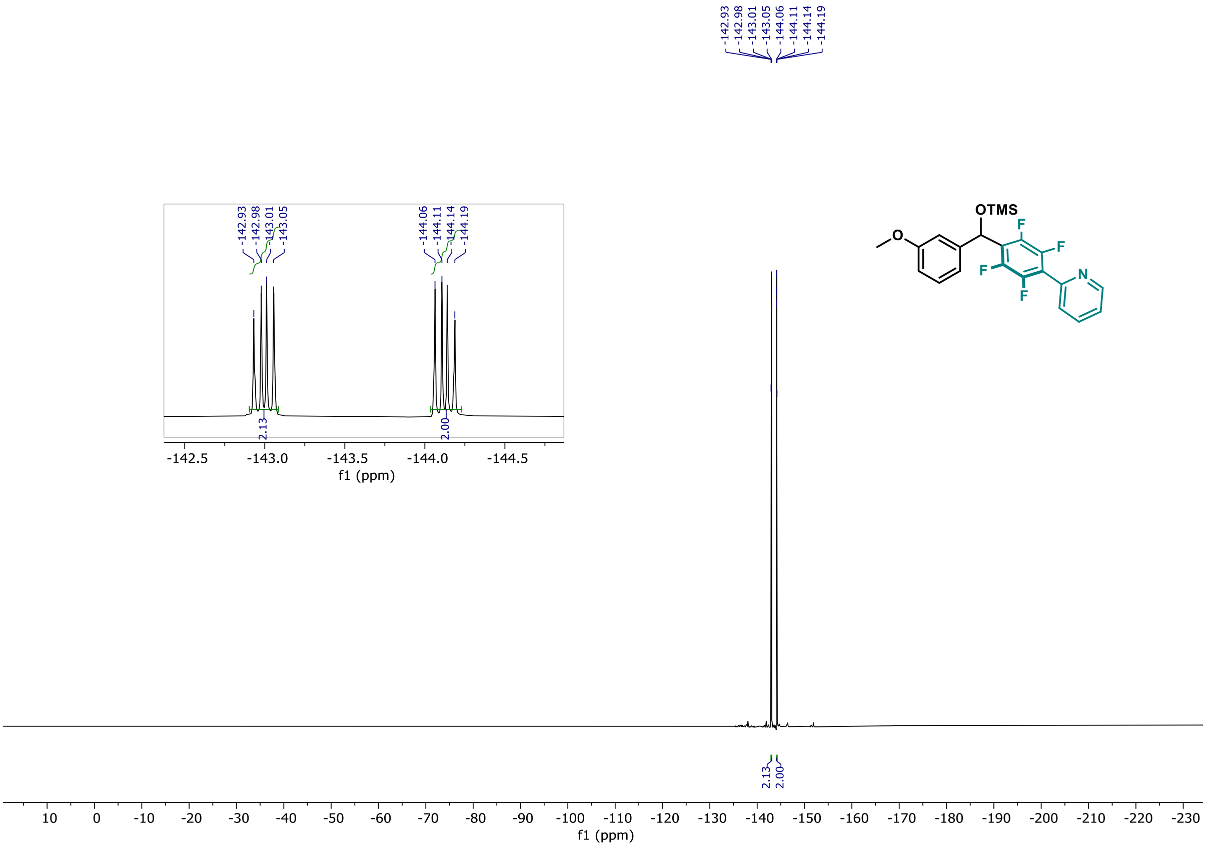

^{29}Si NMR of **4o** (60 MHz, $CDCl_3$, 298K)

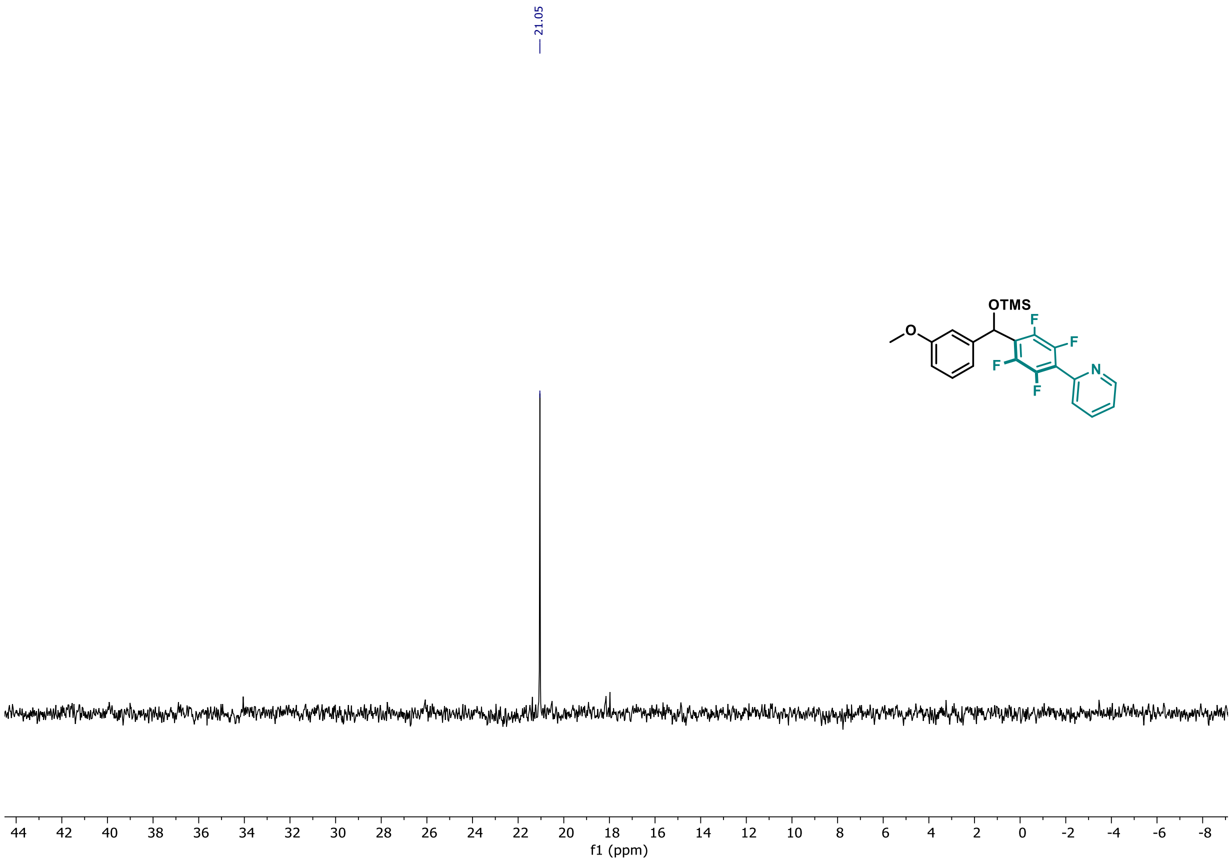

^{13}C NMR of **4o** (75 MHz, $CDCl_3$, 298K)

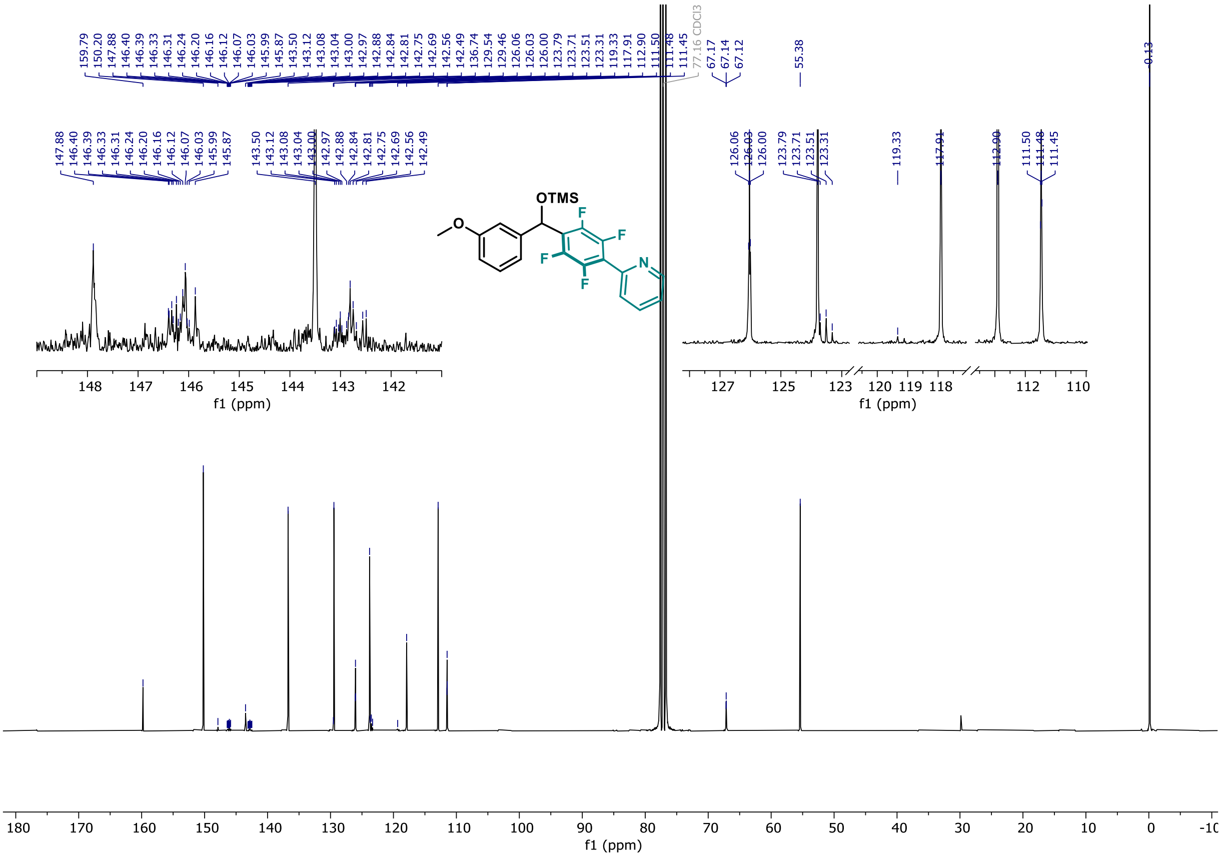

^{1}H NMR of **4p** (300 MHz, $CDCl_3$, 298K)

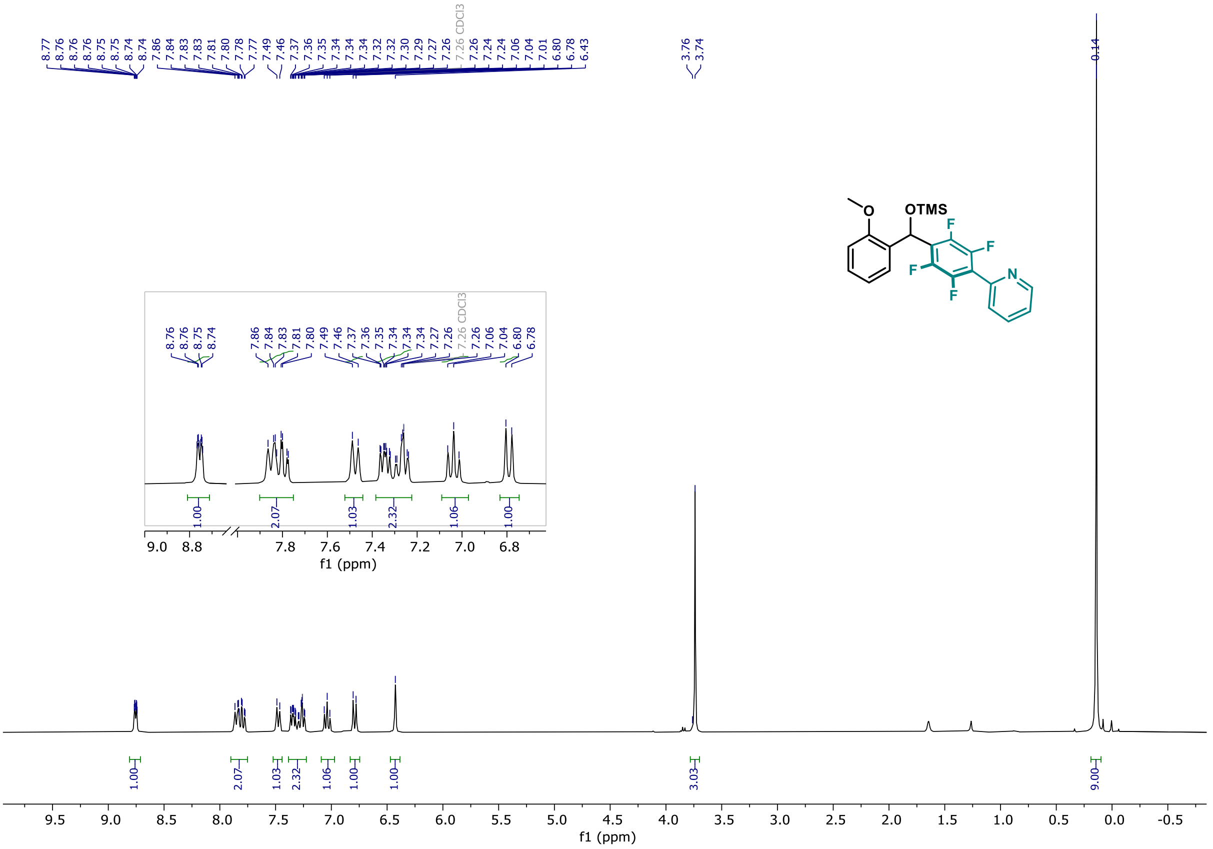

^{19}F NMR of **4p** (282 MHz, $CDCl_3$, 298K)

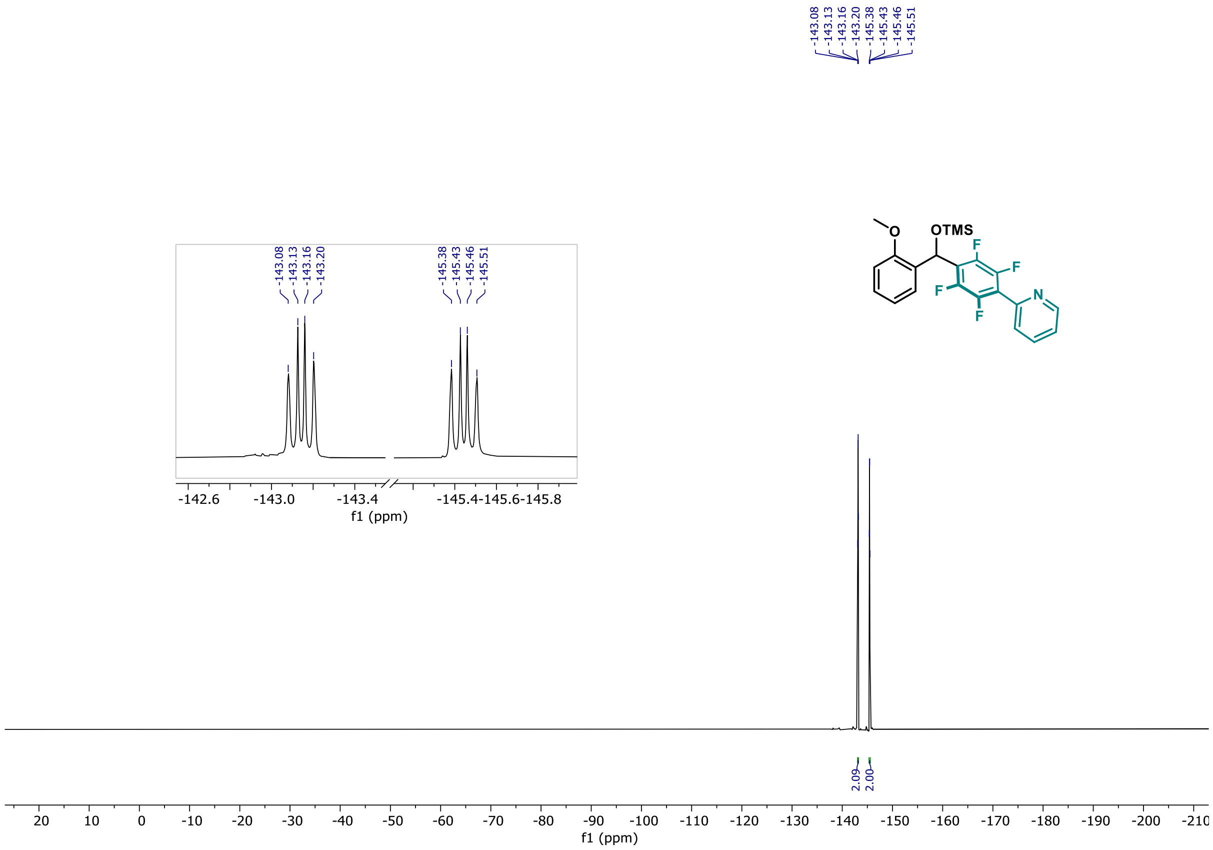

^{29}Si NMR of **4p** (60 MHz, $CDCl_3$, 298K)

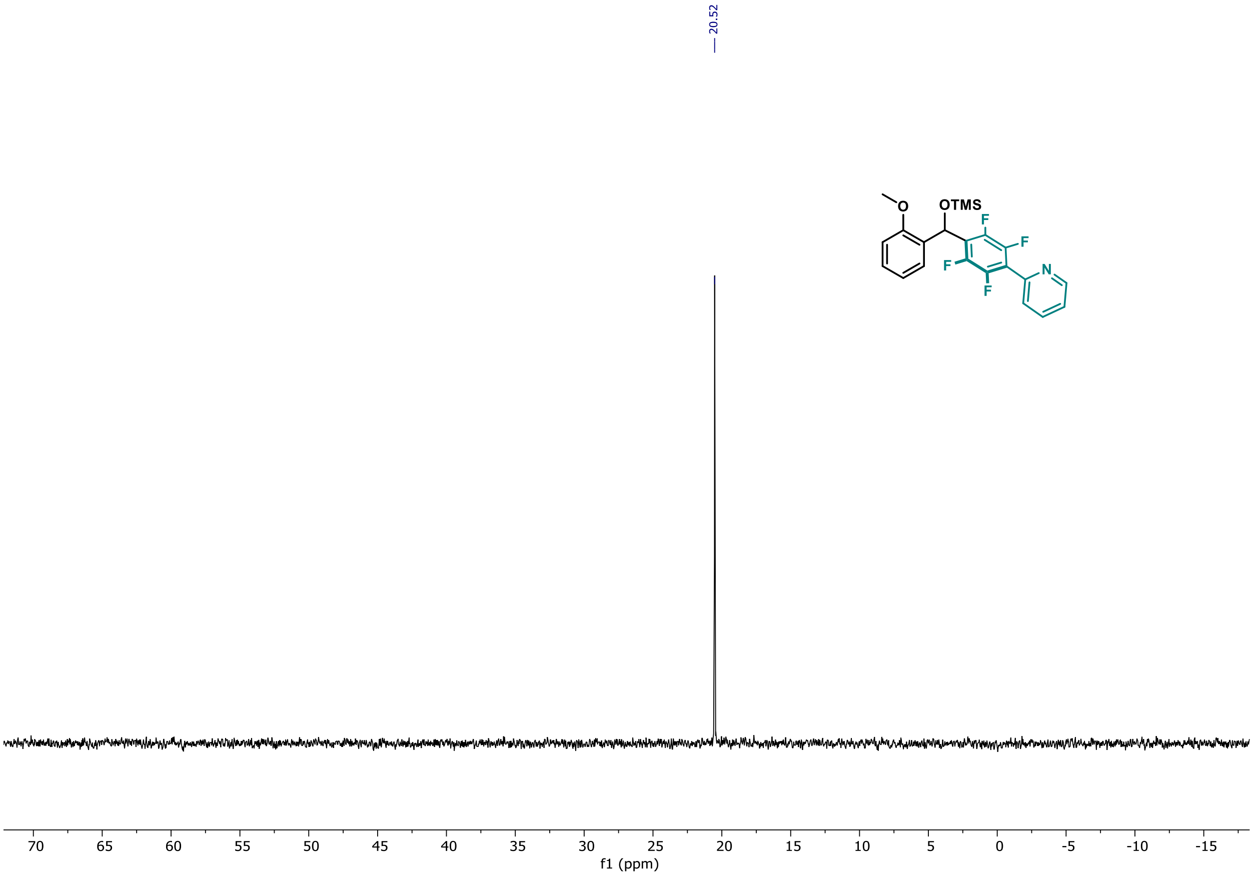

^{13}C NMR of **4p** (75 MHz, $CDCl_3$, 298K)

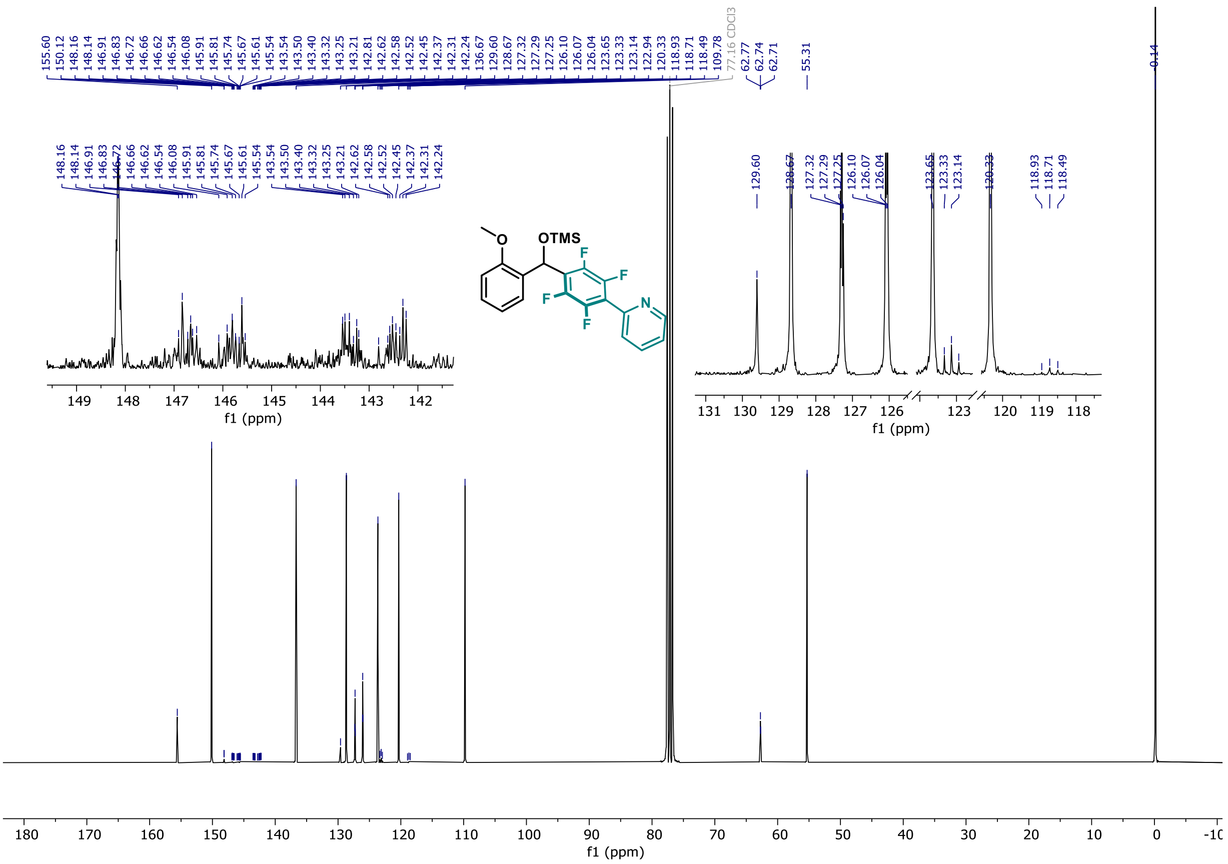

^{1}H NMR of **4q** (300 MHz, $CDCl_3$, 298K)

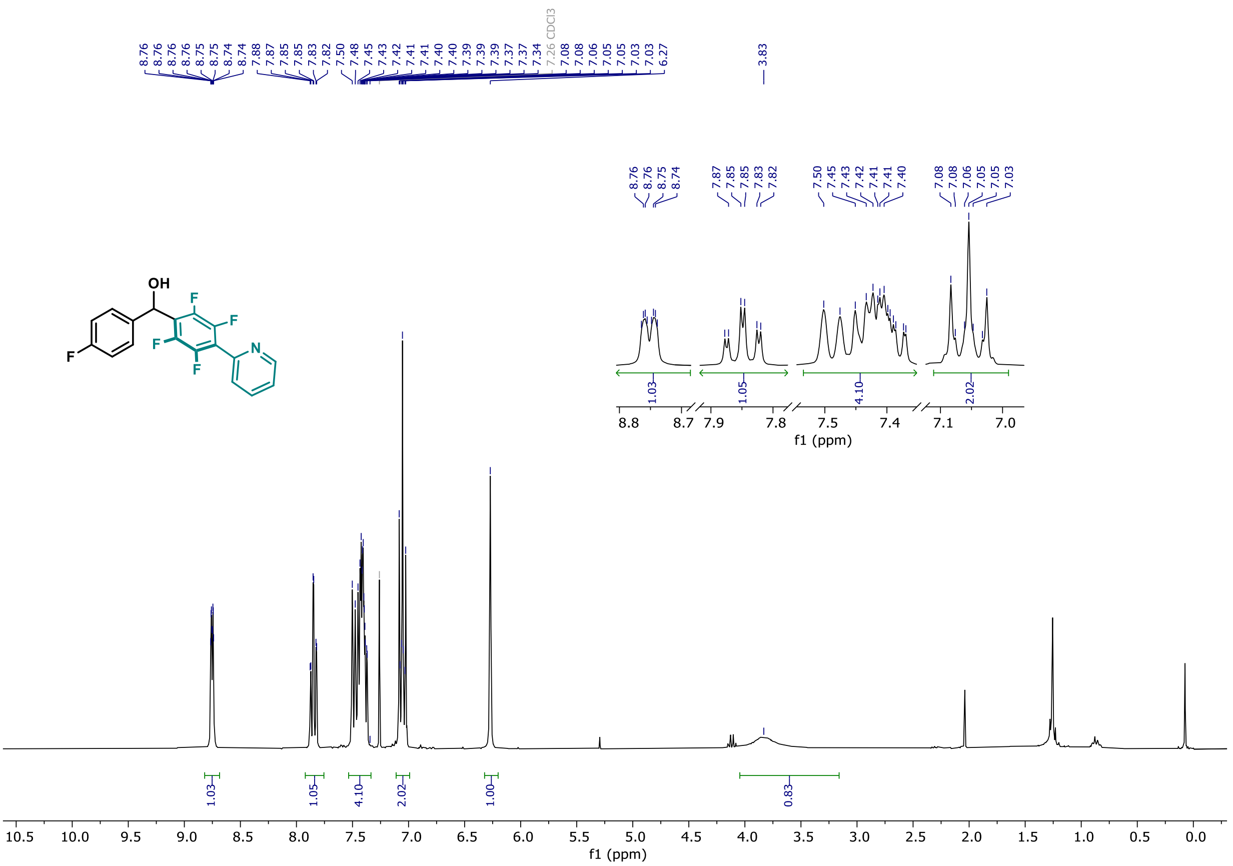

^{19}F NMR of **4q** (282 MHz, $CDCl_3$, 298K)

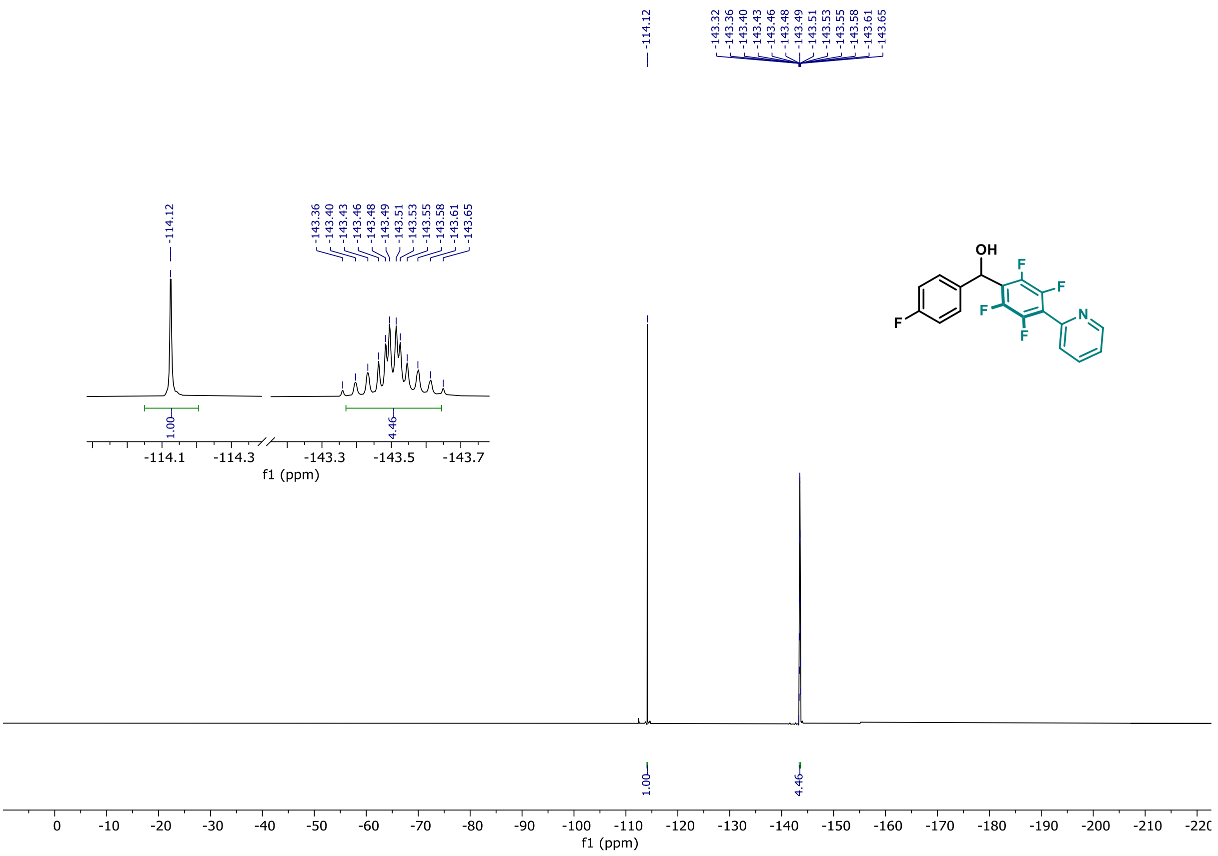

^{13}C NMR of 4q (75 MHz, $CDCl_3$, 298K)

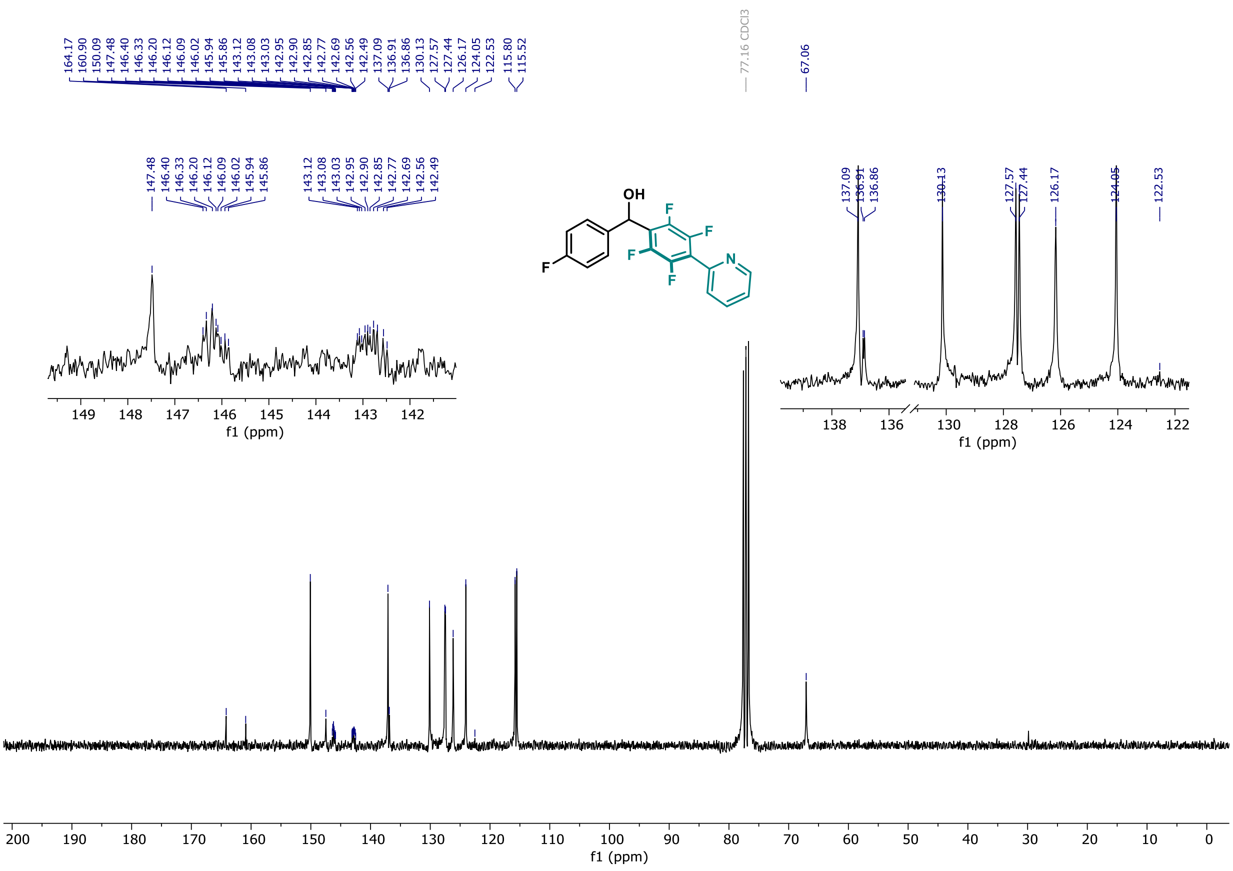

^{1}H NMR of 4r (600 MHz, $CDCl_3$, 298K)

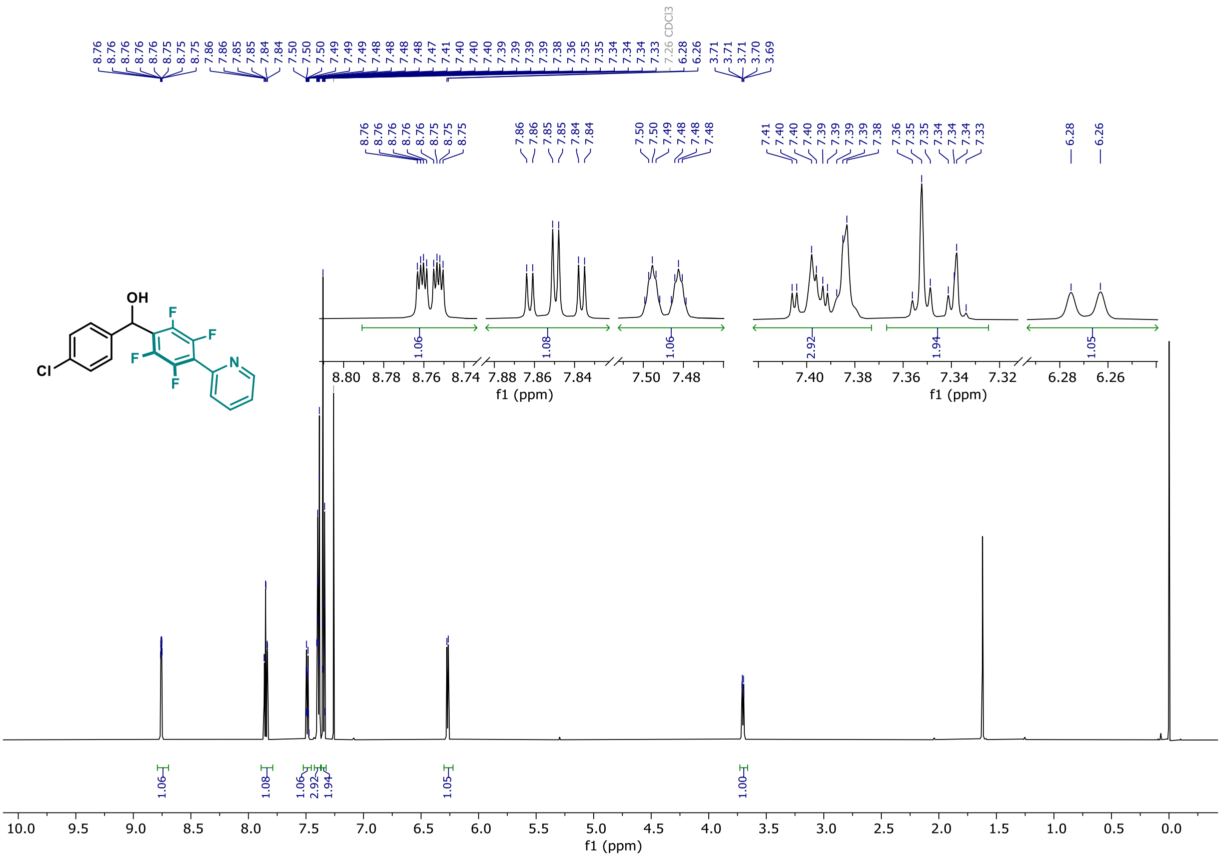

^{19}F NMR of **4r** (565 MHz, $CDCl_3$, 298K)

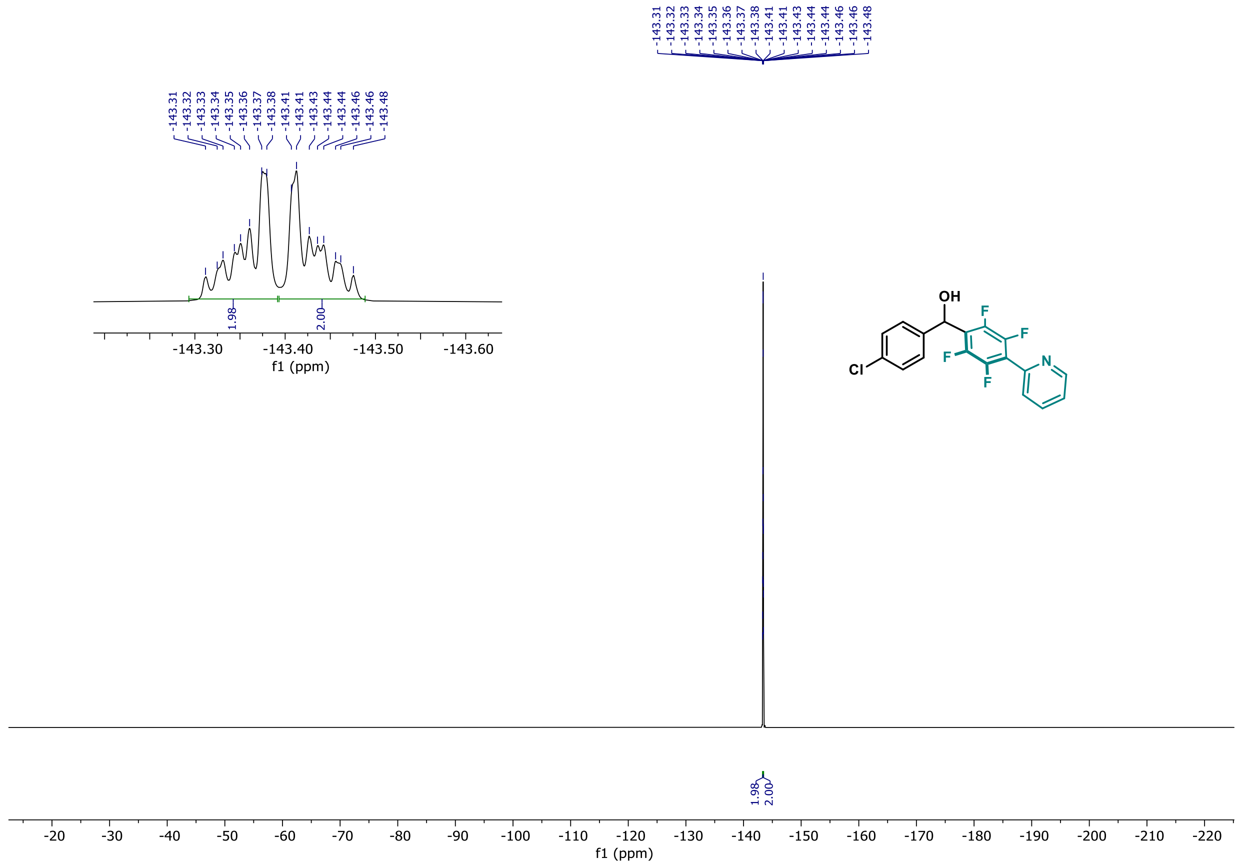

^{13}C NMR of **4r** (151 MHz, $CDCl_3$, 298K)

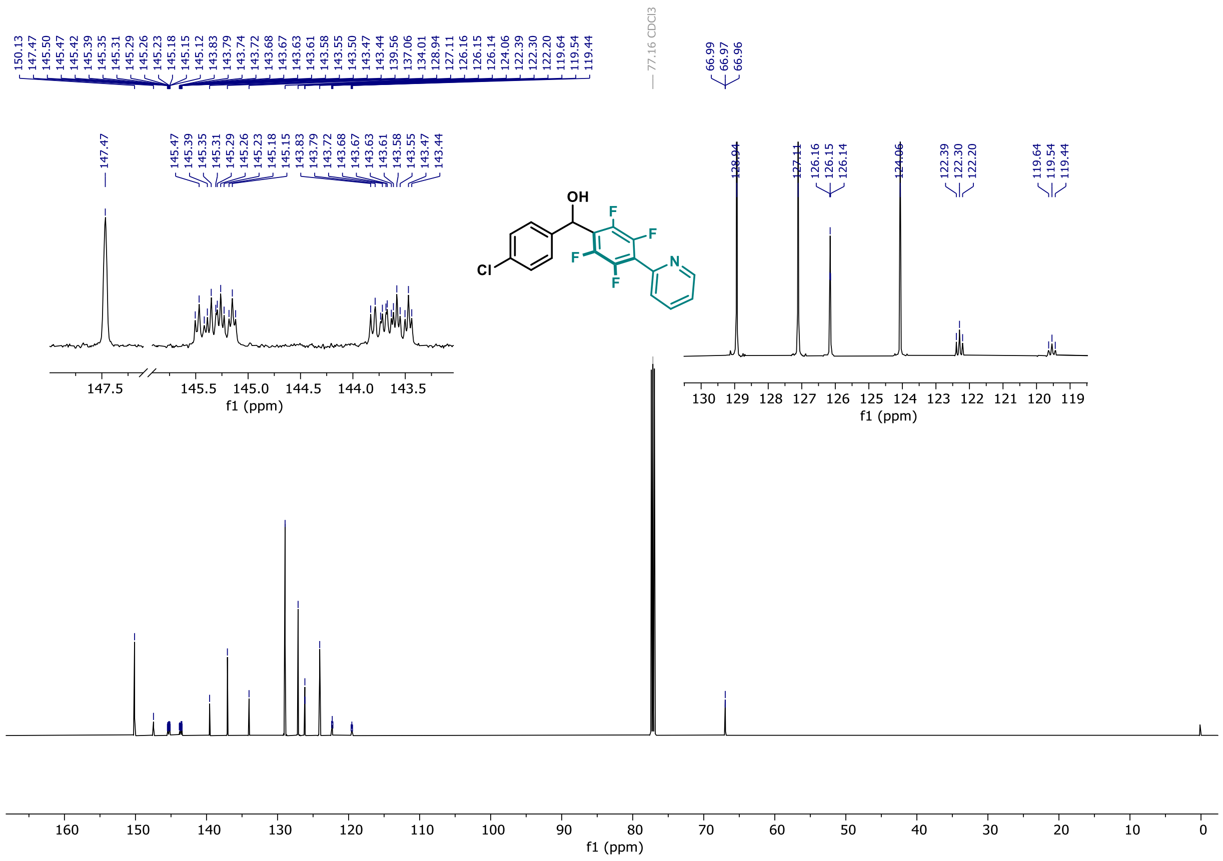

^{1}H NMR of **4s** (300 MHz, $CDCl_3$, 298K)

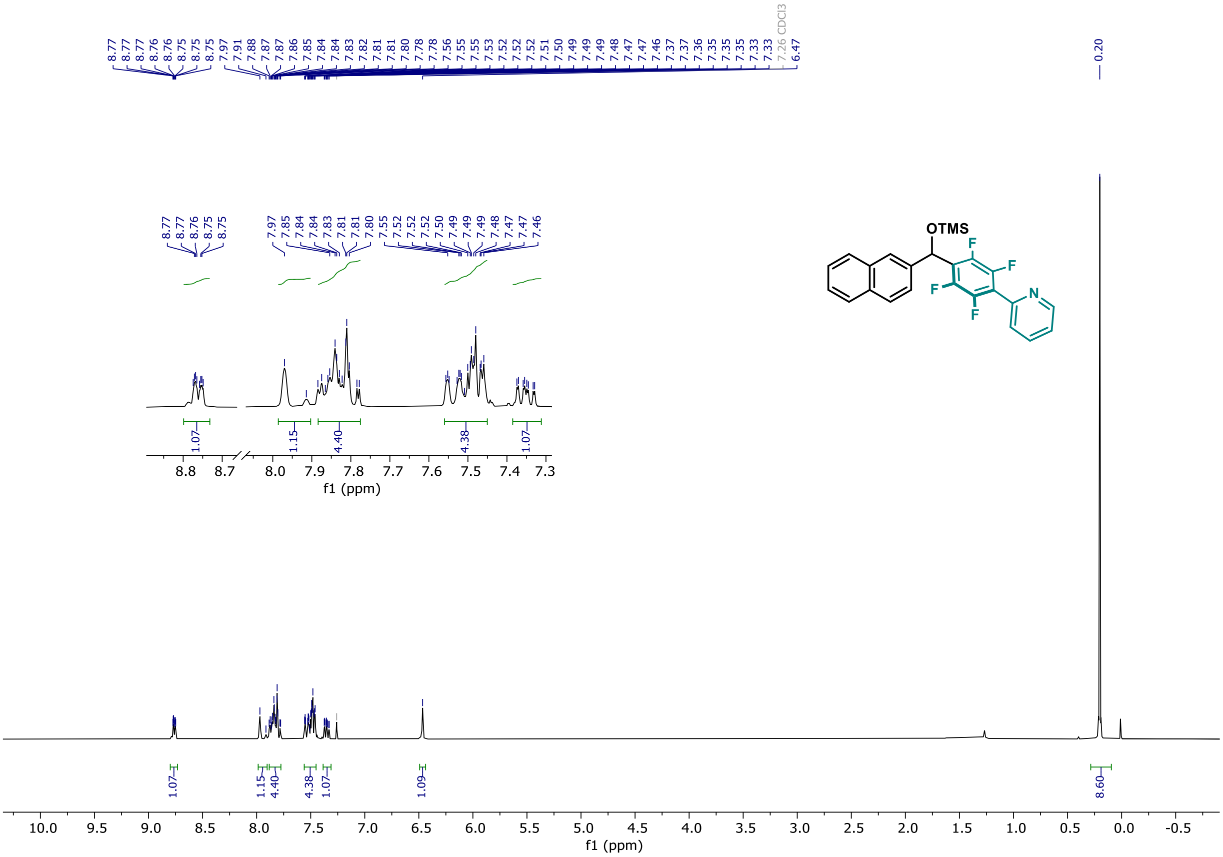

^{19}F NMR of **4s** (282 MHz, $CDCl_3$, 298K)

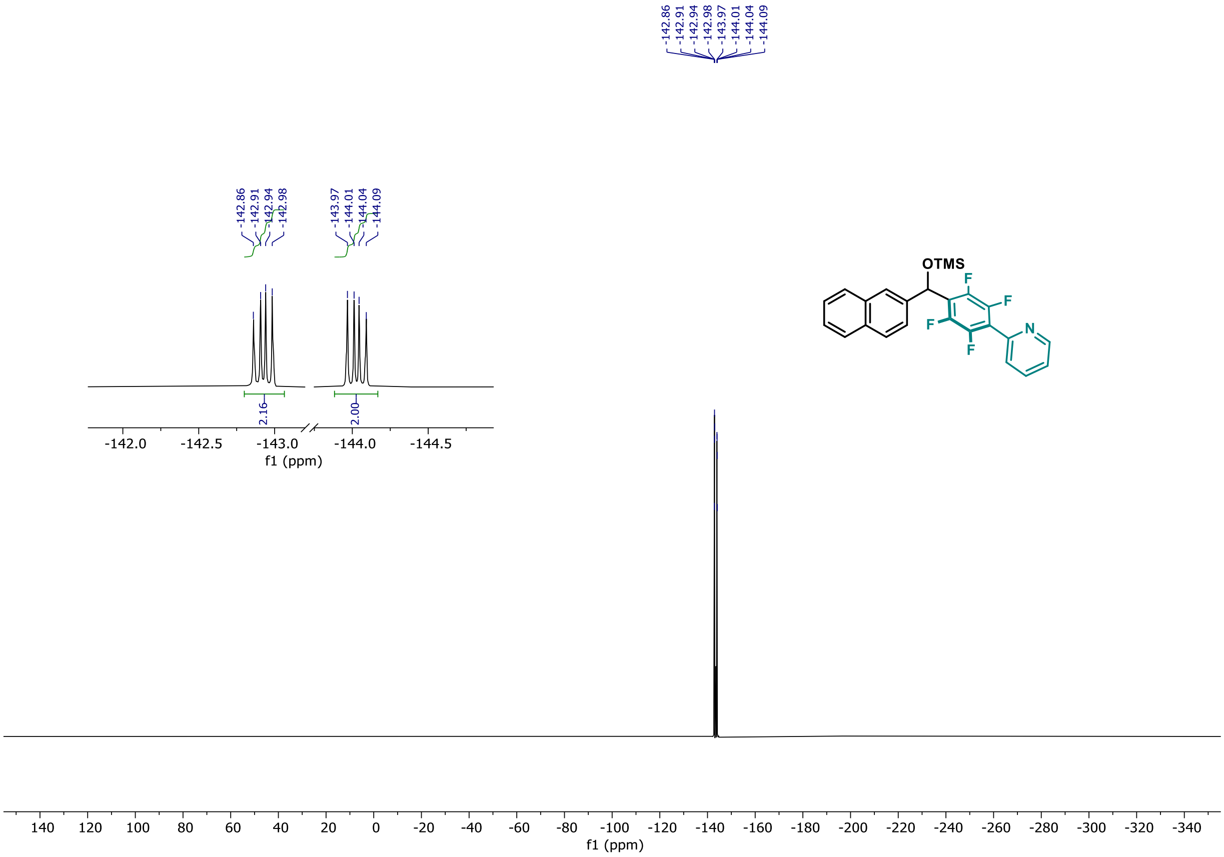

^{29}Si NMR of **4s** (60 MHz, $CDCl_3$, 298K)

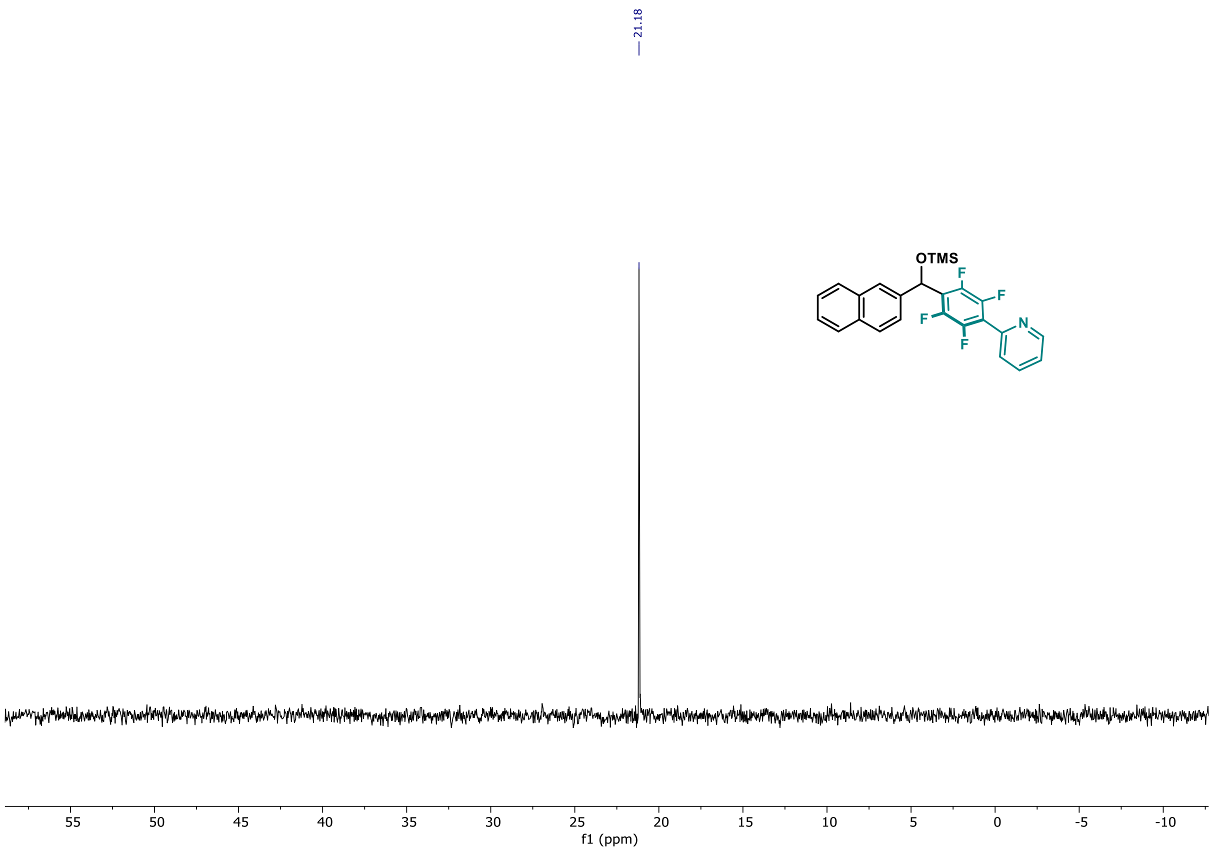

^{13}C NMR of **4s** (75 MHz, $CDCl_3$, 298K)

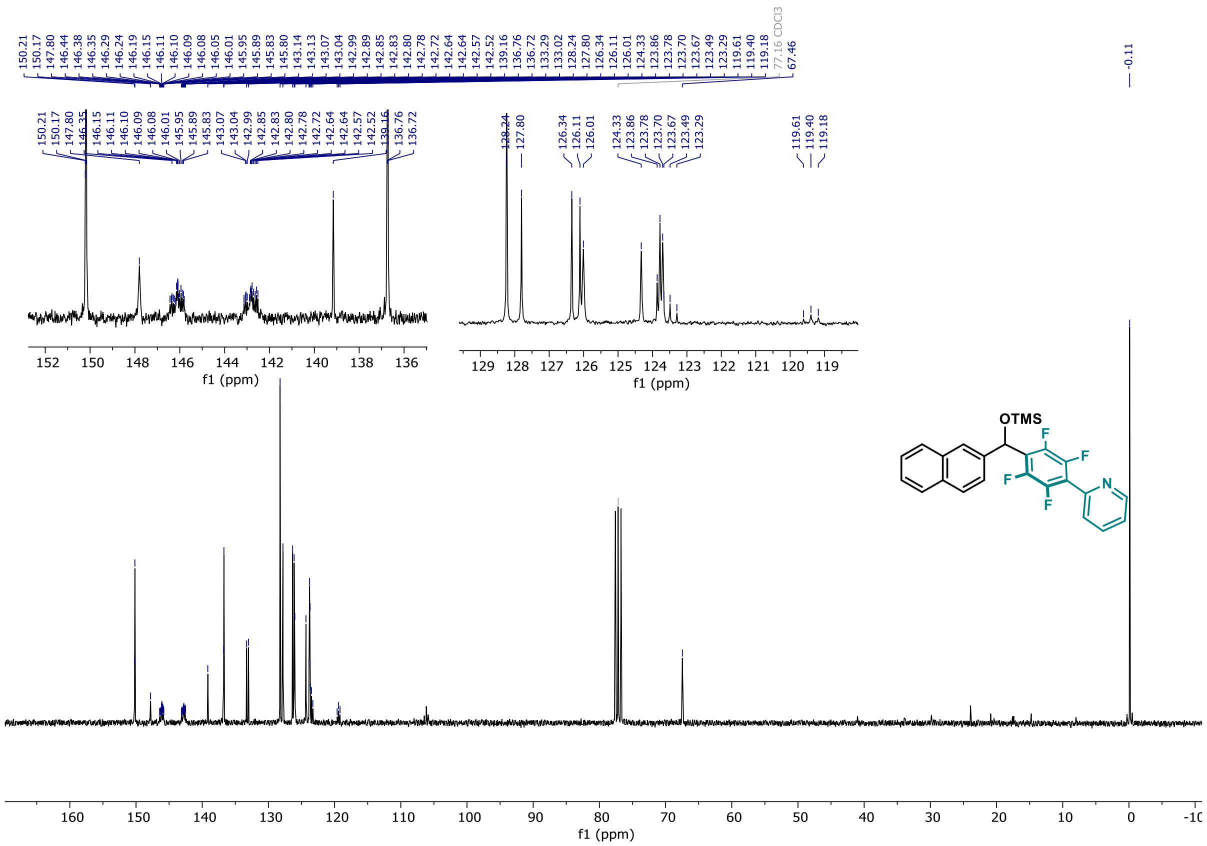

^{1}H NMR of **4t** (400 MHz, $CDCl_3$, 298K)

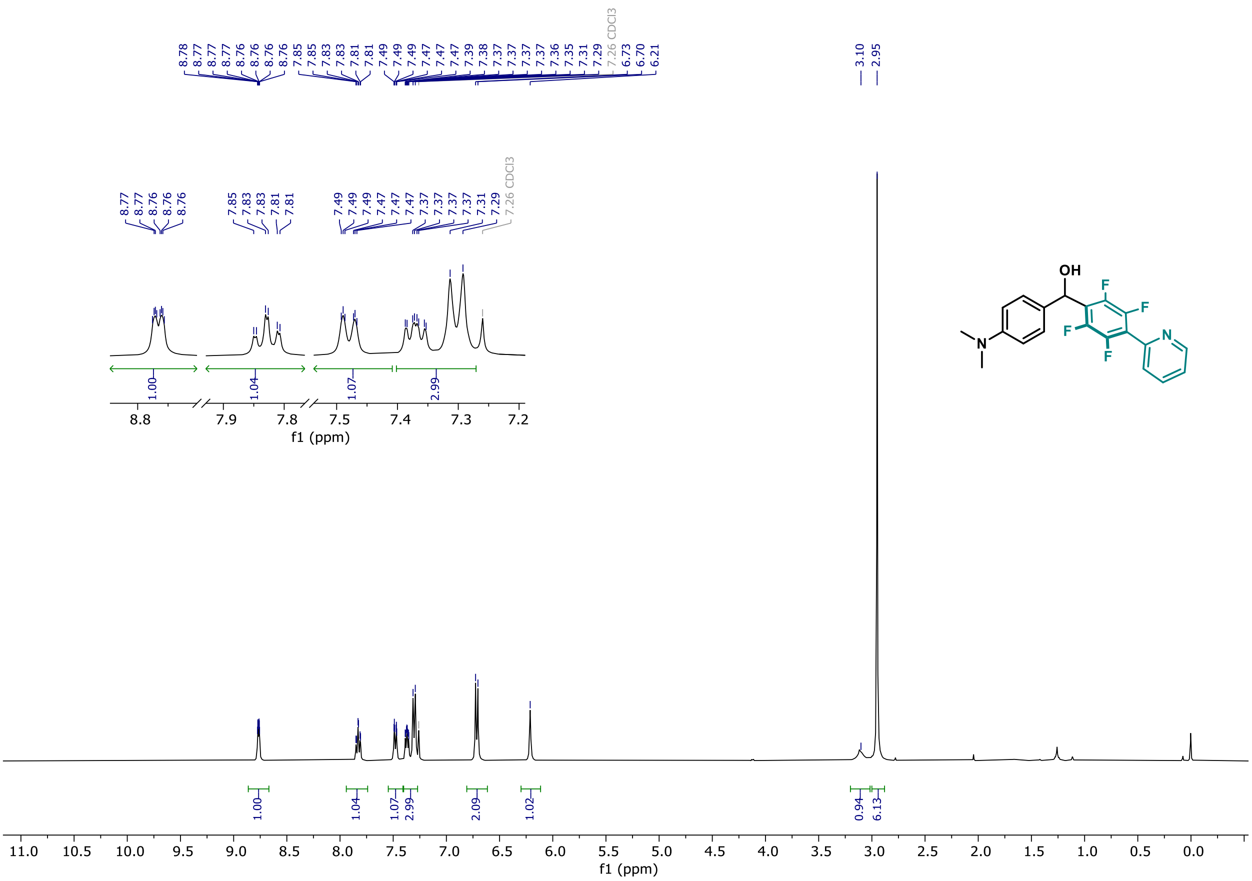

^{19}F NMR of **4t** (282 MHz, $CDCl_3$, 298K)

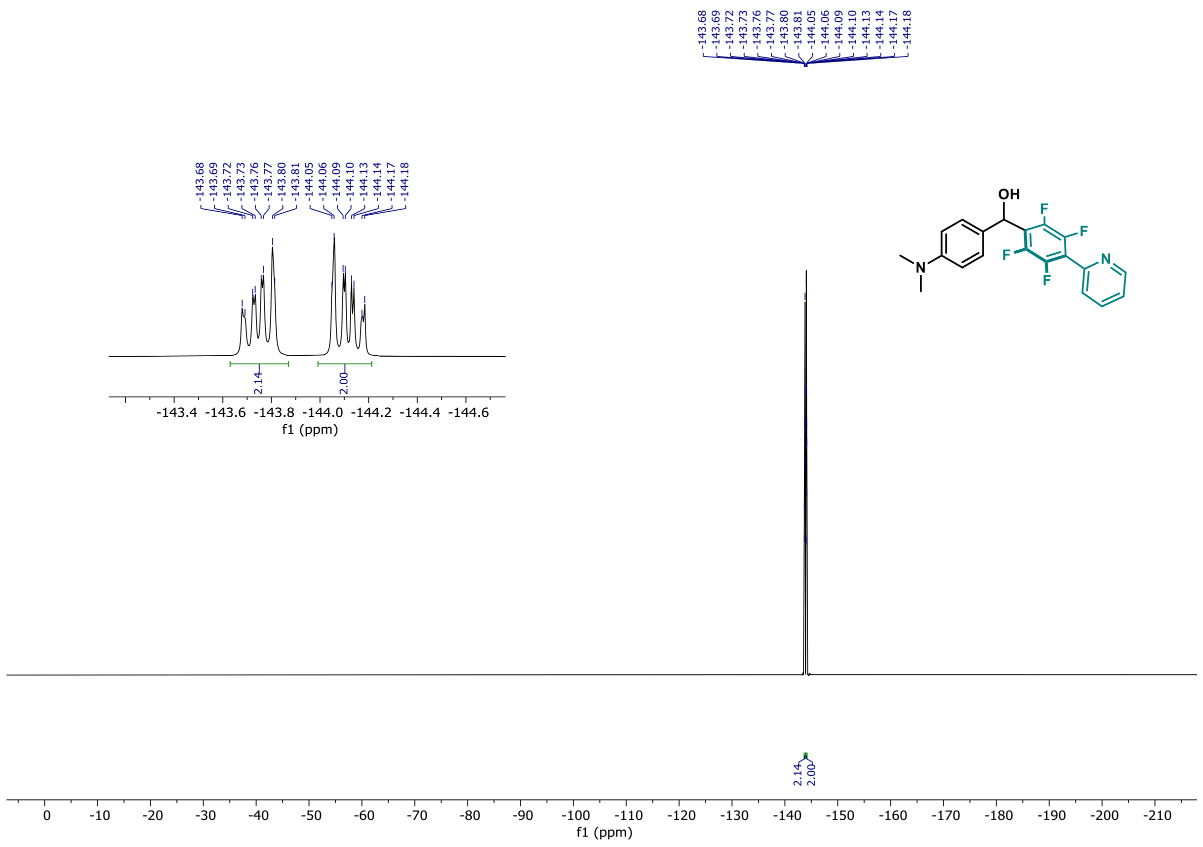

^{13}C NMR of **4t** (101 MHz, $CDCl_3$, 298K)

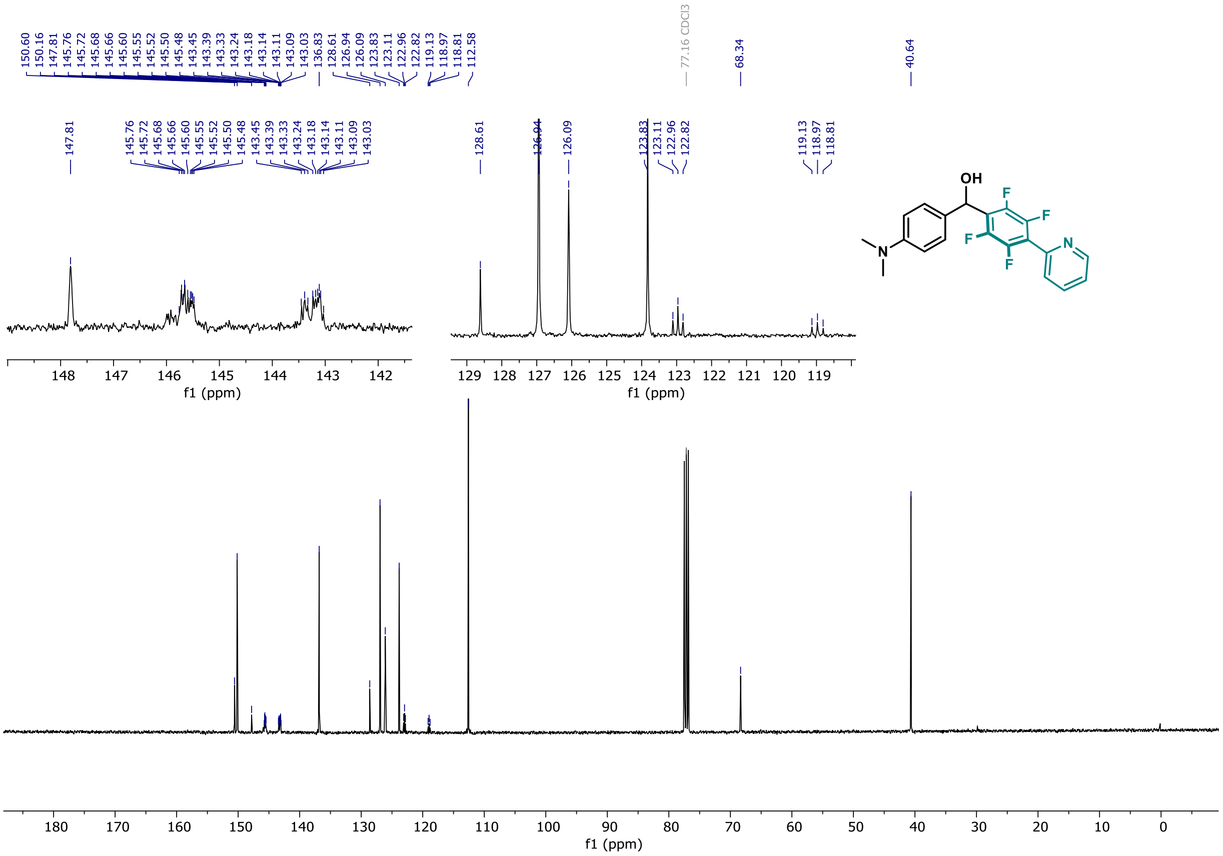

^{1}H NMR of **4u** (300 MHz, $CDCl_3$, 298K)

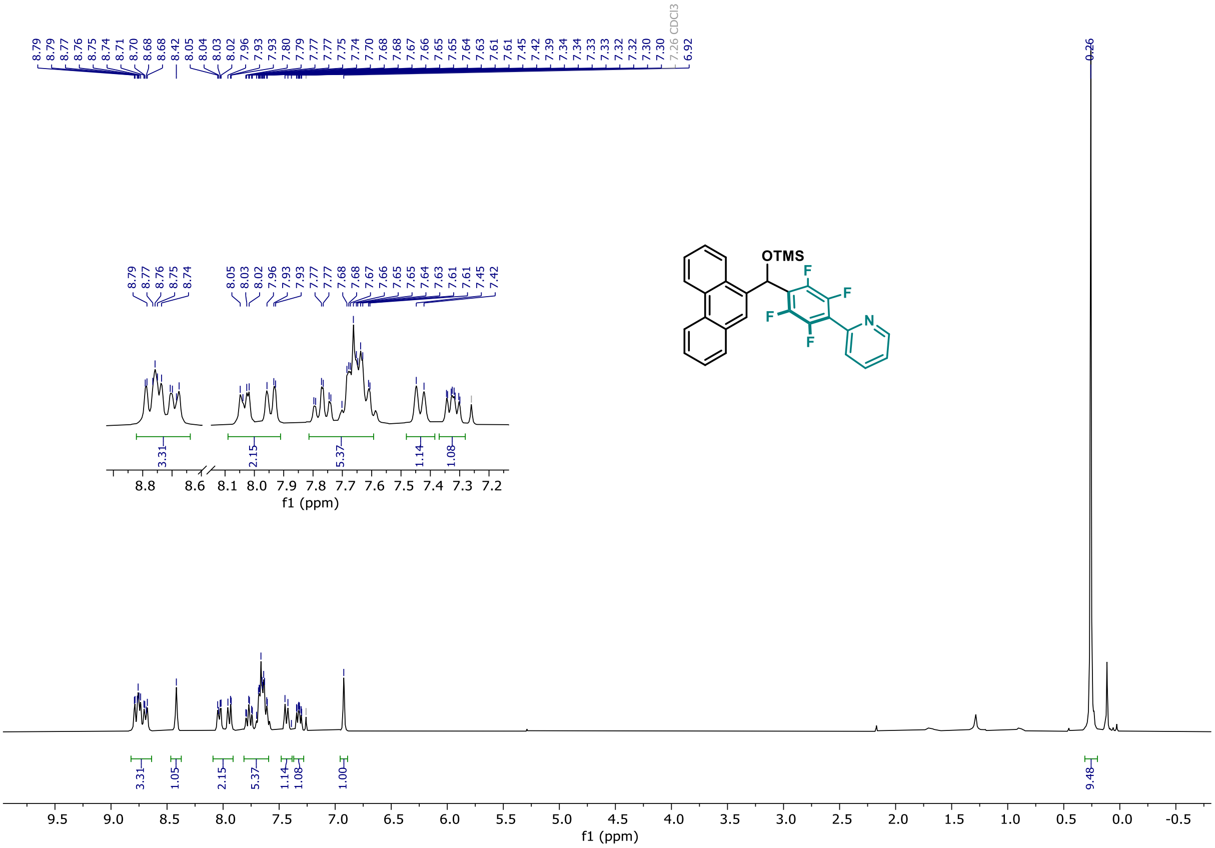

^{19}F NMR of **4u** (282 MHz, $CDCl_3$, 298K)

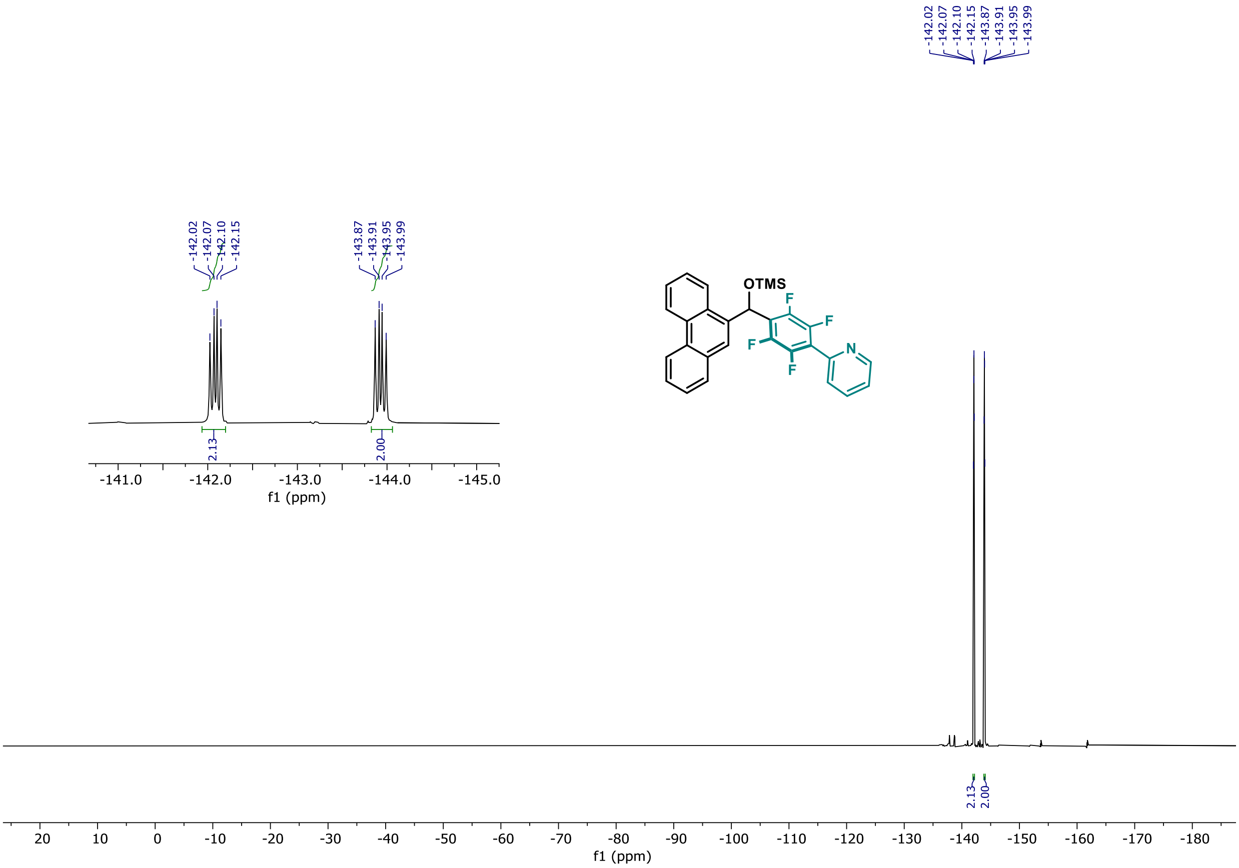

^{29}Si NMR of **4u** (60 MHz, $CDCl_3$, 298K)

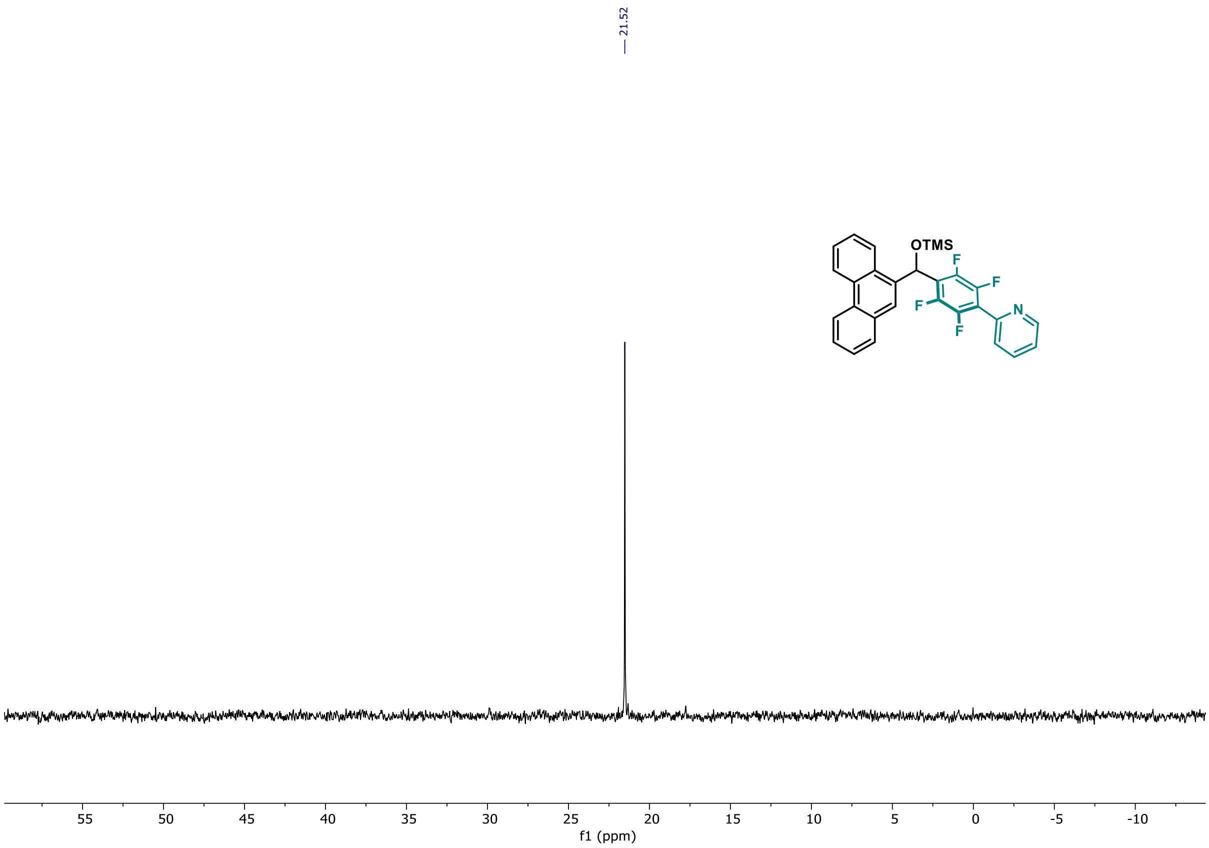

^{13}C NMR of **4u** (75 MHz, $CDCl_3$, 298K)

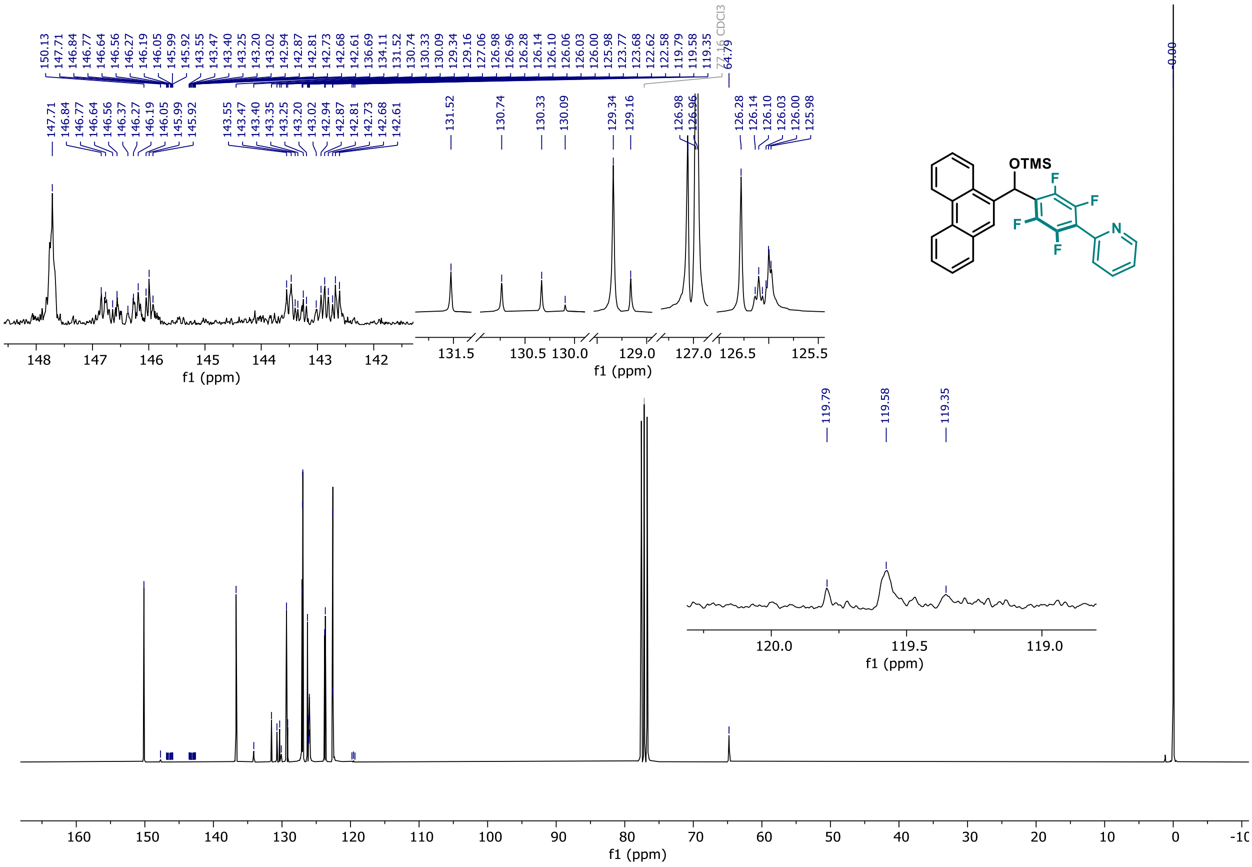

^{1}H NMR of **4v** (300 MHz, $CDCl_3$, 298K)

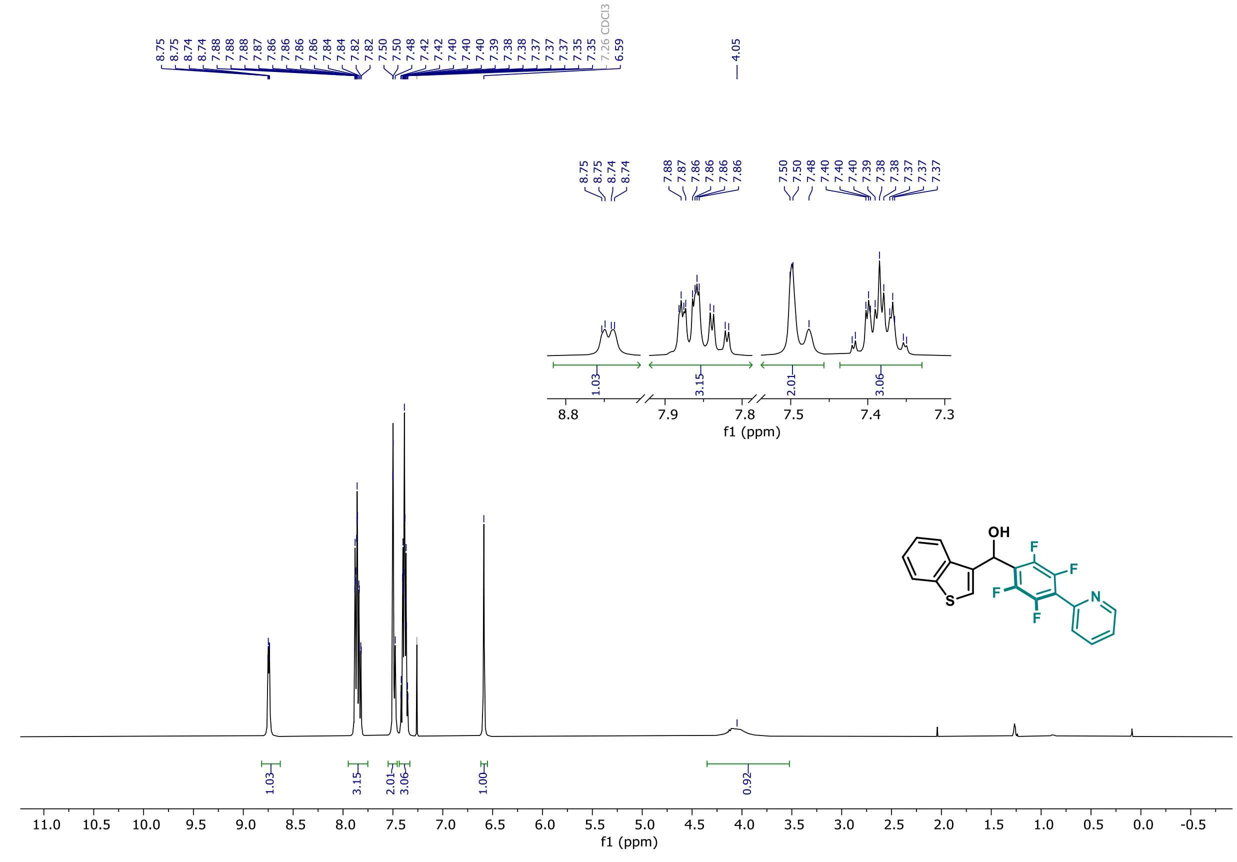

[19]**F NMR** of **4v** (282 MHz, $CDCl_3$, 298K)

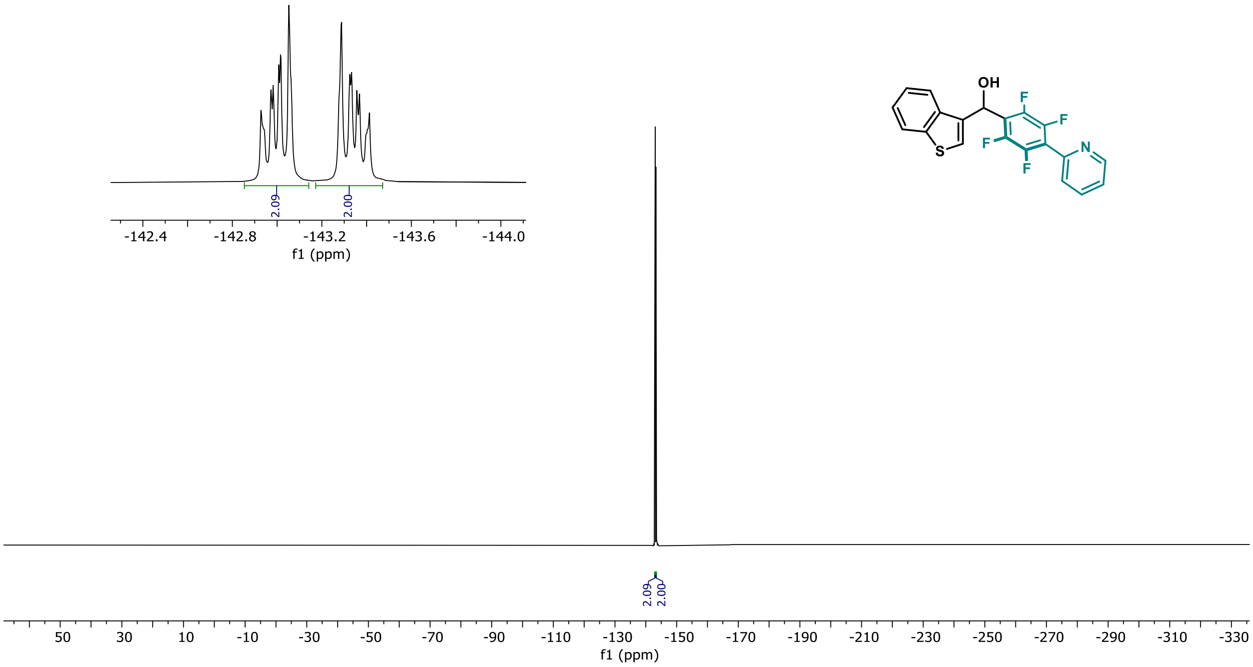

[13]**C NMR** of **4v** (101 MHz, $CDCl_3$, 298K)

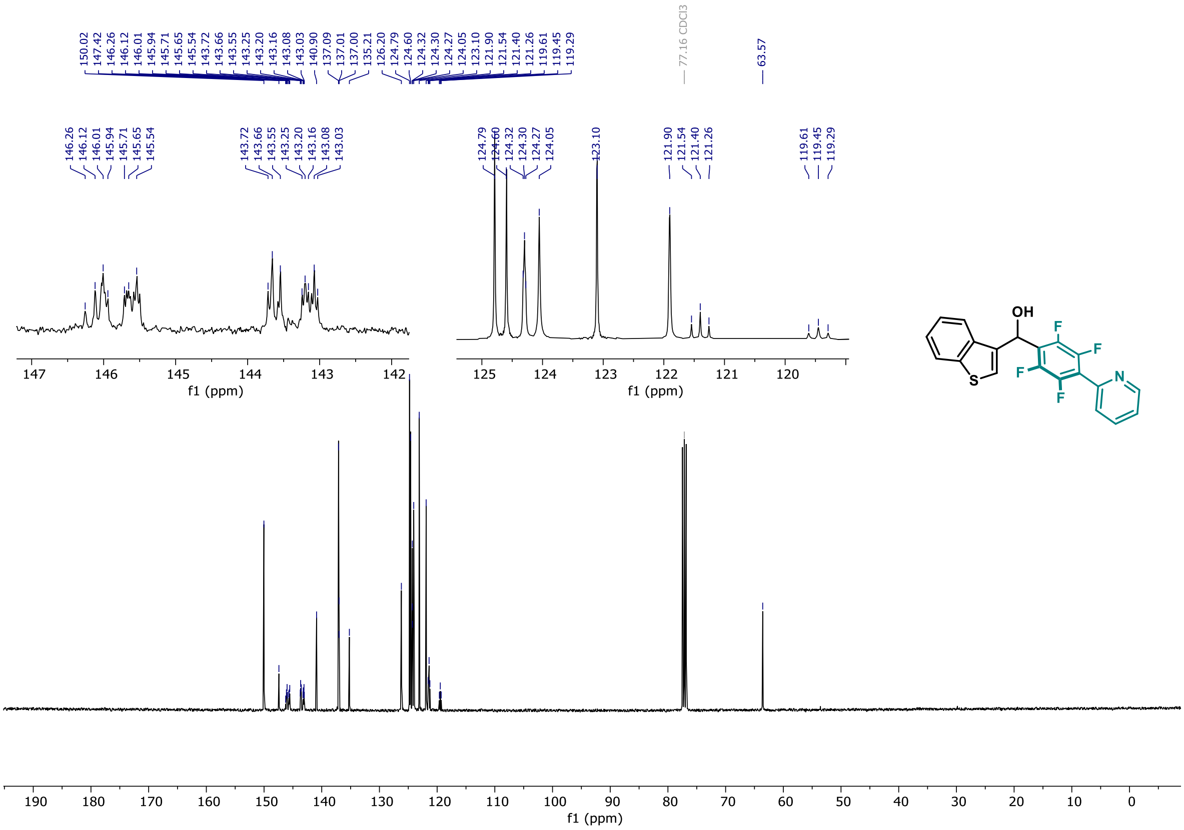

Crude ^{1}H NMR of 4w•TMS (300 MHz, $CDCl_3$, 298K)

Crude ^{1}H NMR yield of **4w**•TMS before TMS deprotection using trichloroethene as internal standard.

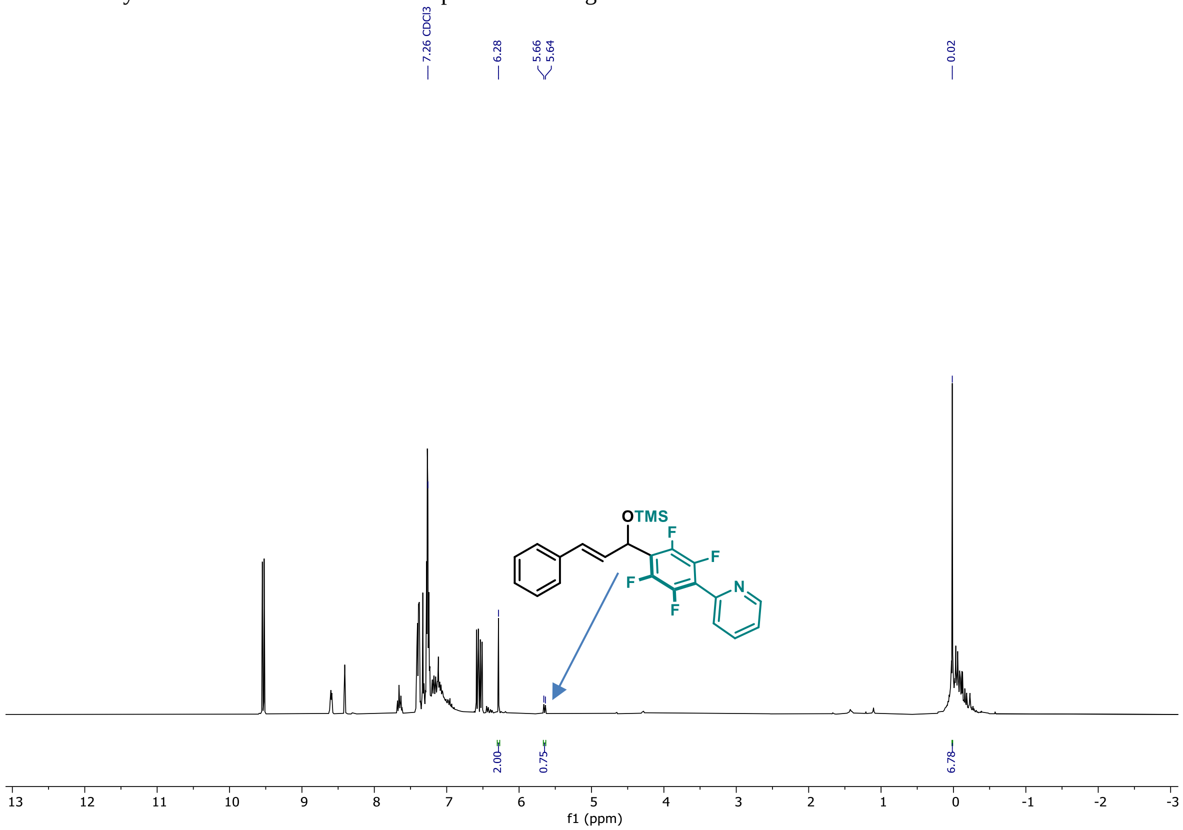

^{1}H NMR of 4w (300 MHz, $CDCl_3$, 298K)

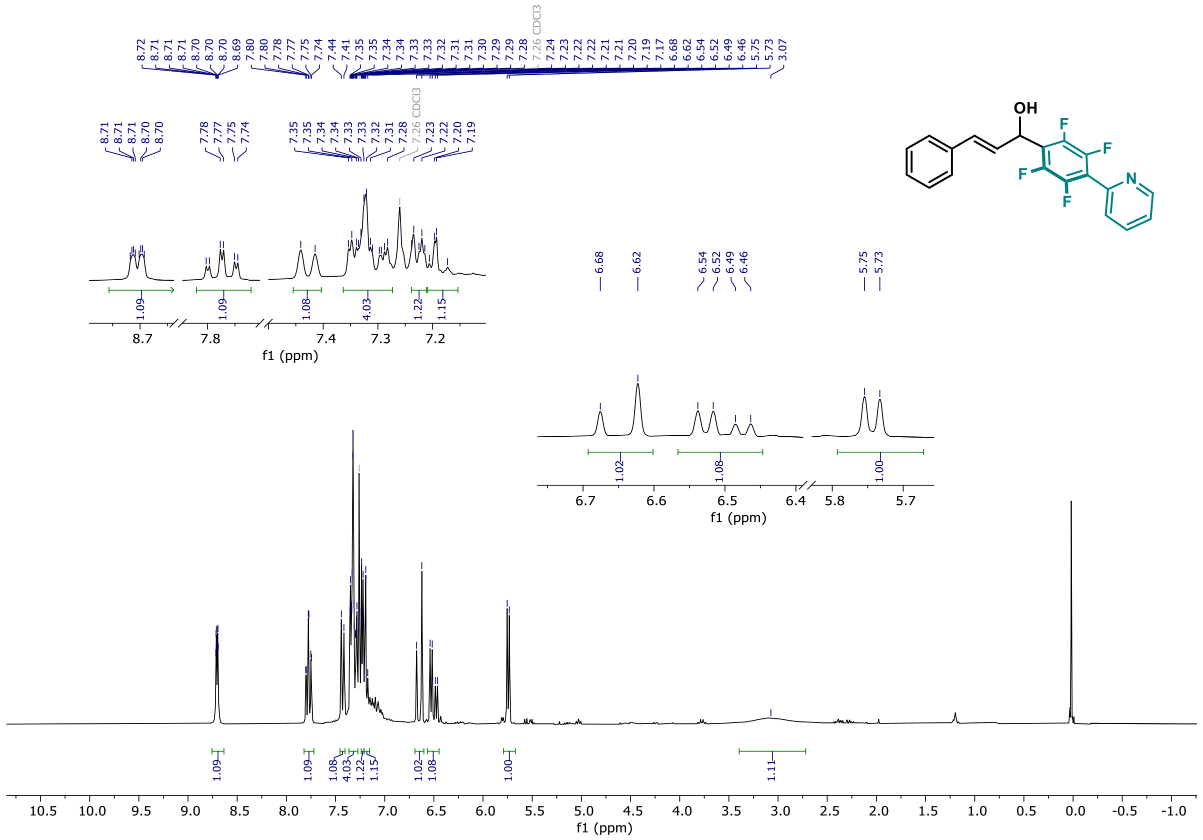

^{19}F NMR of **4w** (282 MHz, $CDCl_3$, 298K)

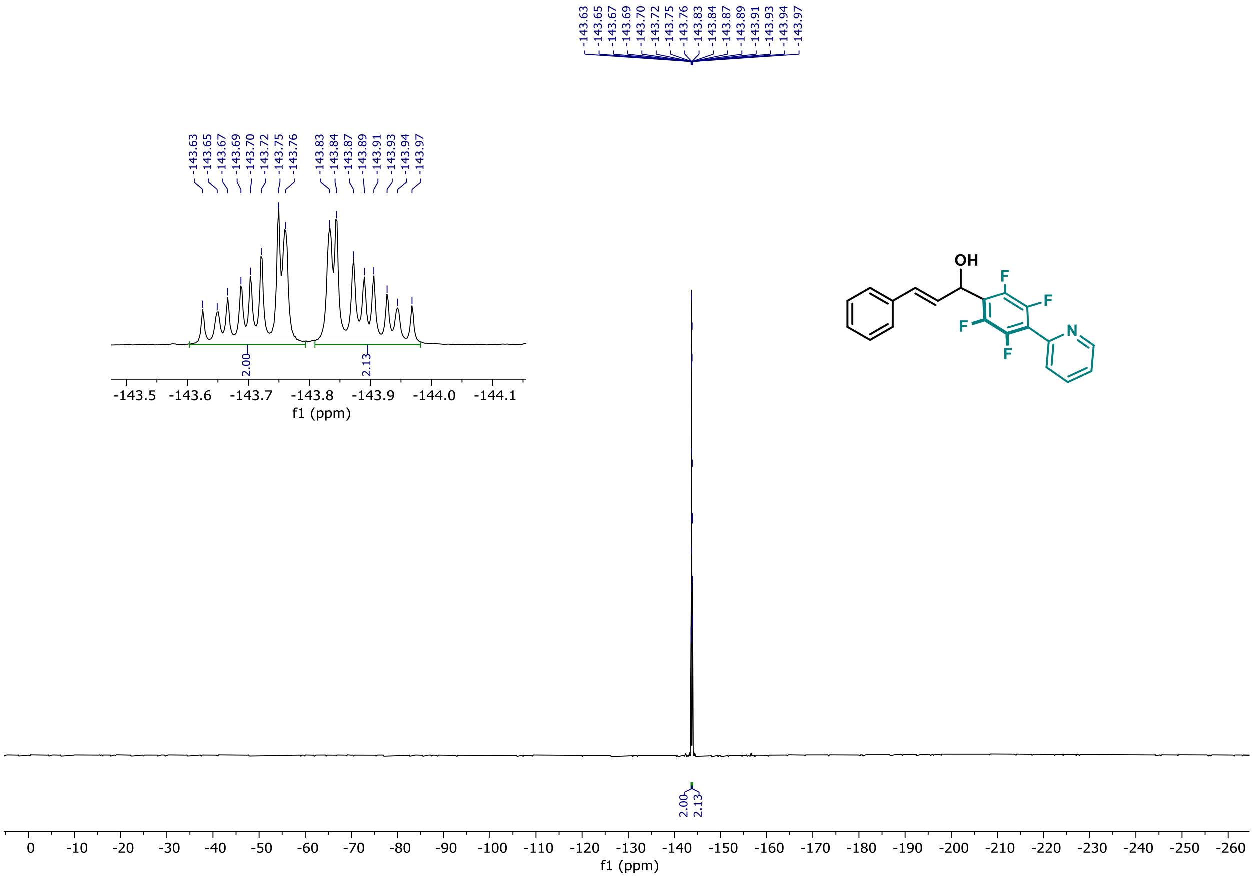

^{13}C NMR of **4w** (101 MHz, $CDCl_3$, 298K)

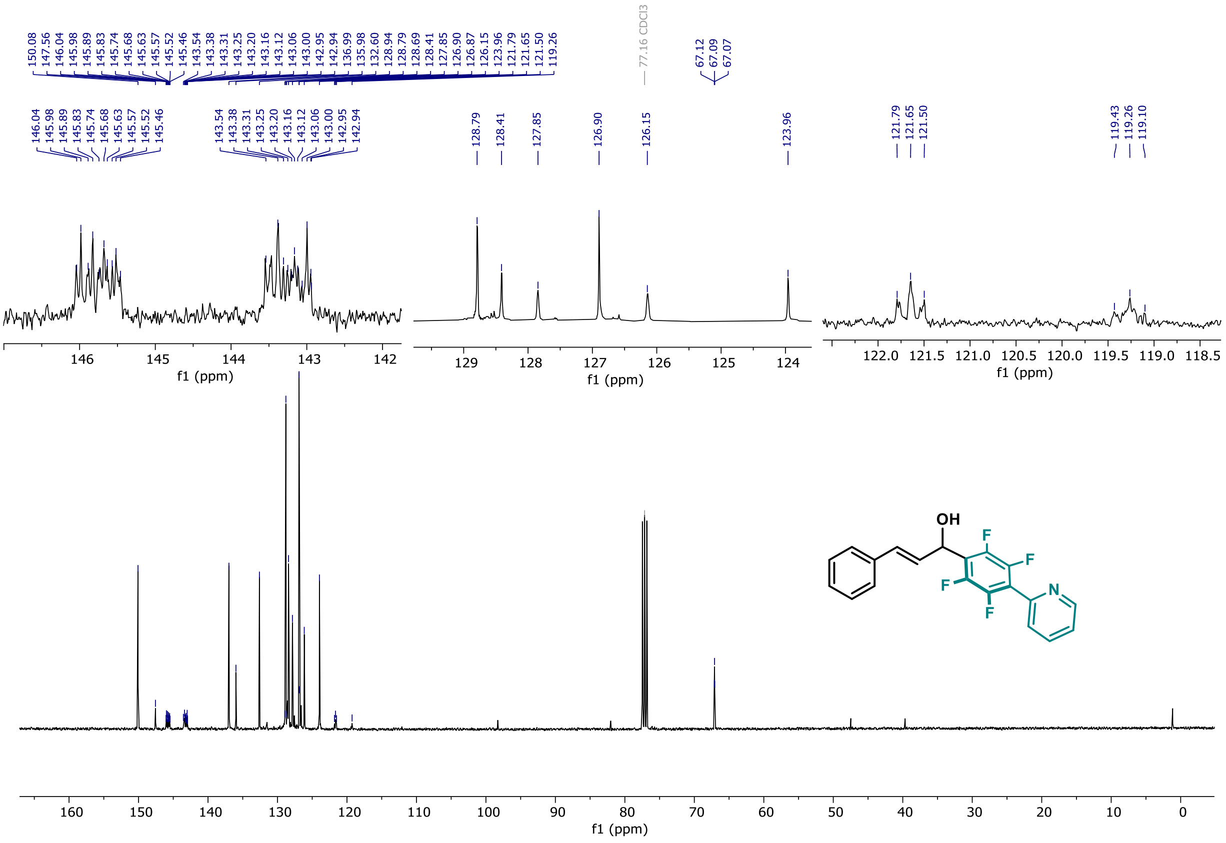

^{1}H NMR of **4x** (400 MHz, $CDCl_3$, 298K)

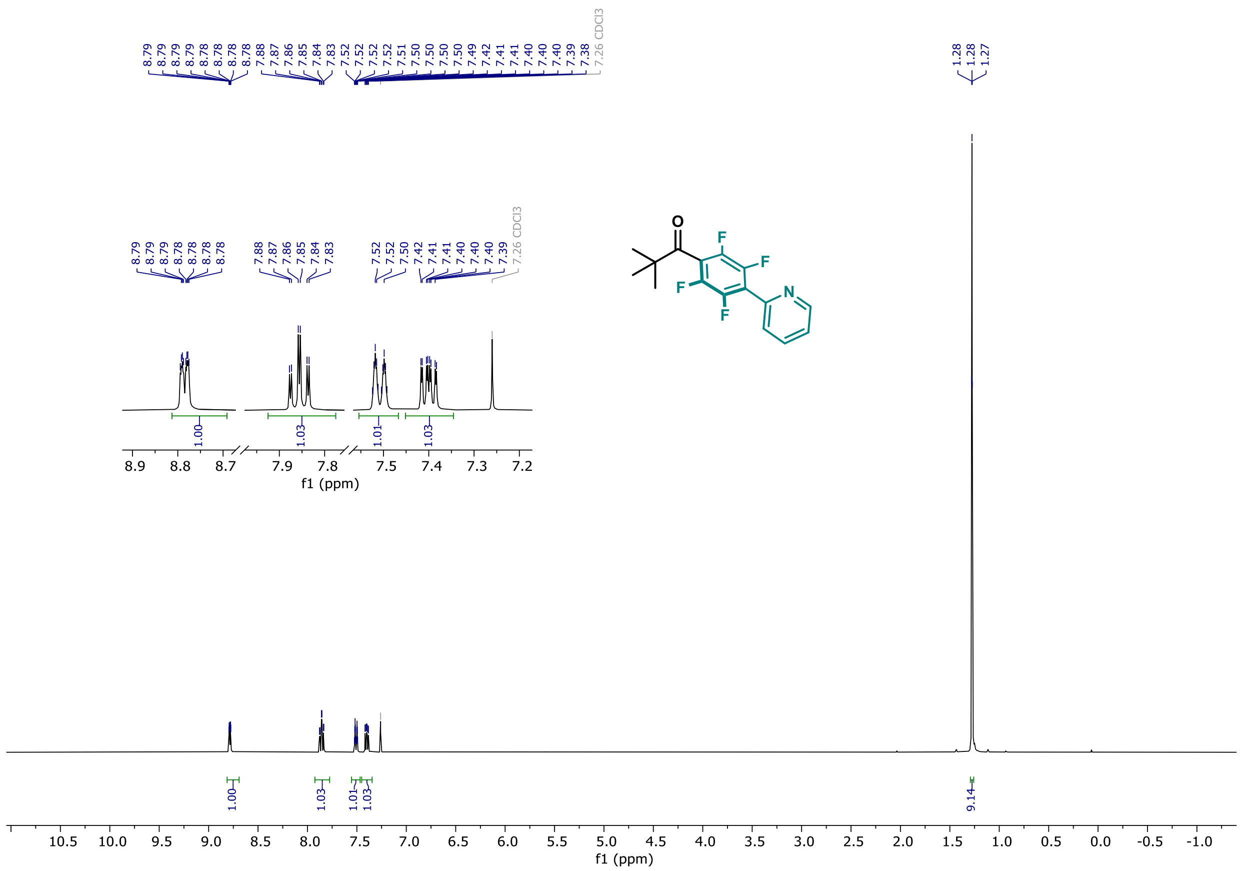

^{19}F NMR of **4x** (282 MHz, $CDCl_3$, 298K)

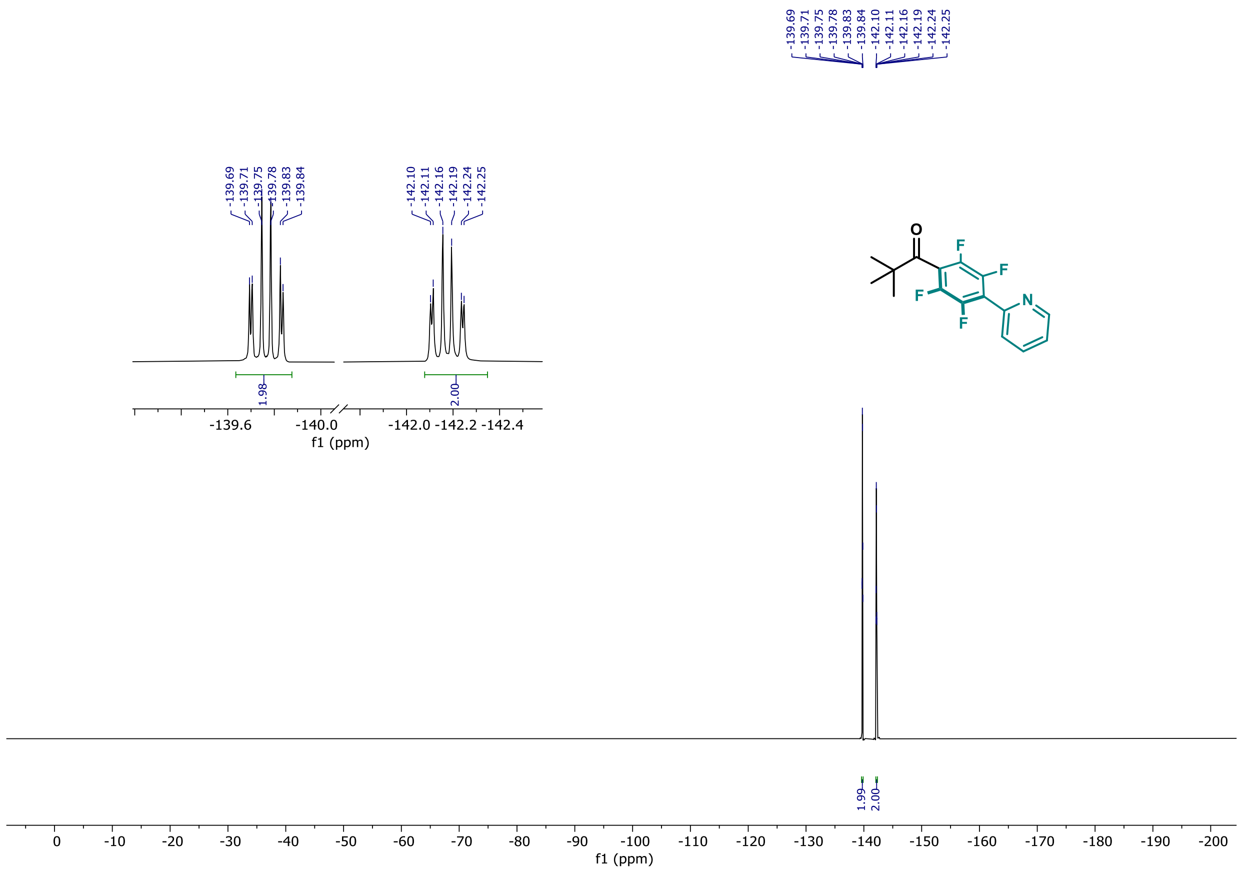

[13]**C NMR** of **4x** (101 MHz, $CDCl_3$, 298K)

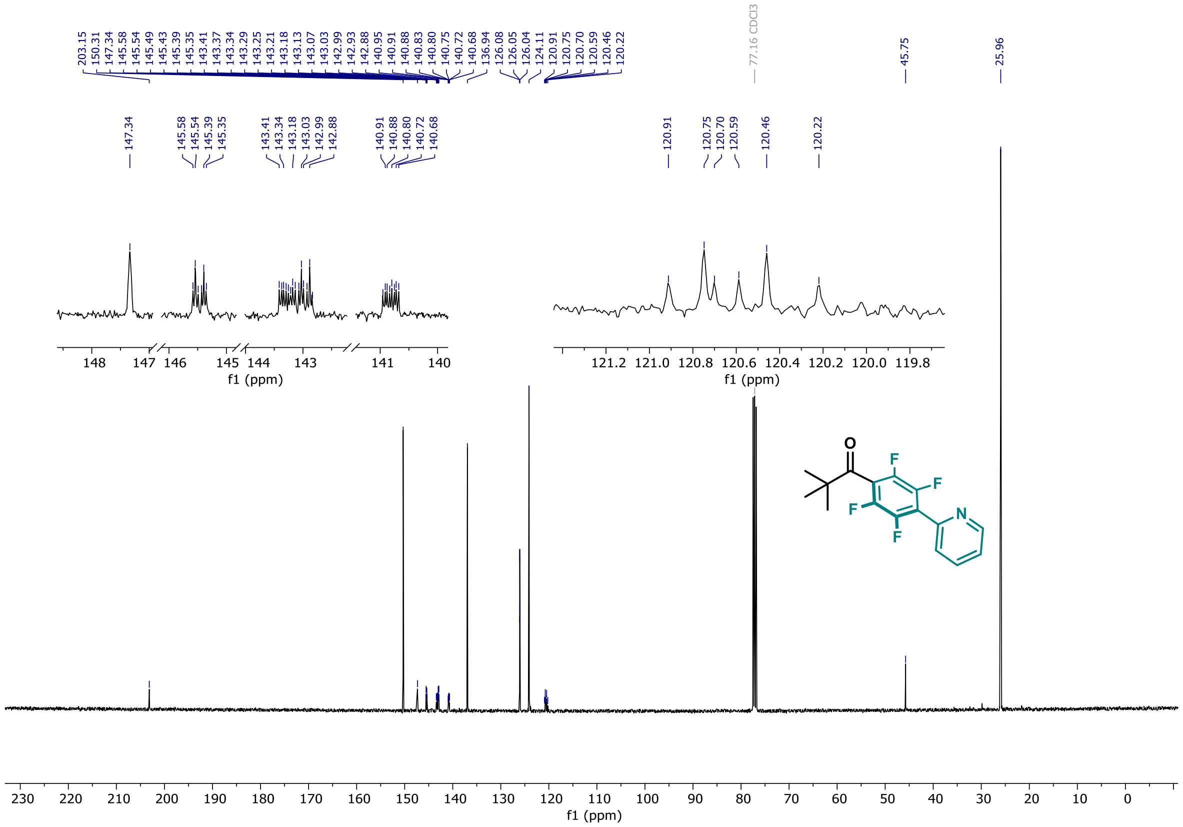

[1]**H NMR** of **4y** (600 MHz, $CDCl_3$, 298K)

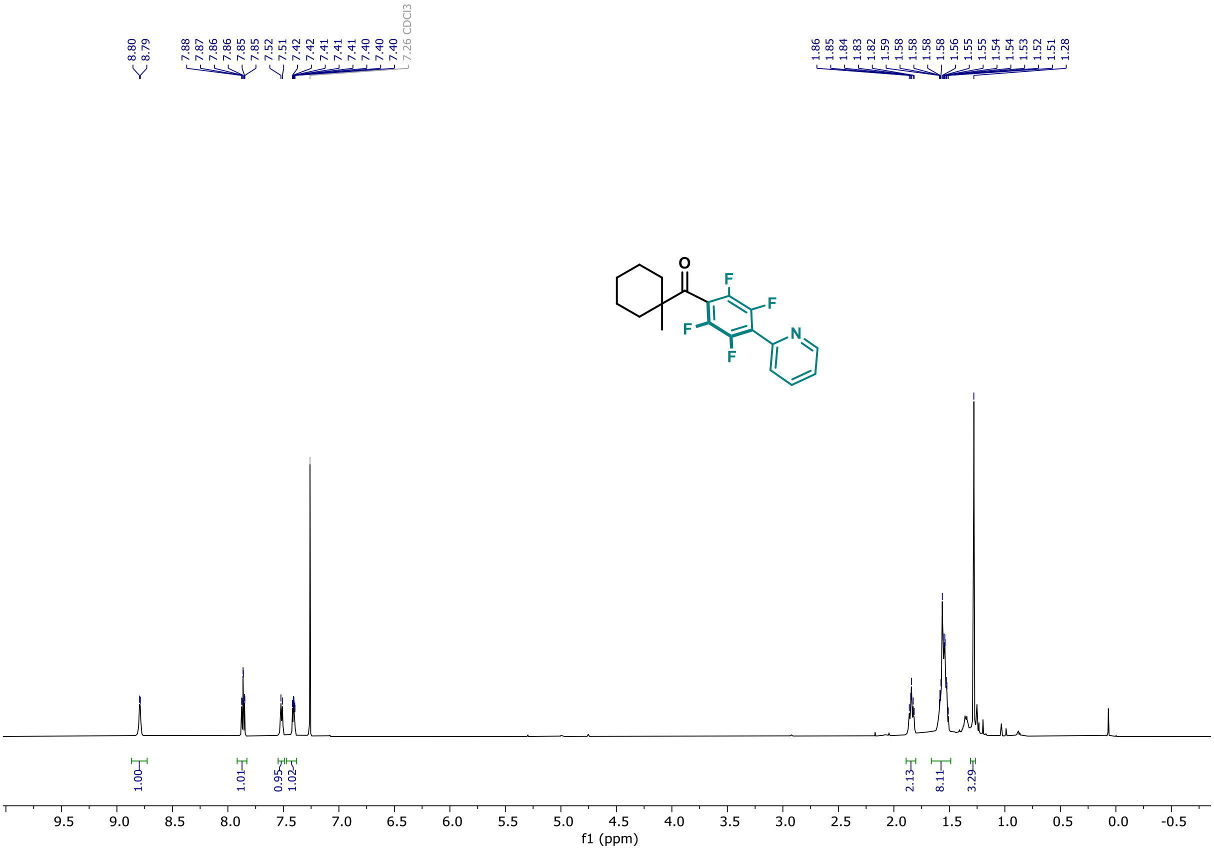

^{19}F NMR of **4y** (565 MHz, $CDCl_3$, 298K)

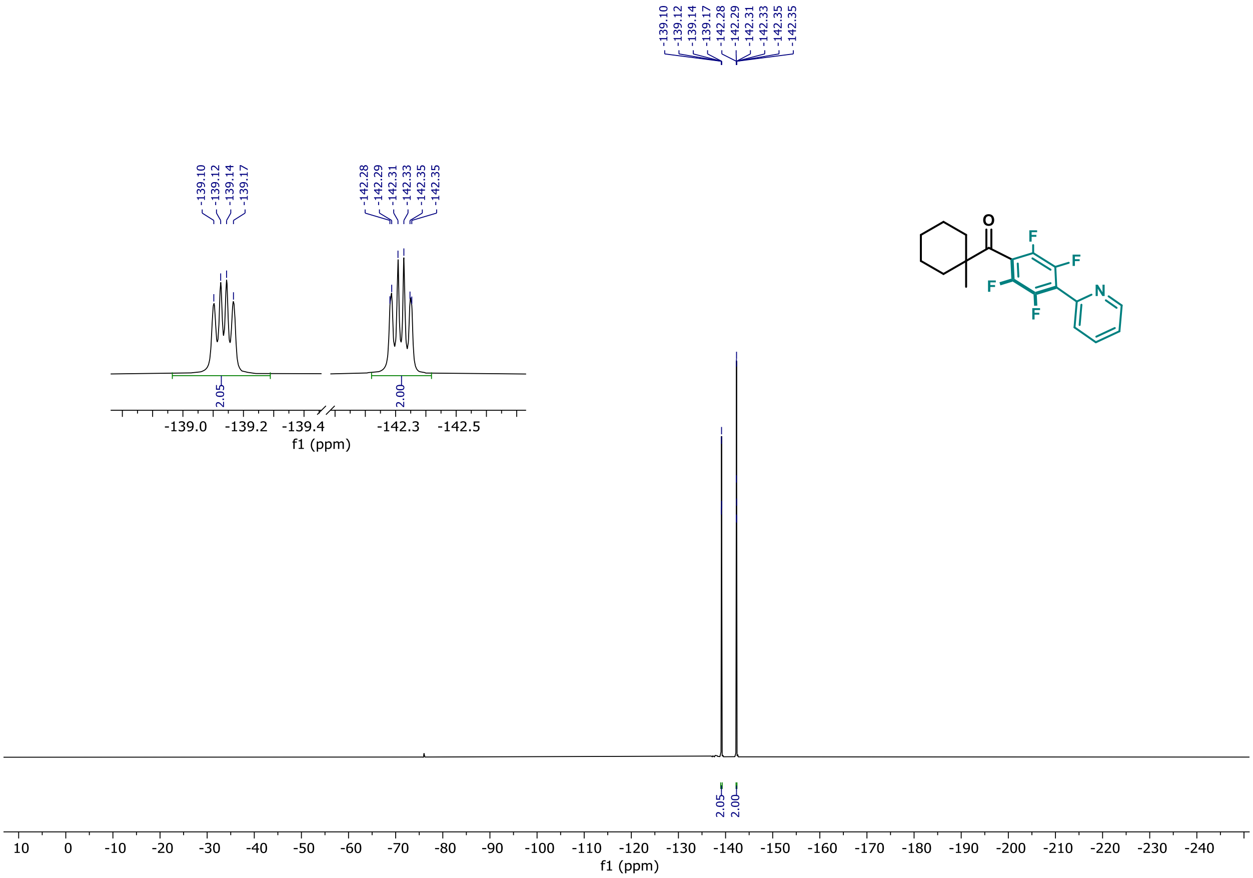

^{13}C NMR of **4y** (151 MHz, $CDCl_3$, 298K)

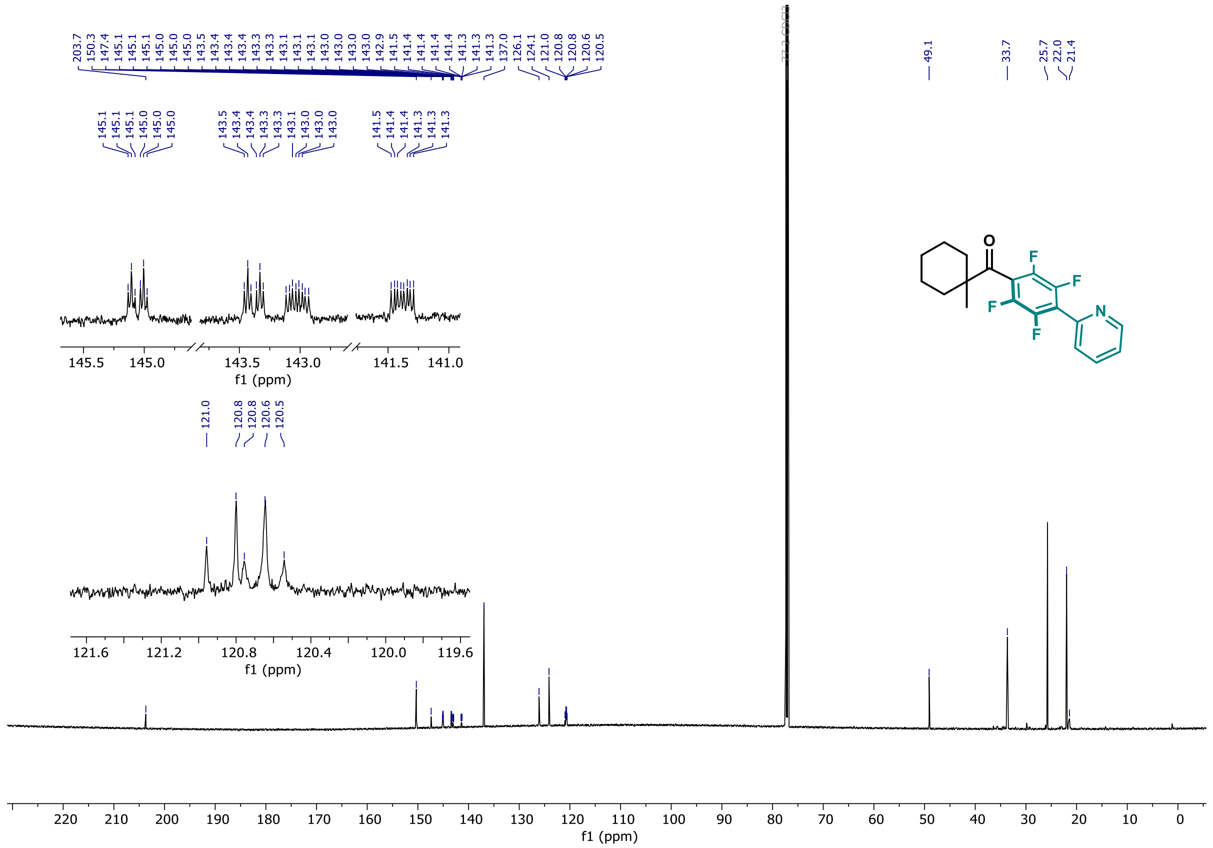

^{1}**H NMR** of **5e** (extracted) (600 MHz, CD_3CN, 298K)

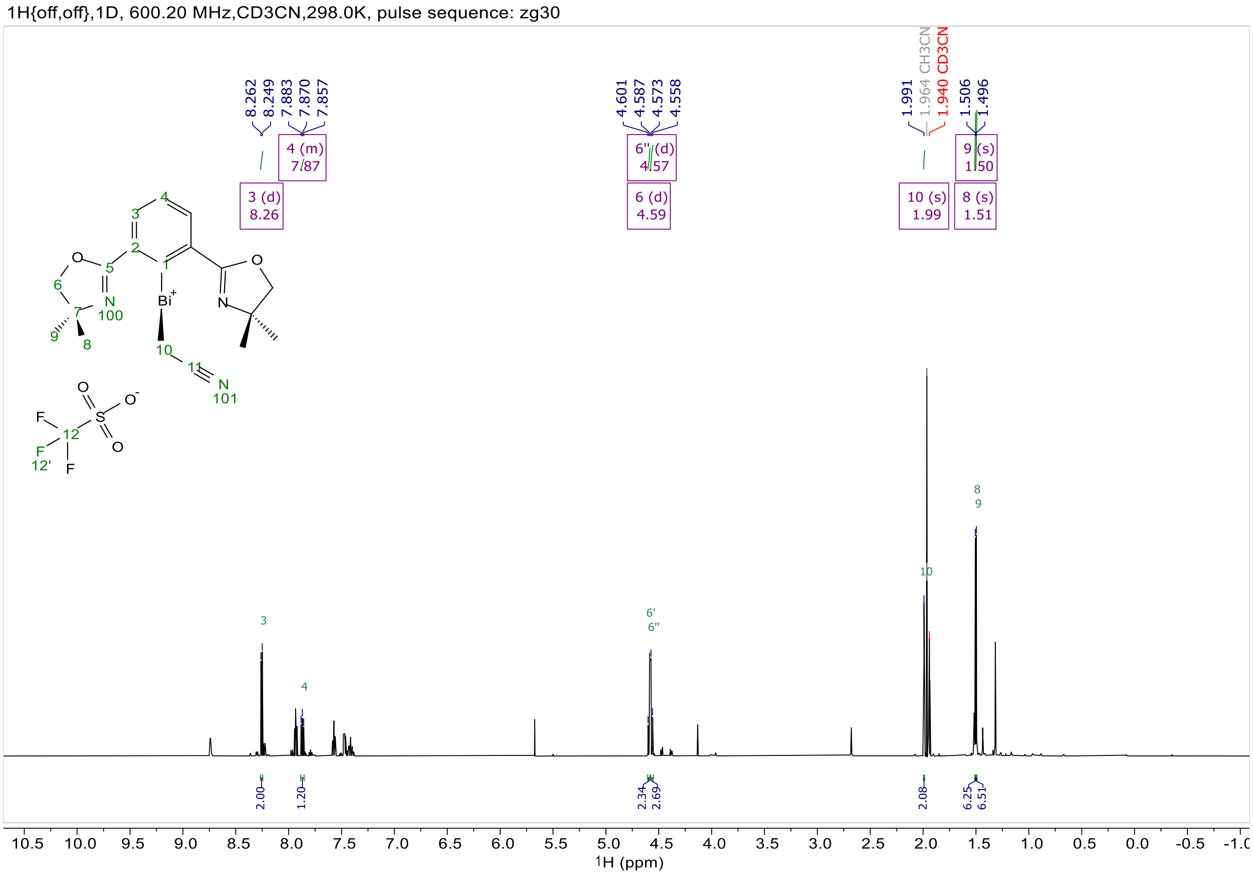

^{13}**C NMR** of **5e** (extracted) (600 MHz, CD_3CN, 298K)

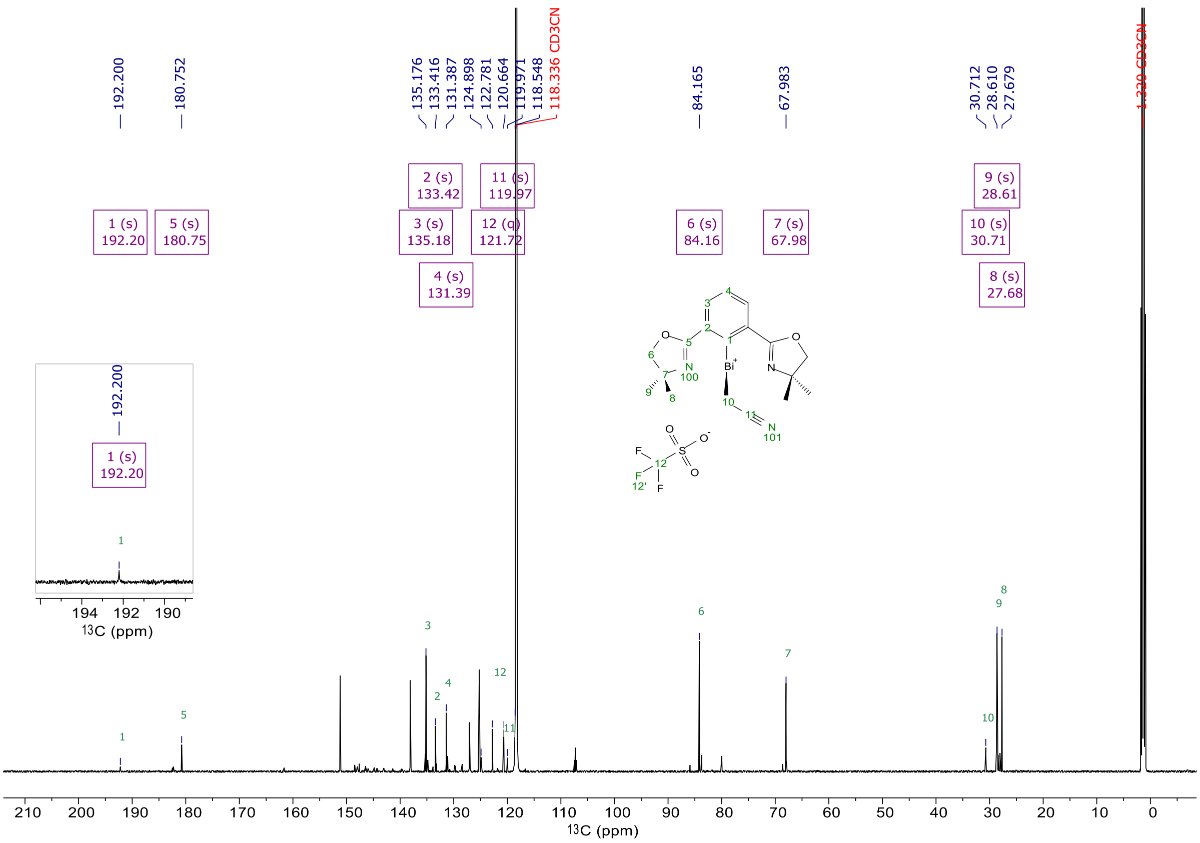

^{19}F NMR of **5e** (extracted) (600 MHz, CD_3CN, 298K)

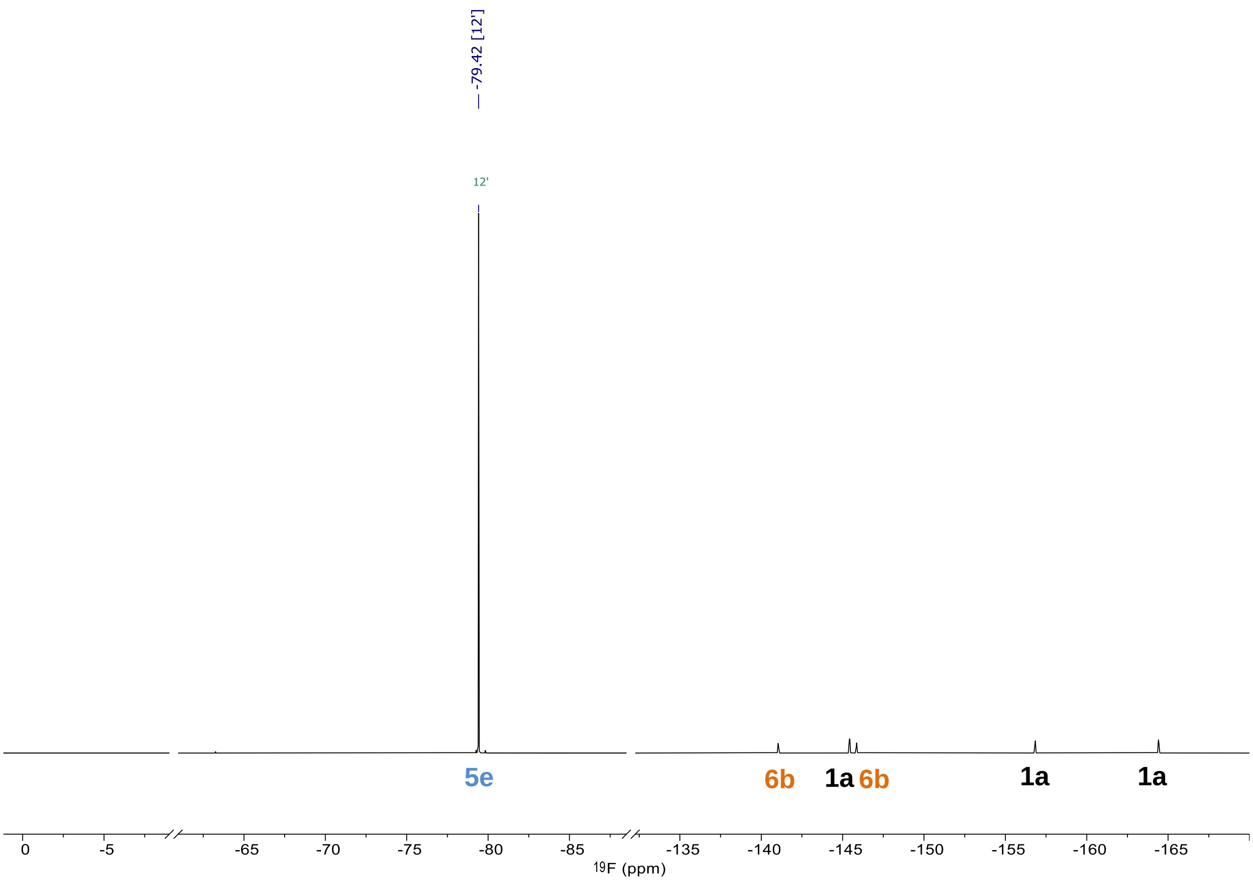

1H{off,off},COSY, 600.20 MHz,CD3CN,298.0K, pulse sequence: cosygpppqf

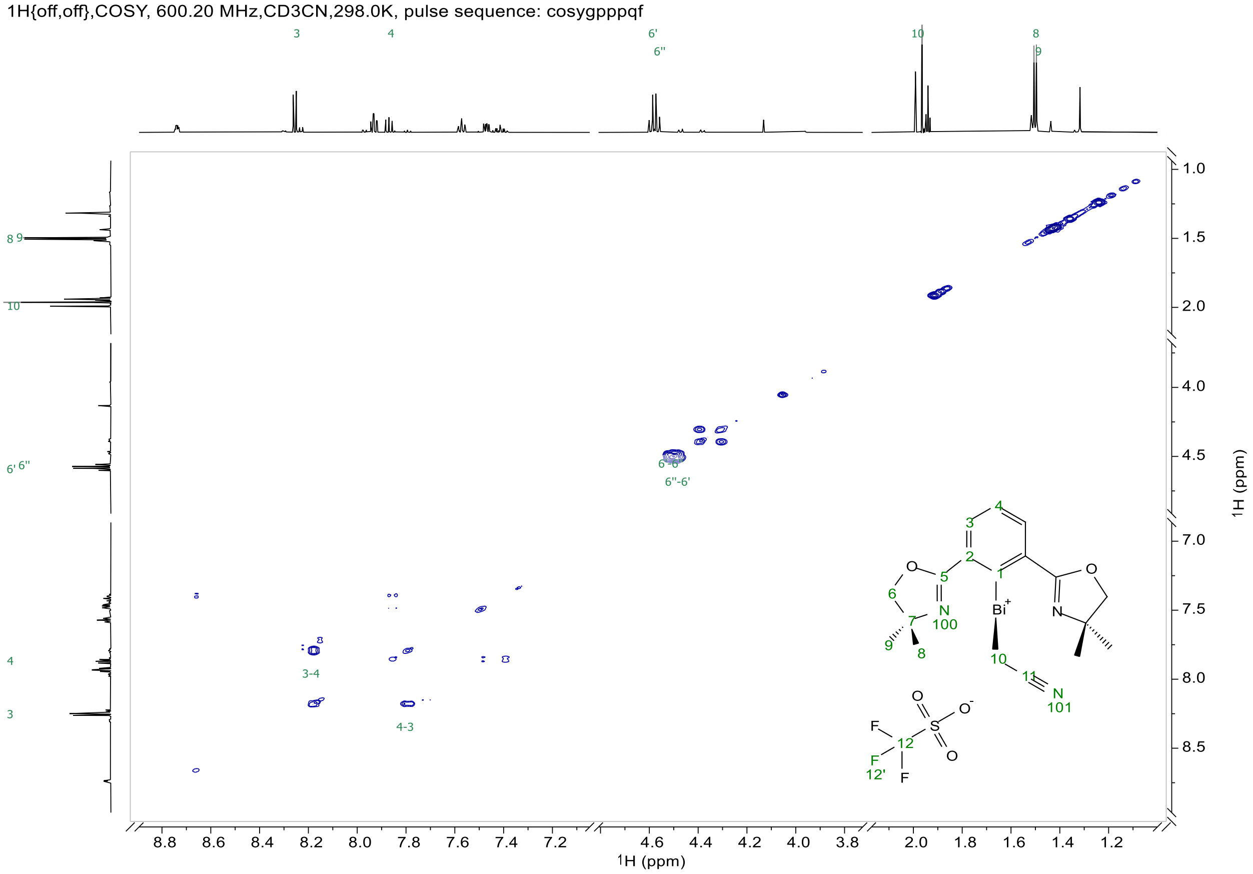

1H{off,off},NOESY, 600.20 MHz,CD3CN,298.0K, pulse sequence: noesygpphpp

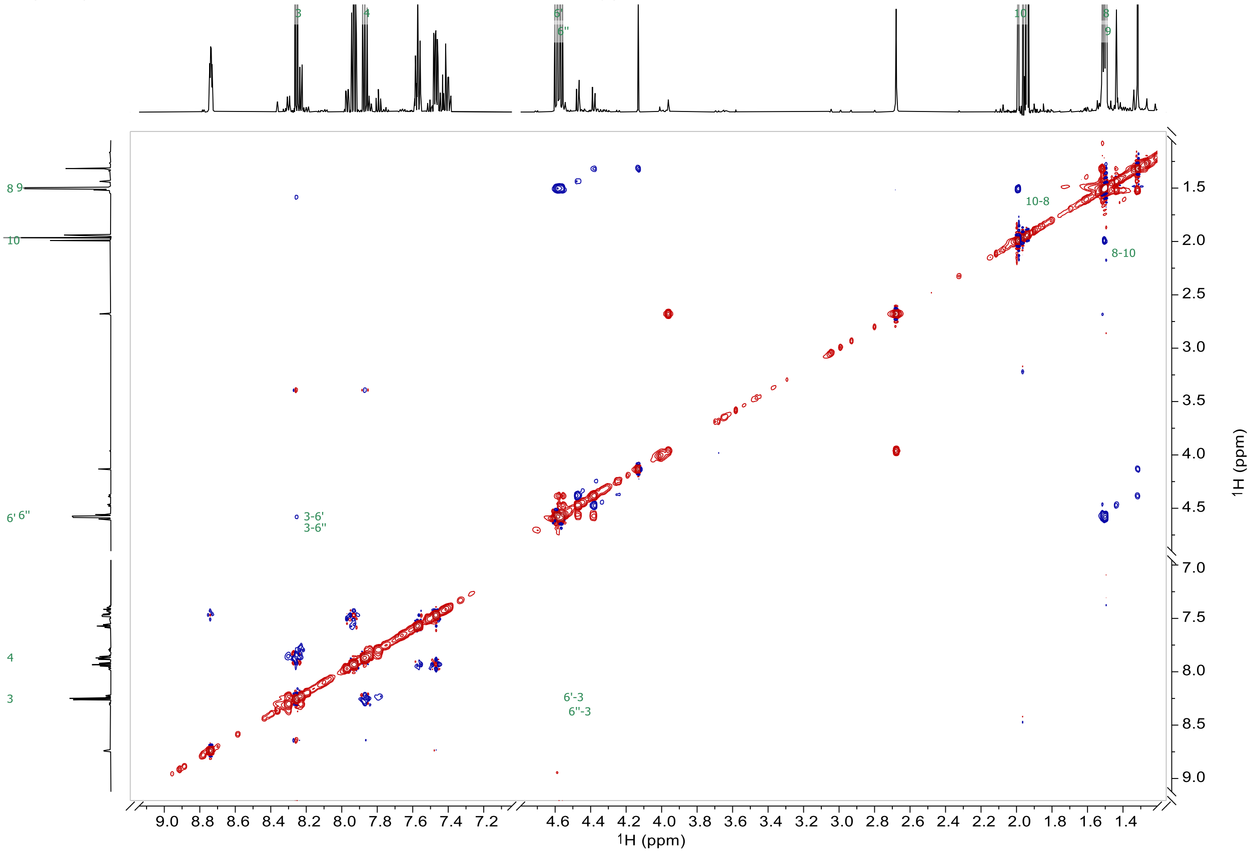

1H{15N,off},HMBC, 600.20 MHz,CD3CN,298.0K, pulse sequence: hmbcgpndqf

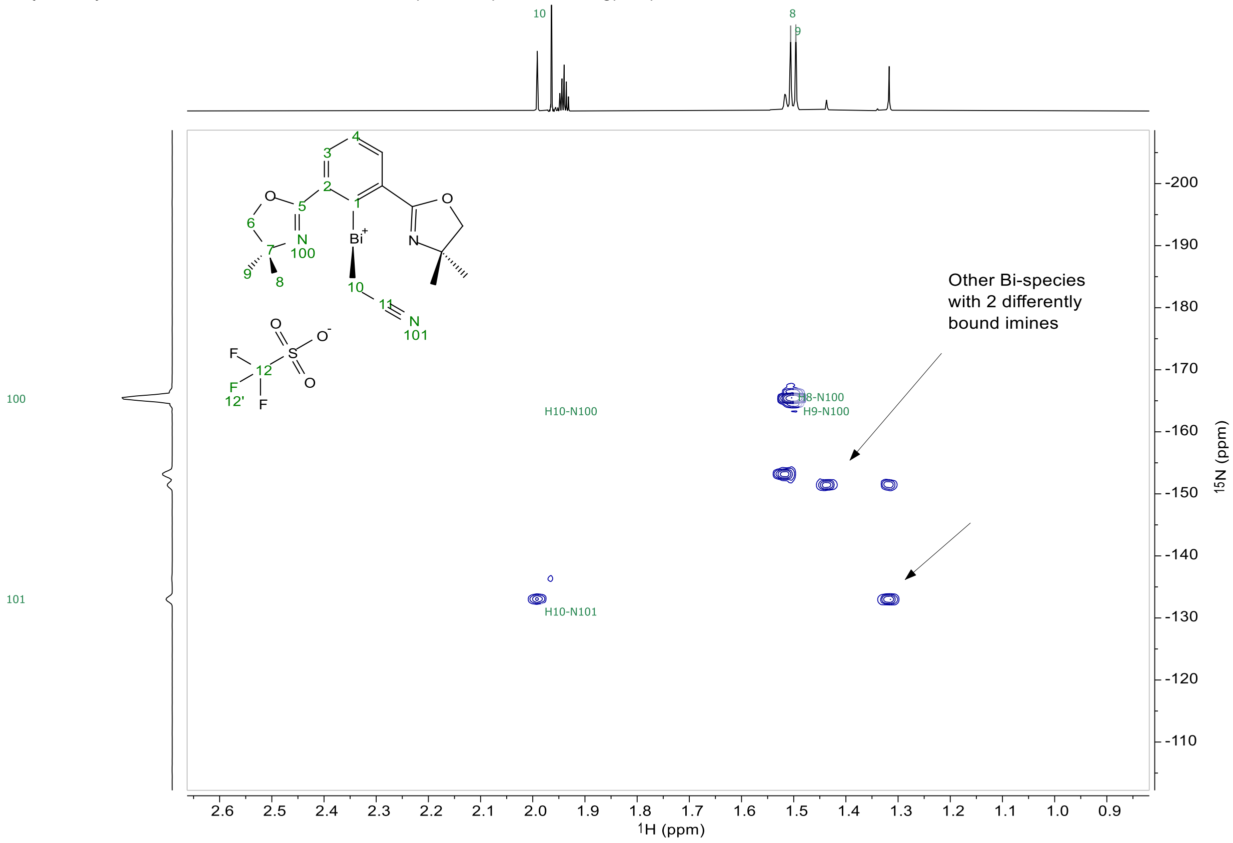

^{1}H-^{13}C HSQC of **5e**

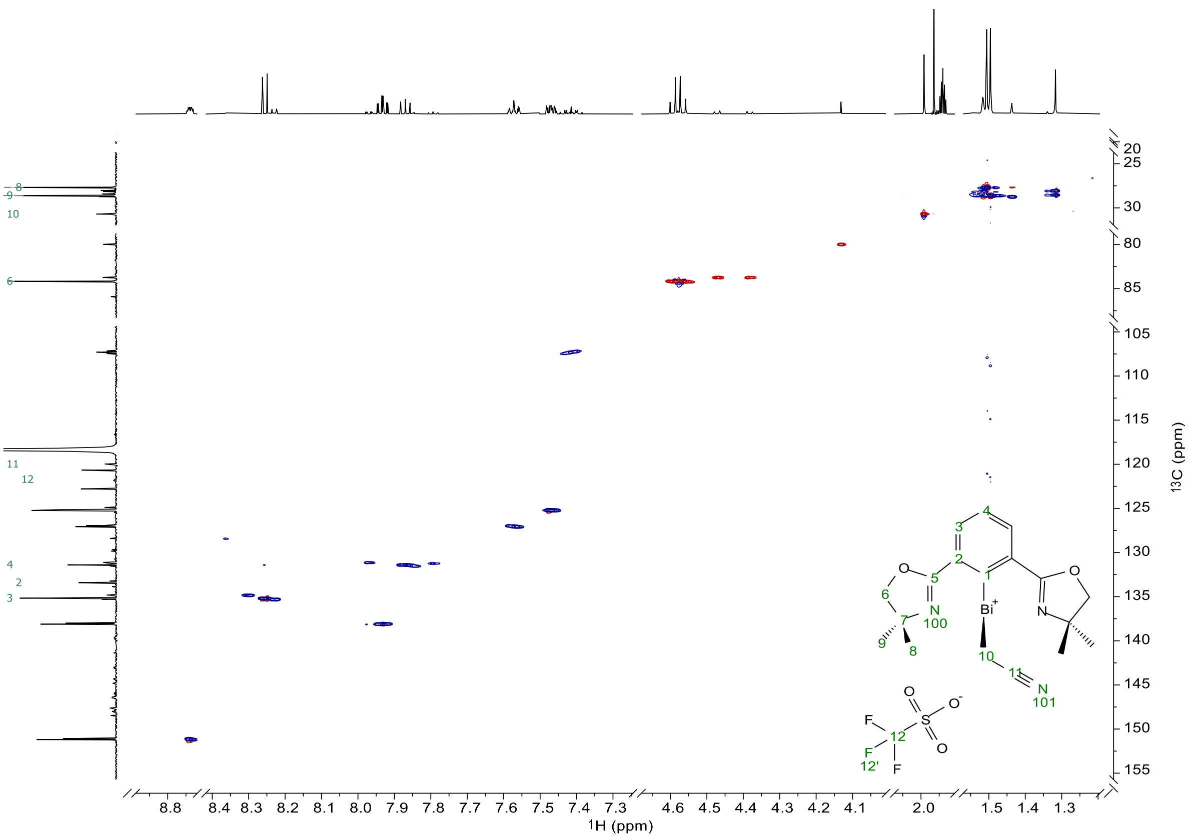

^{1}H-^{13}C HMBC of **5e**

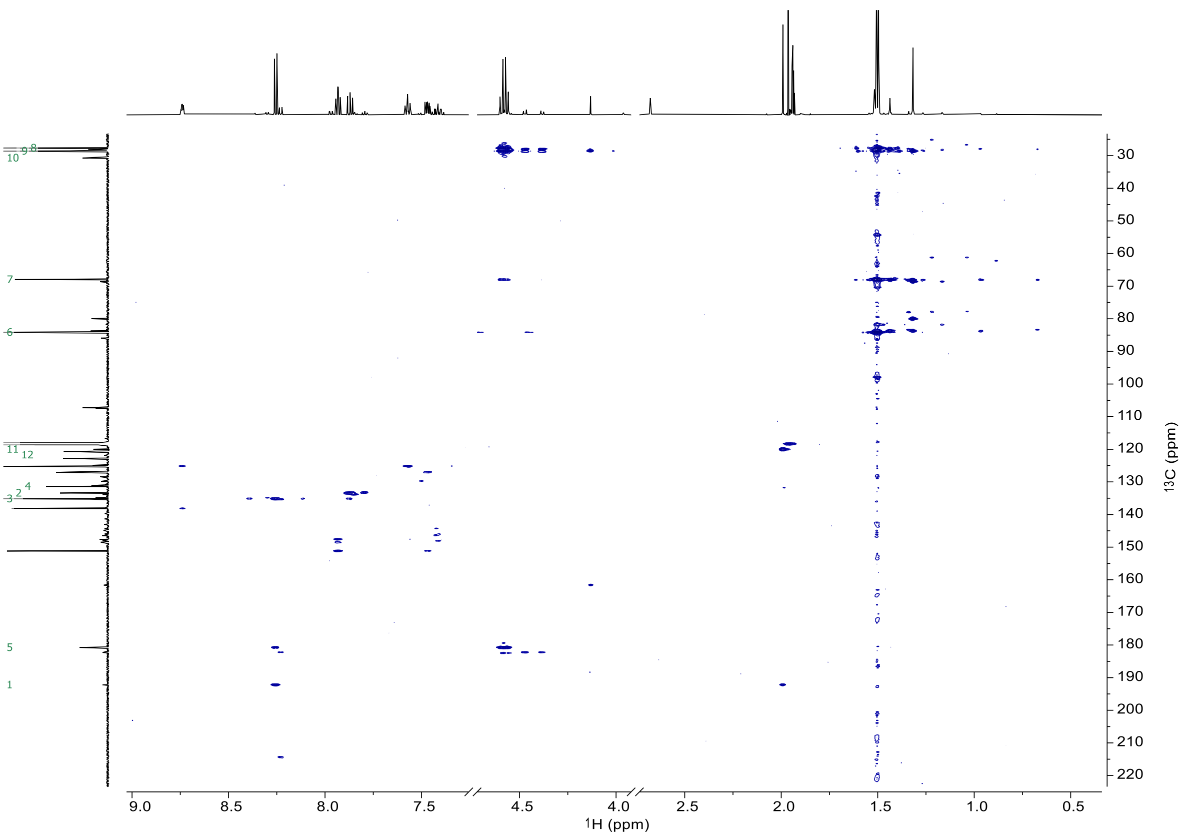

^{1}H NMR of **5i** (400 MHz, THF-d$_8$, 298K)

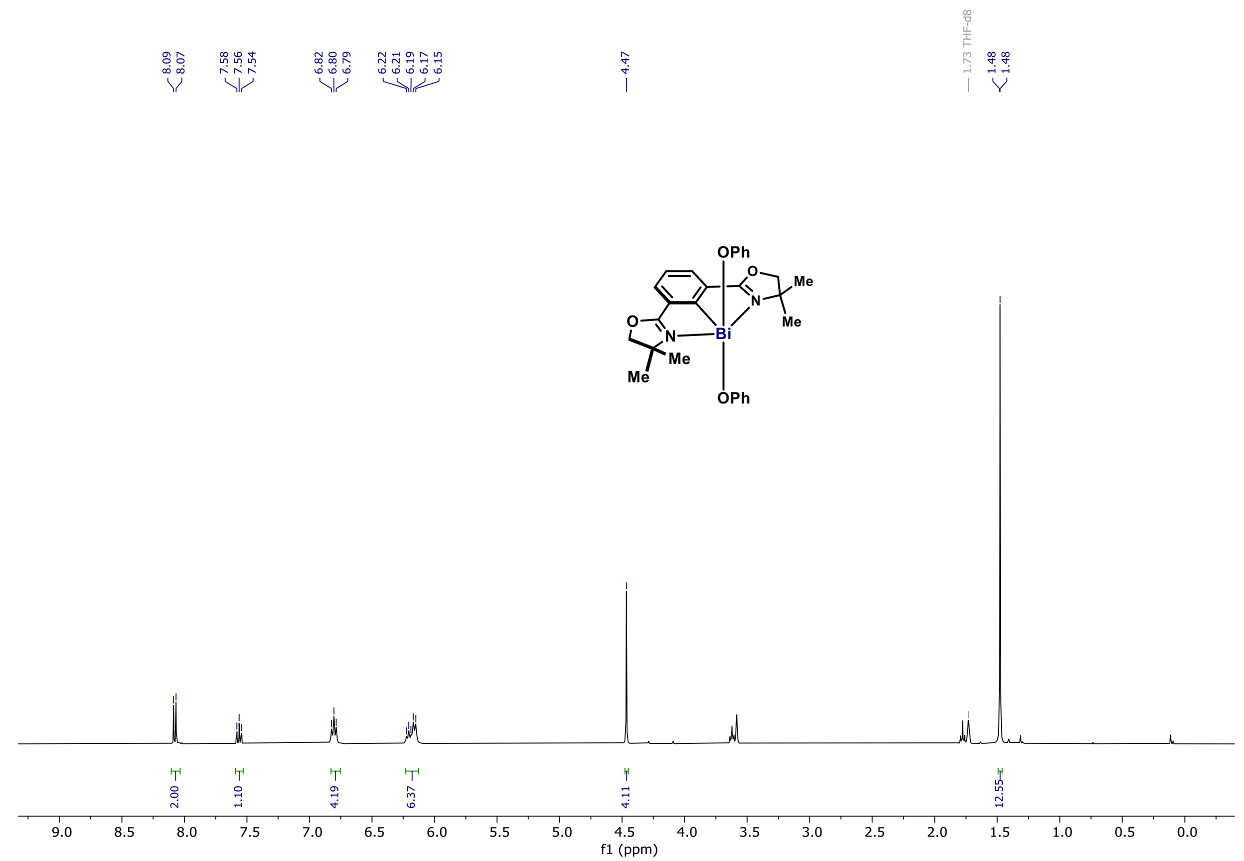

^{13}C NMR of **5i** (101 MHz, THF-d$_8$, 298K)

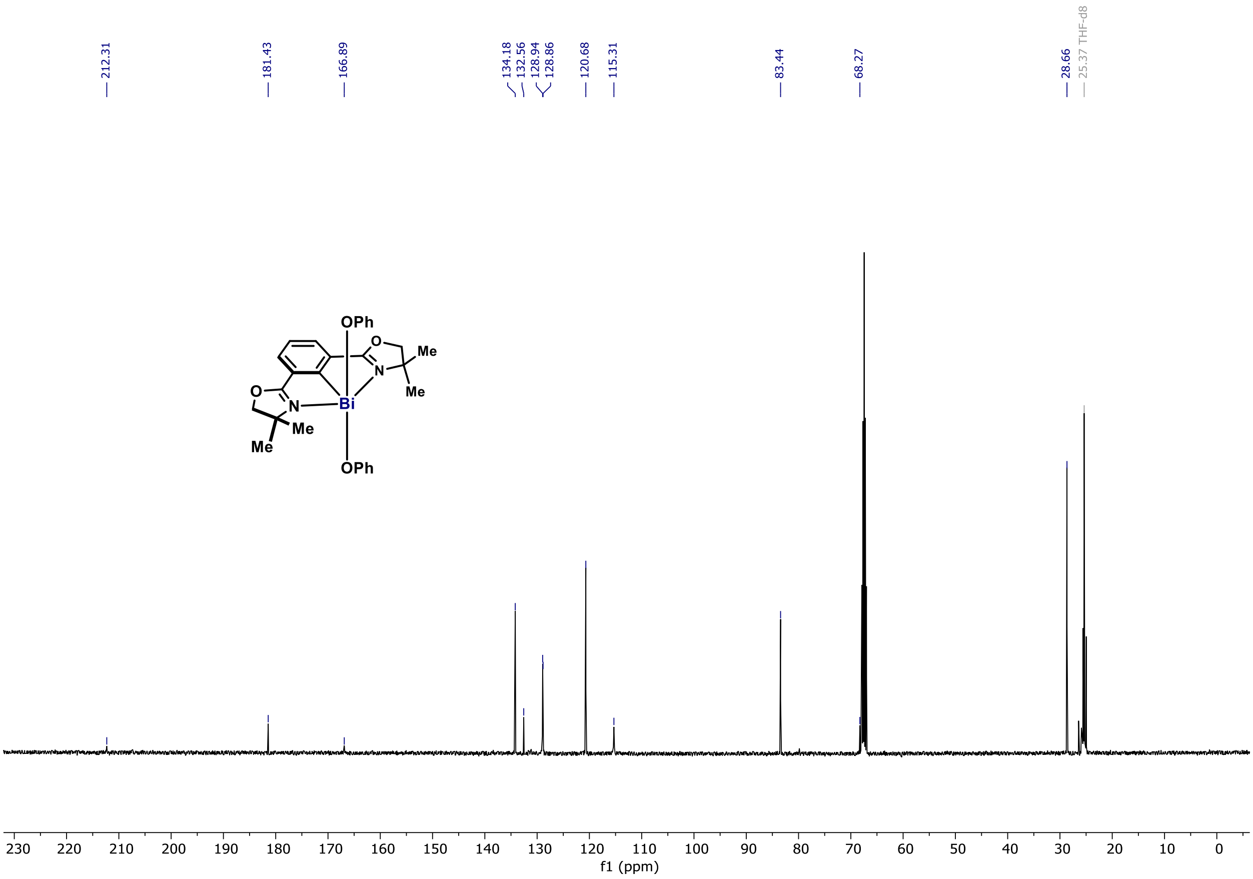

^{1}**H NMR** of **6c** (600 MHz, $CDCl_3$, 298K)

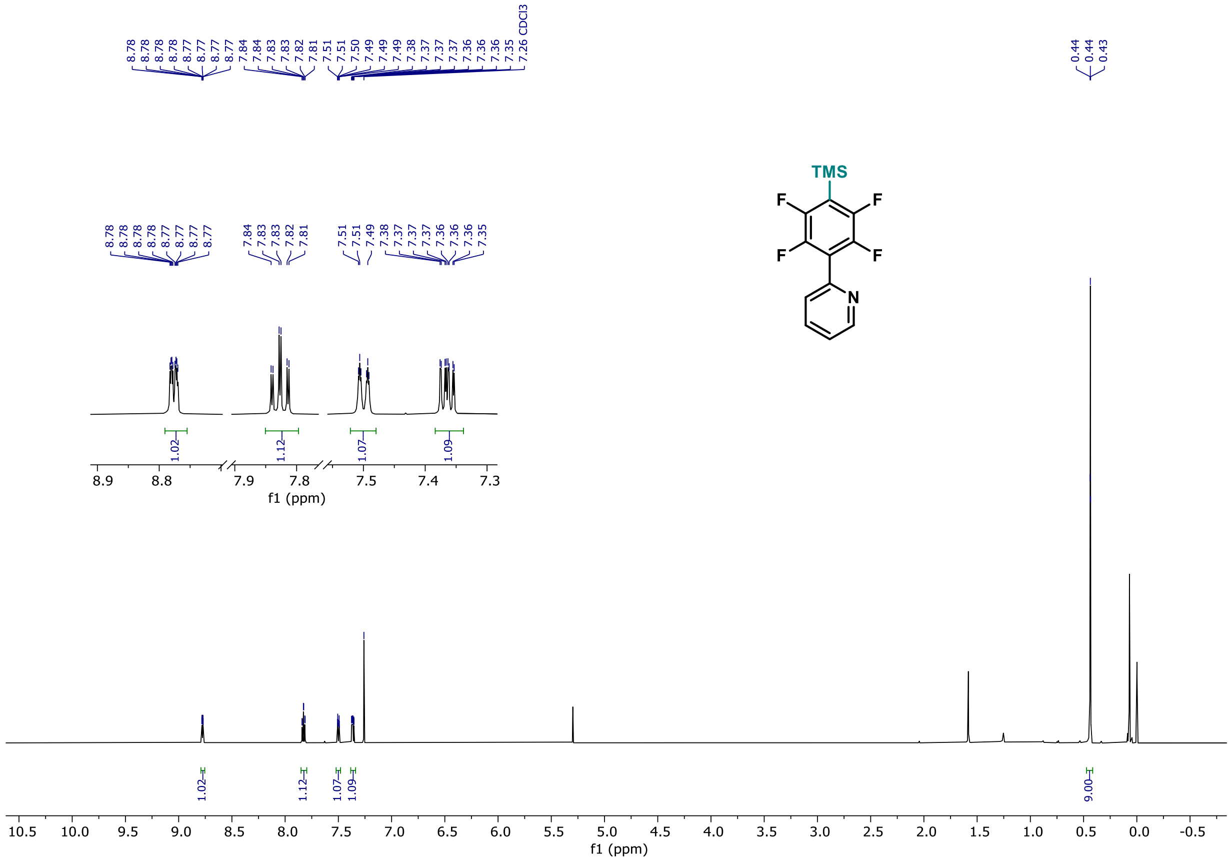

^{19}**F NMR** of **6c** (565 MHz, $CDCl_3$, 298K)

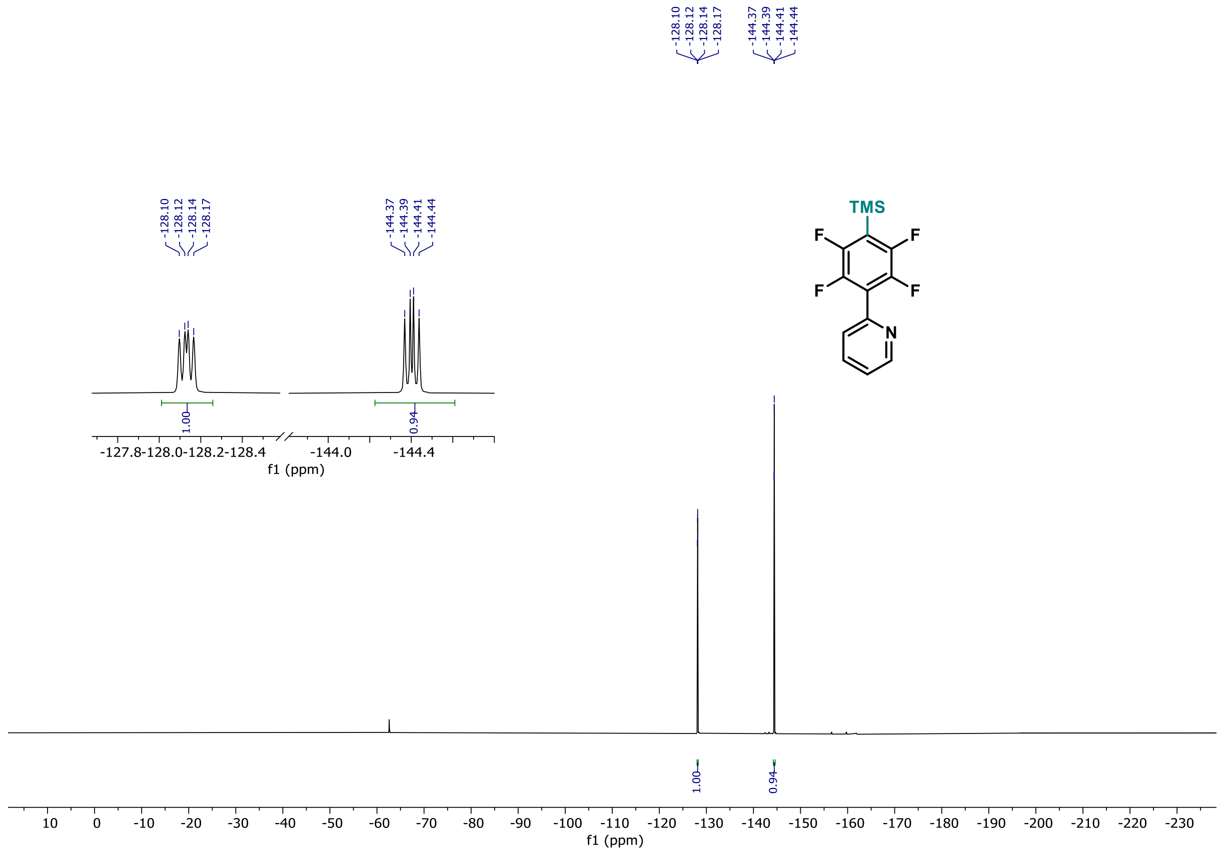

^{29}Si NMR of **6c** (119 MHz, $CDCl_3$, 298K)

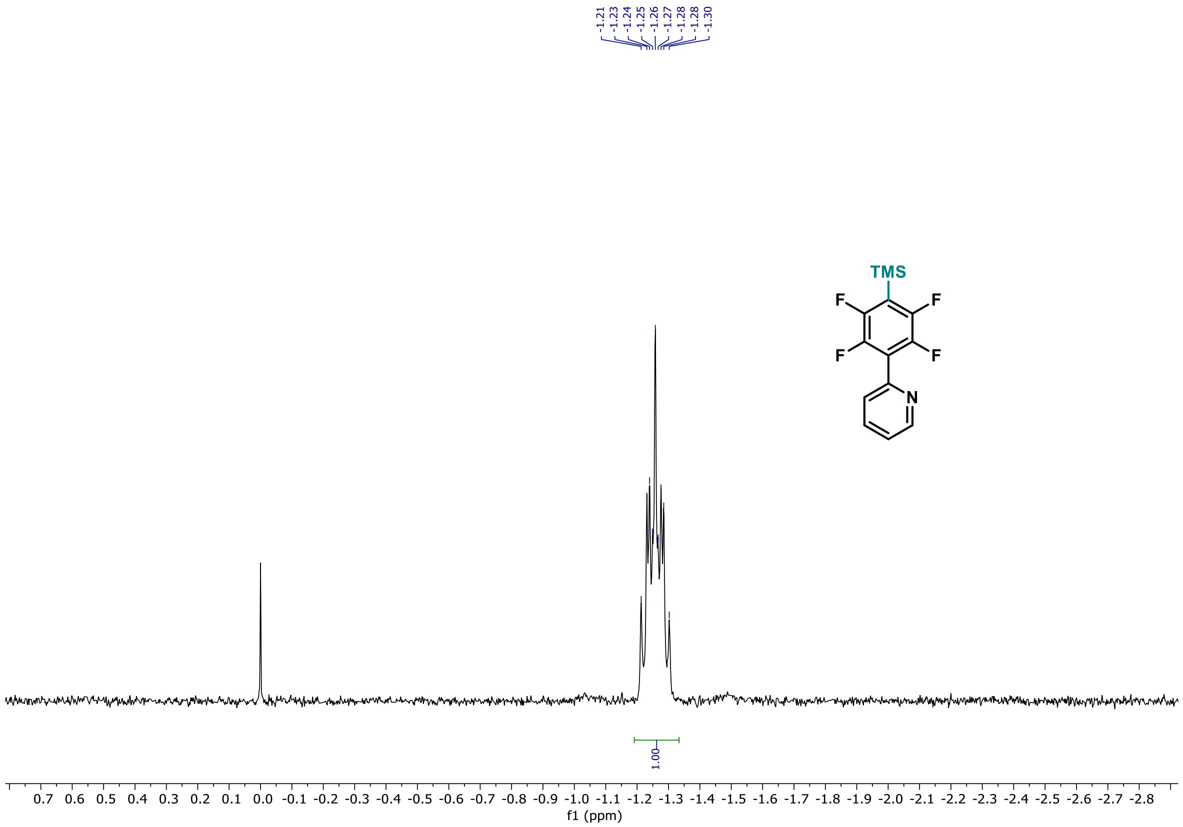

^{13}C NMR of **6c** (151 MHz, $CDCl_3$, 298K)

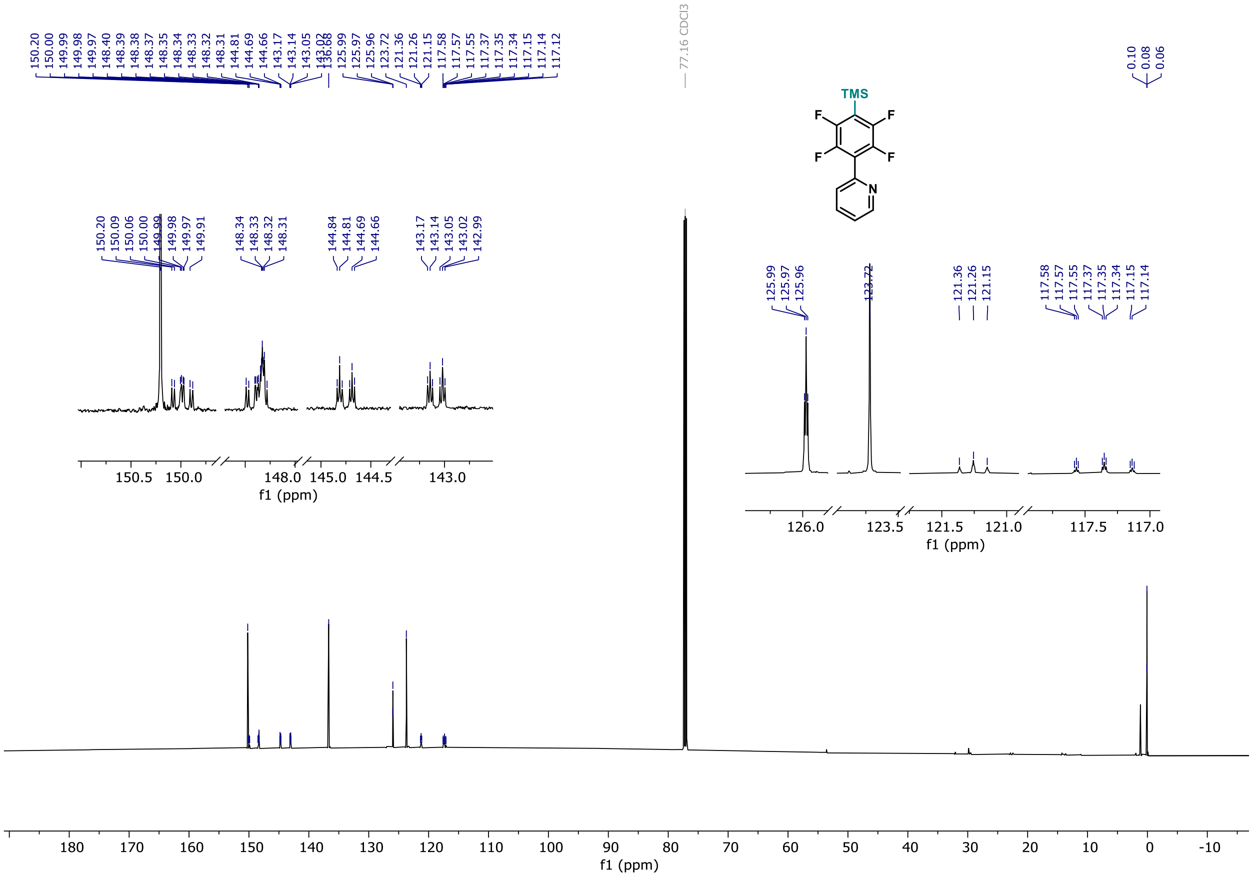

8. Cartesian coordinates

Cartesian coordinates of the stationary points are given in Å, and the absolute thermochemical data are given in Hartrees (Temperature = 298.150 K; Pressure = 1.00000 atm).

5h

Electronic Energy (Def2-SVP) = -2377.14661018

Total enthalpy (Def2-SVP) = -2376.53680058

Gibbs Free Energy (Def2-SVP) = -2376.64905402

Electronic Energy (Def2-TZVP) = -2379.30562349281

Bi	1.11578591598493	10.75654145315859	3.78441989235614
F	0.81284122552726	13.00940780083450	1.14840385002961
F	0.08690965048371	15.54973284219567	0.94739666640544
F	-0.29923282954340	15.80551812790076	5.65260316727118
F	0.44364193566977	13.26781542651629	5.84765787647124
O	3.68703348381679	13.54068590881657	0.60747891096429
O	1.78898289340610	6.75479125443602	1.82629390574181
N	3.79612624343486	13.13119540669479	2.81293491002851
N	1.61221739701705	8.07223953728319	3.62694442059716
C	-0.13899284274832	15.77457841362219	3.29180325116776
C	2.57405225289291	10.34923807744449	2.02881468638265
C	3.30761312469659	11.27633378390310	1.26244115479198
C	3.95122032069794	10.87446012949478	0.07885333089148
H	4.49261268449172	11.62371255716971	-0.50123953346635
C	3.93060935836274	9.55141003252587	-0.34423469941088
H	4.44145461361288	9.25779098929202	-1.26364398486079
C	3.27851374550030	8.60731076798156	0.43457275937279
H	3.27557051969087	7.55361268088176	0.15034885665850
C	2.61536771317141	8.99993787493652	1.60669942123735
C	3.56010853821204	12.68108895470579	1.64671701742673
C	3.89800098600254	14.83000650890364	1.18543018144293
H	2.95956381092756	15.40276436922706	1.10095583895004
H	4.69036414067006	15.34712956193694	0.62737687273411
C	4.26607796931383	14.52062332936859	2.65210564978161
C	5.78064431491862	14.51754072583331	2.85989925354244
H	6.02441426087174	14.14915801020490	3.86765324012997
H	6.18601547030079	15.53541768154136	2.75098470519797
H	6.27393399553173	13.86492151120249	2.12312768613528
C	3.59505898822507	15.47728471997411	3.62441454639910
H	2.50345468110346	15.44527137806086	3.51969474659920
H	3.92982391382269	16.50794342113836	3.42937915821928
H	3.85222986588295	15.22539599597632	4.66305971329927

C	1.98862229728162	7.94577641369320	2.41057447973066
C	1.31896778687473	5.87695063329558	2.85775399597509
H	2.14246505233901	5.19977638571130	3.13543875590291
H	0.47957449719634	5.28958957653225	2.46317510252882
C	0.92661820622569	6.82681756503173	4.01325477748881
C	-0.57812071901057	7.08811993552755	4.04607783412442
H	-0.81294647914617	7.87138476226225	4.78193356466370
H	-1.11812581004097	6.17076947086367	4.32687003058607
H	-0.93873013047197	7.41840638577466	3.05990048738523
C	1.42032804384220	6.31558599358722	5.35758031813158
H	2.51674133581113	6.22758015334855	5.36292867480638
H	0.98873643916238	5.32479008551677	5.56688270849526
H	1.11678432729836	6.99559297553642	6.16639119795614
C	0.72325321620847	13.05922336929234	3.50921322557114
C	0.57068417323529	13.68169928603646	2.28286324817196
C	0.17283186067728	15.00881498479198	2.16440369633437
C	-0.03597528655269	15.13811723069995	4.53255483080011
C	0.38126531527612	13.81254789580131	4.61927226548877
C	-0.54380028151351	17.19687146564570	3.17875622570627
C	0.22256292963719	18.09250082378427	2.42203362908238
N	-1.64913441580334	17.56883937996342	3.83257932230528
C	-0.18511160927463	19.42065697566802	2.34593144182077
C	-2.02861632113034	18.84247138851778	3.75528409462374
C	-1.33627127627992	19.80933606785917	3.02669793622101
H	1.12529975136269	17.75000620101836	1.91339205089677
H	0.39391162679661	20.14359667270236	1.76616686360198
H	-2.93863841261839	19.11447227393161	4.30232650099697
H	-1.69425140250459	20.84053624296470	2.99825570747993
Si	3.13661939902845	10.83550970049827	5.56941683716288
C	2.51799262010377	9.80804404436007	7.02429188664404
H	2.26717910952375	8.78436310287071	6.71068676126580
H	3.30021837877805	9.74994023247452	7.80008630923129
H	1.62153163740891	10.26174987130142	7.47587249125037
C	3.59356036244667	12.55047567770574	6.17733289000376
H	2.72455739081987	13.07562448164071	6.59773142663781
H	4.36838302160881	12.46223565101145	6.95759453516208
H	3.98952885524681	13.13606607047992	5.33746834447018
C	4.61730718878488	9.99980368416063	4.76496768118096
H	5.42846936880443	9.87417777065985	5.50184530084682
H	4.34115632134490	9.00930470365244	4.37245540705353
H	4.98844780327015	10.61404312764382	3.93114425872093

TS1’

Electronic Energy (Def2-SVP) = -2377.10653225

Total enthalpy (Def2-SVP) = -2376.49797658

Gibbs Free Energy (Def2-SVP) = -2376.60644802

Electronic Energy (Def2-TZVP) = -2379.2669196923

Bi	-0.82240575612461	-1.84786325297033	-0.76502479840186
F	-2.23306254784481	0.60867096295603	-0.60873981428329
F	-2.87901418272953	3.13344451424309	-0.82340240069191
F	-0.34802125886415	4.13912274304207	3.04348898146991
F	0.29393822130211	1.61893606712314	3.28647744396571
O	0.47364199497021	0.36274793375892	-1.80462159935771
O	1.12221629747689	-5.73902526747435	-0.58865993891559
N	2.52283391245570	1.27416519943043	-1.92403089822386
N	-0.40723841320151	-4.13244367278931	-0.54578829952347
C	-1.64848218385224	3.74860242182334	1.10318973111111
C	1.37769503933230	-2.14703106825500	-1.18930104814582
C	2.30002324597976	-1.13989611665240	-1.49339732677440
C	3.66571725122768	-1.44071303885513	-1.58380333599719
H	4.36771109478933	-0.63741746482380	-1.81870954628589
C	4.11943488639606	-2.74460113985449	-1.38618943138022
H	5.18591292377819	-2.96637955295432	-1.46030834773552
C	3.21378661291837	-3.76424329061263	-1.10343560219821
H	3.55511217681996	-4.79108582463321	-0.95418306449116
C	1.85089142579382	-3.45892764352504	-1.00462704773034
C	1.82707816468408	0.22658576024983	-1.74221571717049
C	0.24467719243185	1.66959728790400	-2.35312339432411
H	-0.62040762044846	2.11785171314235	-1.85543466315740
H	0.03305331520037	1.55285428649710	-3.42822086578509
C	1.57952027887539	2.39566588581901	-2.09686202390356
C	1.99330504235125	3.26826848472007	-3.27052797801785
H	2.96766275813046	3.73861897063804	-3.07160993773919
H	1.25250190176211	4.06570726585432	-3.43533356659471
H	2.07494985892575	2.67042530032669	-4.19044992016096
C	1.53547854260723	3.19641058085739	-0.79655131006752
H	1.16951214442009	2.57212348373223	0.03252552775896
H	0.85821934402327	4.05777256280456	-0.90125137858895
H	2.53945306661990	3.56617388521146	-0.54126628960049
C	0.83497904815913	-4.45378349552887	-0.70222122226460
C	-0.08831933278281	-6.39006696591091	-0.14374897037608
H	0.02306713940820	-6.60032738438735	0.93067885196901
H	-0.19923963641214	-7.32876290583272	-0.69986811069239

C	-1.20842192457750	-5.36380829663594	-0.42693322546062
C	-1.89628797642870	-5.62763077457739	-1.76459944242469
H	-2.58224464015275	-4.80334148177920	-2.01033201347586
H	-2.47677815009040	-6.56081703774836	-1.71127871465310
H	-1.15645844199242	-5.72033623481878	-2.57397591049563
C	-2.21947174727432	-5.27043350298369	0.70254645210468
H	-1.72710454430440	-5.05333414920256	1.66043598199736
H	-2.76559997685450	-6.22054851871408	0.79685654689364
H	-2.95211192743115	-4.47608135009810	0.49364704225504
C	-0.88500213186397	0.96068819337299	1.32558258376300
C	-1.72532021650927	1.44562350383656	0.34719080742144
C	-2.09494135901175	2.77937196387618	0.20342796544666
C	-0.83828495527734	3.28273126812724	2.14394942212929
C	-0.49113093791484	1.93651351353624	2.22961947662713
C	-1.98788152481960	5.18248862419585	0.94694660889067
C	-1.83794217925575	5.81306152272326	-0.29627846919464
N	-2.42158262574245	5.83485762910422	2.03155199820091
C	-2.15129122928753	7.16382227110558	-0.40572056683536
C	-2.71553722781368	7.12830647262347	1.91665258046594
C	-2.59989792334324	7.84273571903148	0.72475406069595
H	-1.47359935707338	5.24961953498768	-1.15666187866637
H	-2.03984150904767	7.68065840194830	-1.36214797702730
H	-3.06813883480138	7.63070191547562	2.82498420351509
H	-2.85589726158111	8.90365851785909	0.68865267709074
Si	-0.02035946334740	-1.25768383952537	1.83279946409332
C	-1.36336380714130	-1.12766009579255	3.17905285671777
H	-1.41703009095333	-2.04223558513465	3.79087153840605
H	-1.14275370863345	-0.27048113931275	3.83263338602731
H	-2.34773750795815	-0.94577346826439	2.72159040102680
C	1.69467061194744	-0.43182147389469	1.87803064442999
H	1.75291501034472	0.54085185650921	1.37329847530843
H	1.97940885379430	-0.28727409439944	2.93324474566314
H	2.42568526934054	-1.11865545306555	1.42462855764040
C	0.61291061916862	-3.03726207149971	2.29236974944084
H	1.01524877045817	-2.95147513506321	3.31961817120433
H	-0.18657588906012	-3.79311931925163	2.31789592693782
H	1.43156798590987	-3.41709511162508	1.66164318614493

6c

Electronic Energy (Def2-SVP) = -1282.98118259

Total enthalpy (Def2-SVP) = -1282.72064348

Gibbs Free Energy (Def2-SVP) = -1282.78812098

Electronic Energy (Def2-TZVP) = -1284.19681551644

F	-10.48172429837715	22.01343289011243	2.40900309427576
F	-8.15434716198064	23.22543932935225	2.39131289937143
F	-6.59214260088132	20.20096473748321	-0.89043237629771
F	-8.92461078103848	18.98424197208416	-0.86684372943964
C	-7.26766898429466	21.75549707879258	0.76701493641252
C	-9.80979716126300	20.42537415059400	0.78148552575423
C	-9.54966331754311	21.53039877462075	1.58672174256368
C	-8.31902974747072	22.18277493749133	1.58067544066315
C	-7.51881409335370	20.65097021171846	-0.05347194388359
C	-8.75722433916282	20.01870596624429	-0.03630622354283
C	-5.94385693563768	22.42565161700054	0.79290271576360
C	-5.84490466013537	23.82177489657767	0.74898097916390
N	-4.87197347512282	21.63012914490599	0.86901461715874
C	-4.58061383729542	24.40168678398745	0.79294946690683
C	-3.66759547675480	22.19454156421397	0.90610341136346
C	-3.46378840102454	23.57423778437590	0.87379212692667
H	-6.74261720861054	24.43760298515570	0.68010095281801
H	-4.47132635860095	25.48847296503173	0.76148747959506
H	-2.81099230545814	21.51374647358944	0.96893606679668
H	-2.45254238874823	23.98438087687373	0.91100978705373
Si	-11.48249103057575	19.49032283073407	0.69386129179753
C	-12.64417933785004	20.07324497067633	2.04262379267016
H	-13.56790836209654	19.47434171516222	1.98637290457612
H	-12.20319748875531	19.93035331036549	3.04082970085763
H	-12.91074547319009	21.13391753682990	1.92916516593552
C	-11.13194537802922	17.66073645811378	0.92058302378161
H	-10.46534363102404	17.27461752635791	0.13629716016248
H	-10.66569564793567	17.47099995488673	1.90027688944691
H	-12.07975175249722	17.09978375271757	0.87973964350245
C	-12.20200146048344	19.84876080024490	-1.00153645468870
H	-11.51111196900616	19.53517651757634	-1.79882230315969
H	-13.15256855676851	19.30698378168723	-1.13300795509339
H	-12.40087637903372	20.92586570444150	-1.11693982921232

5a

Electronic Energy (Def2-SVP) = -1094.18367782

Total enthalpy (Def2-SVP) = -1093.83558432

Gibbs Free Energy (Def2-SVP) = -1093.90347006

Electronic Energy (Def2-TZVP) = -1095.13135690983

Bi	9.12916952030666	6.52017618373019	9.22893958551888
O	7.89465328193722	4.25919649575004	5.54583663119967
O	6.32105177386702	10.00063707588341	9.70888824300782
N	8.99764061016515	4.83431494967414	7.40671051826767
N	8.01691328364202	8.58378251378494	10.06694836518949
C	7.45067612593186	7.01875632990003	7.90964158280188
C	7.15111310098409	6.21875686157274	6.78241714952475
C	6.07339912968482	6.53348627115348	5.94756883846443
H	5.85448347109241	5.90064780085815	5.08363802110361
C	5.28158638785772	7.65207855892788	6.22060412314174
H	4.44104353757303	7.89602781241348	5.56772722701597
C	5.56250458608106	8.45927891016903	7.32611666768321
H	4.94549957086329	9.33601051701030	7.53913956586100
C	6.63632198763255	8.14623862862077	8.16583137739573
C	8.03108266760966	5.08674626954001	6.58806799802574
C	8.82735717243856	3.18739437126966	5.75621232651087
H	9.35828055738146	2.99534254323051	4.81445412290664
H	8.25820222135449	2.28924210485916	6.04347690352727
C	9.75355446543339	3.68263536005040	6.89709492727689
C	9.93654316671334	2.63422088899431	7.98500673953513
H	8.96351526163271	2.32678150843137	8.39568962695789
H	10.55091084632005	3.03799884839509	8.80380898005096
H	10.44415777611036	1.74582285951398	7.57912996684374
C	11.10210904357115	4.16453619552944	6.36663735493523
H	11.68808716177213	4.62289233671344	7.17715647914553
H	10.96208044415612	4.91258567553780	5.57155342775948
H	11.67445426939394	3.31866307517245	5.95575270062017
C	7.00849273201623	8.91637265406473	9.33264988125155
C	7.04642073207829	10.58120441394671	10.80378965711924
H	6.33613489983237	10.82044835985212	11.60650783483361
H	7.51963100710795	11.50974205170105	10.44784236517856
C	8.09103209186923	9.50753503798715	11.20618110453520
C	7.68353754056962	8.76658150163657	12.47775919002634
H	6.66659524956888	8.35749631527187	12.37920087829446
H	7.70430631277803	9.44949809875446	13.34089706739244
H	8.37672401867543	7.93478156776661	12.67206692199038
C	9.49042748131558	10.09214872986948	11.33562726430949
H	9.51910749315105	10.83819080240706	12.14455730799974
H	9.79560415177077	10.57923521381739	10.39775587975535
H	10.21629486776006	9.29951430623841	11.57161519704195

PhCHO

Electronic Energy (Def2-SVP) = -344.94282601

Total enthalpy (Def2-SVP) = -344.82485427

Gibbs Free Energy (Def2-SVP) = -344.86251829

Electronic Energy (Def2-TZVP) = -345.314988272144

C	-0.01450845862304	1.23171825423711	-0.00662317846505
C	1.36319964339077	1.05047652876809	-0.01015501942891
C	1.89620328207016	-0.24335564249559	-0.00491525195654
C	1.05343218420575	-1.35610651788289	0.00385523535736
C	-0.32819320889555	-1.17608250649508	0.00741703837791
C	-0.86553684593418	0.11693821694703	0.00220352604928
C	-2.32970035809603	0.29772194937993	0.00606872666137
O	-2.88954128540014	1.37212458949554	0.00222167785590
H	2.03039456282952	1.91579942526530	-0.01703654058499
H	2.98028925106096	-0.38328735114103	-0.00771539888971
H	1.47598004684972	-2.36356105648447	0.00790249781782
H	-1.00065807899713	-2.03930535126176	0.01430881617173
H	-2.91516251803782	-0.65417236035821	0.01305468914508
H	-0.45620821642298	2.23110182202604	-0.01059681811126

TS1^{BiC}

Electronic Energy (Def2-SVP) = -2722.09970088

Total enthalpy (Def2-SVP) = -2721.37050925

Gibbs Free Energy (Def2-SVP) = -2721.49565341

Electronic Energy (Def2-TZVP) = -2724.62271710446

Bi	-3.11671504134748	7.64427987042914	2.72000408913147
F	-4.08427116102921	10.26523717090007	0.71471818579014
F	-5.05330247530208	12.71664267078438	0.85799629443275
F	-8.07818136717905	11.32259584910818	4.22271894811579
F	-7.08878571812103	8.88404904044537	4.10644456981290
O	-0.36216084682908	10.27735305241528	5.32187065575724
O	-0.82131134775255	3.75367404325511	2.32219007888926
N	-2.16509304540207	9.64604665825769	4.15421594104130
N	-2.44885746263274	5.27381650372529	2.15065287408612
C	-6.61404618717936	12.11500421239033	2.53527802166695
C	-1.09697093042050	7.10906132533111	3.69835686248728
C	-0.41250724365038	8.01377232405633	4.51240140789854
C	0.80128468813993	7.66744140657445	5.12161218404574
H	1.31759799485063	8.38958919022740	5.75823429112585
C	1.34230483584682	6.39922816120551	4.90729853554564
H	2.28693633827465	6.12350651226227	5.38074830371614

C	0.68224421273284	5.48419500116218	4.08711630812197
H	1.10309358898057	4.49141562653425	3.91143265467840
C	-0.53275384896500	5.84632163557854	3.49144219178079
C	-1.02636534585685	9.32966788737928	4.66134858318192
C	-1.25201731120527	11.40031512048841	5.41148593660074
H	-0.68341212124182	12.31610474808248	5.20464584377114
H	-1.65143977100299	11.43664169985232	6.43736389146345
C	-2.34914497002480	11.09959294394183	4.36696972929709
C	-3.72934012481115	11.43278297544622	4.90285367637472
H	-4.49985846395572	11.16058043661440	4.17305357528503
H	-3.80413470670956	12.51296064658221	5.10030664929547
H	-3.92717689742278	10.89081987059939	5.83815770672371
C	-2.06635797956403	11.82122537031656	3.05024150364010
H	-1.03537380564633	11.62665822982612	2.71679797978275
H	-2.19148058760535	12.90715105745461	3.17969597871059
H	-2.75217090970977	11.48274558475717	2.26374088218146
C	-1.30219141723789	4.95160978118442	2.63520039141357
C	-1.82100049245410	3.09829468128436	1.52255959833353
H	-2.18724313950163	2.22719288535533	2.08531395777124
H	-1.34815689038946	2.75851561263237	0.59109181314391
C	-2.92239066869973	4.16994448160128	1.29634510740437
C	-2.97171608518732	4.64062164162847	-0.15331493534282
H	-3.69420875053151	5.46273907911775	-0.24884990365478
H	-3.28255906730653	3.81269787018571	-0.80905197591776
H	-1.98095861798318	4.99106208508761	-0.48055917667683
C	-4.28450021631148	3.67779918060271	1.76414440075170
H	-4.24135568600889	3.36342646654362	2.81770747014248
H	-4.60352089697925	2.81675478994154	1.15698279562624
H	-5.02192588716461	4.48458385171347	1.65868204695638
C	-5.52614292797949	9.45397889193059	2.41338225747105
C	-5.07202214845356	10.48573061994995	1.61705803444649
C	-5.57337863032603	11.78033157481383	1.66135369722302
C	-7.10209065693855	11.08154074443205	3.34464672531006
C	-6.54354403644594	9.80855061916707	3.27566107847765
C	-7.16368614026134	13.48957111819156	2.59913668295956
C	-6.30690520807736	14.59527871367784	2.69197185637624
N	-8.49521727341019	13.61770973883501	2.56466715109125
C	-6.86075012640553	15.87021001196850	2.74483705628221
C	-9.01500748571138	14.84200755878802	2.62096479191514
C	-8.24687206801321	16.00259713919801	2.70887501264046
H	-5.22597598645214	14.45043535877688	2.72655216239367
H	-6.21601843735473	16.74949146189719	2.81869332442887

H	-10.10878449829281	14.90936724411389	2.59036735565188
H	-8.72750994016358	16.98225984974943	2.74820691058033
Si	-4.24025474133921	6.56872174018547	4.94807461366724
C	-3.12893975690483	5.22547915024210	5.65958808416925
H	-2.15076411197553	5.62525355397355	5.96504325262959
H	-3.62433805088209	4.79817244828783	6.54817803256693
H	-2.96281581112629	4.41197526071910	4.93729020751542
C	-5.91444424951858	5.82335319960088	4.52534769570160
H	-5.83958245716594	5.07766756869784	3.72188499980912
H	-6.31581386905747	5.32520227194368	5.42431561968315
H	-6.61963407019042	6.60819096027443	4.22049987239847
C	-4.45887365013222	7.94482782888597	6.21144141919016
H	-4.88135452865076	7.52228333719319	7.13886112709620
H	-3.49984883853969	8.42758626732746	6.45050247211835
H	-5.15219886794792	8.70781204762434	5.82888868113249
C	-6.71312998697531	8.31106974091861	0.15148369452224
C	-6.02440676318683	8.47376622107291	-1.05490155601335
C	-7.95977295403039	8.92401250317830	0.32692826304277
H	-5.05664428430791	7.98295631918746	-1.18031607388945
H	-8.49370940571696	8.79247632994592	1.27278611192292
C	-6.56669354112753	9.26135334500685	-2.06684753462652
C	-8.50206298302028	9.71191086929463	-0.68411221001922
H	-6.02451629380621	9.39256447638612	-3.00685485758283
H	-9.47124111852629	10.19607805451302	-0.54036167610975
C	-7.80226027078295	9.88674331396940	-1.88102658738379
C	-6.13393394183545	7.48718871119797	1.24290794655557
O	-5.14369270075174	6.76548489562205	1.07749014259713
H	-6.80342904308708	7.33303142560528	2.11152246686598
H	-8.22389231762650	10.50968828078397	-2.67405422919432

INT1^{BiC}

Electronic Energy (Def2-SVP) = -2722.13549717

Total enthalpy (Def2-SVP) = -2721.40401759

Gibbs Free Energy (Def2-SVP) = -2721.52867793

Electronic Energy (Def2-TZVP) = -2724.65680853532

Bi	-3.46479904192480	8.19075575425919	3.04013480866981
F	-5.44299157012277	9.10798244915794	-0.33004845988851
F	-4.86777033950205	11.67890005239758	-0.39404741820065
F	-6.82106649713267	12.21867534103232	3.87528946559003
F	-7.40728760224939	9.65820175489793	3.93379068978768
O	-1.12807587160596	10.44534958690673	6.31351634483250

O	-0.48317314053339	5.32282423008748	1.27927757796179
N	-2.78937649269516	9.83138057623396	4.95137853175685
N	-2.40991167587693	6.33728507440299	1.76574766809697
C	-5.83170962514361	12.06135459672112	1.73099302260981
C	-1.28637057880479	7.85210133650774	3.75118208441354
C	-0.71907419518398	8.61151135187899	4.78083502333027
C	0.59356002233998	8.37529705165862	5.20998114278322
H	1.02192109961873	8.97404182185861	6.01742496504945
C	1.34861025150570	7.37264727265246	4.59767706526082
H	2.37167398919851	7.18486813663983	4.93043322206271
C	0.80706385934787	6.61469034898554	3.55856527641492
H	1.40098236405235	5.83770070712414	3.07127082150967
C	-0.50970045555404	6.86076888450952	3.14734760542848
C	-1.58340630822527	9.64272108521802	5.35001507770322
C	-2.24875738948049	11.24287683266641	6.73739665298782
H	-1.92263707122537	12.28811138324336	6.81622158693526
H	-2.56707035371976	10.88080092747342	7.72731465183943
C	-3.32551010809717	11.00857433949170	5.64870392383918
C	-4.69341907309659	10.71406200692771	6.24487857660867
H	-5.42149183291372	10.48477600373697	5.45456264909987
H	-5.06178890802076	11.58865210130443	6.80200055068205
H	-4.64462810739323	9.85774227492275	6.93282105918165
C	-3.38926953760835	12.16738721007748	4.65649038862514
H	-2.38605106577284	12.40782513669532	4.27306498784989
H	-3.80710430016289	13.06587095758575	5.13545890313865
H	-4.02609736639139	11.89897528186725	3.80275077343436
C	-1.17015401626927	6.15421495264819	2.05299986812463
C	-1.38519232460243	4.85697019528499	0.25758098204798
H	-1.44433082949441	3.76185875061874	0.32860771330094
H	-0.96477209172100	5.13699133390417	-0.71861443344639
C	-2.73925576378391	5.55691706595274	0.56090425498317
C	-3.16185109437085	6.51485856073534	-0.54603496994270
H	-4.05924813919551	7.05809288056281	-0.21962554214523
H	-3.39087582927995	5.95748079290153	-1.46738282920411
H	-2.36022056078061	7.23733469136913	-0.76345125251889
C	-3.84448247244938	4.55594668268923	0.86788434885734
H	-3.51686269034505	3.83787427053774	1.63378027830534
H	-4.11256413281888	3.99789956666269	-0.04240196592666
H	-4.71918562857593	5.11283018821575	1.23595563088756
C	-6.44542031067950	9.26536393900863	1.80648870178533
C	-5.79591402350464	9.84745394174402	0.71826759421300
C	-5.49114157941735	11.20201694767352	0.68153714796791

C	-6.50256024940707	11.48728539663658	2.81202487800375
C	-6.79435805342493	10.12602653144799	2.84283131056697
C	-5.48587567636325	13.50311882269285	1.70894512173996
C	-4.19362043693304	13.91854290175245	1.36313303913202
N	-6.45155977705163	14.36299287772114	2.04872929794513
C	-3.90580548618046	15.27975734785071	1.37384659814087
C	-6.16495948317963	15.66260148144031	2.05805151682414
C	-4.91026906014859	16.17606546945801	1.72972699457442
H	-3.42990032768130	13.18503502824938	1.10057575855645
H	-2.90561684529618	15.63379197202469	1.11253780285372
H	-6.97890583702991	16.33984916199442	2.34103271936193
H	-4.73054408133394	17.25278147153771	1.75470640076551
Si	-4.17172132109991	6.59771680477395	5.09682504869560
C	-3.93343828207565	4.81083411261293	4.55798721955147
H	-2.88781949462123	4.61473282551575	4.27521450433737
H	-4.20040179944364	4.13972537554265	5.39177446154183
H	-4.57404853042778	4.57444408330963	3.69700831450108
C	-5.98835399404824	6.90480557407274	5.48160358198786
H	-6.60909817785608	6.64954426373517	4.61018013644194
H	-6.29817124386265	6.27263162079350	6.33068585992050
H	-6.17970742583969	7.95604884864356	5.74261824535744
C	-3.13934635628873	6.89459093185092	6.64518718960032
H	-3.45991413289406	6.17655297732953	7.41920713752577
H	-2.06772325729563	6.73591067453268	6.45231462994729
H	-3.27285281746747	7.91058099170829	7.04372792937850
C	-7.59640569592509	7.30501063672541	0.80076325973733
C	-7.21291852617993	6.35910921941981	-0.14927421070774
C	-8.89300884326263	7.83211671831831	0.74430539263007
H	-6.20045566190120	5.95688638913699	-0.09029286255426
H	-9.20649689952180	8.57120298696036	1.48928314343214
C	-8.10352311355872	5.94754476971715	-1.14342072277481
C	-9.78512149790014	7.42583972306912	-0.24610133999352
H	-7.78866469061696	5.20572232078051	-1.88294645557052
H	-10.79374486622811	7.84723557485524	-0.27733881737199
C	-9.39171621477617	6.47952858282844	-1.19665530637872
C	-6.62541933575401	7.73730549744485	1.90445598424505
O	-5.43957983907500	7.08511527148548	1.90253810840779
H	-7.20381693344391	7.59394327814303	2.84812963237259
H	-10.09000732494259	6.15828485425503	-1.97426624940912

TS2

Electronic Energy (Def2-SVP) = -2722.11261014

Total enthalpy (Def2-SVP) = -2721.38235464

Gibbs Free Energy (Def2-SVP) = -2721.5065176

Electronic Energy (Def2-TZVP) = -2724.63704652402

Bi	-12.13335530483664	16.59717170317353	3.81256505578806
F	-9.55523840501521	22.05639618548816	2.85298017659847
F	-8.63233057475045	23.18216150352892	0.64843293146964
F	-7.42737714813480	18.92457384326298	-1.01371791114498
F	-8.32350359423775	17.80967203798766	1.19416929034657
O	-11.48816921536428	18.37481584720624	6.11186017398043
O	-16.31728099643011	15.38592827598694	3.22872988399054
N	-12.43682978925767	20.12905317963786	7.14579679404909
N	-14.09479883984722	15.38480333162672	3.20708022362639
C	-7.98869017845804	21.10286133694856	-0.27461821694624
C	-13.89274148994064	17.54507352301281	4.93950276477629
C	-13.85820945257880	18.60655844490929	5.85591548693425
C	-15.04900686318317	19.18260600957481	6.32458924729364
H	-14.98068921211274	20.00820949457045	7.03628243599298
C	-16.28847012068086	18.70368281674807	5.90994570248953
H	-17.20619978848663	19.16170752098367	6.28421546334829
C	-16.34965925692542	17.62476962743081	5.03337965162456
H	-17.31078181800725	17.21793501800774	4.71118190285410
C	-15.16220545266910	17.05577086666522	4.55883812121183
C	-12.58840351575689	19.10643214915039	6.40246482359009
C	-10.44527372666413	18.88514948898053	6.95615633971627
H	-9.52266924143218	18.96359145639920	6.37099551502977
H	-10.29726652794163	18.17031046429033	7.78150373789475
C	-11.00191349212176	20.23913877203758	7.45122487828127
C	-10.79067838785831	20.42749743867462	8.94657683854348
H	-11.23652942638864	21.37622879699968	9.28024892930345
H	-9.71555785163526	20.44853758649008	9.18360660924181
H	-11.25601601077024	19.60530538896363	9.51049608446282
C	-10.42745116302443	21.41958826743675	6.66951000302704
H	-10.56187954433289	21.27954166739762	5.58622449988924
H	-9.35126557514261	21.53007765232612	6.87305160585015
H	-10.93736679258070	22.34994390649603	6.96039608534350
C	-15.17836913578869	15.92377546643706	3.64712368657622
C	-15.96460694519259	14.40561695619950	2.23157247061159
H	-16.23086635470218	14.81936874708365	1.24715243932291
H	-16.54708600288464	13.49503173635016	2.42019566522958
C	-14.43700644671999	14.20923233152675	2.39330336424337
C	-14.10324228904713	12.95076434471189	3.19073954164114

H	-13.02661048257759	12.91719380330327	3.41439201282632
H	-14.36917938781292	12.05313899640453	2.61237393213666
H	-14.65847035381136	12.93364794017521	4.14062530094692
C	-13.70159211026058	14.23028465391486	1.06254033449432
H	-13.90250878067905	15.16250892135586	0.51564429585976
H	-14.02036487171525	13.38148467474114	0.43925460854940
H	-12.61621304766610	14.14566242169250	1.22442416345942
C	-8.99309650544657	19.88399053237865	2.10708755218166
C	-9.03901102333910	21.26034291892881	1.91324475332980
C	-8.55892295260894	21.85759769428467	0.75179566850072
C	-7.92424544262053	19.71970942635014	-0.07357938975146
C	-8.40916364278786	19.13280646572679	1.08846044271397
C	-7.46277834617994	21.73611020953424	-1.50901070618515
C	-8.22260610957313	22.68516366591813	-2.20434357496200
N	-6.24705446607080	21.35237944283426	-1.91202076238898
C	-7.68783828687416	23.25240140295429	-3.35695471701318
C	-5.74674364502930	21.89793362762719	-3.01792137803996
C	-6.42231100571310	22.85256573627430	-3.77803924258989
H	-9.21486087581396	22.96628189819307	-1.84822322905094
H	-8.25779149311770	23.99395332369728	-3.92200755808153
H	-4.74859049709520	21.56059721733109	-3.32004528948542
H	-5.96318707954268	23.26853033565834	-4.67719391713915
Si	-12.44309131789556	18.55474221827091	1.95186240153104
C	-6.39270129505881	17.55712396429839	5.73315230181992
C	-6.52188357318000	18.94080743983660	5.58725145726347
C	-7.28846272862297	16.70930973116653	5.08233397778499
H	-7.19035237116623	15.62557854859449	5.18829523262983
C	-7.54726951809838	19.46581317033707	4.80425221220694
C	-8.31320385416310	17.23887651842466	4.29533423130335
H	-7.65643004027906	20.55017357976620	4.70370300664263
H	-9.01543647790341	16.58333137832173	3.77811709145923
C	-8.45700190249864	18.61907126614332	4.15803149866650
C	-9.59498429515355	19.21988747688901	3.34235761039229
H	-10.00307219432754	20.04370863809266	3.95864043356571
O	-10.56752867085637	18.31572633885523	2.99805753182200
C	-11.50370161879736	18.42349967836875	0.30916109125352
C	-12.58939206535177	20.29674245819946	2.68923498101827
C	-14.26160195539659	18.28576300672362	1.36428052815349
H	-14.40910930918883	17.29982906402619	0.89465010051413
H	-14.50159997647416	19.05590018579175	0.60851795926026
H	-14.99595263780746	18.38522084570520	2.17996899460106
H	-11.08415670092268	19.41054536573363	0.05394802719159

H	-12.17454563635562	18.11650758805645	-0.50760806690969
H	-10.66541563757619	17.71759561413295	0.37211118738456
H	-11.66028915948161	20.87690987348328	2.61968324488598
H	-12.86825567295035	20.24623494666811	3.75432088276408
H	-13.38536757378376	20.85278336237464	2.16884444697557
H	-5.82376757617928	19.61335395226382	6.09255683756818
H	-5.59338990329291	17.14259529549456	6.35272520185781

4z

Electronic Energy (Def2-SVP) = -1627.97456711

Total enthalpy (Def2-SVP) = -1627.59242574

Gibbs Free Energy (Def2-SVP) = -1627.67414408

Electronic Energy (Def2-TZVP) = -1629.55875564359

F	-9.77423599819825	21.25774040723129	3.65555917037775
F	-8.01596719247561	23.00845253064737	2.74014560511552
F	-6.14742044560312	19.67171435020096	-0.03784874665548
F	-7.90994681388165	17.93267471565838	0.87599916406047
C	-7.00607793476551	21.41172103889657	1.31956472074709
C	-8.89605950364537	19.52387022405961	2.31114700291781
C	-8.88664059266028	20.84261181044527	2.75303001737984
C	-7.96787766287533	21.76791276342689	2.26603027039104
C	-7.00964210782832	20.08273893679229	0.88205257654590
C	-7.93654872271256	19.16696695824492	1.36429853465733
C	-6.02144829683678	22.39496268626413	0.80311287597812
C	-6.43950228956868	23.65583740690262	0.36078336023075
N	-4.74214423175384	22.00904519386486	0.78013139972530
C	-5.48180380888601	24.54303276618939	-0.12133313983071
C	-3.83473091411238	22.86437925630781	0.31538531796156
C	-4.14869402656742	24.14247063117566	-0.14724404097192
H	-7.49536416615271	23.92930124274520	0.38977587814355
H	-5.77678836515346	25.53330518378688	-0.47678920759678
H	-2.79491524667463	22.51817653976492	0.31037670258922
H	-3.36266280114294	24.80345613355094	-0.51803957739218
C	-8.13249666934385	15.19105897714437	4.79887515213782
C	-8.89144743951452	14.97665876527541	3.64902356267280
C	-7.97070762231400	16.48969958432765	5.28728207870812
H	-7.38009560910866	16.66665951802985	6.18975844099957
C	-9.48474420437540	16.05247522885280	2.98640591504349
C	-8.56321852391955	17.56321815561749	4.62618478689853
H	-10.07883270855856	15.89690132806133	2.08472071521896
H	-8.43487383004928	18.57949505905449	5.01106569658674

C	-9.32164367013239	17.35055844358855	3.46977908306527
C	-9.95659289613055	18.53528471886436	2.76587826165944
H	-10.58096780602921	19.06102467601656	3.51015168218360
O	-10.73697894455122	18.10810882535498	1.69146335284243
H	-9.02338430206434	13.96305924422609	3.26121595175403
Si	-11.71234433297930	19.10620980108690	0.71693496641465
C	-12.67302677145968	20.27236739582859	1.82187016069692
H	-13.34212555981311	20.90123234769154	1.21239932186948
H	-13.29061074603023	19.71382960477234	2.54310086599222
H	-12.00311742882456	20.94121758517826	2.38412293255905
C	-12.82383858396136	17.90082804409911	-0.17304142244244
H	-12.23147182942313	17.20019847410942	-0.78261755662573
H	-13.42472803837157	17.31743372281336	0.54183053840432
H	-13.51186406568500	18.43902250909686	-0.84444559026561
C	-10.65428870285042	20.05976654426930	-0.50139872313788
H	-9.94445520519027	19.38865753181000	-1.01074284326023
H	-11.29980749500591	20.52015531653341	-1.26732437870615
H	-10.08288141861530	20.86384384917574	-0.01295817147420
H	-7.66765447420352	14.34797397296482	5.31624733583055

TS1^{BiSi}

Electronic Energy (Def2-SVP) = -2722.0522823

Total enthalpy (Def2-SVP) = -2721.32495905

Gibbs Free Energy (Def2-SVP) = -2721.45020672

Electronic Energy (Def2-TZVP) = -2724.58029508963

Bi	0.09596101294021	-0.76806316474869	0.42356319502663
F	-2.82377899588712	0.59354392642265	-0.96277893628322
F	-3.73342690434051	3.05884673392275	-1.19208195070350
F	-0.36538007697843	4.58301020197675	1.74930302156395
F	0.56293662887110	2.12748045563034	1.95735576481214
O	-0.20884168506600	2.46765245169711	-2.87035153031598
O	-1.11157351884787	-4.61183087424881	-2.27452671819943
N	1.41214793637640	1.45742889356695	-1.68758086676291
N	-0.38325442717819	-3.59557341072904	-0.41177367545887
C	-2.09035248435050	3.91960448633278	0.26863024951130
C	-0.49224717317187	-1.01529629082367	-1.83202175610500
C	-0.34942461602894	0.08037357492777	-2.70459478698611
C	-0.71580107154787	-0.02183526765014	-4.05642788470564
H	-0.62150599774906	0.85545573008888	-4.70038155180059
C	-1.14851650955090	-1.23310178836253	-4.58410669151810
H	-1.41706754399195	-1.30985776655011	-5.64009541538326

C	-1.18201244078320	-2.35390597137957	-3.76590302967659
H	-1.44988023032064	-3.32938914786581	-4.17451454767467
C	-0.85405322043818	-2.24640970760963	-2.40424340905452
C	0.31808071546451	1.34719465275791	-2.33361062723545
C	0.59365066908665	3.55116511969922	-2.38370192875089
H	0.02792060532625	4.06836099455010	-1.59206815762631
H	0.78500101049248	4.24876867685725	-3.20968637489617
C	1.86171576823480	2.85556185696104	-1.84709832546411
C	2.97563404605636	2.85784242427002	-2.89541105416550
H	3.81615016053973	2.23280792614656	-2.55921372136267
H	3.34449134238146	3.88242337615784	-3.05837613085811
H	2.61023896785840	2.46259043982913	-3.85578663627196
C	2.36575375258494	3.45524111769911	-0.54495271885335
H	1.59689197450920	3.44009984591830	0.23554906472247
H	2.67270238500018	4.50023540993344	-0.70409675916652
H	3.23818768912652	2.89303828865567	-0.17986234424621
C	-0.78658252977949	-3.48512940708564	-1.61839292178724
C	-0.77088973364589	-5.70261818328046	-1.41709070339635
H	0.08111991574974	-6.24067984616487	-1.86176380192359
H	-1.63222443239305	-6.38189443281345	-1.35436751090913
C	-0.41860443355971	-5.03113896455606	-0.06487232224638
C	-1.51418688752482	-5.28959745181202	0.96437713691605
H	-1.32259999043114	-4.73511970302486	1.89085271647743
H	-1.55802656263077	-6.36335935250024	1.20369261878862
H	-2.49375059997460	-4.98030997617263	0.56971046876390
C	0.94573857199211	-5.48301712372145	0.43750676892363
H	1.72658326539178	-5.24823569679248	-0.30139548000450
H	0.94708831882534	-6.57017414248953	0.61152590996814
H	1.20507562990712	-4.98057169047417	1.37966140323019
C	-1.05478555666083	1.26044221940925	0.47190634118122
C	-2.17908358574980	1.55146109483844	-0.28958130284272
C	-2.67493648929355	2.84842206862519	-0.41079496813329
C	-1.00795502013682	3.61662133445420	1.09965177123548
C	-0.51876982547451	2.31848731978375	1.18884826546160
C	-2.55655523907750	5.31661394515522	0.08608111676788
C	-2.71384103134262	5.84367268693726	-1.20251927606752
N	-2.78118448264372	6.03515087996945	1.19047168802205
C	-3.11615154825949	7.16926651302458	-1.33629011207856
C	-3.16689753320634	7.30170437526809	1.05105146664200
C	-3.34792656542414	7.91954489458160	-0.18621508129569
H	-2.51055484471273	5.22540494517973	-2.07873700350780
H	-3.23948207858807	7.61193394679068	-2.32776461275835

H	-3.34373692433464	7.86285138094227	1.97578660121112
H	-3.66073851178599	8.96439292282240	-0.24094933623618
C	2.24422887733941	-0.43545978551342	2.01627182579715
O	1.49095361434074	-0.59124743951643	2.99094687835133
Si	-0.89923025819813	-1.41782710072940	3.10069077068138
C	3.09647117935526	-1.51610864668070	1.48233467495154
C	3.13983745186062	-2.75582650180405	2.13119340085990
C	3.88522606466127	-1.29451698489831	0.34461531188015
C	3.97863135004260	-3.76006733983795	1.65549427436569
C	4.71512673695187	-2.30094052046094	-0.13237059087853
C	4.76841967120170	-3.53434872971629	0.52677977818430
H	2.51069479182796	-2.91257244498932	3.00927991235658
H	3.81141740965409	-0.33519488194134	-0.17675275531832
H	4.01749412732166	-4.72387108180360	2.16890820805886
H	5.32527439695246	-2.12711721128528	-1.02176000797270
H	5.43035728438162	-4.32140810675298	0.15796249534062
C	-0.25511261311529	-3.08667971680549	3.69699094637368
H	-1.08360499146660	-3.65329282186510	4.15374411879928
H	0.16629208523322	-3.69195932758132	2.88440204365026
H	0.53023802644216	-2.94295740028898	4.45396961144936
C	-2.72263584975492	-1.49522810514055	2.63719837891505
H	2.43063926455599	0.58036228349471	1.60973559729368
C	-0.80075498988582	-0.18231746982391	4.52941205151076
H	-2.90267288164286	-2.22644607558426	1.83620956142345
H	-3.03056600217579	-0.49968406005484	2.27827516189011
H	-3.33344096970375	-1.75235354852996	3.51916532776555
H	-1.64528559920629	-0.39158056984642	5.21003267701722
H	-0.87297685238743	0.86814251949402	4.21056388385143
H	0.13848961156044	-0.30708668246571	5.08716285688941

INT1^{BiSi}

Electronic Energy (Def2-SVP) = -2722.13457902

Total enthalpy (Def2-SVP) = -2721.40462247

Gibbs Free Energy (Def2-SVP) = -2721.53041522

Electronic Energy (Def2-TZVP) = -2724.66524064241

Bi	1.72564625801483	10.85609271193604	4.32380208703736
F	-0.28171756572442	12.52461240042252	2.28015613426419
F	-1.12105276431198	15.00854341064813	1.94684444915514
F	1.41492698479846	16.36003475311411	5.70241060007380
F	2.28349499699569	13.89819727679917	6.00500954039287
O	2.67878872712808	14.40399072584629	1.28032941114496

O	1.80968298618931	7.27994462354628	1.53084764907481
N	4.04779149215613	13.45327768630000	2.78543573509116
N	2.25114868743589	8.29436480037083	3.47679201336339
C	0.09320231302718	15.78088580461336	3.82354705026267
C	2.39251745588912	10.86683099356381	2.12710279373309
C	2.67929927453086	12.00590460217528	1.35556115440623
C	2.69932567816288	11.92813568859124	-0.04722801666958
H	2.90166572436684	12.83412712995143	-0.62239680982176
C	2.50375737608167	10.71679713197938	-0.70078084820397
H	2.52840762788825	10.67068702004157	-1.79156910238882
C	2.31568497552127	9.56260344387600	0.04863358299527
H	2.20751403108265	8.59181467455874	-0.43812433707160
C	2.26217788253209	9.63880224807493	1.44678572348508
C	3.13673875074910	13.29948741522322	1.90824470602070
C	3.29921850349434	15.51671965644229	1.93893664786423
H	2.54800949968667	15.99932995985148	2.58389472509859
H	3.63701580912736	16.23278729146204	1.17800654563021
C	4.44701731149123	14.87246285714034	2.74374925047343
C	5.76666652611803	14.94615322857688	1.97364402978747
H	6.53734769027317	14.34814784677340	2.48228873238503
H	6.11551759819383	15.98849812697670	1.91006636539991
H	5.64441701431777	14.55685500193578	0.95096469158263
C	4.61439682459152	15.46519144943416	4.13324095761684
H	3.69050160513257	15.39913916199517	4.71908679348976
H	4.90159252451202	16.52531311424307	4.06098261049663
H	5.40698882765804	14.93189342145734	4.67905236777893
C	2.11054114843927	8.39496120512800	2.21027407766998
C	1.86555377287810	6.20311031102654	2.47732950465588
H	2.76420035022841	5.60396916039352	2.26237497257602
H	0.97122327865865	5.57897120491833	2.34938444461003
C	1.94083358202523	6.90651546361313	3.85587103247279
C	0.59453965631810	6.87898102632934	4.57631079993216
H	0.63715241802277	7.48068854903164	5.49591926666008
H	0.33089613610772	5.84587085891327	4.85012590361491
H	-0.20037493711119	7.28470936525039	3.93223417812935
C	3.04550732510059	6.32826478660235	4.72744500947710
H	4.01811210116222	6.40146171796773	4.21945461700262
H	2.84123045250204	5.26929767314389	4.94788567980179
H	3.11198128791709	6.87498157611522	5.67851681947696
C	1.05360263930479	13.10649747440973	4.14517406280967
C	0.16175568121475	13.45164001068682	3.14240587709805
C	-0.29584257230287	14.75296247797024	2.96152282888946

C	0.95180651212000	15.42967425577785	4.87030789818044
C	1.41380425613370	14.12519805530041	5.01095931046331
C	-0.35037709871506	17.18026706320137	3.61559779943244
C	-0.24990043939504	17.76979281239559	2.34876587539180
N	-0.82544767968941	17.83563553133564	4.67940981069728
C	-0.66272598361616	19.08913375853398	2.19230687292622
C	-1.21547350805369	19.09832095148500	4.51921290288924
C	-1.15708856632229	19.77322865707465	3.29996583661457
H	0.15130960077008	17.20128733647082	1.50822734680256
H	-0.59424887095509	19.57713507296366	1.21701967280980
H	-1.60068367259656	19.60682087666835	5.41047072085988
H	-1.49181479647699	20.80997835715367	3.22620574142433
C	3.84017562309917	11.29276809029234	5.32732047568107
O	3.76181795957725	11.38232418350441	6.70505547436332
Si	2.74556808449219	10.82423350396583	7.93054784147740
C	4.83719266159010	10.29135229098997	4.84352818626954
C	5.31709361263052	9.27291029642925	5.67695968978159
C	5.33514007390448	10.36751418126840	3.53032949790446
C	6.23682194768496	8.33573019952664	5.20507535862478
C	6.24830025986139	9.42926268408860	3.05951263373367
C	6.69930027905780	8.40003046762863	3.89155065270669
H	4.97296772327605	9.21824466655106	6.70980797474976
H	5.00356275208088	11.18697309997259	2.88890777631610
H	6.59409464624756	7.54806750864994	5.87398517684996
H	6.62028142588905	9.50604927692907	2.03407731690459
H	7.41587525559239	7.66291412422504	3.52048768506827
C	2.36938608373566	8.99722623299377	7.73115740957001
H	1.60744877604170	8.70396147445935	8.47182052386722
H	1.96671514977502	8.76408480508587	6.73353245632520
H	3.26066429238505	8.37231030175120	7.89218772526470
C	1.13825981869914	11.78937254672739	7.96511970592407
H	4.04251578115278	12.27988573036382	4.88726524584814
C	3.72902283932943	11.14836536035835	9.48350582704441
H	0.51035650900478	11.61574662251976	7.07678081998703
H	1.32905202315829	12.86918767747911	8.04312665261009
H	0.55559377043525	11.47042918225434	8.84510660657566
H	3.15571992343674	10.84781476466575	10.37478111577630
H	3.96995059733739	12.21918117724444	9.57408779181031
H	4.67276188674143	10.58104148927481	9.47267644964225

5c^{eqF}

Electronic Energy (Def2-SVP) = -2068.01108403

Total enthalpy (Def2-SVP) = -2067.51698402

Gibbs Free Energy (Def2-SVP) = -2067.6149442

Electronic Energy (Def2-TZVP) = -2070.07228771462

Bi	2.79615915009610	11.13997513560654	3.82694629763805
F	0.08219312727625	11.71509465315251	1.64366587086830
F	-1.60700490700181	13.71542805760986	1.39537511851335
F	0.53681797959784	15.98560674241774	4.94721364991196
F	2.17024009093339	13.92578045690019	5.26249918910396
O	5.58565483993047	13.59176626475618	0.98347766855191
O	0.87608751845868	8.13042612782800	1.12118101713932
N	4.36492329107573	13.10521646818362	2.79493172603523
N	1.49099219433238	9.28880718536179	2.91638998868713
C	-0.61245159338923	14.92756425550283	3.16553859201202
C	3.10556339784598	10.96723865188552	1.50369218687113
C	3.98843883667866	11.78820910470744	0.79971208051931
C	4.23186426451901	11.57880534006236	-0.56585085207827
H	4.92366924629341	12.23156456329327	-1.10344309159313
C	3.58607729248405	10.53575598745877	-1.23069675069589
H	3.77050611943603	10.37411463906955	-2.29489890670596
C	2.71311755305067	9.69291217691116	-0.54126046792818
H	2.21497625843904	8.86765881135983	-1.05564572917645
C	2.48985056226063	9.91655328555462	0.82258831793442
C	4.64074805943759	12.85068737641067	1.56882400507791
C	6.17366713331449	14.38471523717225	2.02864388680231
H	6.30022604672448	15.41098481383543	1.66050915868593
H	7.15956755297870	13.95493434985580	2.26700481681295
C	5.17542140681355	14.26451436773501	3.20532314217177
C	5.86511884601604	13.98788963002074	4.53214849135905
H	5.11528815862129	13.85674543034229	5.32648191982956
H	6.51952060247145	14.82817924285940	4.80985648052773
H	6.47474316942611	13.07414599788801	4.47071392206925
C	4.28159673477828	15.50097154275243	3.29212352050431
H	3.78929258954181	15.69155629046118	2.32632119530110
H	4.88594914362990	16.38286320450775	3.55408695250294
H	3.50899473451895	15.36852295976163	4.05951939114580
C	1.60923725452968	9.09611542930871	1.65119505711034
C	0.20743386569754	7.47163726688768	2.21849733573582
H	0.67107675649783	6.48169343482635	2.34465227226314
H	-0.85067411074073	7.35181765541023	1.95247743544135
C	0.44001547634356	8.39959999214656	3.43749403088564

C	-0.78720032050215	9.24401607881286	3.77049281484528
H	-0.48972408018686	9.97166207770155	4.53832431445374
H	-1.59795598478434	8.60422908134655	4.15141832373063
H	-1.14572270942435	9.77992625495677	2.87922229970574
C	0.93423736395930	7.64818558581609	4.66352242543844
H	1.83967822399742	7.06852931252812	4.42858132363897
H	0.15874973166577	6.95717827372679	5.02729944905982
H	1.16424177370606	8.38968774109384	5.44093305515600
C	1.21652195124831	12.75831791914387	3.44816587415773
C	0.22481814795310	12.74443052631351	2.48212311052849
C	-0.66996600219398	13.80340391792923	2.33813594914779
C	0.37996730211161	14.93368915717427	4.15131881170161
C	1.24639047560013	13.85608851463362	4.29194177477096
C	-1.56228558811210	16.05765813161246	3.01795191983486
C	-1.81660731605239	16.61927371312960	1.76074979774112
N	-2.14418973015206	16.50290479200465	4.13639837149563
C	-2.71204927690022	17.68093759495179	1.67396038379713
C	-2.99740871570575	17.51970031217554	4.04268365975822
C	-3.31837344532002	18.14560289421783	2.83811222467993
H	-1.31554327678809	16.23237699292366	0.87217425226721
H	-2.92808128010272	18.14244759111240	0.70733773102846
H	-3.45705350240228	17.85903026806192	4.97804076924624
H	-4.02601298132375	18.97699104662851	2.81910504225593
F	1.36806059679050	10.61575009219239	5.47140740172312

TS1^{eqF,MI}

Electronic Energy (Def2-SVP) = -2412.92373668

Total enthalpy (Def2-SVP) = -2412.31116505

Gibbs Free Energy (Def2-SVP) = -2412.42216187

Electronic Energy (Def2-TZVP) = -2415.34395864709

Bi	3.05818759570352	11.27981295079875	4.26376958699967
F	-0.53536712170100	11.37845140896084	5.14467126100763
F	-2.08675234139624	12.35197921489323	3.25095745876514
F	0.89385511952495	15.99388831094323	2.72586747994089
F	2.49793679082854	14.92936067567534	4.50137699559543
O	5.41989587267364	14.90109498595661	2.48526100673478
O	0.29877725633211	10.21286764193828	0.75783526151723
N	4.85059223542816	13.21488171301053	3.83761001580202
N	1.47200001938676	10.08232195277878	2.65043131438357
C	-0.64919738320844	14.19947303568554	2.89094138307163
C	2.67995178303995	12.51263263276214	2.24744425839200

C	3.43777830137157	13.64141887290003	1.92391515407388
C	3.14855616450613	14.40366573830765	0.78525578473943
H	3.74598206138076	15.28847397534006	0.55367703013102
C	2.10504617835111	14.01552349489621	-0.05721109589049
H	1.86631061053423	14.61440152405996	-0.93858081605403
C	1.40123185407625	12.83914735011696	0.19500976036116
H	0.62986209213640	12.49451474521168	-0.49743928259380
C	1.70693616870125	12.09189373067964	1.34126834659421
C	4.58134773985864	13.91337067910573	2.79665206892219
C	6.53521399727406	14.78470118172136	3.38592892438501
H	6.76224558445286	15.78031380980588	3.78876706154676
H	7.39812775042488	14.41721369652869	2.80917897761114
C	6.05973892468311	13.77515801535425	4.46186945147998
C	7.07861239548820	12.67182097153010	4.70944826827057
H	6.68892931110146	11.95268658681323	5.44571011542142
H	8.01538731222244	13.09434222744671	5.10382038683154
H	7.30211438542763	12.13205387539792	3.77723155789262
C	5.68380642885994	14.48037863328598	5.76116692750522
H	4.99952356976836	15.31789978431731	5.55882948831441
H	6.58614921730633	14.87497581826174	6.25287371238424
H	5.16911970629737	13.77988045783422	6.43206529776534
C	1.13986604099663	10.77394077677447	1.62020266685787
C	0.16463828568209	8.83652805486517	1.16712695093587
H	0.74997354511834	8.22213815092799	0.46538790265047
H	-0.89495622900941	8.55829503370806	1.10627437735935
C	0.73165937583392	8.80700384369132	2.60688051526767
C	-0.38511643732248	8.82124131511723	3.64341418145111
H	0.05306351579588	9.01214407016710	4.63049884094246
H	-0.91705224832386	7.85751929500729	3.63349928040103
H	-1.10334008540195	9.62429090776369	3.42384312722322
C	1.68323090138457	7.64525776295879	2.84425051824031
H	2.51592909204355	7.66457950430861	2.12548117210871
H	1.15282057934761	6.68619330428867	2.74387317829118
H	2.09114367656793	7.71328238636107	3.86318121143978
C	1.13413295656581	13.01794822431368	4.80089731912421
C	-0.10854190530273	12.46276866931615	4.50256935130565
C	-0.95576909078208	12.99858268260427	3.54137923232060
C	0.52765358321382	14.84648849561375	3.28800060912570
C	1.37849161853463	14.25968633109656	4.21586020394749
C	-1.51442204134539	14.74150947547033	1.81830962995103
C	-1.98850533209608	13.89632652597346	0.80568016881032
N	-1.78939643433518	16.04926256006205	1.85291588936125

C	-2.77295540021494	14.44284772010442	-0.20482687395327
C	-2.54245956284018	16.56091722160271	0.88254954209096
C	-3.05848376413897	15.80536103243964	-0.17068661870971
H	-1.73463079527691	12.83548340633736	0.80903756514910
H	-3.15198574074057	13.81069120036045	-1.01147904557327
H	-2.75195020713863	17.63508772155216	0.94171809217371
H	-3.66856366631593	16.27903251503803	-0.94255626231977
F	1.78634957948455	10.02007889373086	5.52873311482186
C	2.27314591082618	14.92566597780359	7.69260490476541
C	1.77975798122654	16.02965691084251	8.38481211970350
C	0.44328110376911	16.06252726873948	8.78806582570118
C	-0.39951044591870	14.98402395464519	8.50403322445695
C	0.09437540442912	13.88112683422743	7.81383771468759
C	1.43161061727253	13.84819784610568	7.39791671501223
C	1.94405093894170	12.66090660695616	6.65895018676656
O	3.19034470158981	12.48271914195063	6.50554832203810
H	2.43955637392911	16.87102695532083	8.61182585943643
H	0.05624741455734	16.93049078547645	9.32812274895117
H	-1.44437568675658	15.00667992750023	8.82369692040982
H	-0.55990555661663	13.03553707401060	7.58048748043801
H	1.28571121886642	11.77426545052904	6.71333075415143
H	3.31456663306313	14.88312849200314	7.36982824078061

INT2^{eqF,MI}

Electronic Energy (Def2-SVP) = -2413.00094668

Total enthalpy (Def2-SVP) = -2412.38532424

Gibbs Free Energy (Def2-SVP) = -2412.49622427

Electronic Energy (Def2-TZVP) = -2415.42171822073

Bi	3.29266010978614	11.25558985152959	4.34884845167495
F	-0.91986346537848	11.70804120706068	5.32234851920161
F	-2.26995549965021	12.22821772003345	3.11875410336876
F	0.47485772200528	16.00155586861229	2.34402361960129
F	1.89997670065360	15.39419716361680	4.48047207840177
O	5.22497409715630	15.17528813732797	3.03578086481870
O	0.19041471846462	10.47343957147349	0.89389289485519
N	5.05026070967415	13.15349146209062	3.96744250383815
N	1.56728643078005	10.00890458714756	2.59617469566020
C	-0.95012951292941	14.13213353803657	2.63422917836894
C	2.70927498816213	12.56916888112935	2.51728792871918
C	3.42012132332740	13.75480617050968	2.29231459840414
C	3.10364850278081	14.59494206894165	1.21762443133066

H	3.65986895005440	15.52400052159289	1.07664517609020
C	2.10103906514082	14.22512452676781	0.32352309889805
H	1.84972485394135	14.87732799272686	-0.51521755807698
C	1.44534840973037	13.00730011179992	0.47986995417325
H	0.70165952065493	12.68076146518757	-0.24909297705181
C	1.75549396273388	12.18569655315302	1.57265665592834
C	4.57859417427559	14.01694742670689	3.14866429737532
C	6.40039045187404	15.07183479342015	3.85841658943820
H	6.43990097651317	15.95083550887927	4.51557918735516
H	7.27901542028195	15.07092444195481	3.19620998509149
C	6.22734951256507	13.73422622554060	4.62863319344812
C	7.42618649752174	12.81162409754214	4.45210088706834
H	7.24745009615354	11.85304930254263	4.96183557801070
H	8.32869304705598	13.27008430390267	4.88443008608018
H	7.61034718371085	12.61282272723284	3.38589583714827
C	5.92897310884972	13.97167840438266	6.10436292053009
H	5.08194082180382	14.66144252477286	6.21930172560461
H	6.80993160308279	14.40392053184748	6.60308793956536
H	5.67132879308546	13.02528437020664	6.60163055722285
C	1.17062646717320	10.85285851989831	1.72343869530753
C	0.01164190269714	9.06435842055559	1.13006833677495
H	0.51404238976016	8.51832890437638	0.31530826512926
H	-1.06261079205536	8.84176832735099	1.11841357467678
C	0.68325223420604	8.83104454100092	2.50162397145497
C	-0.34571068349932	8.88320681935837	3.62881195567893
H	0.16988064023088	8.96726167301856	4.59325391461337
H	-0.98025280815054	7.98397753255441	3.60324234427913
H	-0.98799386523214	9.76980534203553	3.52037500191136
C	1.49621173950636	7.54845899756600	2.55015240622279
H	2.27080265902131	7.54654110163762	1.76896217479309
H	0.84543211345361	6.67327735244807	2.40255225821960
H	1.98584513013543	7.45153483087108	3.53068810347735
C	0.57778662632315	13.49803989973156	4.95949012805992
C	-0.51695032677693	12.73084632633551	4.57621322478647
C	-1.24820975113097	13.02538295235826	3.42986892860090
C	0.12026577554824	14.93293742852741	3.04787763778470
C	0.87278307906750	14.60923365643625	4.17049375867600
C	-1.73541069843165	14.44311798249860	1.41537782393630
C	-2.04396744685635	13.43248158252257	0.49523705637623
N	-2.11729059430641	15.71305908550287	1.24584474501299
C	-2.77880245467083	13.76579517482376	-0.63815008249831
C	-2.81746022354747	16.02108841445380	0.15676251834370

C	-3.17688324762308	15.08842780413096	-0.81604246337799
H	-1.70118901129639	12.41041705005498	0.66386001522043
H	-3.03195057048874	13.00055409140429	-1.37586550738325
H	-3.11477617996192	17.07049654120817	0.04856117659592
H	-3.75399693023560	15.39714285485770	-1.69004745211888
F	1.62718964424876	10.37196607727144	5.37489805789249
C	2.57739618554450	14.45607819679926	7.93480454116524
C	2.52149970467683	15.36395637615734	8.99375480317543
C	1.30248429271284	15.92223473029278	9.37768810981074
C	0.13788467579519	15.56190405287907	8.69629149259809
C	0.19649167440264	14.65654472982449	7.63806132555118
C	1.41866492483758	14.09849921300349	7.24289617893441
C	1.47866229252043	13.08250303318955	6.11298268052488
O	2.78073255024177	12.89923214936238	5.67416875577453
H	3.43934004414370	15.63763630352646	9.52138851606507
H	1.25808281227086	16.63652746339830	10.20389811204699
H	-0.82371249390341	15.99215351521353	8.98864527953324
H	-0.72127964369174	14.38419181632170	7.10879093186870
H	1.05646610297728	12.13684676501154	6.49649154817030
H	3.52387398382048	14.01433839394376	7.62279287899073

TS1^{eqF,ISO}

Electronic Energy (Def2-SVP) = -2067.99717675

Total enthalpy (Def2-SVP) = -2067.5044181

Gibbs Free Energy (Def2-SVP) = -2067.60135845

Electronic Energy (Def2-TZVP) = -2070.06105377813

Bi	1.32855548085396	-1.49046653022942	1.23041841382299
F	-1.66212966864966	-1.06820133184242	-0.97901506609816
F	-3.27033796745214	0.97178671272418	-1.30573382561114
F	-1.07915877849143	3.29483768651869	2.18217980842622
F	0.48692401815839	1.20457861538403	2.57720170433899
O	4.10030210404073	0.95352442452277	-1.57653076196510
O	-0.88884269786565	-4.26304472361368	-1.50945257921643
N	2.78130028405962	0.53364246621963	0.18111232718711
N	-0.17923592767352	-3.18991547994198	0.30355289742082
C	-2.25301415356144	2.21160049027960	0.43096631754699
C	1.64065680070573	-1.69369236169783	-1.06106513537086
C	2.52538450263441	-0.87328848422273	-1.76327306520613
C	2.74259715589440	-1.06798879422681	-3.13450371918588
H	3.44436056265054	-0.42546035967190	-3.67129988402126
C	2.04274873728857	-2.06876277230540	-3.80969787472108
H	2.20528140377508	-2.21587929330422	-4.87951993857004
C	1.11908522071768	-2.86521698027518	-3.13211494769286
H	0.54356050232089	-3.62585337626252	-3.66462504911794
C	0.93224213140913	-2.67244137036366	-1.75826623287903
C	3.14157763875519	0.22115362869946	-1.00808749007198
C	4.58236846159313	1.84581958258530	-0.55518202453648
H	4.66072909901850	2.85408549653464	-0.98231743446292
H	5.58073719658809	1.49852226615508	-0.24687067647454
C	3.53511278280332	1.73208141565091	0.58156009739803
C	4.17453196672955	1.52757186970143	1.94656470254545
H	3.39516581942429	1.39557107592704	2.71170169776533
H	4.78417204566864	2.40197426913842	2.22098882402062
H	4.81988387214085	0.63664441969696	1.94470263540672
C	2.58953555407388	2.93232816033528	0.58851279612740
H	2.13529512050261	3.07443851246564	-0.40393384791042
H	3.14209580986793	3.84704534377191	0.85243769933430
H	1.78672800743973	2.78556969452210	1.32281909153739
C	-0.05110197111983	-3.39604414283489	-0.95560259300900
C	-1.87817340221589	-4.54936548138761	-0.49623564422320
H	-2.12098300615946	-5.61806991109724	-0.53851156384067
H	-2.77264650779778	-3.95051727312843	-0.72953543213355
C	-1.20200024793319	-4.11517466075205	0.82173488125719

C	-2.14774857630696	-3.39189139865738	1.76458345902556
H	-1.59583632679301	-3.06443688485669	2.65814441741486
H	-2.95857341017279	-4.06249754524589	2.08601358965777
H	-2.58818556703656	-2.50992967211987	1.27736387593663
C	-0.50444092152101	-5.28688290055150	1.50896872443832
H	0.07484280780012	-5.86970365470256	0.77523224741595
H	-1.24692547476486	-5.95262204057931	1.97489489318272
H	0.19155203707402	-4.88208909790924	2.25677399584486
C	-0.50518014403050	-0.02116053164492	0.81848104385715
C	-1.48697642847973	-0.02583374708824	-0.15913582151778
C	-2.34287244099686	1.05736295896491	-0.35158531420325
C	-1.27175971850225	2.21767954187500	1.42686484179413
C	-0.44403626925124	1.11514062935120	1.60931444046921
C	-3.15129348168355	3.37320668569845	0.21783086848862
C	-3.35464968147320	3.89121372518794	-1.06746086416937
N	-3.73594642958182	3.89167624570082	1.30278240730583
C	-4.19865089180745	4.98697476803360	-1.21940291820247
C	-4.53985998706797	4.94085158758201	1.14666157575898
C	-4.80725643538876	5.52810151812018	-0.08997322250811
H	-2.85344275894742	3.44491451413673	-1.92769015945867
H	-4.37344401639301	5.41525696245267	-2.20942106573055
H	-5.00310146935515	5.34033480029958	2.05613577355918
H	-5.47598500314432	6.38849056264058	-0.16004192305789
F	1.45646263762946	-3.29754983036315	2.56958602688145

5c^{axF}

Electronic Energy (Def2-SVP) = -2068.00726079

Total enthalpy (Def2-SVP) = -2067.51356638

Gibbs Free Energy (Def2-SVP) = -2067.61397102

Electronic Energy (Def2-TZVP) = -2070.07507388729

Bi	2.99044306450360	11.26130761683564	3.77355307855648
F	0.17731950462916	11.90868297499851	1.28952059237843
F	-1.67932600379279	13.75703697168920	1.14246774941722
F	0.13261166210328	15.90881475289427	4.95060433830992
F	1.97012587505545	14.01902875129166	5.13160647468106
O	5.83952516866639	13.46445329021389	1.06578255425239
O	0.72967789624928	8.36622564211938	1.23516097672797
N	4.62479823950243	12.94823378124153	2.86127116537227
N	1.41476007953576	9.54066708910381	2.99502485531900
C	-0.85094217706069	14.91107199441999	3.03552031568601
C	3.22439566300100	10.98690252660449	1.54568176229522

C	4.16595718087176	11.72691940439532	0.83636071753352
C	4.37936275339006	11.48651160839411	-0.52757805736242
H	5.11729599696254	12.07287080955122	-1.07984252858817
C	3.64110672247840	10.49125389302098	-1.16978119620035
H	3.80165287818326	10.29988990705844	-2.23275466008385
C	2.70067255908552	9.73608987393326	-0.46539412991304
H	2.12633906594358	8.95568397686091	-0.96991014595527
C	2.50203589232170	9.98846839655645	0.89680791492104
C	4.88259775635256	12.73346778373164	1.62042640725219
C	6.44519627808544	14.22111889826858	2.13403463930465
H	6.55867612608593	15.26026519750180	1.80004185517210
H	7.43636409903723	13.78568588195623	2.33197109768185
C	5.47171267706436	14.05635914825004	3.32869061319716
C	6.18672436005263	13.66879265366641	4.61388514184169
H	5.45573483071688	13.51946327842500	5.42290581206634
H	6.88277692887136	14.46403722508771	4.92035235610732
H	6.75361196229125	12.73632484151951	4.47579269131794
C	4.60511770030765	15.29761741427955	3.52214349182086
H	4.12151769539853	15.58228674117539	2.57524545881124
H	5.22401914479098	16.14045709357163	3.86537063082285
H	3.82228086896660	15.10481844316369	4.26745793271638
C	1.53863008377580	9.28331552538272	1.74143420555738
C	-0.00819969942853	7.81135242028782	2.34444105376000
H	0.36756387901055	6.79209919918253	2.51916256203413
H	-1.06894925265921	7.77177498152386	2.06609222190109
C	0.28755827467824	8.76564039293181	3.52986209637932
C	-0.87239249031089	9.71997157789457	3.80000710088083
H	-0.57637743223134	10.46993575606777	4.54841055932237
H	-1.74257431177137	9.16475811916226	4.18130833997306
H	-1.16045452903240	10.24712173011312	2.87874496414950
C	0.70167943355704	8.01710797979190	4.78737756576669
H	1.58569173070750	7.39225514241556	4.59341410477298
H	-0.11852190157498	7.37189522500473	5.13613220461024
H	0.94445886329671	8.72941963228402	5.59035672180190
C	1.18023976397099	12.89058694869000	3.21032979146674
C	0.21785882806869	12.88286426299167	2.21751268168560
C	-0.77366191410782	13.85600240787855	2.12021336426381
C	0.11225424228728	14.92755966459957	4.05063953333797
C	1.08074257898222	13.93091967596748	4.11490882198150
C	-1.90238114614738	15.95225131080326	2.93847255915360
C	-2.19063664799212	16.56371649514264	1.71092371793256
N	-2.54582264560698	16.27369788462484	4.06628906604869

C	-3.18240115100300	17.53811612872742	1.66454086586515
C	-3.49041437677521	17.20964839254543	4.01189527869335
C	-3.85062100356174	17.87342397420281	2.83948783934850
H	-1.64068993624046	16.28026710404319	0.81251383132513
H	-3.42569053023463	18.03286689784777	0.72102065908391
H	-3.99647831669973	17.44737607268072	4.95476905229604
H	-4.63510580038081	18.63286129567091	2.85271820109774
F	4.54994295777227	9.71845593975913	3.83129113004978

TS2[axF,MI]

Electronic Energy (Def2-SVP) = -2412.95456194

Total enthalpy (Def2-SVP) = -2412.34191263

Gibbs Free Energy (Def2-SVP) = -2412.45363241

Electronic Energy (Def2-TZVP) = -2415.38174590687

Bi	3.14887589686545	11.37303651248511	4.00961294046263
F	-0.74756504260689	11.61142290560546	3.29038109112447
F	-1.39463232880769	13.64053115062114	1.72300078028163
F	1.87929654716338	16.32035576505906	3.85070905974891
F	2.44093431329604	14.30327213441795	5.48610238934836
O	4.20562325311003	14.81345460381718	1.26034680922785
O	0.54228973255613	8.63288807074033	1.64170342601763
N	4.18650238260670	13.46745809065401	3.03571135354494
N	1.74681476411919	9.48701961214539	3.30438581346894
C	0.20172734314484	15.06576251324423	2.73638334446838
C	2.35031468534662	11.76176280380948	1.91256811980406
C	2.69590696478522	12.91999895899848	1.22155828841587
C	2.12633596002611	13.19637452653818	-0.02750663756606
H	2.39522813691952	14.10825858880024	-0.56530875602019
C	1.21487944441762	12.29210491660792	-0.57687370794881
H	0.76248960805170	12.50487900376157	-1.54765419805281
C	0.89024861227894	11.10916579083331	0.09081327556580
H	0.19825431707768	10.39199603754433	-0.35621963465075
C	1.46731933040912	10.85179410181492	1.33915167500698
C	3.70480230263316	13.75366067132319	1.87762711395031
C	5.09481711957724	15.44380317017647	2.20713002488449
H	4.57427483485831	16.32429003012795	2.61348942429083
H	6.00289915924464	15.75492030431066	1.67600586699812
C	5.33367944345206	14.36070494162207	3.28271001213205
C	6.60726613516496	13.55970321659873	3.01730278841988
H	6.65026707353712	12.68646552966909	3.68478744045917
H	7.49627063920490	14.18454709651861	3.19150923072200

H	6.62999551234522	13.19663509766625	1.97857151189497
C	5.32508476414037	14.93361809830773	4.68894936688625
H	4.40223392129151	15.49615081089833	4.87667230720199
H	6.18790687600029	15.60248995975047	4.82568944077550
H	5.39688424082572	14.12301717254714	5.42921070541799
C	1.24448886841397	9.63957658955917	2.12868602557077
C	0.69673136547034	7.53584915649776	2.56810958065404
H	1.34793805583080	6.78852219065011	2.09070033768604
H	-0.29299652126019	7.10027911643659	2.75325323676039
C	1.33683462928343	8.17243799355376	3.82790825241666
C	0.31572985444467	8.36388375401978	4.94339354392924
H	0.76319109372858	8.95622629209052	5.75374237801765
H	-0.00080437813407	7.38536096055792	5.33506483694342
H	-0.57129027360718	8.89420324637410	4.56559258191540
C	2.55556047612305	7.40258016793010	4.31428329172876
H	3.32673008784460	7.35810764105036	3.53142649223995
H	2.27267369577597	6.37670857175235	4.59457801848079
H	2.98473412938811	7.89345155879043	5.20096661407771
C	0.89415026724032	12.87130787863931	4.44269583191772
C	-0.09316501289334	12.77200442495623	3.47591902729730
C	-0.43940813177479	13.82468694369175	2.63762256021514
C	1.18017290672909	15.19152565827206	3.73169203932823
C	1.47984550703966	14.12027991531591	4.56357224122955
C	-0.12592774213753	16.18818050819309	1.82563561658464
C	-0.25503413881997	15.97334709784219	0.44696519353842
N	-0.27751443522812	17.39582722566242	2.38056237427473
C	-0.54805851419162	17.05726365488616	-0.37431666501786
C	-0.55863701060240	18.42575862988728	1.58625200562417
C	-0.70396059265868	18.31473099125068	0.20336824282997
H	-0.12084389177084	14.97435618832910	0.03161001447662
H	-0.64875585790199	16.91908609512860	-1.45362544882443
H	-0.67827409839157	19.39989290784257	2.07424701931586
H	-0.93415204182300	19.19484942850408	-0.40049116965967
F	4.79312541481260	10.33781597374679	3.17432980919249
C	1.11889203554159	13.21223506303472	8.05169852854532
C	0.70217210102101	14.22889504211972	8.90863348467770
C	-0.58671893733001	14.75456786464101	8.79621939082828
C	-1.46154401695626	14.25421062772742	7.82777593751329
C	-1.04242553200372	13.23943326611207	6.97144995161734
C	0.25254454047239	12.71813533045089	7.07289436220122
C	0.69532542022124	11.60815809174328	6.16970919443897
O	1.73609150671976	10.95728329427468	6.41073966340634

H	1.38424163595483	14.61495871403009	9.67084446851773
H	-0.91330134584415	15.55282520097509	9.46776614298813
H	-2.47329851359872	14.65907740091220	7.74304373790858
H	-1.71900210202040	12.84760651366752	6.20521675540656
H	-0.15031126925313	11.12052936178418	5.64213146143954
H	2.12018682311042	12.78209028010063	8.12604237148586

INT2^{axF,MI}

Electronic Energy (Def2-SVP) = -2413.00508033

Total enthalpy (Def2-SVP) = -2412.38987269

Gibbs Free Energy (Def2-SVP) = -2412.50249097

Electronic Energy (Def2-TZVP) = -2415.43376092699

Bi	3.72458504865437	11.07083814229366	4.03684582854964
F	-1.12519754515209	12.26539186012694	3.13582670639773
F	-1.49117126186162	14.21130522264288	1.38856337979109
F	1.98928756105119	16.67794854230203	3.43233282363826
F	2.27302049481791	14.78663801589471	5.23231940932435
O	3.99870333537401	14.79656173288114	1.41290697201348
O	0.23228893228827	8.73884719753611	2.47982642088440
N	4.48117137585577	13.30425144603907	2.99920427067843
N	1.98349877090669	9.35422746820301	3.70666336886083
C	0.22054709001509	15.53802332461196	2.34236317112687
C	2.42621940824410	11.62445667397675	2.24185950739755
C	2.65538484996189	12.78902746739678	1.51260131078427
C	1.85586845654767	13.11234123776283	0.41071829543839
H	2.04192684698373	14.03174524937955	-0.14898855758479
C	0.81834245027775	12.25416525956206	0.04061672963695
H	0.18820966906281	12.50460430812196	-0.81517148270539
C	0.57359212919087	11.08683746089849	0.76302286033903
H	-0.24523663270445	10.42114536699010	0.48195218534817
C	1.38530296808511	10.77982302466033	1.86296605760961
C	3.74439937098181	13.63478641754198	2.00124717298504
C	5.00720343257698	15.44475585087555	2.21498206806102
H	4.51781866340967	16.26644375515166	2.75819046617912
H	5.77657131138444	15.84778564875844	1.54427658190318
C	5.52289436849971	14.33124359240406	3.16139928892326
C	6.85183157308971	13.75162617588702	2.68486709834291
H	7.12389415256031	12.87363071323394	3.28954152537249
H	7.65057051626916	14.50330843553042	2.77725915000174
H	6.78398392871891	13.44011657643473	1.63130283073021
C	5.59946714474716	14.79442237377810	4.60720403016564

H	4.62219366273401	15.16618227418872	4.94295162196590
H	6.34290709284629	15.59909006678141	4.71180614837659
H	5.90059984282950	13.95894723077063	5.25698664669542
C	1.21138925066057	9.59854743252707	2.70843302442355
C	0.40537627425155	7.65493313439227	3.41693369324507
H	0.71309251607051	6.76671868234943	2.84590042391586
H	-0.56052452544955	7.46485304650722	3.90212427797885
C	1.50398848345061	8.14927840339232	4.39599879586278
C	0.92643779395498	8.55092088329608	5.74975809356252
H	1.69163929226986	9.06136298625045	6.35227911199236
H	0.57847857561073	7.66107936678536	6.29568084821450
H	0.07667390154273	9.23758964288609	5.61940858123986
C	2.63945725967337	7.14742333288567	4.54445308633257
H	3.10640903843016	6.94652163727763	3.56914122626564
H	2.26332401336253	6.20108909409637	4.96136406551545
H	3.40859022003690	7.54315787260395	5.22469274715416
C	0.61432823107656	13.42599073994729	4.23210968321108
C	-0.34724489632804	13.34632585890423	3.23029634278491
C	-0.54247319109483	14.36890126645880	2.30840262024826
C	1.18873675262113	15.61956032040052	3.34956629537454
C	1.36153083430958	14.60194452875747	4.28121479594996
C	-0.01109533891815	16.65396289142830	1.39267844845428
C	-0.18515039301259	16.41643016615278	0.02322354127005
N	-0.05569979432007	17.88317415609959	1.91880896287301
C	-0.41745157587215	17.50106104443381	-0.81756336241558
C	-0.27492664097750	18.91314862132105	1.10598816254517
C	-0.46531342955608	18.78002542709787	-0.26944112050506
H	-0.13875124269674	15.40177138999948	-0.37339339275744
H	-0.55698144229194	17.34613365695219	-1.89016321028406
H	-0.30659843527934	19.90467566660055	1.57208544027483
H	-0.64689935529998	19.66004434353118	-0.88984458226885
F	4.91919223651459	9.83732463264245	2.70488726978500
C	0.88890446536102	12.88081341504516	7.66539085229484
C	0.25021748041506	13.19013273337935	8.86731367402172
C	-1.14282492303191	13.22169270343481	8.93365809714834
C	-1.89489134964370	12.94066697332402	7.79008566417229
C	-1.25494714832811	12.63448804929560	6.59034832109740
C	0.14282520623855	12.60479250894241	6.51908863761741
C	0.85668026891673	12.27258945529628	5.20922416747539
O	2.17989032035779	12.01220197538260	5.39921608244721
H	0.84481684537402	13.40522544646053	9.75954477392786
H	-1.64388202625118	13.46046040238060	9.87532910434681

H	-2.98712063126537	12.95639991897392	7.83533714736424
H	-1.84788848768092	12.41153893035073	5.69793532106087
H	0.31064388214491	11.40739784307369	4.77660034201573
H	1.97691021021656	12.84017777794624	7.58881491415049

5c^{eqAr}

Electronic Energy (Def2-SVP) = -2067.98411834

Total enthalpy (Def2-SVP) = -2067.49096004

Gibbs Free Energy (Def2-SVP) = -2067.59122057

Electronic Energy (Def2-TZVP) = -2070.05021230468

Bi	0.51369709874113	9.95734503709146	3.94698094115510
F	-0.36095164391888	10.36399487285176	0.41297171718350
F	-2.17837786640870	11.85232107714755	-0.79365677280584
F	-3.55807184528184	13.57903718190315	3.38914156584400
F	-1.68723923353645	12.12299277046594	4.58784035274857
O	2.14382163486326	12.05585672801302	-0.19069157843425
O	2.60340081272007	5.98269183154455	3.62038304143012
N	1.85831864818708	12.37697597878432	2.01342502560668
N	1.94613503370169	7.78087606055762	4.77658323466029
C	-2.94802671466860	12.76313167267066	1.24895028794987
C	2.11323557913143	9.40212991859897	2.35506381053735
C	2.47953791831278	10.14930390930911	1.22251660402995
C	3.25750821272896	9.57243630965886	0.20624270295244
H	3.51359034642697	10.17479693495051	-0.66697991225864
C	3.70747339360661	8.26140133961660	0.30911497104655
H	4.31030093681530	7.82323997686900	-0.48911799467070
C	3.40226335728032	7.52173526465583	1.44452411906586
H	3.76760361786799	6.49981883683532	1.56069890280159
C	2.62157318955552	8.09092654060664	2.45980232419046
C	2.12078484140092	11.56812521505784	1.06884447768195
C	1.64759210829562	13.39590565482234	-0.09653700057444
H	0.60917286215767	13.40177989982334	-0.46793984746499
H	2.26627166079568	14.04981078328575	-0.72555681998004
C	1.72529734351908	13.71492936325748	1.41383675000764
C	2.98882300522817	14.50175631564197	1.76177801326772
H	3.09749244576277	14.57818044667782	2.85389159888218
H	2.93870590709098	15.51744998481549	1.34027378068863
H	3.88247340474970	14.00013803127383	1.35941687399714
C	0.48601155524195	14.43416065145492	1.92209190429785
H	-0.42626726860764	13.89511596564611	1.63576651303527
H	0.43308663035222	15.45022205870797	1.50236156398471
H	0.51436538031966	14.51028464846177	3.01911721719186

C	2.37206001641496	7.29799068638949	3.67193116216407
C	2.40508632534686	5.48593356629412	4.95455933900615
H	3.39121767600478	5.23675123313242	5.37610576567566
H	1.79023853477740	4.57799520176864	4.89917041389046
C	1.72090802395731	6.65818554335454	5.70082996775223
C	0.21607019530151	6.43861768129311	5.84448106427715
H	-0.26485982898882	7.34022483736570	6.25250963983215
H	0.01619728364891	5.59747913663274	6.52573663031992
H	-0.23926894760862	6.21233911638064	4.86790817094246
C	2.36830738732296	6.93971229194398	7.04788433872954
H	3.44195827319480	7.14631973130289	6.92693831621546
H	2.25144541905072	6.07404848939860	7.71767595143975
H	1.89512292029808	7.81110086280454	7.52427404439232
C	-0.93744200474213	11.21047946202422	2.54998711736175
C	-1.12152598987261	11.15588617234701	1.17960562915458
C	-2.08295422776845	11.92766044049426	0.53387742186934
C	-2.79345660890187	12.79247691472681	2.63866180906411
C	-1.80330432394794	12.03116686548629	3.25176258869504
C	-3.98359206925874	13.57704049532861	0.56613811421004
C	-3.65518424593226	14.36071793134021	-0.54703925899101
N	-5.22152286025439	13.52244922045201	1.06832291950801
C	-4.65916059020970	15.10713515919264	-1.15625312425740
C	-6.17358066702949	14.24299669599927	0.48037760904834
C	-5.94890402788149	15.05054877935103	-0.63445651870959
H	-2.63032920922313	14.38607262540827	-0.92054741786901
H	-4.43345928378179	15.72992152812179	-2.02524401098622
H	-7.17669366412607	14.17514314958197	0.91676710370011
H	-6.76835806580542	15.61997654834664	-1.07778965902874
F	1.48533220758320	11.51104837267858	4.92009650454458

TS3^{eqAr,MI}

Electronic Energy (Def2-SVP) = -2412.94066359

Total enthalpy (Def2-SVP) = -2412.32815321

Gibbs Free Energy (Def2-SVP) = -2412.4410196

Electronic Energy (Def2-TZVP) = -2415.36819972589

Bi	-2.97457367653718	7.79067607901409	2.50831965256835
F	-3.87633416320862	10.32751525701996	0.67942306779440
F	-4.97366471108949	12.73814178171960	0.83419898912920
F	-8.00058849316786	11.13008431902224	4.09654985994289
F	-6.86947390612344	8.74143453667460	3.96645513314927
O	-0.33364987398436	10.23913886443864	5.39668667731749

O	-1.05724985298442	3.68717137850067	2.51900255101742
N	-2.05952565748805	9.71053726029837	4.07559919338022
N	-2.36898544415585	5.40335589235329	1.96354455349383
C	-6.53516405779178	12.02518711416459	2.46736192805023
C	-1.09369614370133	7.11710565215523	3.71084158157712
C	-0.43042564150730	7.98363505379854	4.57507414390213
C	0.69297935560635	7.56310607341592	5.30059214484971
H	1.19973325432623	8.25505373872723	5.97703231008179
C	1.15094428359373	6.25347669796617	5.15344882012807
H	2.02360112311299	5.91727802175466	5.71725686705507
C	0.50064777169908	5.36956969704960	4.29102984598933
H	0.85742471135193	4.34360137483465	4.17463530589585
C	-0.61806904908282	5.81335438908887	3.57481425407883
C	-0.98306399612814	9.33641840845291	4.67192670657140
C	-1.14564691593341	11.42432290017248	5.40123362397580
H	-0.50466168711303	12.28667847536395	5.17610491316382
H	-1.57331714576738	11.53668464636066	6.40898609537840
C	-2.22651593809528	11.15919485397631	4.32405209519175
C	-3.61369154913837	11.48124219049307	4.85556002436221
H	-4.37773098229344	11.28728978874639	4.09484170837506
H	-3.66881286102740	12.54434262660831	5.13570782511776
H	-3.84027367288794	10.87363749559746	5.74383576148564
C	-1.92336505836006	11.92158699006054	3.03659642935276
H	-0.89375581834565	11.72146688980330	2.70301133708860
H	-2.03221927146781	13.00413761890401	3.20335851357681
H	-2.60937131092143	11.62370802125516	2.23369204288180
C	-1.37553276308468	4.96570782248371	2.65245971777512
C	-1.92038867850323	3.14986573088203	1.49878668789876
H	-2.36271498619299	2.21904599472096	1.87729196990809
H	-1.30078999311978	2.93106340138014	0.61645454118662
C	-2.96333214156537	4.26679264965317	1.23423477802586
C	-3.08994327464431	4.57447898845765	-0.25061493581509
H	-3.78913580358429	5.40737891753306	-0.39909387560245
H	-3.46743364138676	3.69000038476601	-0.78645730305638
H	-2.11111739721872	4.84610390246088	-0.67359395500408
C	-4.31601048733493	3.93436360289898	1.85772961408460
H	-4.20452579039501	3.71522487042229	2.93054941532582
H	-4.75526103885827	3.05463870872663	1.36277483674615
H	-4.99418755730512	4.78955799158648	1.73990324908381
C	-5.29751704459352	9.43964471370686	2.34319574639908
C	-4.88484837009571	10.50363631023970	1.56458874935880
C	-5.45673003022371	11.76694762614230	1.61382980903261

C	-6.98171876005129	10.95691198844479	3.25520159933062
C	-6.35023940694103	9.71761349115730	3.19607312539911
C	-7.16701775632037	13.36501850076070	2.53420082058431
C	-6.37982612365635	14.51377788729236	2.68959572991368
N	-8.50044957160818	13.41565810598983	2.44163556656198
C	-7.00912386588252	15.75317426555486	2.74798326732231
C	-9.09273311052701	14.60644718268323	2.50167929938643
C	-8.39754469663453	15.80600859215860	2.65254789223228
H	-5.29475985656076	14.42951361307446	2.76901828359788
H	-6.42106797899942	16.66589363829822	2.87151967498702
H	-10.18599883086236	14.61243590613695	2.42226852598240
H	-8.93591495814928	16.75513732135842	2.69364524270126
F	-4.05695803810899	6.92398405915238	4.03888127664368
C	-6.60828085748052	8.38561100542367	0.15748047149951
C	-6.04188546444169	8.68333470151907	-1.08686115316597
C	-7.89900737373805	8.83779405425165	0.45906594370234
H	-5.03585207007164	8.32076427160609	-1.30871323093298
H	-8.33618198041596	8.60235418999279	1.43392670457182
C	-6.75311481213569	9.44280984476326	-2.01201850882033
C	-8.60917003656153	9.59847409550047	-0.46551551825007
H	-6.30821402622337	9.67996462094982	-2.98177339313246
H	-9.61220483953910	9.95804533558570	-0.22266780865771
C	-8.03438290226048	9.90578733760948	-1.70137827198697
C	-5.84564714567123	7.60173978341462	1.16254530479846
O	-4.79847084970053	6.99102890578246	0.86510909448378
H	-6.42553203211204	7.29931848791338	2.05551253189617
H	-8.58845385762795	10.50755122477289	-2.42635696791901

INT2^{eqAr,MI}

Electronic Energy (Def2-SVP) = -2412.98405703

Total enthalpy (Def2-SVP) = -2412.36912199

Gibbs Free Energy (Def2-SVP) = -2412.48113881

Electronic Energy (Def2-TZVP) = -2415.40942716893

Bi	-2.67175191433825	8.29202470388360	2.13499004203316
F	-4.65951236403015	10.41833419715604	-0.06898382033942
F	-5.01236165553493	12.85954449547437	0.82959155467981
F	-7.77070180274152	11.19696404072065	4.29106200869115
F	-7.43003566254144	8.74838540790742	3.37919354403070
O	-0.69125347565539	10.42413596178641	5.72279065946808
O	-1.44395853952274	3.97008132257306	2.56898682344330
N	-1.84706428767956	10.09196951797774	3.84317828436962

N	-2.39886327327671	5.84049169777451	1.83042141065947
C	-6.42955816569262	12.13933189408704	2.58188862461124
C	-1.34642042512035	7.36912553968815	3.83796963724539
C	-0.76548139484124	8.17027258721417	4.82323035449285
C	0.08569299399002	7.61729448607072	5.78943007614838
H	0.52723931666100	8.25318228044490	6.56055419750895
C	0.36583327996325	6.24950353234858	5.75690227213056
H	1.02710272578657	5.81424978212749	6.50919243271432
C	-0.18416559421021	5.43689073491297	4.76469480201224
H	0.04665147915916	4.36953074075428	4.72966780601763
C	-1.03459495362910	6.01164065772661	3.81077719859417
C	-1.11482892068890	9.59323087274885	4.77030146858804
C	-1.09300819944939	11.74363719368975	5.30495017677344
H	-0.20108363281258	12.26340264763610	4.92074456122753
H	-1.48749622906312	12.28030216905884	6.17705983106716
C	-2.14405194887697	11.48476143846325	4.20196437759285
C	-3.56610787275019	11.53019643663828	4.75690327039612
H	-4.27321380028917	11.15165843747566	4.00694570196105
H	-3.84612672980041	12.56053965053655	5.02278058901568
H	-3.65165285625695	10.89816309196250	5.65362746281495
C	-1.98265795041109	12.40592652545418	3.00404812858147
H	-0.97769487843611	12.30702084690241	2.56821966576168
H	-2.13305245167793	13.45438166274629	3.30312937912792
H	-2.72515567044888	12.16140033991854	2.22985409722977
C	-1.64803780072654	5.27134182768713	2.70656341763944
C	-2.03563474395339	3.59776909232624	1.30617625592291
H	-2.59141851141387	2.66225134610885	1.44698849735089
H	-1.21572719269902	3.43872669661006	0.58905559388996
C	-2.93022094627705	4.80257378103015	0.92969357246018
C	-2.77378535633275	5.22065078347700	-0.52306980059947
H	-3.37852176898127	6.12108332441110	-0.70061547591699
H	-3.12078074270287	4.41867280933539	-1.19210471428949
H	-1.72095605172916	5.44043259063080	-0.75474428770484
C	-4.39430016383553	4.55362518157874	1.28139118975923
H	-4.49258482005406	4.22211012953055	2.32665876503863
H	-4.81584468323680	3.77639650079479	0.62581523561396
H	-4.94951602148423	5.49117957204603	1.14711350931172
C	-6.02186077893576	9.47181155497969	1.62654317050205
C	-5.43067265349216	10.57076979638346	1.00607215007581
C	-5.62164105699073	11.86619326176787	1.47622023139738
C	-7.02755300378048	11.03767283113544	3.20198038482095
C	-6.82673279450543	9.74551076160136	2.72963655489839

C	-6.64325417457806	13.52174954374419	3.07421549908369
C	-5.56606017545769	14.40448850400120	3.22506983200296
N	-7.90203754836786	13.86943910618221	3.36211157730153
C	-5.81723851601639	15.69085500830806	3.69221137025193
C	-8.13120118850375	15.10145125072204	3.80970054222886
C	-7.12755088300203	16.05263237278475	3.99356745638624
H	-4.55206667651670	14.08432045516866	2.98481986313008
H	-4.99575136017245	16.39935984030966	3.82330053267368
H	-9.17417690226292	15.35293469014224	4.03412811784019
H	-7.37306147946499	17.05024030191382	4.36347059322897
F	-4.16670525115133	8.30856784114527	3.59280151515322
C	-6.70485120717283	7.81388208828557	-0.06232200239515
C	-6.17726818102606	7.82733850698389	-1.35530160766446
C	-8.07904379718943	7.60971102796821	0.10901812113696
H	-5.10237452160880	7.97793728493637	-1.47146883440212
H	-8.50060614583543	7.59423684499614	1.11895285641107
C	-7.00763681493422	7.64246137764029	-2.46144630116517
C	-8.91086699992981	7.42371330978776	-0.99440309869697
H	-6.58217598153678	7.65301862269854	-3.46877679210302
H	-9.98172182969839	7.25966296944368	-0.84663857925911
C	-8.37701403434289	7.43979835791671	-2.28510191272297
C	-5.79264244482933	8.03392585938830	1.14421567304379
O	-4.48885642944215	7.76185640679615	0.85414973549777
H	-6.18996577404554	7.39306884565768	1.95772388895421
H	-9.02762673395818	7.28991779381621	-3.15064290056942

TS1^ISO

Electronic Energy (Def2-SVP) = -2377.11904198

Total enthalpy (Def2-SVP) = -2376.51079279

Gibbs Free Energy (Def2-SVP) = -2376.62251943

Electronic Energy (Def2-TZVP) = -2379.28132029463

Bi	-0.06336304278895	-1.09483764226151	1.01921386745829
F	-1.34408643678071	0.55392025720399	-2.18588133983813
F	-2.64261160627484	2.77397739203052	-2.80587514650109
F	-2.55245253638388	4.10238533222080	1.74069582644786
F	-1.26107378183659	1.87678410588639	2.36113862511159
O	2.68034858772811	1.24746000574985	-2.01168451756125
O	-1.12775430467356	-4.83541225563414	-1.22355842518541
N	1.73483052516375	0.81430860031713	-0.02610609368429
N	-0.72383189038407	-3.50072709084688	0.52002264131658
C	-2.67046199580800	3.51411365889036	-0.55558964168521
C	0.62961211204228	-1.64104012615835	-1.05566771288660
C	1.43915300466787	-0.79718361405429	-1.82728389217037
C	1.82038750648634	-1.17382843260398	-3.12170216308696
H	2.45023020044548	-0.50321405411452	-3.70953998807511
C	1.40985621231995	-2.39739743442796	-3.65144629350847
H	1.71016975720579	-2.68231740140153	-4.66186623173674
C	0.63333546566649	-3.26109743192163	-2.88479497088099
H	0.32247960970123	-4.23154767559830	-3.27820177604044
C	0.25463976299784	-2.88296857856817	-1.58960499123944
C	1.93164949630097	0.44873686608647	-1.23520338219545
C	3.18848468246339	2.28026783512632	-1.15790153381595
H	3.03034085484580	3.25020828330247	-1.64839241669467
H	4.26865662718733	2.11279015570142	-1.02283299964181
C	2.39449653513895	2.11946236321425	0.16462497203591
C	3.31332428016845	2.09203572488039	1.37758237715806
H	2.72712407126912	1.94409505728110	2.29607050908325
H	3.85110689084712	3.04854644490556	1.46450787126747
H	4.05361146625309	1.28358852505913	1.29908984709369
C	1.34701965792414	3.21784350402617	0.32706306370189
H	0.65998289631709	3.23815793697570	-0.53142876173585
H	1.84590318411148	4.19592597120240	0.40690983497888
H	0.75707002051120	3.05029492636434	1.23738089859318
C	-0.53587807482640	-3.75122416094054	-0.72664334753752
C	-1.72443246051938	-5.51512353490961	-0.10351681895561
H	-1.10509015037961	-6.39578142681612	0.12769458176909
H	-2.73302235322333	-5.83872562619250	-0.39076098164339
C	-1.70613380794490	-4.46891911816419	1.03665178456797

C	-3.05443507514374	-3.76542207234957	1.18081354888872
H	-2.97913604266713	-2.94121668929179	1.90574196467696
H	-3.81758659678020	-4.47470036299437	1.53566237564974
H	-3.38263808119999	-3.35108490476065	0.21529302381917
C	-1.25133279818085	-5.06593643421351	2.35788943387448
H	-0.26695607830495	-5.54314366211418	2.25299049195144
H	-1.97348099583669	-5.82188047710822	2.70170446407679
H	-1.18119028531570	-4.28256311894126	3.12715316890180
C	-1.26466673924239	1.10753610840197	0.12245443308831
C	-1.63838724896165	1.40035634147096	-1.17089275039628
C	-2.31336279056000	2.56658267317851	-1.52298963802906
C	-2.30442766118456	3.22346561479264	0.76500871731649
C	-1.62796853703835	2.04533898550553	1.06220632444968
C	-3.40828705247017	4.74978944640075	-0.91062552464941
C	-3.00345681983474	5.54002304859884	-1.99577068113415
N	-4.46723929275578	5.06618947718784	-0.15539943681095
C	-3.72992223169770	6.68637082780692	-2.30036831283430
C	-5.15206068602113	6.16872959901191	-0.45138321040804
C	-4.83098184604574	7.01410104577184	-1.51267976350220
H	-2.13060844210544	5.25665572232427	-2.58574328660154
H	-3.43503548311331	7.31956724504011	-3.14088684614852
H	-6.01221262192720	6.39525334345055	0.18950230502208
H	-5.43108333120200	7.90434257021022	-1.71199551305737
Si	2.49163708139612	-1.95859818342088	1.75426227643263
C	2.33085083790724	-3.81691767062975	2.08077239570565
H	2.02895237587601	-4.35610535332110	1.16961167816906
H	3.30122814159249	-4.22025519688979	2.41907302733442
H	1.58127000655088	-4.02231107953405	2.85989987922969
C	2.98436156646386	-1.14620178984169	3.39009092815969
H	2.22962607390228	-1.35580888558980	4.16491299815166
H	3.95128792530767	-1.55012421128636	3.73641617203609
H	3.08254185619834	-0.05520372577881	3.29486047587594
C	3.88249316899771	-1.74936153950330	0.49098205710071
H	4.78234764570971	-2.26606380734064	0.86729208452860
H	3.60872719408121	-2.20012008448259	-0.47552319617098
H	4.13751189766684	-0.69484014157106	0.31650066102011

5h^{ax}

Electronic Energy (Def2-SVP) = -2377.12973478

Total enthalpy (Def2-SVP) = -2376.52035986

Gibbs Free Energy (Def2-SVP) = -2376.63350889

Electronic Energy (Def2-TZVP) = -2379.29261893492

Bi	3.04787524206977	11.94800272349868	4.27437100473034
F	0.71577215014973	12.40552800158082	1.06295396119326
F	-1.73630690920643	13.35231571597442	1.10385257408075
F	-0.39805664158790	16.74199479276827	4.13154629338097
F	2.04358981017704	15.73640902494817	4.17706952405720
O	6.54278737165800	12.87697015119921	1.65852186755648
O	0.44566632448820	9.01693386631183	2.11001153733691
N	5.14782678410435	12.87257726160257	3.39387604198289
N	1.23979910593936	10.34543444631208	3.71234740113648
C	-1.16620738619441	15.09081186079604	2.61131912832228
C	3.45767903036786	11.04739985929186	2.27285219041701
C	4.60898351680881	11.40752898048692	1.56944234158929
C	4.89379474217943	10.81943721143012	0.33016779331594
H	5.79469356567616	11.10425157474100	-0.21824467204562
C	4.01677904178683	9.86837693582483	-0.19382493466708
H	4.23491684901757	9.40598743480168	-1.15861975620770
C	2.86141490565457	9.50365711337669	0.50187542951314
H	2.17532965255981	8.76284039900886	0.08503110356597
C	2.58676575604323	10.09668271747852	1.73879354035747
C	5.44298375788321	12.40479131250653	2.23068041152185
C	7.19280196295952	13.69480848893939	2.65446351671613
H	7.55828350815365	14.60790375631807	2.16846298145423
H	8.04133647837310	13.11997702189187	3.05658811931934
C	6.09838879989291	13.94890005439832	3.71704678986943
C	6.61428960898696	13.81192339770132	5.13971310152349
H	5.78725489116789	13.93434514214173	5.85529305132965
H	7.36683009963981	14.58656898891465	5.34950077425168
H	7.07236227678698	12.82534436485706	5.29945499835221
C	5.40434792008748	15.29148477384681	3.49989814100033
H	5.07937272512151	15.39366077518325	2.45320313761526
H	6.09386580629611	16.11628681724365	3.73560052909299
H	4.51432230764199	15.37623313235648	4.13878991411849
C	1.40637547365264	9.81884617213869	2.54983660779288
C	-0.48588731671907	8.86420428320962	3.20076481610381
H	-0.34687229607384	7.85721295999697	3.62297716552205
H	-1.50347029102894	8.96075474913114	2.80193174583234
C	-0.10149906349305	9.98380407042879	4.19867688050807
C	-1.01907002595610	11.19617197283455	4.05741055160303
H	-0.62750948951702	12.04554768443038	4.63698953638152
H	-2.02700738609346	10.95310067983768	4.42643362695218
H	-1.09525241350565	11.50406676386359	3.00515999182486

C	-0.04667402755799	9.49389156837400	5.63602856106304
H	0.63593696922389	8.63769201269127	5.73363479566492
H	-1.04766166369864	9.18341778404205	5.97086746195503
H	0.30262580499086	10.29961355458592	6.29923808128476
C	1.53003476878032	14.04384261006842	2.58834585342228
C	0.50454467451397	13.50738769078984	1.84064863695306
C	-0.80072783857724	13.98793567884033	1.83092019969909
C	-0.14458975320768	15.65832668886257	3.38606217360267
C	1.14052542169702	15.12112025172389	3.35581287256718
C	-2.54848093575255	15.62211586749359	2.61724724444992
C	-3.24088738240480	15.82345158873901	1.41372312456866
N	-3.09741112621933	15.90170155600689	3.80636014223663
C	-4.53498745404411	16.33027324148836	1.45673865251480
C	-4.33484257840517	16.39181537677355	3.83624714433876
C	-5.10066645175078	16.62603130847906	2.69508809017758
H	-2.76240847072028	15.58976757322704	0.46157644957543
H	-5.09240746661584	16.49923591416358	0.53191961912959
H	-4.74886023151834	16.60951437243927	4.82792475881385
H	-6.11212584623515	17.02881825919709	2.77943901977073
Si	4.57428232828715	9.79802690735255	5.11626547473664
C	3.70789511476127	8.17478858353129	4.69930253585760
H	3.53455916507426	8.08278084929366	3.61578606832817
H	4.34305411605343	7.33002357060005	5.01591427763141
H	2.73847357646402	8.09001200167151	5.21164449509486
C	4.78834159981394	9.94256493179835	6.98681111526612
H	3.81179151602253	9.92471997449080	7.49635114263383
H	5.39440739757712	9.10575370256394	7.37348847648679
H	5.29413584279845	10.88485780339245	7.25157166938778
C	6.27256802096295	9.77964200741629	4.29263545644744
H	6.83572441637259	8.90234802600088	4.65416403279143
H	6.18586820753168	9.69937712292882	3.19802913541529
H	6.85450655383274	10.68310213436435	4.52672902883133
